# A Telescoped Strategy for the Preparation of Five‐Membered Hetero‐ and Carbocycles via Hydrogen Atom Transfer Photocatalysis in Flow

**DOI:** 10.1002/cssc.202501012

**Published:** 2025-07-12

**Authors:** Filippo Sacchelli, Elena Quadri, Luna Raineri, Alexandra Jorea, Marzia Pessina, Anna Lo Presti, Nicola Della Ca’, Davide Ravelli, Luca Capaldo

**Affiliations:** ^1^ SynCat Lab Department of Chemistry Life Sciences and Environmental Sustainability University of Parma Parco Area delle Scienze 17/A 43124 Parma Italy; ^2^ PhotoGreen Lab Department of Chemistry University of Pavia viale Taramelli 12 27100 Pavia Italy; ^3^ CIRCC (Interuniversity Consortium Chemical Reactivity and Catalysis) via Celso Ulpiani 27 70126 Bari Italy

**Keywords:** flow chemistry, Hunsdiecker condensation, Paal–Knorr synthesis, photocatalyzed hydrogen atom transfer, tetrabutylammonium decatungstate

## Abstract

Five‐membered heterocycles such as pyrroles and thiophenes, along with cyclopentenones, are key scaffolds in pharmaceutical chemistry. However, the synthesis of these motifs via diversity oriented synthesis is hindered by the limited accessibility of unsymmetrical 1,4‐diketones. Herein, a practical and sustainable flow protocol is presented for the telescoped synthesis of pyrroles, thiophenes, and cyclopentenones. The process begins with decatungstate‐photocatalyzed hydrogen atom transfer from aldehydes to generate acyl radicals, which undergo regioselective addition to functionalized enones. The resulting diketones are then fed into a second reactor—tubular or packed‐bed—to undergo Paal–Knorr or Hunsdiecker condensation reactions. This modular strategy enables rapid access to densely substituted, biorelevant hetero‐ and carbocycles with unconventional substitution patterns, as demonstrated by the synthesis of tri‐, tetra‐, and pentasubstituted pyrroles and thiophenes, and mono‐, di‐, and trisubstituted cyclopentenones.

## Graphical abstract

Pyrroles, thiophenes, and cyclopentenones are vital in drug discovery, yet their synthesis via diversity oriented synthesis is limited. Herein, a modular platform is developed for the scalable, telescoped synthesis of named structures in continuous flow by streamlining photocatalyzed hydrogen atom transfer and Paal–Knorr or Hunsdiecker condensation reactions.

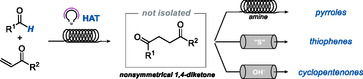

## 1 Introduction

**Scheme 1:**
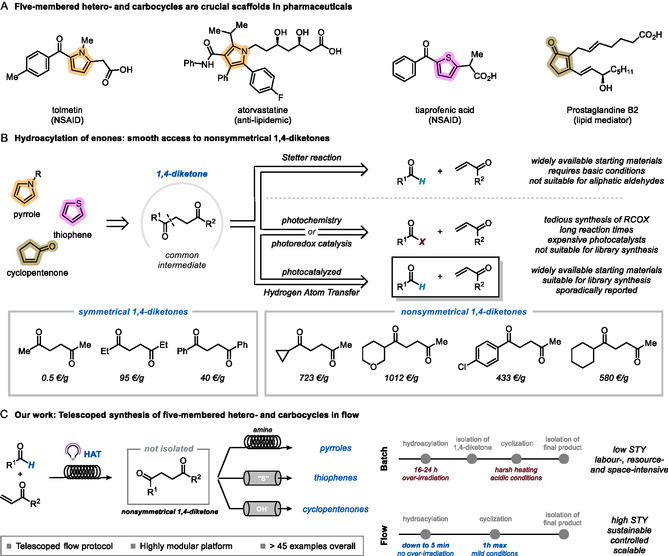
Context and background: A) Five‐membered heterocycles and carbocycles are appealing scaffolds for the pharmaceutical industry. B) The hydroacylation of enones provides smooth access to nonsymmetrical 1,4‐diketones, an important value‐added scaffold for medicinal chemists. C) Our work: a telescoped platform for the modular synthesis of five‐membered heterocycles and carbocycles. Prices calculated as of May 6, 2025.^[^ ^49^ ^]^

Five‐membered heterocycles, such as pyrroles^[^ ^1^ ^]^ and thiophenes,^[^ ^2^ ^]^ represent cornerstone scaffolds in organic chemistry. They hold profound significance in the pharmaceutical industry, as they are present in a range of bioactive derivatives, spanning from nonsteroidal anti‐inflammatory drugs to anti‐lipidemic active principles (**Scheme** **1**A). Cyclopentenones are highly valuable pharmaceutical motifs as well, constituting the core framework of prostaglandins,^[^ ^3^ ^]^ essential mediators in various physiological and pathological processes (Scheme 1A). Given the ubiquity of such cyclic structures in bioactive compounds and natural products, there have been strides toward the design of efficient strategies for their synthesis.^[^ ^4^, ^5^, ^6^ ^]^ However, approaches for the rapid and modular preparation of all these five‐membered hetero‐ and carbocycles are still an urgent need, as this would facilitate the discovery of novel therapeutics and drug candidates with enhanced pharmacological properties.

From a retrosynthetic perspective, pyrroles, thiophenes, and cyclopentenones can be traced back to a common precursor: 1,4‐diketones (Scheme 1B). On one side, the Paal–Knorr (PK) reaction enables the formation of five‐membered heterocycles by condensing such dicarbonyl compounds, respectively, with a primary amine to yield pyrroles^[^ ^7^, ^8^ ^]^ and with P_4_S_10_ or the Lawesson reagent^[^ ^9^ ^]^ to produce thiophenes. On the other side, cyclopentenones are efficiently synthesized from 1,4‐diketones via Hunsdiecker condensation (HC).^[^ ^10^, ^11^ ^]^

Unfortunately, 1,4‐diketones are generally not commercially available, and their synthesis, especially of nonsymmetrical ones, can be a daunting task.^[^ ^12^ ^]^ This severely limits the creativity of medicinal chemists in generating libraries of hetero‐ and carbocycles for new generations of pharmaceuticals via the so‐called diversity oriented synthesis (DOS) tactic.^[^ ^13^ ^]^ Accordingly, the development of an expedited synthetic approach to access structurally diverse 1,4‐diketones is crucial. The hydroacylation of enones is a convenient approach to this end (Scheme 1B). The venerable Stetter reaction is the predominant strategy in this domain, where a cyanide ion^[^ ^14^ ^]^ or a *N*‐heterocyclic carbene^[^ ^15^, ^16^ ^]^ is exploited to facilitate the addition of an aldehyde to an enone via the intermediacy of an acyl anion equivalent. Despite the impact of this transformation, a major limitation stands out: it demands basic conditions that are not tolerated by aliphatic aldehydes, which undergo benzoin‐type or self‐condensation in these conditions. This has introduced a strong bias for aryl‐substituted 1,4‐diketones.^[^ ^17^ ^]^

Shifting from polar to radical reactivity, state of the art methods rely on the light‐induced radical addition of acyl radicals to enones.^[^ ^18^, ^19^, ^20^, ^21^, ^22^ ^]^ Several tactics have been proposed for the generation of the requisite acyl radical intermediates,^[^ ^23^, ^24^ ^]^ however, they often rely on *ad hoc* engineered substrates (e.g., Hantzsch ester‐containing ketones, phenylglyoxylic acid, acylsilanes, anhydrides). As a result, this may discourage the adoption of such elegant strategies for the fast generation of libraries of 1,4‐diketones and, consequently, limit the access to an expanded range of structural diversity in the synthesis of five‐membered hetero‐and carbocycles.

A more broadly applicable—albeit only sporadically reported—approach involves the utilization of commercially available bulk chemicals such as aldehydes via a photocatalyzed hydrogen atom transfer (HAT) manifold, which results in a 100% atom‐economical protocol for the radical hydroacylation of enones.^[^ ^25^, ^26^, ^27^, ^28^, ^29^ ^]^ Merging such a tactic with the PK or HC reaction represents an exceptionally powerful strategy for medicinal chemists in DOS campaigns to access functionally rich molecular architectures and advance structure–activity relationship studies for hetero‐ and carbocycles.

Flow chemistry has become a powerful tool for synthetic and medicinal chemists,^[^ ^30^, ^31^, ^32^ ^]^ particularly in photochemical endeavors,^[^ ^33^ ^]^ enabling precise control over reaction selectivity across a wide range of transformations. This is achieved by minimizing the optical path length of the reaction mixture and allowing fine‐tuned adjustment of reaction time through modulation of flow rates. We hypothesized that these advantages could be harnessed to develop a streamlined platform^[^ ^34^ ^]^ for the on‐demand synthesis of five‐membered hetero‐ and carbocycles, achieving high combinatorial efficiency in the generation of these valuable structures. Remarkably, although extensively investigated in batch,^[^ ^35^, ^36^, ^37^ ^]^ to the best of our knowledge the translation of the PK and HC reactions into flow conditions has been relatively overlooked, despite its synthetic potential.^[^ ^38^, ^39^, ^40^ ^]^

Herein, we present the successful implementation of this approach in flow (Scheme 1C). In the first step, an acyl radical is generated from commercially available aldehydes^[^ ^26^ ^]^ via decatungstate‐photocatalyzed HAT in a tubular flow photoreactor.^[^ ^41^ ^]^ The resulting acyl radical is efficiently trapped by enones to yield a wide variety of nonsymmetrical 1,4‐diketones in a short reaction time (down to 5 min). In the second step, the dicarbonyl compound is directly introduced into a second tubular or packed‐bed reactor for the synthesis of tri‐, tetra‐, and pentasubstituted pyrroles and thiophenes. Moreover, mono‐, di‐, and trisubstituted cyclopentenones can be seamlessly prepared by flowing the dicarbonyl intermediate through a packed‐bed reactor filled with basic resin.

## 2 Results and Discussion

### 2.1 Telescoped Synthesis of Pyrroles

Our studies commenced with the decatungstate‐enabled hydroacylation of methyl vinyl ketone (MVK) using heptaldehyde as the H‐donor (see Section 5.1 in the Supporting Information). Following a careful optimization of different reaction parameters, we found that the photocatalytic radical addition performed optimal in continuous‐flow using a 3D‐printed reactor^[^ ^41^ ^]^ (U‐Flow, FEP capillary, ID: 0.8 mm; V_1_ = 2.5 mL) equipped with a Kessil lamp (*λ* = 390 nm, full intensity). The expected diketone, that is, undecane‐2,5‐dione, was obtained in 75% ^1^H‐NMR yield (CH_2_Br_2_ as internal standard) when a CH_3_CN solution of the enone (0.1 M), heptanal (1 equiv.) and tetrabutylammonium decatungstate (TBADT, (*n*Bu_4_N)_4_[W_10_O_32_], 4 mol%) as the photocatalyst was irradiated for 5 min (flow rate = 0.50 mL min^−1^, τ_R1_ = 5 min). This corresponds to a space‐time yield (STY) of the 1,4‐diketone of 900 mmol L^−1^ h^−1^ and is a remarkable improvement compared to a batch precedent (2.5 mmol L^−1^ h^−1^)^[^ ^26^ ^]^ and a flow precedent for a related compound^[^ ^42^ ^]^ (31 mmol L^−1^ h^−1^).^[^ ^27^ ^]^

Next, we optimized the second step of our telescoped approach, namely the conversion of undecane‐2,5‐dione to pyrrole **1** upon reaction with ethanolamine (see Section 5.2 in the Supporting Information). We found that combining a 0.1 M CH_3_CN solution of undecane‐2,5‐dione (flow rate = 0.50 mL min^−1^) with a feed containing ethanolamine and PTSA (1 and 0.1 M, respectively, in CH_3_CN; flow rate = 0.20 mL min^−1^) via a PEEK T‐mixer allowed to readily obtain the desired pyrrole in quantitative ^1^H‐NMR yield by using a tubular reactor (FEP capillary, V_2_ = 15 mL, ID: 0.8 mm, T = 60 °C, τ_R2_ = 21.4 min). The outflow of the reactor was collected in a saturated solution of ammonium chloride for quenching.

**Scheme 2:**
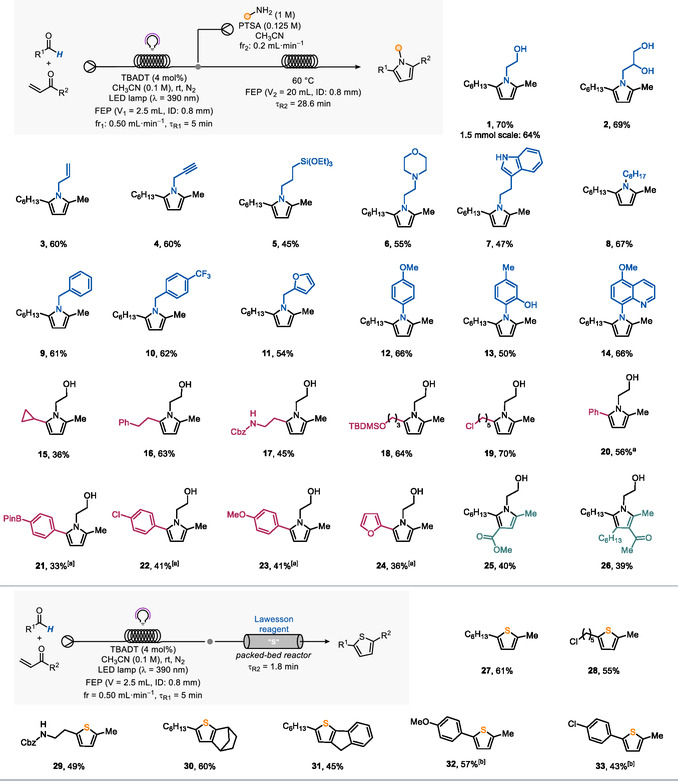
Scope for the telescoped on‐demand synthesis of pyrroles (top) and thiophenes (bottom) in flow. All yields refer to the isolated heterocycle after two steps on a 0.1 mmol scale. Pyrroles were prepared according to GP1; thiophenes were prepared according to GP2 (see Sections 6.1 and 6.2 in the Supporting Information). [a] τ_R1_  = 10 min, fr_1 _= 0.25 mL min^−1^, fr_2_ = 0.1 mL min^−1^, τ_R2_ = 43 min; [enone] = 0.12 M, [amine] = 1.25 M, and [PTSA] 0.25 M in CH_3_CN. [b] [enone] = 0.12 M, fr_1_ = 0.25 mL min^−1^, τ_R1_ = 10 min, τ_R2_ = 3.6 min.

Eventually, we streamlined the two optimized steps; however, we noticed incomplete conversion of the diketone in the second step, possibly due to interference of the photocatalyst. By simply increasing τ_R2_ to 28.6 min (V_2_ = 20 mL) and adjusting the concentration of PTSA to 0.125 M, we managed to produce **1** in 70% yield over two steps, corresponding to a productivity of 2.1 mmol h^−1^ and an STY of 93.3 mmol L^−1^ h^−1^ (**Scheme** **2**). We demonstrated the scalability of the approach by performing the synthesis of **1** on 1.5 mmol scale with comparable yield.

With optimized conditions for the telescoped synthesis of pyrroles, we turned to evaluate the generality of the approach (Scheme 2; see also Section 5.3 in the Supporting Information). At first, we evaluated the impact of using different amines, both aliphatic (**1**–**11**) and aromatic ones (**12**–**14**), on the telescoped procedure. Our conditions for the PK reaction in continuous flow proved to have a great functional group tolerance. For example, free hydroxy groups were well tolerated (**1**–**2**, 70% and 69%, respectively), as well as a terminal alkene (**3**), an alkyne (**4**), and a silyl ether (**5**), enabling space for maneuver for downstream derivatization. Biorelevant cores, such as morpholine (**6**) and tryptamine (**7**), did not interfere with the process. When benzylic amines were used for the Paal–Knorr step, the corresponding pyrroles (**9**–**11**) were obtained in good to excellent yields (54%–61%) over two steps. Finally, electron‐rich anilines were employed in our protocol, delivering **12**–**14** in serviceable yields (>50%).

Next, we focused our attention on the aldehyde employed in the hydroacylation step, which allowed to unlock a remarkable structural variety on the 2‐position of pyrroles. In fact, several aliphatic aldehydes were employed (**15**–**19**), demonstrating an excellent functional group tolerance. These results are remarkable, since they prove that benzylic, α–to–N, and α–to–O positions remain untouched in the HAT step when a formyl group is present as a H‐donor group (**17**–**18**).^[^ ^43^ ^]^ Moreover, a chlorine group was also well accommodated onto the pyrrole (**19**), working as a handle for late‐stage reaction with nucleophiles. By doubling the residence time of the first step (see variant of GP1 in the Supporting Information) to account for the lower reactivity of aromatic aldehydes in the HAT step,^[^ ^44^ ^]^ we also managed to extend our platform to the synthesis of 2‐aryl‐substituted pyrroles (**20**–**24**).

In the attempt to challenge our strategy, we replaced methyl vinyl ketone with densely functionalized enones. This allowed us to prepare densily substituted pyrroles (**25** and **26**) in a single streamlined fashion in about 30 min, which would be unattainable with conventional batch synthesis.

### 2.2 Telescoped Synthesis of Thiophenes

Regarding the synthesis of thiophenes in flow, no precedents have been reported in the literature to the best of our knowledge. We hypothesized that a packed‐bed reactor containing a suitable solid sulfur source would be an effective strategy to address this gap. The Lawesson's reagent (LR) was selected as the packing material,^[^ ^45^ ^]^ and the reaction conditions were optimized by studying the telescoped synthesis of thiophene **27** (Sections 5.4 and 5.5 of the Supporting Information). Thus, the outflow from the photochemical setup employed for pyrroles was passed through a cartridge (ID = 7.8 mm, L: 100 mm) packed with LR. Upon optimizing the cartridge loading, a 61% overall yield over two steps was achieved, corresponding to a maximum productivity of 1.8 mmol h^−1^ and a STY of 538 mmol L^−1^ h^−1^.

Our approach could be embraced for the preparation of pharmaceutically appealing^[^ ^46^ ^]^ thiophenes **27**–**33** in good to excellent yields over two steps. Intriguingly, enones featuring exocyclic C=C double bonds could be seamlessly used to build complex fused cyclic structures **30** and **31** in 60% and 45% yield, respectively. Finally, aromatic aldehydes could be exploited as well in the photochemical step, as exemplified by compounds **32** and **33**.

### 2.3 Telescoped synthesis of cyclopentenones

**Scheme 3:**
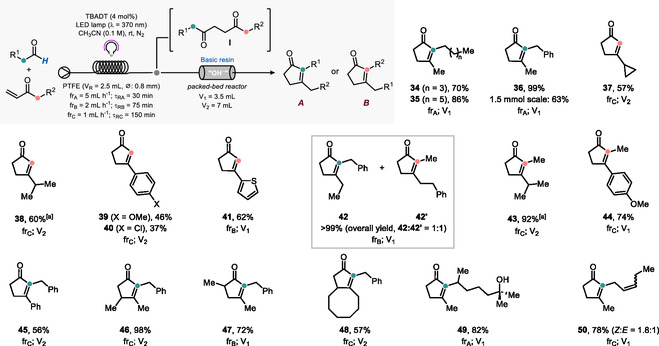
Scope of the modular synthesis of cyclopentenones in flow. All reactions were performed on a 0.5 mmol scale. All yields refer to the isolated carbocycle after two steps. [a] Photocatalytic hydroacylation step performed at 0 °C.

Next, we shifted our focus to the telescoped synthesis of the cyclopentenone core via our streamlined approach. For the sake of clarity, key positions of the starting aldehyde and enone backbones in the 1,4‐diketone intermediate **I** and in the final product are highlighted in orange and green, respectively (**Scheme** **3**). Thus, the feasibility of the cyclization process of interest requires the presence of an alpha‐methylene/methyl group to one of the carbonyls (see orange/green circles in **I**), which initially undergoes deprotonation to give an enolate prone to trigger the intramolecular aldol condensation involved in the HC. Based on the actual structure of the diketone intermediate, two different cyclization modes can be envisaged, wherein either the aldehyde or the enone carbonyl group is retained in the final cyclopentenone unit (see structures ***A*** and ***B*** in Scheme 3 and Scheme S1 in the Supporting Information). From the experimental standpoint, for this application we adopted a tubular photoreactor (PTFE capillary, ID: 0.8 mm; V = 2.5 mL) mounted on an aluminum support, which enables to control the temperature of the photocatalytic step thanks to a dedicated heat sink module, and equipped with a Kessil lamp (*λ* = 370 nm, full intensity; see Section 4 in the Supporting Information for details). The outlet of the photoreactor containing the 1,4‐diketone was connected to a packed‐bed reactor containing a basic resin (macroporous anionic resin AG‐MP1, hydroxide form) to promote the cyclization step. Upon careful consideration of reaction parameters, including flow rate and volume of the second reactor, we managed to find the optimal conditions for the synthesis of a diverse array of carbocycles (Scheme 3). Depending on the actual aldehyde/enone combination, three different flow rates (fr) have been employed, namely 5, 2, and 1 mL h^−1^ (tagged as A, B, and C, respectively), as well as two different versions of the packed‐bed reactor (V_1_ and V_2_), characterized by 3.5 and 7 mL volume, respectively (see Sections 5.6 and 6.3 of the Supporting Information).

Thus, when we combined primary aliphatic aldehydes with MVK, cyclopentenones **34**–**36** were selectively obtained in up to quantitative yield after isolation (70%–99%). Such carbocycles arise from a selective HC promoted by the enolate obtained through deprotonation at the methylene group, in line with previous literature reports.^[^ ^47^ ^]^ Gratifyingly, we managed to obtain an excellent overall yield for product **36**, namely a 99% yield over two steps, corresponding to a productivity of 0.5 mmol h^−1^ and a STY of 82.5 mmol L^−1^ h^−1^. Also, here, we demonstrated the scalability of the approach by performing the synthesis of **36** on 1.5 mmol scale with good yield. We next explored secondary aliphatic aldehydes, containing a methine (CH) next to the carbonyl, with MVK. While the hydroacylation with cyclopropanecarbaldehyde could be performed at room temperature, an ice bath was adopted in the case of isobutyraldehyde to work at 0 °C and avoid any competitive thermal decarbonylation pathway.^[^ ^48^ ^]^ With these starting materials, carbocycles **37** and **38** were selectively obtained in good yields (57%–60%; the adoption of the larger packed‐bed reactor was required to obtain complete conversion of the 1,4‐diketone to the desired cyclopentenone) through deprotonation at the terminal methyl (CH_3_; from MVK) and ensuing HC‐type cyclization. (Hetero)aromatic aldehydes were likewise competent substrates, delivering carbocycles **39**–**41** in up to 62% yield, following the same cyclization pattern observed for secondary aliphatic aldehydes.

Next, we explored the reactivity of ethyl vinyl ketone (EVK) with a diverse set of aldehydes. With primary hydrocinnamaldehyde, a 1:1 mixture of **42** and **42′** is formed, resulting from the equally favorable deprotonation and ensuing cyclization at the two methylene groups present in the 1,4‐diketone intermediate. A clean reaction was observed instead with secondary aliphatic (hydroacylation realized at 0 °C) and aromatic aldehydes, with adducts **43** and **44** formed in excellent yields through deprotonation of the available methylene group (from EVK).

Neither the aromatic ring present in phenyl vinyl ketone, nor the presence of substituents on the α‐ or β‐position of the enone double bond, had a major impact on the reaction course, as testified by the successful preparation of cyclopentenones **45**–**48**.

Finally, we adopted the fragrance ingredient 7‐hydroxycitronellal in our synthetic strategy and combined it with MVK to prepare **49** in an excellent 82% yield. At the same time, we prepared jasmone **50** starting from cis‐4‐heptenal and MVK; while the reaction was successful, delivering the hoped for product in 78% yield, the photochemical step caused partial isomerization of the double bond (a *Z*:*E* = 1.8:1 mixture has been isolated for **50**, as confirmed by ^1^H‐NOESY analysis; see Section 9.4 of the Supporting Information for further details).

## 3 Conclusion

In conclusion, we have developed a practical and flexible methodology for the telescoped synthesis of five‐membered hetero‐ and carbocycles in flow. Our approach combines HAT photocatalysis with the Paal–Knorr reaction for the synthesis of pyrroles and thiophenes, and with the Hunsdiecker condensation for the synthesis of cyclopentenones. Central to the success of this strategy is the rapid preparation of unsymmetrical 1,4‐diketones—valuable intermediates that are typically scarce and costly—achieved in as little as 5 min. Longer reaction times have been typically employed in the preparation of cyclopentenones to ensure complete conversion of the 1,4‐diketone intermediate into the carbocycle of interest. Nevertheless, our decatungstate‐based photocatalytic system demonstrated a high level of flexibility, allowing for fine‐tuning the overall flow rate (a single stream is used in this application) to improve the performance of the second step, without affecting 1,4‐diketone formation.

This streamlined protocol brings several advantages in terms of sustainability. First, the required diketones are formed in a single 100% atom‐economical step, where efficient irradiation is achieved according to the Beer–Lambert law, and reactants are used in a 1:1 stoichiometry. Second, STY values are higher compared to the batch manifold, suggesting the potential for process intensification: we proved this point by scaling the protocol up to 1.5 mmol. Third, the process does not require the purification of intermediates, thus positively impacting the sustainability of the overall process.

Thanks to its flexibility, we expect our approach to serve as an enabling platform for the medicinal chemistry community to smoothly access named compounds according to a DOS approach.

## Conflict of Interest

The authors declare no conflict of interest.

## Supporting information

Supplementary Material

## Data Availability

The data that support the findings of this study are available from the corresponding author upon reasonable request.

## References

[1] B. H. Ganesh , A. G. Raj , B. Aruchamy , P. Nanjan , C. Drago , P. Ramani , ChemMedChem 2024, 19, e202300447. 37926686 10.1002/cmdc.202300447

[2] R. M. D. da Cruz , F. J. B. Mendonça‐Junior , N. B. de Mélo , L. Scotti , R. S. A. de Araújo , R. N. de Almeida , R. O. de Moura , Pharmaceuticals 2021, 14, 692. 34358118 10.3390/ph14070692 PMC8308569

[3] N. A. Nelson , J. Med. Chem. 1974, 17, 911. 4415344 10.1021/jm00255a001

[4] J. Jose , T. V. Mathew , Tetrahedron 2024, 150, 133747.

[5] V. Estévez , M. Villacampa , J. C. Menéndez , Chem. Soc. Rev. 2014, 43, 4633. 24676061 10.1039/c3cs60015g

[6] R. Mancuso , B. Gabriele , Molecules 2014, 19, 15687. 25268722 10.3390/molecules191015687 PMC6271676

[7] C. Paal , Ber. Dtsch. Chem. Ges. 1885, 18, 367.

[8] L. Knorr , Ber. Dtsch. Chem. Ges. 1884, 17, 1635.

[9] G. Minetto , L. F. Raveglia , A. Sega , M. Taddei , Eur. J. Org. Chem. 2005, 2005, 5277.

[10] H. Hunsdiecker , Ber. Dtsch. Chem. Ges. 1942, 75, 447.

[11] Z. Wang , Hunsdiecker Condensation, in Comprehensive Organic Name Reactions and Reagents, John Wiley & Sons, Inc., Hoboken, NJ 2010, pp. 1508–1510.

[12] M. Lemmerer , M. Schupp , D. Kaiser , N. Maulide , Nat. Synth. 2022, 1, 923.

[13] W. R. J. D. Galloway , A. Isidro‐Llobet , D. R. Spring , Nat. Commun. 2010, 1, 80. 20865796 10.1038/ncomms1081

[14] H. Stetter , M. Schreckenberg , Angew. Chem. Int. Ed. 1973, 12, 81.

[15] H. Stetter , Angew. Chem. Int. Ed. 1976, 15, 639.

[16] D. A. DiRocco , K. M. Oberg , D. M. Dalton , T. Rovis , J. Am. Chem. Soc. 2009, 131, 10872. 19722669 10.1021/ja904375q PMC2747345

[17] M. M. Heravi , V. Zadsirjan , K. Kafshdarzadeh , Z. Amiri , Asian J. Org. Chem. 2020, 9, 1999.

[18] L. Capaldo , R. Riccardi , D. Ravelli , M. Fagnoni , ACS Catal. 2018, 8, 304. 10.1021/acscatal.0c02250 PMC8009479 33815891

[19] J.‐J. Zhao , H.‐H. Zhang , X. Shen , S. Yu , Org. Lett. 2019, 21, 913. 30694064 10.1021/acs.orglett.8b03840

[20] G.‐Z. Wang , R. Shang , W.‐M. Cheng , Y. Fu , Org. Lett. 2015, 17, 4830. 26366608 10.1021/acs.orglett.5b02392

[21] G. Goti , B. Bieszczad , A. Vega‐Peñaloza , P. Melchiorre , Angew. Chem. Int. Ed. 2019, 58, 1213. 10.1002/anie.201810798 PMC6468318 30419156

[22] T. Morack , C. Muck‐Lichtenfeld , R. Gilmour , Angew. Chem. Int. Ed. 2019, 58, 1208. 10.1002/anie.201809601 30427568

[23] A. Banerjee , Z. Lei , M.‐Y. Ngai , Synthesis 2019, 51, 303. 31057188 10.1055/s-0037-1610329 PMC6497162

[24] C. Raviola , S. Protti , D. Ravelli , M. Fagnoni , Green Chem. 2019, 21, 748.

[25] F. A. Macias , J. M. G. Molinillo , I. G. Collado , G. M. Massanet , F. Rodriguez‐Luis , Tetrahedron Lett. 1990, 31, 3063.

[26] S. Esposti , D. Dondi , M. Fagnoni , A. Albini , Angew. Chem. Int. Ed. 2007, 46, 2531. 10.1002/anie.200604820 17318938

[27] F. Bonassi , D. Ravelli , S. Protti , M. Fagnoni , Adv. Synth. Catal. 2015, 357, 3687.

[28] Y.‐L. Liu , Y.‐J. Ouyang , H. Zheng , H. Liu , W.‐T. Wei , Chem. Commun. 2021, 57, 6111. 10.1039/d1cc02112e 34113948

[29] X.‐Y. Yuan , J.‐F. Zheng , X. Ma , X. Huang , Q. Lv , K. Sun , X. Chen , L. Qu , B. Yu , Org. Chem. Front. 2025, 12, 849.

[30] L. Capaldo , Z. Wen , T. Noël , Chem. Sci. 2023, 14, 4230. 37123197 10.1039/d3sc00992k PMC10132167

[31] P. Natho , M. Colella , M. Andresini , L. Degennaro , R. Luisi , Org. Lett. 2024, 26, 3032. 38547907 10.1021/acs.orglett.4c00644 PMC11041934

[32] E. Graziano , P. Natho , M. Andresini , F. Mastrolorito , I. Mahdi , E. Mesto , M. Colella , L. Degennaro , O. Nicolotti , R. Luisi , Adv. Synth. Catal. 2024, 366, 3894.

[33] D. Cambie , C. Bottecchia , N. J. Straathof , V. Hessel , T. Noël , Chem. Rev. 2016, 116, 10276. 26935706 10.1021/acs.chemrev.5b00707

[34] L. Capaldo , S. Bonciolini , A. Pulcinella , M. Nuno , T. Noël , Chem. Sci. 2022, 13, 7325. 35799818 10.1039/d2sc01581a PMC9214841

[35] A. Balakrishna , A. António , P. J. M. Sobral , M. Y. Wani , J. A. e Silva , A. J. F. N. Sobral , Catal. Rev. 2019, 61, 84.

[36] S. Alvi , H. Ahmad , R. Ali , Polycyclic Aromat. Compd. 2024, 44, 3558.

[37] L. H. Dapper , K. M. da Rosa , V. T. Mena , R. O. M. A. de Souza , F. L. N. da Silva , T. Anjos , F. Penteado , E. J. Lenardão , RSC Sustainability 2024, 2, 521.

[38] P. J. Nieuwland , R. Segers , K. Koch , J. C. M. van Hest , F. P. J. T. Rutjes , Org. Process Res. Dev. 2011, 15, 783.

[39] P. B. Cranwell , M. O’Brien , D. L. Browne , P. Koos , A. Polyzos , M. Peña‐López , S. V. Ley , Org. Biomol. Chem. 2012, 10, 5774. 22532036 10.1039/c2ob25407g

[40] J. S. Moore , K. F. Jensen , Angew. Chem. Int. Ed. 2014, 53, 470. 10.1002/anie.201306468 24288212

[41] T. M. Masson , S. D. A. Zondag , J. H. A. Schuurmans , T. Noël , React. Chem. Eng. 2024, 9, 2218. 10.1039/d4re00540f PMC11726180 39816783

[42] The calculation was reported on the radical addition of heptanal to cyclohexenone.

[43] E. Cassera , V. Martini , V. Morlacci , S. Abrami , N. Della Ca' , D. Ravelli , M. Fagnoni , L. Capaldo , JACS Au 2025, 10.1021/jacsau.5c00530. PMC12308375 40747018

[44] D. Ravelli , M. Zema , M. Mella , M. Fagnoni , A. Albini , Org. Biomol. Chem. 2010, 8, 4158. 20661511 10.1039/c0ob00066c

[45] T. Ozturk , E. Ertas , O. Mert , Chem. Rev. 2007, 107, 5210. 17867708 10.1021/cr040650b

[46] S. Massa , F. Corelli , M. Artico , A. Mai , R. Ragno , A. De Montis , A. G. Loi , S. Corrias , M. E. Marongiu , P. La Colla , J. Med. Chem. 1995, 38, 803. 7877145 10.1021/jm00005a007

[47] M. P. DeMartino , K. Chen , P. S. Baran , J. Am. Chem. Soc. 2008, 130, 11546. 18680297 10.1021/ja804159y

[48] P. M. McCurry Jr. , R. K. Singh , J. Org. Chem. 1974, 39, 2316.

[49] Prices of symmetrical diketones are based on BLDPharm, Hexane‐2,5‐dione [110‐13‐4] https://www.bldpharm.com/products/110‐13‐4.html (accessed May 6, 2025). Octane‐3,6‐dione [2955‐65‐9] https://www.bldpharm.com/products/2955‐65‐9.html (accessed May 6, 2025). 1,4‐Diphenylbutane‐1,4‐dione [495‐71‐6] https://www.bldpharm.com/products/495‐71‐6.html (accessed May 6, 2025). Prices of unsymmetrical diketones are based on Enamine, 1‐cyclopropylpentane‐1,4‐dione [167692‐57‐1] https://new.enaminestore.com/catalog/BBV‐33911276 (accessed May 6, 2025). 1‐(oxan‐3‐yl)pentane‐1,4‐dione [77342‐68‐8] https://new.enaminestore.com/catalog/BBV‐37842012 (accessed May 6, 2025). 1‐(4‐chlorophenyl)pentane‐1,4‐dione [53842‐12‐9] https://new.enaminestore.com/catalog/BBV‐27034564 (accessed May 6, 2025). 1‐cyclohexylpentane‐1,4‐dione [61771‐79‐7] https://new.enaminestore.com/catalog/BBV‐33910872 (accessed May 6, 2025). Prices converted to euros as of May 6, 2025.

