# Supplementary material for: A Telescoped Strategy for the Preparation of Five‐Membered Hetero‐ and Carbocycles via Hydrogen Atom Transfer Photocatalysis in Flow
Source: ChemSusChem. 2025 Jul 12;18(16):e202501012. doi: 10.1002/cssc.202501012 (PMC12330334; doi:10.1002/cssc.202501012)
Supplement: Supplementary file 1 — Supplementary Material [file CSSC-18-e202501012-s001.pdf]

Supplementary Information

A Telescoped Strategy for the Preparation of Five-Membered Hetero- and Carbocycles via Hydrogen Atom Transfer Photocatalysis in Flow

Filippo Sacchelli,[a,‡] Elena Quadri,[b,‡] Luna Raineri,[a,‡] Alexandra Jorea,[b] Marzia Pessina,[a] Anna Lo Presti,[b] Nicola Della Ca',[a,c] Davide Ravelli,*[,b] Luca Capaldo*[,a,c]

[a] SynCat Lab, Department of Chemistry, Life Sciences and Environmental Sustainability, University of Parma, Parco Area delle Scienze 17/A, 43124 Parma, Italy. E-mail: luca.capaldo@unipr.it

[b] PhotoGreen Lab, Department of Chemistry, University of Pavia, viale Taramelli 12, 27100 Pavia, Italy. E-mail: davide.ravelli@unipv.it

[c] CIRCC (Interuniversity Consortium Chemical Reactivity and Catalysis), via Celso Ulpiani 27, 70126 Bari (Italy).

[‡] These authors contributed equally to this work.

luca.capaldo@unipr.it; davide.ravelli@unipv.it

Contents

1. General information S3
2. Chart of starting materials S4
3. Synthesis of starting materials S5

Synthesis of **S1e** S5

Synthesis of **S1f** S5

Synthesis of **S2c** S6

Synthesis of **S2e** S6

Synthesis of **S2g** S7

Synthesis of **S2j** S7

4. Reactor design S9
5. Optimization of reaction conditions S11

5.1. Optimization of the radical hydroacylation step (OP1 & OP2). S11

5.2. Optimization of the Paal-Knorr step for pyrroles synthesis (OP3 & OP4). S12

5.3. Optimization of the telescoped pyrrole synthesis (OP5 & OP6). S14

5.4. Optimization of the Paal-Knorr step for thiophenes synthesis (OP7). S16

5.5. Optimization of the telescoped thiophene synthesis (OP8). S16

5.6. Optimization of the Hunsdiecker condensation step for cyclopentenones synthesis (OP9). S17

6. General procedures for preparative experiments S19

6.1. Telescoped synthesis of pyrroles (GP1) S19

6.2. Telescoped synthesis of thiophenes (GP2) S20

6.3. Telescoped synthesis of cyclopentenones (GP3) S20

6.4. Scale-up for the synthesis of compound **1** in continuous-flow S21

6.5. Scale-up for the synthesis of compound **36** in continuous-flow S22

7. Characterization data for products S23
8. Additional comments on the Hunsdiecker condensation (HC) S34
9. References S35
10. Copy of NMR spectra S36

10.1 Starting Materials S37

10.2 Pyrroles S43

10.3 Thiophenes S70

10.4 Cyclopentenones S77

1. General information

Reagents and consumables. All reagents and solvents were bought from Sigma Aldrich, TCI, Fluorochem, and BLDPharm and used as received unless otherwise specified. The solvents employed in this work were purchased from Carlo Erba or Sigma Aldrich and used as received. Disposable syringes were purchased from B. Braun. TLC analysis was performed using Silica on aluminum foils TLC plates (F254, Merck) with visualization under ultraviolet light (254 nm and 365 nm) or appropriate TLC staining (potassium permanganate). Flash column chromatography was performed on silica gel 60 (70–230 mesh) manually. Syringe pumps were purchased from Chemyx Inc. (model F-100X Touch). The peristaltic pump was purchased form Darwin Microfluidics (model LabV1 Intelligent Low Flow Rate Peristaltic Pump). Capillary tubing (FEP, PTFE inner diameter 0.8 mm) and microfluidic fittings were purchased from IDEX Health & Science, Sepachrom and Darwin Microfluidics. Omnifit labware column (internal section area: 0.7854 cm^2) with 1 fixed and 1 adjustable end-piece were used for packed-bed reactors. The photocatalyst was synthesized according to a published procedure.[1]

NMR spectroscopy. ^1H and ^{13}C NMR spectra were recorded at 300 K on a Bruker AVANCE 400 Hz, JEOL 600 MHz ECZ600R or on a 300 MHz Bruker spectrometer in $CDCl_3$ or CD_3CN, using the solvent residual signals as internal reference (7.26 and 77.2 ppm for $CDCl_3$, 1.94 and 1.3 ppm for, respectively for ^1H and ^{13}C). ^{19}F spectra were registered on JEOL 600 MHz ECZ600R at 565 Hz. The terms *m*, *s*, *d*, *t*, *q, quint, dd and td* refer to multiplet, singlet, doublet, triplet, quadruplet, quintet, doublet of doublet and triplet of doublet, respectively. Chemical shifts (δ) and coupling constants (*J*) are given in ppm and in Hz, respectively. NMR data were processed using the MestReNova 14.1.0 software package. Known products were characterized by comparing to the corresponding ^1H NMR, ^{13}C NMR with those available in the literature.

GC-FID. GC-FID analyses were performed on an Agilent 7820A chromatograph. The injection was performed at 250 °C in split mode. The initial oven temperature of 80 °C was maintained for 2 min, increased by 10 °C/min to 250 °C and held for 5 min. An Agilent HP5 30 m × 0.32 mm × 0.25 μm film thickness capillary column was used with nitrogen as the carrier gas at a constant flow rate of 6.0 $mL \cdot min^{-1}$.

Melting point. Melting points were measured with an Electrothermal apparatus and are uncorrected.

HRMS. High resolution mass spectrometry data were acquired using a X500B QTOF System (SCIEX, Framingham, MA 01701 USA) available at the CGS of the University of Pavia, equipped with the Twin Sprayer ESI probe and coupled to an ExionLC™ system (SCIEX). The SCIEX OS software 2.1.6 was used as operating platform. For MS detection the following parameters were applied: curtain gas: 30 psi, ion source gas 1: 45 psi, ion source gas 2: 55 psi, temperature: 450 °C, polarity positive, ion spray voltage: -4500 V, TOF mass range: 50-1600 Da, declustering potential: -60 V and collision energy: -10 V.

2. Chart of starting materials

Starting materials

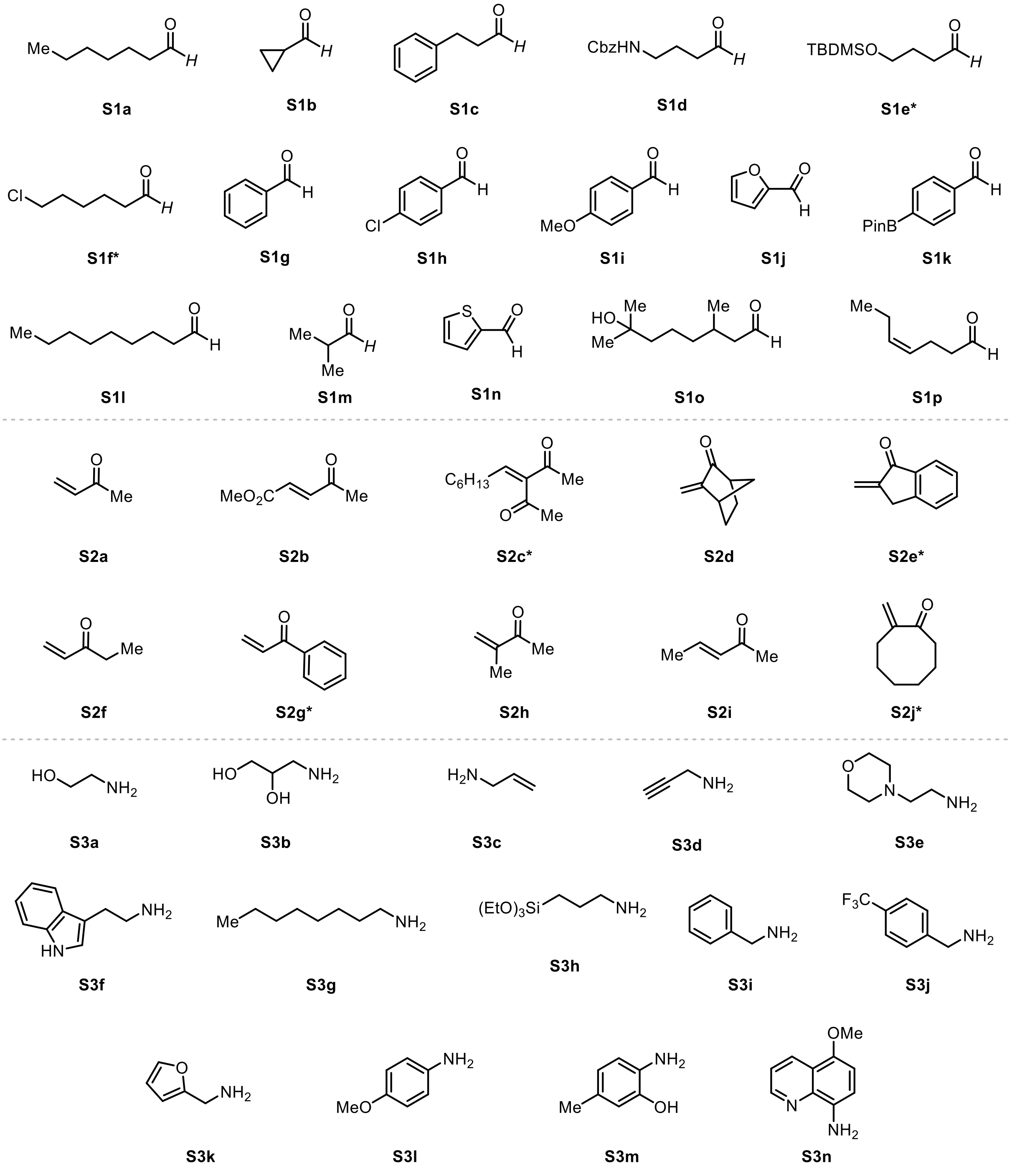

* synthesized according to procedures reported in the literature

3. Synthesis of starting materials

*Synthesis of **S1e***

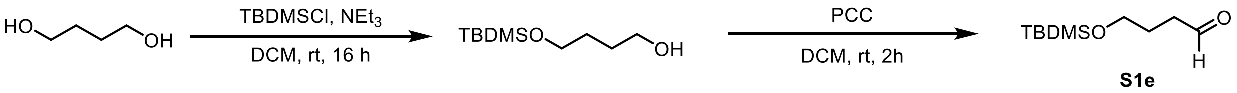

Compound **S1e** was prepared adapting a procedure from the literature.[2] 1,4-butanediol (4 mmol, 1.0 eq.) was dissolved in CH_2Cl_2 (12 mL); triethylamine (4 mmol, 1.0 eq.) and a solution of *tert*-butyldimethylsilyl chloride (4 mmol, 1.0 eq.) in CH_2Cl_2 (2 mL) were added. After stirring for 16 h at room temperature, the reaction mixture was successively extracted with 10% aqueous $NaHCO_3$, water, and brine. The organic layer was dried over Na_2SO_4, filtered, and concentrated under reduced pressure. The crude product was purified by flash column chromatography (Hexane:EtOAc 8:1) to afford 4-((*tert*-butyldimethylsilyl)oxy)butan-1-ol (384 mg, 40%) as a colourless liquid.

^{1}H NMR (600 MHz, $CDCl_3$) δ 3.59 (t, *J* = 6 Hz, 2H), 3.55 (t, *J* = 6 Hz, 2H), 3.13 (s, 1H), 1.60 – 1.51 (m, 4H), 0.83 (s, 9H), -0.01 (s, 6H).

^{13}C NMR (151 MHz, $CDCl_3$) δ 63.3, 62.5, 30.0, 29.8, 25.9, 18.3, -5.4.

Spectroscopic data are in accordance with the literature.[3]

A mixture of PCC (480 mg, 2.24 mmol, 1.4 eq.) and SiO_2 (400 mg) was suspended in CH_2Cl_2 (5 mL) under an N_2 atmosphere at 0 °C. 4-((*tert*-butyldimethylsilyl)oxy)butan-1-ol (1.6 mmol, 1.0 eq.) was added and stirred at room temperature for 2 hours until the complete conversion of the starting material. The reaction mixture was filtered and washed with CH_2Cl_2. The combined organic layer was dried over anhydrous Na_2SO_4. After evaporation of the solvent under reduced pressure, the residue was purified by flash column chromatography (Hexane:EtOAc 10:1) to afford aldehyde **S1e** (188 mg, 58% yield) as a colourless oil.

^{1}H NMR (600 MHz, $CDCl_3$) δ 9.75 (s, 1H), 3.62 (t, *J* = 6 Hz, 2H), 2.47 (t, *J* = 7 Hz, 2H), 1.83 (quint, *J* = 7 Hz, 2H), 0.85 (s, 9H), 0.01 (s, 6H).

^{13}C NMR (151 MHz, $CDCl_3$) δ 202.6, 62.2, 40.9, 26.0, 25.6, 18.3, -5.3.

Spectroscopic data are in accordance with the literature.[3]

*Synthesis of **S1f***

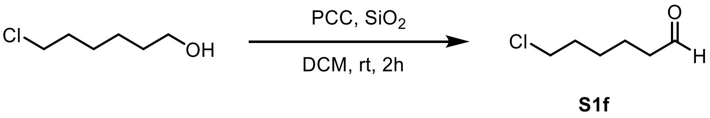

Compound **S1f** was synthesized following a literature procedure.[4] A suspension of PCC (603 mg, 2.8 mmol) and SiO_2 (480 mg) in CH_2Cl_2 (5 mL) was prepared under an N_2 atmosphere at 0 °C. 6-chlorohexan-1-ol (2 mmol, 1.0 eq.) was added and stirred at room temperature for 2 hours until the complete conversion of the starting material. The reaction mixture was filtered and washed with CH_2Cl_2. The combined organic layer was dried over anhydrous Na_2SO_4. After evaporation of the solvent under reduced pressure, the residue was purified via flash column chromatography (Hexane:EtOAc 10:1) to afford aldehyde **S1f** (269 mg, 99% yield) as a colourless oil.

^{1}H NMR (600 MHz, $CDCl_3$) δ 9.70 (t, *J* = 2 Hz, 1H), 3.48 (t, *J* = 7 Hz, 2H), 2.40 (td, *J* = 7, 2 Hz, 2H), 1.78 – 1.67 (m, 2H), 1.65 – 1.54 (m, 2H), 1.47 – 1.35 (m, 2H)

^{13}C NMR (151 MHz, $CDCl_3$) δ 202.2, 44.7, 43.6, 32.3, 26.3, 21.3.

Spectroscopic data are in accordance with the literature.[5]

Synthesis of ***S2c***

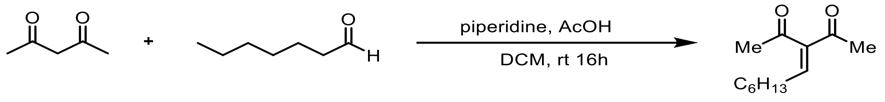

S2c

Compound **S2c** was synthesized adapting a literature procedure.[6] A stirred solution of the **S1a** (2.00 mmol) and 2,4-pentanedione (2.20 mmol, 1.1 eq.) in anhydrous CH_2Cl_2 (3 mL) was treated with piperidine (0.04 mmol, 2 mol%) and acetic acid (0.04 mmol, 2 mol%) at 0 °C. The reaction mixture was stirred at room temperature for 16 h, diluted with CH_2Cl_2, washed with brine, and dried (Na_2SO_4). After removing the solvent, the crude residue was purified by column chromatography (Hexane:EtOAc 11:1) to afford the compound **S2c** (287 mg, 73% yield).

^{1}H NMR (400 MHz, $CDCl_3$) δ 6.62 (t, *J* = 8 Hz, 1H), 2.24 (s, 3H), 2.23 (s, 3H), 2.20 – 2.12 (m, 2H), 1.41 (quint, *J* = 7 Hz, 2H), 1.29 – 1.17 (m, 6H), 0.81 (t, *J* = 7 Hz, 3H).

^{13}C NMR (101 MHz, $CDCl_3$) δ 203.5, 197.2, 147.0, 145.1, 31.7, 31.5, 29.6, 29.0, 28.6, 26.0, 22.5, 14.0.

Synthesis of ***S2e***

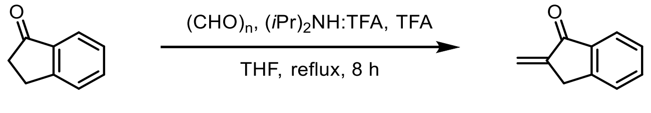

S2e

Compound **S2e** was synthesized following a literature procedure.[7] To a mixture of indanone (4.0 mmol) and paraformaldehyde (8.0 mmol, 2 eq.) in dry THF (4.0 mL) is added diisopropylammonium 2,2,2-trifluoroacetate (4.0 mmol, 1 eq.) and trifluoroacetic acid (0.4 mmol, 10 mol%). The reaction mixture

was stirred at reflux for 2 h. The mixture became clear with time, then the reaction mixture was cooled down to room temperature and a second addition of paraformaldehyde (8.0 mmol, 2 eq.) was performed. Next, the reaction mixture was stirred at reflux for an additional 6 h. The reaction mixture was cooled down, the solvent was removed under reduced pressure, and the residue was dissolved in Et_2O and washed sequentially with 1N HCl, 1N NaOH, and brine. The solution mixture was dried over Na_2SO_4 and concentrated under vacuum. The crude product was purified by silica gel column chromatography (Hexane:EtOAc 20:1) to afford the compound **S2c** (374 mg, 65% yield).

^{1}H NMR (400 MHz, $CDCl_3$) δ 7.73 (dd, J = 7, 3 Hz, 1H), 7.55 – 7.43 (m, 1H), 7.42 – 7.32 (m, 1H), 7.31 – 7.24 (m, 1H), 6.29 – 6.21 (m, 1H), 5.55 – 5.49 (m, 1H), 3.61 (s, 2H).

^{13}C NMR (101 MHz, $CDCl_3$) δ 193.1, 149.7, 143.2, 138.0, 134.7, 127.4, 126.2, 124.3, 119.0, 31.6. Spectroscopic data are in accordance with the literature.[7-8]

*Synthesis of **S2g***

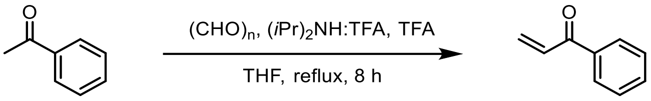

S2g

Compound **S2g** was synthesized following a literature procedure.[7]

^{1}H NMR (400 MHz, CD_3CN) δ 7.96 (m, 2H), 7.66 – 7.47 (m, 3H), 7.25 (m, 1H), 6.36 (dd, *J* = 17.1, 1.8 Hz, 1H), 5.93 (m, 1H).

^{13}C NMR (101 MHz, CD_3CN) δ 191.51, 138.29, 134.09, 133.52, 130.48, 130.44, 129.74, 129.53, 118.29.

Spectroscopic data are in accordance with the literature.[7]

*Synthesis of **S2j***

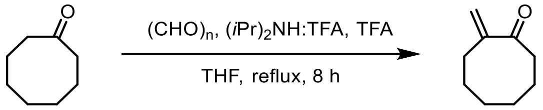

S2j

Compound **S2j** was synthesized adapting a literature procedure. [7] To a mixture of cyclooctanone (10.0 mmol) and paraformaldehyde (20.0 mmol, 2 eq.) in dry THF (10.0 mL) is added the diisopropylammonium 2,2,2-trifluoroacetate (10.0 mmol, 1 eq.) and trifluoroacetic acid (1.0 mmol, 10 mol%). The reaction mixture was stirred at reflux for 2 h. The mixture became clear with time, then the

reaction mixture was cooled down to room temperature and a second addition of paraformaldehyde (20.0 mmol, 2 eq.) was performed. Next, the reaction mixture was stirred at reflux for an additional 6 h. The reaction mixture was cooled down, the solvent was removed under reduced pressure, and the residue was dissolved in Et_2O and washed sequentially with 1N HCl, 1N NaOH, and brine. The solution mixture was dried over Na_2SO_4 and concentrated under vacuum. The crude product was purified by silica gel column chromatography (Cyclohexane:EtOAc 8:2) to afford the compound **S2j** (214 mg, 16% yield).

^{1}H NMR (400 MHz, CD_3CN) δ 5.67 (d, *J* = 2.3 Hz, 1H), 5.15 (dt, *J* = 2.1, 1.0 Hz, 1H), 2.58 – 2.47 (m, 4H), 1.70 – 1.62 (m, 2H), 1.56 – 1.46 (m, 4H), 1.38 – 1.30 (m, 2H).

^{13}C NMR (101 MHz, CD_3CN) δ 207.10, 150.34, 121.69, 40.07, 31.93, 31.57, 29.68, 26.99, 26.28.

Spectroscopic data are in accordance with the literature.[9]

4. Reactor design

Experimental setup for pyrrole synthesis. For the synthesis of pyrroles, we adopted a telescoped reactor composed of a first photoreactor and a thermal tubular reactor. The photochemical reaction was conducted in a 3D-printed reactor (PLA) equipped with a commercially available Kessil LED PR160L 390 nm, tubular microreactor (FEP, ID: 0.8 mm). The full characterization of this reactor is available in the literature.[10] The subsequent thermal transformation (condensation) was conducted in a tubular FEP reactor (ID: 0.8 mm), maintained at the desired temperature using a thermostated water bath.

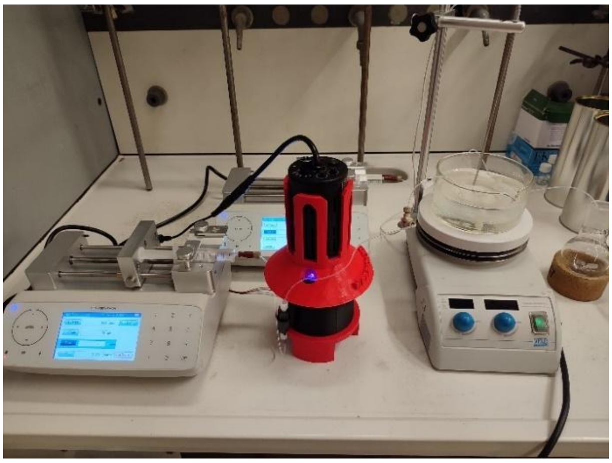
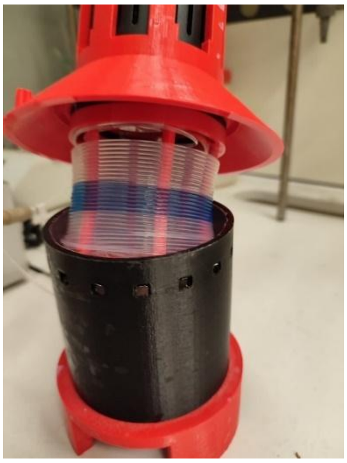

Figure S1: Set-up used for the telescoped synthesis of pyrroles (left) and detail of the photoreactor (right).

Experimental setup for thiophene synthesis. For the synthesis of thiophenes, we adopted a telescoped reactor composed of a first photoreactor and a packed-bed reactor. The photochemical reaction was conducted as described for the synthesis of pyrroles. The condensation step was conducted in a packed-bed reactor filled with the thionation reagent (see OPs).

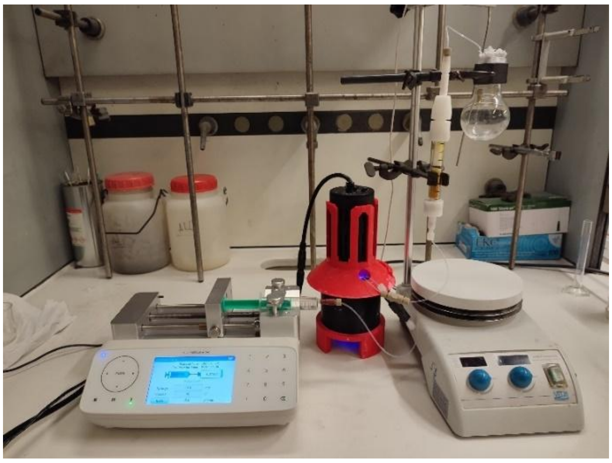
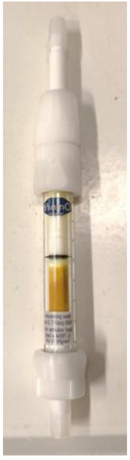

Figure S2: Set-up used for the telescoped synthesis of thiophenes (left) and detail of the packed-bed reactor (right).

Experimental setup for cyclopentenones synthesis. For the synthesis of cyclopentenones, we used a home-made photochemical reactor composed of PTFE tubing (internal diameter: 0.8 mm; total volume: 2.5 mL) installed on a flat 3D-printed support (PLA) and equipped with a Kessil LED PR160L 370 nm (the light source-reactor distance: 5 cm during operation).

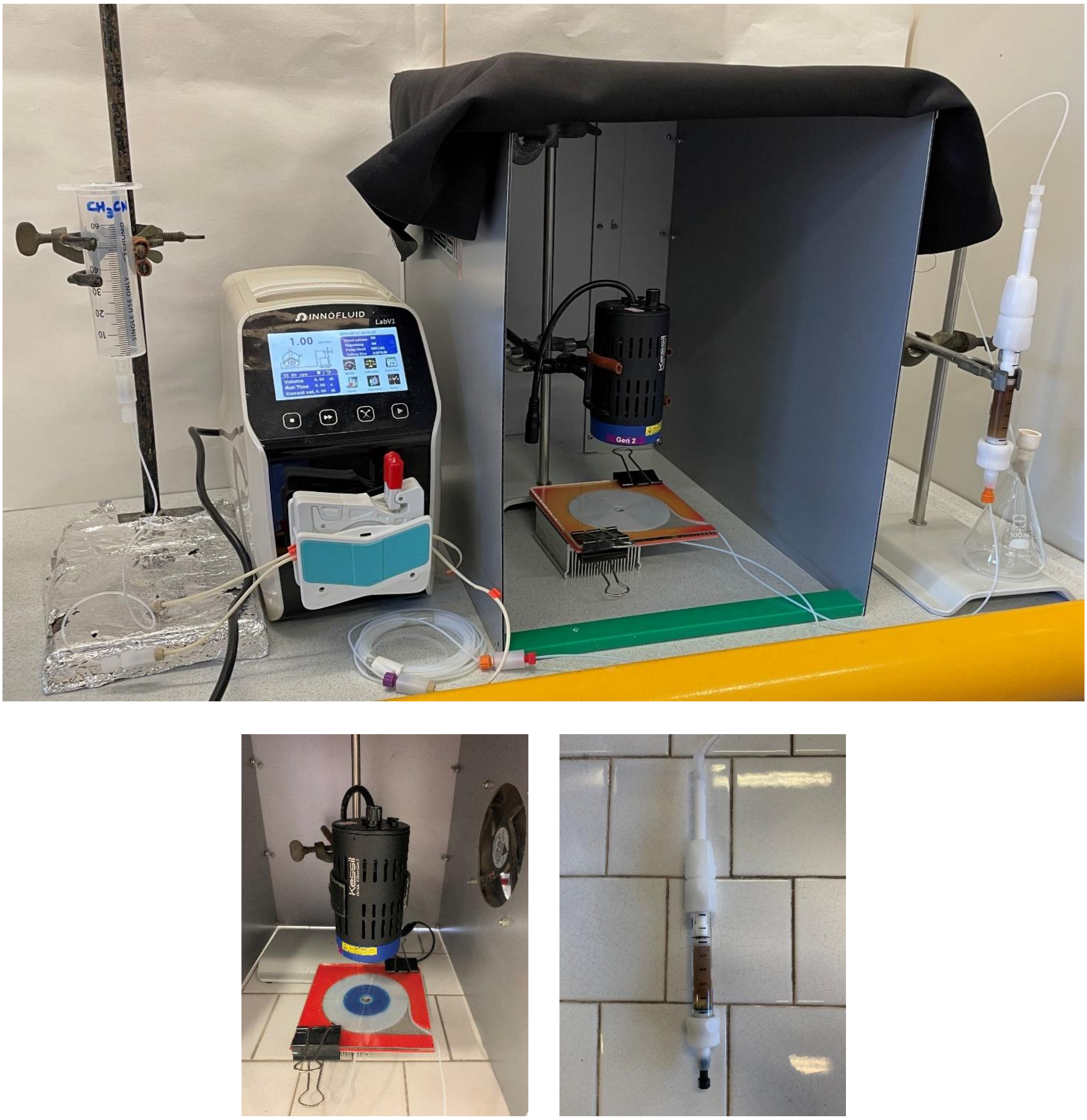

Figure S3: Set-up used for the telescoped synthesis of cyclopentenones (upper part); detail of the photoreactor (left) and of the packed-bed reactor (right).

5. Optimization of reaction conditions

Optimization Procedures (OPs) are reported below: first, we optimized separated steps and then we performed a last round of fine tuning for the telescoped processes.

5.1. Optimization of the radical hydroacylation step (OP1 & OP2).

OP1 - Optimization of the hydroacylation step for aldehydes **S1a-S1f**: The optimization of the reaction conditions was carried out by studying the radical addition of heptanal (**S1a**) onto freshly distilled methyl vinyl ketone (**S2a**) to give undecane-2,5-dione on a 0.1 mmol scale (see Table S1). A stock solution of **S1a**, **S2a** and TBADT in CH_3CN was prepared in a volumetric flask, 2 mL of this solution were transferred to a vial and the mixture was N_2-bubbled (2 min). The vial was sealed. Under positive pressure of N_2 (via balloon), 1 mL of the degassed solution was withdrawn with a plastic disposable syringe and mounted on a syringe pump. The solution was pushed into a 2.5 mL FEP photoreactor at the desired flow rate and irradiated with a 40 W Kessil lamp (λ = 390, 370 or 456 nm) by utilizing the reactor shown in Figure S1. The crude was collected at the end of the photoreactor in a round-bottomed flask under air and the solvent was removed under reduced pressure. Next, the crude was suspended in 600 μL of $CDCl_3$, CH_2Br_2 (0.1 mmol, 7 μL) was added and the mixture was sonicated and filtered through a short cotton plug in a Pasteur pipette. The filtrate was analysed via ^{1}H-NMR.

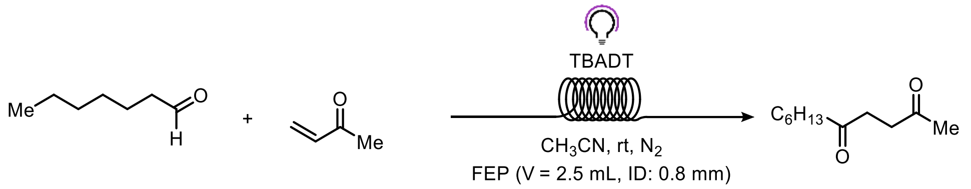

Table S1. Optimization of the hydroacylation step for aliphatic aldehydes.

Entry	[S1a] (*n* eq.)	[S2a] (*n* eq.)	TBADT (mol%)	fr (mL/min)	λ (nm), intensity	τ_R (min)	Yield [a]
1	0.11 M (1.1 eq.)	0.1 M (1 eq.)	4	2.5	390, 100%	1	47%
2	0.11 M (1.1 eq.)	0.1 M (1 eq.)	4	1.5	390, 100%	1.7	56%
3	0.11 M (1.1 eq.)	0.1 M (1 eq.)	4	1.0	390, 100%	2.5	66%
4	0.11 M (1.1 eq.)	0.1 M (1 eq.)	4	0.5	390, 100%	5	75%
5	0.11 M (1.1 eq.)	0.1 M (1 eq.)	4	0.25	390, 100%	10	73%
6	0.11 M (1.1 eq.)	0.1 M (1 eq.)	4	0.5	390, 75%	5	70%
7	0.11 M (1.1 eq.)	0.1 M (1 eq.)	4	0.5	390, 50%	5	62%
8	0.11 M (1.1 eq.)	0.1 M (1 eq.)	4	0.5	390, 25%	5	56%
9	0.11 M (1.1 eq.)	0.1 M (1 eq.)	4	0.5	None	5	n.d.
10	0.11 M (1.1 eq.)	0.1 M (1 eq.)	4	0.5	456, 100%	5	n.d.
11	0.11 M (1.1 eq.)	0.1 M (1 eq.)	4	0.5	370, 100%	5	76%
12	0.11 M (1.1 eq.)	0.1 M (1 eq.)	4	0.5	370, 50%	5	65%
13	0.11 M (1.1 eq.)	0.1 M (1 eq.)	4	1.5	370, 100%	1.7	73%
14	0.11 M (1.1 eq.)	0.1 M (1 eq.)	4	2.5	370, 100%	1	62%

**15**	**0.1 M (1 eq.)**	**0.1 M (1 eq.)**	**4**	**0.5**	**390, 100%**	**5**	**75%**
16	0.1 M (1 eq.)	0.11 M (1.1 eq.)	4	0.5	390, 100%	5	72%
17	0.1 M (1 eq.)	0.1 M (1 eq.)	2	0.5	390, 100%	5	65%
18	0.1 M (1 eq.)	0.1 M (1 eq.)	1	0.5	390, 100%	5	34%

[a] Yield calculated via ^1H NMR using CH_2Br_2 as internal standard.

<u>OP2 – Optimization of the hydroacylation step for aldehydes **S1g-S1k**:</u> The optimization of the reaction conditions was carried out by studying the radical addition of benzaldehyde (**S1g**) onto freshly distilled methyl vinyl ketone (**S2a**) to give 1-phenylpentane-1,4-dione on a 0.1 mmol scale (see Table S2). The reaction mixture was prepared, used and processed as described above for OP1.

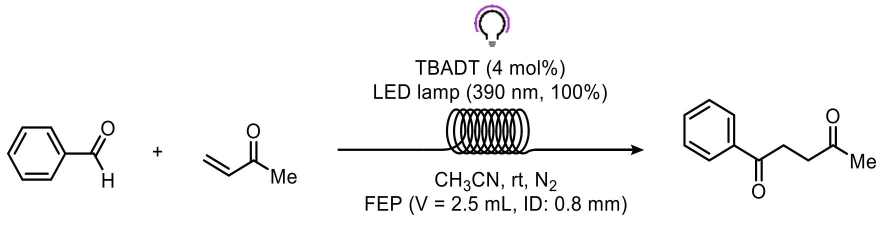

Table S2. Re-optimization of the hydroacylation step for aromatic aldehydes.

Entry	[S1g] (*n* eq.)	[S2a] (*n* eq.)	solvent	fr (mL/min)	τ_R (min)	Yield [a]
1	0.1 M (1 eq.)	0.1 M (1 eq.)	CH_3CN	0.5	5	45%
2	0.1 M (1.1 eq.)	0.1 M (1 eq.)	CH_3CN	0.5	5	50%
3	0.1 M (1.1 eq.)	0.1 M (1 eq.)	CH_3CN	0.25	10	56%
4	0.1 M (1.1 eq.)	0.1 M (1 eq.)	CH_3CN:HCl 0.1M (9:1)	0.25	10	32%
5	0.12 M (1.2 eq.)	0.1 M (1 eq.)	CH_3CN	0.25	10	43%
**6**	**0.1 M (1 eq.)**	**0.12 M (1.2 eq.)**	**CH_3CN**	**0.25**	**10**	**63%**
7	0.1 M (1 eq.)	0.12 M (1.2 eq.)	CH_3CN	0.15	16.7	60%

[a] Yield calculated via ^1H NMR using CH_2Br_2 as internal standard.

5.2. Optimization of the Paal-Knorr step for pyrroles synthesis (OP3 & OP4).

<u>OP3 - Optimization of the Paal-Knorr reaction for 1,4-diketones deriving from aldehydes **S1a-S1f**:</u> The optimization of the process was carried out by studying the reaction of undecane-2,5-dione with ethanolamine (**S3a**) to give 2-(2-hexyl-5-methyl-1H-pyrrol-1-yl)ethan-1-ol (**1**) on a 0.1 mmol scale (Table S3). Thus, two stock solutions in CH_3CN were prepared: the first one contained 1,4-diketone (0.1 M), while the second one contained **S3a** and PTSA (*p*-toluenesulfonic acid) as indicated below. The solutions were withdrawn via plastic disposable syringes and mounted on two different syringe pumps. The undecane-2,5-dione solution (<u>*Feed A*</u>) was pumped at 0.5 mL min^{-1} and mixed with the amine solution (<u>*Feed B*</u>) pumped at the desired flow rate via a PEEK T-mixer. The outflow of the T-mixer was pushed into a thermostated FEP reactor. The crude was collected at the end of the tubular reactor a round-bottomed flask containing saturated NH_4Cl solution as the quenching agent under air. After extraction with DCM, the solvent was removed under reduced pressure. Next, the crude was suspended

in 600 μL of $CDCl_3$, CH_2Br_2 (0.1 mmol, 7 μL) was added and the mixture was sonicated. The mixture was analysed via ^{1}H-NMR.

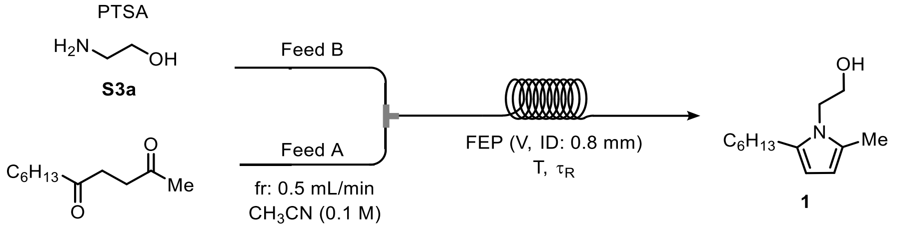

Table S3. Optimization of the Paal-Knorr step for aliphatic diketones.

Entry	[S3a] (*n* eq.)	[PTSA], mol%	fr_{S3a} (mL/min)	V (mL)	τ_R (min)	T (°C)	Yield[a]
1	1 M (1.1 eq.)	-	0.055	5	9	20	21%
2	1 M (1.1 eq.)	-	0.055	5	9	50	32%
3	1 M (1.5 eq.)	-	0.075	5	8.7	50	53%
4	1 M (2 eq.)	-	0.100	5	8.3	50	49%
5	1 M (2 eq.)	-	0.100	10	16.6	50	59%
6	1M (4 eq.)	-	0.200	10	14.3	50	73%
7	1M (4 eq.)	-	0.200	10	14.3	60	77%
8	1 M (4 eq.)	-	0.200	10	14.3	70	44%
9	1 M (4 eq.)	-	0.200	15	21.4	60	58%
10	1.5 M (4 eq.)	-	0.133	10	15.8	60	52%
11	2 M (4 eq.)	-	0.025	10	19	60	24%
12	1 M (4 eq.)	0.05 M (20 mol%)	0.200	10	14.3	60	84%
13	1 M (4 eq.)	0.075 M (30 mol%)	0.200	10	14.3	60	75%
14	1 M (4 eq.)	0.1 M (40 mol%)	0.200	10	14.3	60	86%
**15**	**1 M (4 eq.)**	**0.1 M (40 mol%)**	**0.200**	**15**	**21.4**	**60**	**99%**

[a] Yield calculated via ^{1}H NMR using CH_2Br_2 as internal standard.

<u>OP4 – Optimization of the Paal-Knorr reaction for 1,4-diketones deriving from aldehydes **S1g-S1k**</u>: The optimization of the process was carried out by studying the reaction of 1-phenylpentane-1,4-dione with ethanolamine (**S3a**) to give 2-(2-methyl-5-phenyl-1H-pyrrol-1-yl)ethan-1-ol (**20**) on a 0.1 mmol scale (see Table S4). Thus, two stock solutions in CH_3CN were prepared: the first one contained 1,4-diketone (0.1 M), while the second one contained **S3a** and PTSA as indicated below.

The 1-phenylpentane-1,4-dione solution (*<u>Feed A</u>*) was pumped at 0.25 mL min^{-1} and mixed with the amine solution (*<u>Feed B</u>*) pumped at the desired flow rate via a PEEK T-mixer. The outflow of the T-mixer was pushed into a thermostated FEP reactor. The crude was collected at the end of the tubular reactor in a round-bottomed flask containing saturated NH_4Cl solution as the quenching agent under air. The crude was processed as described for OP3.

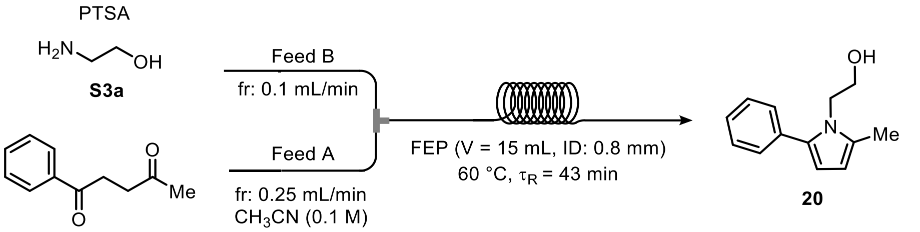

Table S4. Optimization of the Paal-Knorr step for other diketones.

Entry	[S3a] (*n* eq.)	[PTSA], mol%	Yield[a]
1	1 M (4 eq.)	-	4%
2	1 M (4 eq.)	0.05 M (20 mol%)	48%
3	1 M (4 eq.)	0.1 M (40 mol%)	60%
4	1.25 M (5 eq.)	0.1 M (40 mol%)	72%
5	1.5 M (6 eq.)	0.1 M (40 mol%)	70%
**6**	**1 M (4 eq.)**	**0.2 M (80 mol%)**	**82%**
7	1.25 M (5 eq.)	0.2 M (80 mol%)	70%
8	1 M (4 eq.)	0.25 M (100 mol%)	77%
9	1.25 M (5 eq.)	0.25 M (100 mol%)	80%

[a] Yield calculated via ^{1}H NMR using CH_2Br_2 as internal standard.

5.3. *Optimization of the telescoped pyrrole synthesis (OP5 & OP6).*

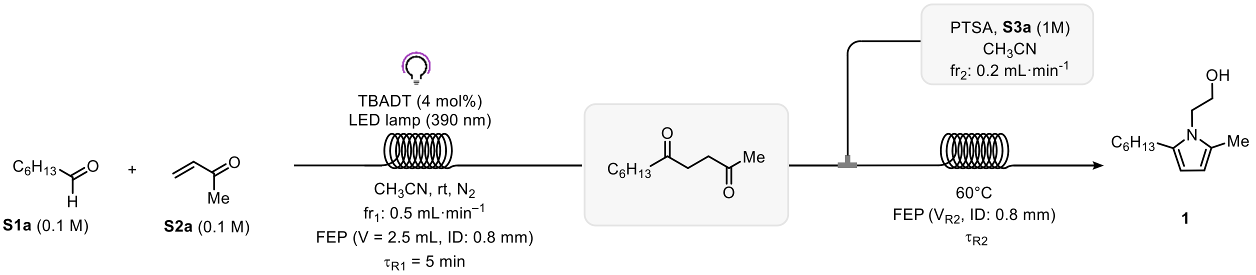

<u>OP5 – Optimization of the telescoped process for pyrroles from aldehydes **S1a-S1f**:</u> When streamlining OP1 and OP3, we realized that some undecane-2,5-dione was left at the end of the process. We attribute this observation to the interference of TBADT with the Paal-Knorr step. Thus, we opted to run a further refinement of the reaction conditions of the telescoped process, as detailed in Table S5.

Table S5. Optimization of the telescoped synthesis of pyrrole 1.

Entry	[S3a] (*n* eq.)	[PTSA], mol%	V_{R2} (mL)	t_{R2} (min)	Yield[a]
1	1 M (4 eq.)	0.1 M (40 mol%)	15	21.4	60%
2	1 M (4 eq.)	0.155 M (60 mol%)	15	21.4	55%
3	1 M (4 eq.)	0.1 M (40 mol%)	20	28.6	62%
**4**	**1 M (4 eq.)**	**0.125 M (50 mol%)**	**20**	**28.6**	**72%**

[a] Yield over two steps calculated via ^{1}H NMR using CH_2Br_2 as internal standard.

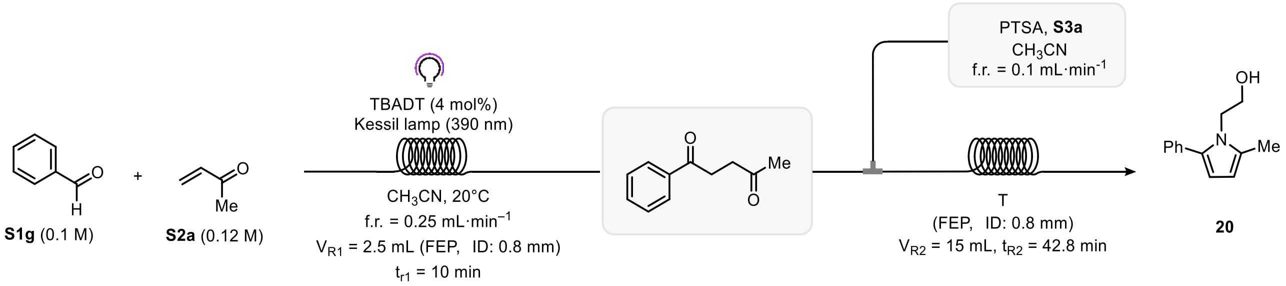

OP6 – Optimization of the telescoped process for pyrroles from aldehydes **S1g-S1k**: When streamlining OP2 and OP4, we realized that some 1-phenylpentane-1,4-dione was left at the end of the process. We attribute this observation to the interference of TBADT with the Paal-Knorr step. We also noted that TBADT precipitation was observed when mixing the two feeds. To prevent clogging in the second reactor, we implemented a mixing loop and an in-flow cotton-plug filter between the two reactors.

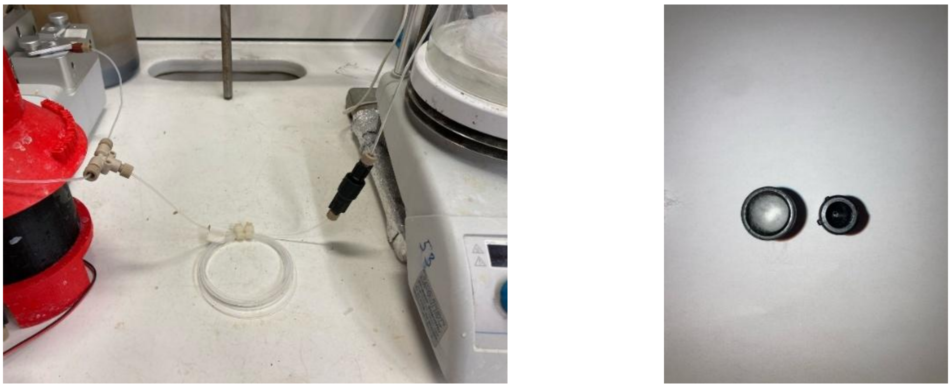

Figure S4: Set-up used for the telescoped synthesis of pyrrole 20 (left) and detail of the in-flow cotton-plug filter (right).

Thus, we opted to run a further refinement of the reaction conditions of the telescoped process, as detailed in Table S6.

Table S6. Optimization of the telescoped synthesis of pyrrole 20.

Entry	[S3a] (*n* eq.)	[PTSA], mol%	T (°C)	Yield[a]
**1**	**1.25 M (5 eq.)**	**0.250 M (100 mol%)**	**60**	**60%**
2	1.5 M (6 eq.)	0.250 M (100 mol%)	60	36%
3	1.5 M (6 eq.)	0.250 M (100 mol%)	70	33%
4	1.75 M (7 eq.)	0.250 M (100 mol%)	60	41%
5	1.25 M (5 eq.)	0.300 M (120 mol%)	60	37%

[a] Yield calculated via ^{1}H NMR using CH_2Br_2 as internal standard.

5.4. Optimization of the Paal-Knorr step for thiophenes synthesis (OP7).

<u>OP7 – Optimization of the Paal-Knorr step for thiophenes</u>: The optimization of the reaction conditions was carried out by studying the reaction between undecane-2,5-dione and Lawesson's reagent to give 2-hexyl-5-methylthiophene (**27**) on a 0.1 mmol scale (see Table S7). Thus, a CH_3CN stock solution of diketone (0.1 M) was prepared in a volumetric flask, 1 mL of such solution was withdrawn with a disposable syringe and mounted on a syringe pump. The packed bed reactor was prepared by filling an Omnifit EZ SolventPlus glass column (7.8 mm internal diameter, 100 mm length and adjustable endpieces) with a mixture of Lawesson's reagent and Na_2SO_4 as desiccant. The solution was flowed through the packed-bed reactor at 0.5 mL·min^{-1} (Figure S2). Afterwards, a syringe containing neat CH_3CN was mounted on the syringe pump and the reactor was flushed with 5 mL of solvent. The crude was collected at the end of the packed-bed reactor in a round-bottomed flask under air, the solvent was removed under reduced pressure. Next, the crude was suspended in 600 μL of $CDCl_3$, CH_2Br_2 (0.1 mmol, 7 μL) was added and the mixture was sonicated. The mixture was analysed via ^{1}H-NMR.

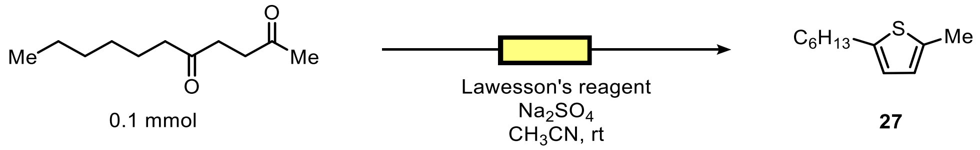

Table S7. Optimization of the Paal-Knorr step for aliphatic diketones.

Entry	Lawesson's reagent	Na_2SO_4	Yield[a]
1	809 mg (20 eq.)	-	67%
2	809 mg (20 eq.)	2.4 g	92%
**3**	**404 mg (10 eq.)**	**2.4 g**	**93%**
4	202 mg (5 eq.)	2.4 g	85%

[a] Yield calculated via ^{1}H NMR using CH_2Br_2 as internal standard.

5.5. Optimization of the telescoped thiophene synthesis (OP8).

<u>OP8 – Optimization of the telescoped process for preparing thiophenes</u>: The optimization of the telescoped platform to prepare thiophenes was carried out by streamlining the best conditions from OP1 and OP7 (Table S8).

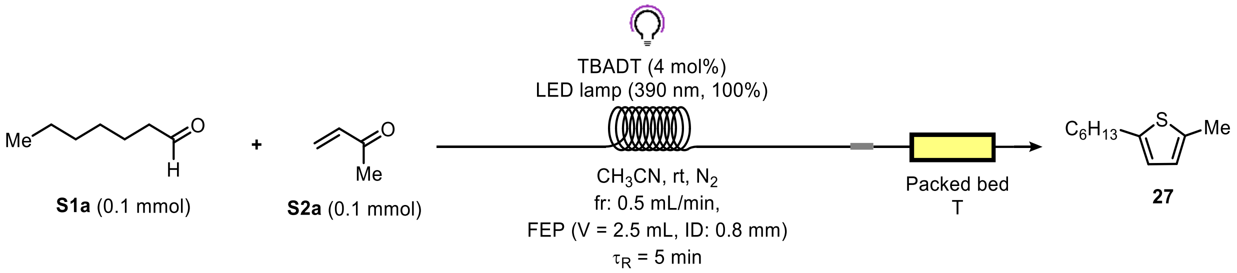

Table S8. Optimization of the telescoped synthesis of thiophene 27.

Entry	Filler for packed-bed reactor	T (°C)	Yield[a]
1	Mix (20 eq. Lawesson's reagent + 2.4 g Na_2SO_4)	20	55%
**2**	**Mix (10 eq. Lawesson's reagent + 2.4 g Na_2SO_4)**	**20**	**63%**
3	SiO_2 + Mix (10 eq. Lawesson's reagent + 2.4 g Na_2SO_4)	20	62%
4	Mix (10 eq. Lawesson's reagent + 2.4 g Na_2SO_4)	50	38%

[a] Yield calculated via ^1H NMR using CH_2Br_2 as internal standard

5.6. Optimization of the Hunsdiecker condensation step for cyclopentenones synthesis (OP9).

OP9 – Optimization of the Hunsdiecker condensation step for cyclopentenones: The optimization of the reaction conditions was carried out by studying the cyclization of undecane-2,5-dione to give 3-methyl-2-pentylcyclopent-2-en-1-one (**34**) on a 0.1 mmol scale (see Table S9). Thus, a solution of diketone (0.1 M) in 5 mL of CH_3CN was prepared in a volumetric flask and charged into a coiled tubing reservoir (PTFE, internal diameter: 0.8 mm). By means of a peristaltic pump, the solution was pushed through the packed-bed reactor (Figure S3) prepared as described in GP3. The crude was collected at the end of the packed-bed reactor in a round-bottomed flask under air and the solvent was removed under reduced pressure. Next, the crude was dissolved in ethyl acetate and the progress of the reaction was monitored by GC-FID. At first, we kept the same flow rate of the photochemical reaction (see OP1), but under these conditions we observed some unreacted 1,4-diketone in the chromatogram. Accordingly, we gradually reduced the flow rate until we obtained the total conversion of the 1,4-diketone into the cyclopentenone product of interest.

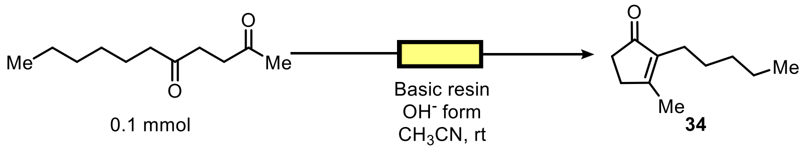

Table S9. Optimization of the Hunsdiecker condensation (HC) for aliphatic diketones.

Entry	fr (mL/min)	V_1 (mL)	τ_R (min)	Yield[b]
1	0.5	3.5	7	45%
2	0.25	3.5	14	62%
**3**	**0.083**	**3.5**	**42**	**70%**
4	0.033	3.5	106	59%

[b] Isolated yield

The described conditions worked well with the diketones deriving from primary aliphatic aldehydes (**S1a**, **S1c**, **S1l**, and **S1o**) and methyl vinyl ketone (**S2a**). When performing the reaction on different

substrates, we observed that the performance of the HC step was affected by the structure of the 1,4-diketone (see Scheme S1). For this reason, it was necessary to re-optimize the reaction conditions on a case by case basis, through proper modification of the following parameters: flow rate and volume of the packed-bed reactor (as for the latter aspect, we doubled the volume of the packed-bed reactor by connecting two Omnifit EZ SolventPlus glass columns in series). The most demanding situations in terms of reaction conditions, requiring dedicated optimization, are represented by the HC of 6-methylheptane-2,5-dione and 1-(4-methoxyphenyl)pentane-1,4-dione to give 3-isopropylcyclopent-2-en-1-one (**38**) and 3-(4-methoxyphenyl)cyclopent-2-en-1-one (**39**), respectively.

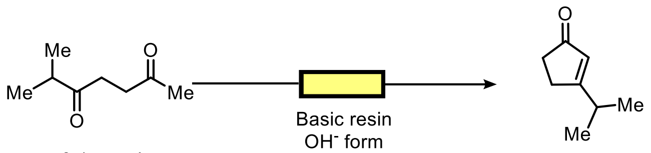

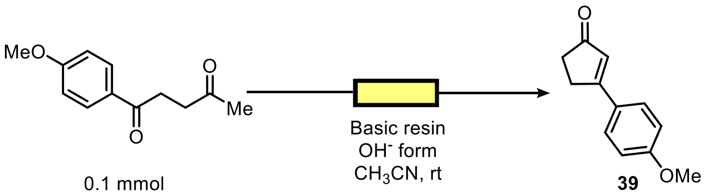

Here the slowest flowrate (1 mL h^{-1}) and the largest volume of the packed-bed reactor (V_2 = 3.5 + 3.5 mL = 7 mL) were needed to obtain a total conversion of the relevant 1,4-diketone. Different combinations of the studied parameters ($f.r._A$ = 5 mL h^{-1}, $f.r._B$ = 2 mL h^{-1} or $f.r._C$ = 1 mL h^{-1} and V_1 = 3.5 mL or V_2 = 7 mL) were applied to the different diketones, according to the results obtained by GC-FID analysis.

The best conditions obtained in OP9 were then employed unchanged for the telescoped synthesis of cyclopentenones, with the optimized flow rates were extended to the photochemical step as well, since the HC reaction appeared to be the slowest step of the overall process. The final conditions are reported in Scheme 3 in the main text.

6. General procedures for preparative experiments

6.1. Telescoped synthesis of pyrroles (GP1)

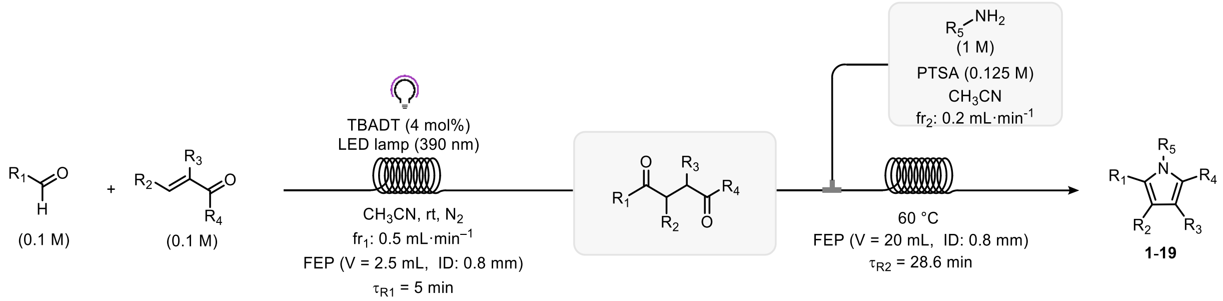

General Procedure 1 (GP1): A stock solution of aldehyde (0.1 M), enone (0.1 M) and TBADT ($4\cdot10^{-3}$ M) in CH_3CN was prepared in a volumetric flask. 2 mL of this solution were transferred to a vial and the mixture was N_2-bubbled (2 min). The vial was sealed. Under positive pressure of N_2 (via balloon), 1 mL of the degassed solution was withdrawn with a plastic disposable syringe and mounted on a syringe pump (*Feed A*). The solution was pushed into a 2.5 mL FEP photoreactor at 0.5 mL$\cdot$min^{-1} and irradiated with a 40 W Kessil lamp (λ = 390, full intensity) by utilizing the reactor shown in Figure S1.
Parallelly, in a vial, a stock solution of the desired amine (1 M) and PTSA (0.125 M) in CH_3CN was prepared. This stock solution was taken up with a plastic disposable syringe and mounted on a second syringe pump (*Feed B*). The outflow of the photoreactor was then mixed with *Feed B* (pumped at 0.2 mL min^{-1}) through a PEEK T-mixer. The outflow of the T-mixer was pushed into 20 mL FEP reactor kept at 60°C. The crude was collected at the end of the tubular reactor a round-bottomed flask containing saturated NH_4Cl solution as the quenching agent under air. After extraction with DCM, the solvent was removed under reduced pressure. Next, the crude was purified via flash chromatography on silica gel to afford pyrroles **1-19, 25** and **26**.

For aldehydes **S1g**-**S1k** we prepared a stock solution of **S2a** (0.12M) and Feed A was pushed into a 2.5 mL FEP photoreactor at 0.25 mL$\cdot$min^{-1}. A stock solution of ethanolamine (1.25 M) and PTSA (0.250 M) in CH_3CN was prepared. The outflow of the photoreactor was then mixed with *Feed B* (pumped at 0.1 mL min^{-1}) through a PEEK T-mixer. The outflow of the T-mixer was pushed into 15 mL FEP reactor kept at 60°C.

6.2. Telescoped synthesis of thiophenes (GP2)

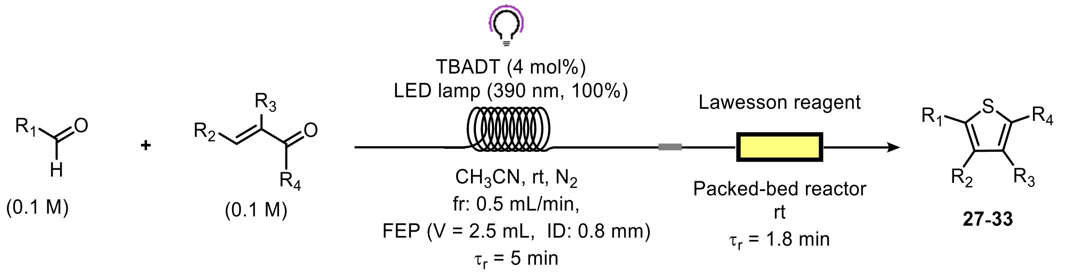

General procedure 2 (GP2): The hydroacylation step was conducted as described in GP1, except for the synthesis of thiophene **32 and 33**, for which the variant described in GP1 was followed.

The packed-bed reactor was prepared by filling an Omnifit EZ SolventPlus glass column (7.8 mm internal diameter, 100 mm length and adjustable endpieces) with a mixture of Lawesson's reagent (404 mg, 10 eq.) and Na_2SO_4 (2.4 g). The outflow of the photoreactor was directly flowed through the packed-bed reactor, which was then flushed with CH_3CN. The crude was collected at the end of the packed bed reactor in a round-bottomed flask under air, the solvent was removed under reduced pressure. Next, the crude was purified by flash chromatography on silica gel to afford the products (**27-33**).

6.3. Telescoped synthesis of cyclopentenones (GP3)

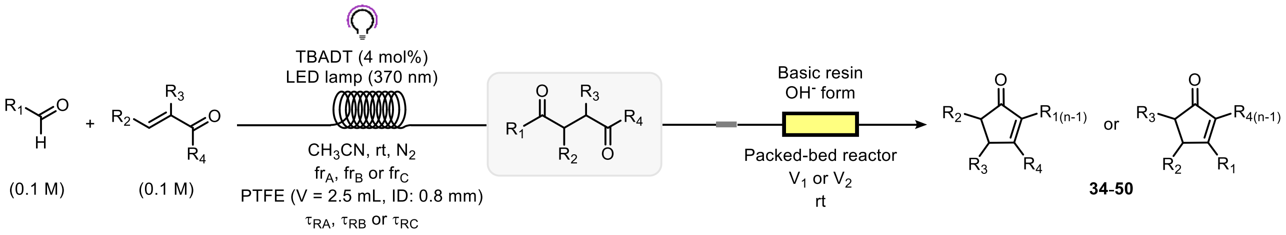

General Procedure 3 (GP3): A solution of aldehyde (0.1 M, 0.5 mmol), enone (0.1 M, 0.5 mmol) and TBADT ($4\cdot10^{-3}$ M, 4 mol%) in 5 mL of CH_3CN was prepared in a volumetric flask, N_2-bubbled (2 min) and charged into a coiled tubing reservoir (PTFE, internal diameter: 0.8 mm). By means of a peristaltic pump, the reaction mixture was pushed into a PTFE photoreactor (V_R = 2.5 mL) at the chosen flowrate (f.r.$_A$ = 5 mL h^{-1}, f.r.$_B$ = 2 mL h^{-1} or f.r.$_C$ = 1 mL h^{-1}) and irradiated with a 40 W Kessil lamp (λ = 370 nm, full intensity) by utilizing the reactor shown in Figure S3. The photoreactor was mounted on an aluminium support, which enables to control the temperature of the photocatalytic step thanks to a dedicated heat sink module. Thus, an ice bath was adopted in the case of aldehyde **S1m** to work at 0°C and avoid any competitive thermal decarbonylation pathway.[11] The packed-bed reactor was prepared by filling an Omnifit EZ SolventPlus glass column (7.8 mm internal diameter, 100 mm length and adjustable endpieces) with a basic resin (macroporous anionic resin AG-MP1, hydroxide form). The outflow of the photoreactor was directed through the packed-bed reactor, which was then flushed with CH_3CN. The

crude was collected at the end of the packed bed reactor in a round-bottomed flask under air, then the solvent was removed under reduced pressure. Next, the crude was purified by flash chromatography on silica gel to afford the products (**34-50**).

Preparation and maintenance of the packed-bed reactor: An Omnifit EZ SolventPlus glass column (7.8 mm internal diameter, 100 mm length and adjustable endpieces) was filled with 2 grams of a macroporous anionic resin AG-MP1, chloride form. The resin was activated through a treatment with 50 mL of a NaOH solution (1 M) to promote the anion exchange between Cl^- and OH^- counterions. Then it was washed with 50 mL of water until neutrality and flushed with CH_3CN. At the end of this process, the endpieces were adjusted to pack the bed of the reactor and its volume was set at 3.5 mL (V_1), which corresponded to the volume occupied by the resin (covering a length of 65 mm in the column). When a larger volume (V_2 = 7 mL) was needed for the telescoped reaction, two identical packed-bed reactors were connected in series (see Figure S3). The same batch of resin was employed for the whole work maintaining the same performances, provided that each packed-bed reactor was re-activated by washing the resin as described above, every three reaction runs.

*6.4. Scale-up for the synthesis of compound **1** in continuous-flow*

NB: upon prolonged operation of the reactor (ca. 1 mmol of material processed), we observed the formation of a fine blue precipitate (reduced decatungstate) in the second reactor. Therefore, to prevent clogging, we precautionally added 5% water to the amine and PTSA solution.

15 mL of a CH_3CN solution of heptanal (0.1 M), methyl vinyl ketone (0.1 M) and TBADT ($4\cdot10^{-3}$ M) were prepared in a volumetric flask and N_2-bubbled for 15 minutes. The solution was withdrawn with a disposable syringe and mounted on a syringe pump. The solution was pushed into a 2.5 mL FEP photoreactor at 0.5 $mL\cdot min^{-1}$ and irradiated with a 40 W Kessil lamp (λ = 390, full intensity) by utilizing the Uflow reactor (Figure S1). Parallelly, in vial a stock solution of ethanolamine (1 M) and PTSA (0.125 M) in CH_3CN:H_2O (95:5) was prepared. This stock solution was taken up with a syringe and mounted on a syringe pump (Feed B). The blue outflow of the photoreactor was then mixed with Feed B (pumped at 0.2 mL min^{-1}) through a PEEK T-mixer. The outflow of the T-mixer was pushed into 20 mL FEP reactor kept at 60°C. The crude was collected at the end of the tubular reactor in a round-bottomed flask containing saturated NH_4Cl solution as the quenching agent under air. After extraction with DCM, the solvent was removed under reduced pressure. Next, the crude was purified by flash chromatography on silica to afford the 2-(2-hexyl-5-methyl-1H-pyrrol-1-yl)ethan-1-ol (**1**), 200 mg, 64% yield after isolation.

*6.5. Scale-up for the synthesis of compound **36** in continuous-flow*

A solution of 3-phenylpropionaldehyde (0.1 M, 1.5 mmol), methyl vinyl ketone (0.1 M, 1.5 mmol) and TBADT ($4 \cdot 10^{-3}$ M, 4 mol%) in 15 mL of CH_3CN was prepared in a volumetric flask, N_2-bubbled (15 min) and charged into a coiled tubing reservoir (PTFE, internal diameter: 0.8 mm). By means of a peristaltic pump, it was pushed into a 2.5 mL PTFE photoreactor at 5 mL h^{-1} and irradiated with a 40 W Kessil lamp (λ = 370 nm, full intensity) by utilizing the reactor shown in Figure S3. The outlet of the photoreactor was directed through the packed-bed reactor (two column connected in series for a total volume 7 mL, as illustrated in Figure S3). The crude was collected at the end of the packed bed reactor in a round-bottomed flask under air, then the solvent was removed under reduced pressure. Next, the crude was purified by flash chromatography on silica gel to afford 2-benzyl-3-methylcyclopent-2-en-1-one (**36**), 177 mg, 62% yield after isolation.

7. Characterization data for products

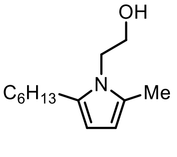

2-(2-hexyl-5-methyl-1H-pyrrol-1-yl)ethan-1-ol (1)

Prepared following GP1. The crude was purified by flash chromatography on silica (Hexane/EtOAc 3:1) to afford the product as a yellow oil (14.6 mg, 70% after isolation).

^{1}H NMR (400 MHz, $CDCl_3$) δ 5.82 (s, 2H), 3.91 (t, *J* = 6 Hz, 2H), 3.74 (td, *J* = 6, 2 Hz, 2H), 2.54 (t, *J* = 8 Hz, 2H), 2.25 (s, 3H), 1.96 (s, 1H), 1.65 (quint, *J* = 8 Hz, 2H), 1.47 – 1.27 (m, 6H), 0.92 (t, *J* = 6 Hz, 3H). **^{13}C NMR (101 MHz, $CDCl_3$)** δ 133.3, 128.0, 105.7, 104.2, 62.3, 45.4, 31.9, 29.4, 28.8, 26.9, 22.7, 14.2, 12.7. HRMS (ESI) m/z: $[M+H]^+$ calcd. for $C_{13}H_{24}NO^+$ 210.1852; found 210.1855.

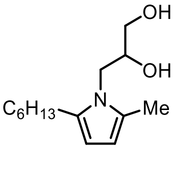

3-(2-hexyl-5-methyl-1H-pyrrol-1-yl)propane-1,2-diol (2)

Prepared following GP1. The solution of **S3b** and PTSA was prepared in CH_3CN:EtOH 9:1. The crude was purified by flash chromatography on silica (Hexane/EtOAc 1:1) to afford the product as an orange oil (16.5 mg, 69% after isolation).

^{1}H NMR (400 MHz, $CDCl_3$) δ 5.87 – 5.77 (m, 2H), 3.98 – 3.91 (m, 1H), 3.91 – 3.78 (m, 2H), 3.71 (dd, *J* = 11, 3 Hz, 1H), 3.55 (dd, *J* = 11, 6 Hz, 1H), 2.58 – 2.45 (m, 2H), 2.32 (s, 1H), 2.23 (s, 3H), 2.01 (s, 1H), 1.63 (quint, *J* = 8 Hz, 2H), 1.43 – 1.25 (m, 6H), 0.90 (t, *J* = 7 Hz, 3H). **^{13}C NMR (101 MHz, $CDCl_3$)** δ 133.5, 128.2, 106.1, 104.5, 71.7, 64.1, 46.0, 31.9, 29.5, 28.8, 26.9, 22.8, 14.2, 12.9. HRMS (ESI) m/z: $[M+H]^+$ calcd. for $C_{14}H_{26}NO_2^+$ 240.1958; found 240.1956.

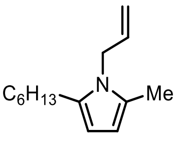

1-allyl-2-hexyl-5-methyl-1H-pyrrole (3)

Prepared following GP1. The crude was purified by flash chromatography on silica (Hexane/DCM 1:1) to afford the product as a pale yellow oil (12.2 mg, 60% after isolation).

^{1}H NMR (400 MHz, $CDCl_3$) δ 5.88 (ddt, *J* = 17, 10, 4 Hz, 1H), 5.83 – 5.76 (m, 2H), 5.09 (dq, *J* = 10, 1 Hz, 1H), 4.72 (dq, *J* = 17, 2 Hz, 1H), 4.37 (dt, *J* = 4, 2 Hz, 2H), 2.53 – 2.41 (m, 2H), 2.18 (s, 3H), 1.61 (quint, *J* = 7 Hz, 2H), 1.41 – 1.26 (m, 6H), 0.89 (t, *J* = 7 Hz, 3H). **^{13}C NMR (101 MHz, $CDCl_3$)** δ 134.5, 132.9, 127.7, 115.7, 105.2, 104.0, 45.6, 31.9, 29.4, 29.0, 26.7, 22.8, 14.2, 12.3. HRMS (ESI) m/z: $[M+H]^+$ calcd. for $C_{14}H_{24}N^+$ 206.1903; found 206.1901.

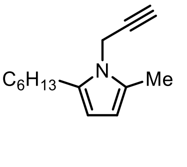

2-hexyl-5-methyl-1-(prop-2-yn-1-yl)-1H-pyrrole (4)

Prepared following GP1. The crude was purified by flash chromatography on silica (DCM) to afford the product as a yellow oil (12.1 mg, 60% after isolation).

^{1}H NMR (600 MHz, $CDCl_3$) δ 5.82 (d, *J* = 3 Hz, 1H), 5.80 (d, *J* = 3 Hz, 1H), 4.51 (d, *J* = 2 Hz, 2H), 2.64 – 2.51 (m, 2H), 2.29 (s, 3H), 2.25 (t, *J* = 2 Hz, 1H), 1.65 (quint, *J* = 8 Hz, 2H), 1.41 (quint, *J* = 7 Hz, 2H), 1.37 – 1.29 (m, 4H), 0.90 (t, *J* = 7 Hz, 3H). **^{13}C NMR (151 MHz, $CDCl_3$)** δ 132.7, 127.7, 105.9, 104.8, 79.0, 72.0, 32.8, 31.8, 29.3, 29.0, 26.8, 22.8, 14.2, 12.3. HRMS (ESI) m/z: $[M+H]^+$ calcd. for $C_{14}H_{22}N^+$ 204.1747; found: 204.1745.

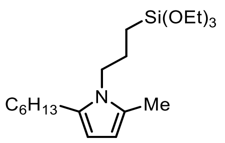

2-hexyl-5-methyl-1-(3-(triethoxysilyl)propyl)-1H-pyrrole (5)

Prepared following GP1. The crude was purified by flash chromatography on silica (Hexane/EtOAc 10:1) to afford the product as a yellow oil (16.5 mg, 45% after isolation).

^{1}H-NMR (400 MHz, CDCl$_3$) δ 5.88 – 5.67 (m, 2H), 3.82 (q, *J* = 7 Hz, 6H), 3.75 – 3.66 (m, 2H), 2.58 – 2.45 (m, 2H), 2.22 (s, 3H), 1.78 – 1.68 (m, 2H), 1.67 – 1.59 (m, 2H), 1.44 – 1.28 (m, 6H), 1.22 (t, *J* = 7 Hz, 9H), 0.90 (t, *J* = 7 Hz, 3H), 0.68 – 0.60 (m, 2H). **^{13}C NMR (101 MHz, CDCl$_3$)** δ 132.6, 127.3, 105.2, 103.7, 58.6, 46.0, 31.9, 29.5, 29.0, 26.8, 24.7, 22.8, 18.4, 14.2, 12.6, 7.8. HRMS (ESI) m/z: $[M+H]^+$ calcd. for $C_{20}H_{40}NO_3Si^+$ 370.2772; found 370.2768.

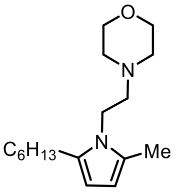

4-(2-(2-hexyl-5-methyl-1H-pyrrol-1-yl)ethyl)morpholine (6)

Prepared following GP1. The crude was purified by flash chromatography on silica (Hexane/EtOAc 3:1) to afford the product as an orange oil (15.2 mg, 55% % after isolation).

^{1}H NMR (600 MHz, CDCl$_3$) δ 5.79 (d, *J* = 3 Hz, 1H), 5.78 (d, *J* = 3 Hz, 1H), 3.95 – 3.83 (m, 2H), 3.78 – 3.67 (m, 4H), 2.61 – 2.43 (m, 8H), 2.24 (s, 3H), 1.64 (quint, *J* = 8 Hz, 2H), 1.42 – 1.29 (m, 6H), 0.92 – 0.88 (m, 3H).**^{13}C NMR (151 MHz, CDCl$_3$)** δ 132.7, 127.4, 105.6, 104.2, 67.0, 59.0, 54.2, 41.0, 31.9, 29.5, 29.0, 26.8, 22.8, 14.2, 12.6. HRMS (ESI) m/z: $[M+H]^+$ calcd. for $C_{17}H_{31}N_2O^+$ 279.2431, found 279.2431.

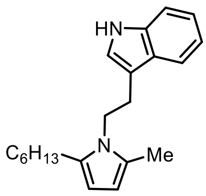

3-(2-(2-hexyl-5-methyl-1H-pyrrol-1-yl)ethyl)-1H-indole (7)

Prepared following GP1. The solution of **S3f** and PTSA was prepared in $CH_3CN:H_2O$ 9:1. The crude was purified by flash chromatography on silica (Cy/EtOAc 9:1) to afford the product as a red oil (14.6 mg, 47% after isolation).

^{1}H NMR (400 MHz, CDCl$_3$) δ 8.00 (s, 1H), 7.59 (d, *J* = 8 Hz, 1H), 7.39 (d, *J* = 8 Hz, 1H), 7.23 (t, *J* = 8 Hz, 1H), 7.16 (t, *J* = 7 Hz, 1H), 6.93 (d, *J* = 2 Hz, 1H), 5.88 – 5.77 (m, 2H), 4.07 – 4.00 (m, 2H), 3.10 – 3.03 (m, 2H), 2.52 (t, *J* = 8 Hz, 2H), 2.24 (s, 3H), 1.64 (quint, *J* = 8 Hz, 2H), 1.43 – 1.28 (m, 6H), 0.90 (t, *J* = 7 Hz, 3H).**^{13}C NMR (101 MHz, CDCl$_3$)** δ 136.4, 132.7, 127.4, 127.4, 122.4, 122.0, 119.7, 118.6, 113.0, 111.4, 105.4, 103.9, 44.3, 31.9, 29.5, 29.1, 27.2, 26.9, 22.8, 14.3, 12.6. HRMS (ESI) m/z: $[M+H]^+$ calcd. for $C_{21}H_{29}N_2^+$ 309.2325; found 309.2322.

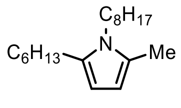

2-hexyl-5-methyl-1-octyl-1H-pyrrole (8)

Prepared following GP1. The crude was purified by flash chromatography on silica (Hexane/EtOAc 40:1) to afford the product as a yellow oil (18.6 mg, 67% after isolation).

^{1}H NMR (400 MHz, CDCl$_3$) δ 5.90 – 5.69 (m, 2H), 3.77 – 3.63 (m, 2H), 2.59 – 2.44 (m, 2H), 2.22 (s, 3H), 1.70 – 1.55 (m, 4H), 1.43 – 1.24 (m, 16H), 0.95 – 0.86 (m, 6H). **^{13}C NMR (101 MHz, CDCl$_3$)** δ 132.6, 127.2, 105.1, 103.7, 43.7, 31.9, 31.9, 31.3, 29.5, 29.4, 29.3, 29.0, 27.2, 26.9, 22.8, 14.3, 14.2, 12.6. HRMS (ESI) m/z: $[M+H]^+$ calcd. for $C_{19}H_{36}N^+$ 278.2842; found: 278.2847.

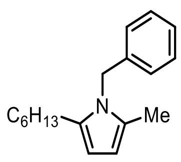

1-benzyl-2-hexyl-5-methyl-1H-pyrrole (9)

Prepared following GP1. The crude was purified by flash chromatography on silica (Hexane/EtOAc 9:1) to afford the product as a yellow oil (15.5 mg, 61% after isolation).

^{1}H NMR (400 MHz, CDCl$_3$) δ 7.29 (t, *J* = 7 Hz, 2H), 7.23 (t, *J* = 7 Hz, 1H), 6.88 (d, *J* = 7 Hz, 2H), 5.92 – 5.85 (m, 2H), 5.02 (s, 2H), 2.44 (t, *J* = 8 Hz, 2H), 2.13 (s, 3H), 1.56 (quint, *J* = 7 Hz, 2H), 1.35 – 1.21 (m, 6H), 0.86 (t, *J* = 7 Hz, 3H).

^{13}C NMR (101 MHz, $CDCl_3$) δ 138.9, 133.2, 128.8, 128.0, 127.1, 125.7, 105.6, 104.4, 46.7, 31.8, 29.3, 29.0, 26.8, 22.7, 14.2, 12.5. HRMS (ESI) m/z: $[M+H]^+$ calcd. for $C_{18}H_{26}N^+$ 256.2060; found 256.2059.

2-hexyl-5-methyl-1-(4-(trifluoromethyl)benzyl)-1H-pyrrole (10)

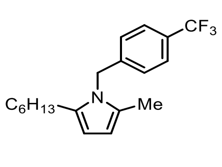

Prepared following GP1. The solution of **S3j** and PTSA was prepared in $CH_3CN:H_2O$ 9:1. The crude was purified by flash chromatography on silica (Hexane/EtOAc 9:1) to afford the product as a yellow oil (20 mg, 62% after isolation).
^{1}H NMR (400 MHz, $CDCl_3$) δ 7.55 (d, *J* = 8 Hz, 2H), 6.96 (d, *J* = 8 Hz, 2H), 5.95 – 5.85 (m, 2H), 5.07 (s, 2H), 2.41 (t, *J* = 8 Hz, 2H), 2.11 (s, 3H), 1.54 (quint, *J* = 7 Hz, 2H), 1.37 – 1.19 (m, 6H), 0.85 (t, *J* = 7 Hz, 3H). **^{13}C NMR (101 MHz, $CDCl_3$)** δ 143.1, 133.1, 129.6 (q, *J* = 33 Hz), 127.8, 126.0, 125.8 (q, *J* = 4 Hz), 124.2 (q, *J* = 272 Hz), 106.1, 104.9, 46.4, 31.8, 29.2, 29.0, 26.8, 22.7, 14.2, 12.4. **^{14}F NMR (565 MHz, $CDCl_3$)** δ -62.4. HRMS (ESI) m/z: $[M+H]^+$ calcd. for $C_{19}H_{25}F_3N^+$ 324.1934; found 324.1934.

1-(furan-2-ylmethyl)-2-hexyl-5-methyl-1H-pyrrole (11)

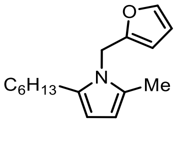

Prepared following GP1. The crude was purified by flash chromatography on silica (Cy/EtOAc 9:1) to afford the product as a yellow oil (13.2 mg, 54% after isolation).
^{1}H NMR (400 MHz, $CDCl_3$) δ 7.33 (d, *J* = 2 Hz, 1H), 6.28 (dd, *J* = 3, 2 Hz, 1H), 5.98 (d, *J* = 3 Hz, 1H), 5.86 – 5.78 (m, 2H), 4.92 (s, 2H), 2.59 – 2.53 (m, 2H), 2.25 (s, 3H), 1.61 (quint, *J* = 8 Hz, 2H), 1.42 – 1.27 (m, 6H), 0.92 – 0.87 (m, 3H). **^{13}C NMR (101 MHz, $CDCl_3$)** δ 151.7, 142.2, 133.1, 128.0, 110.4, 107.0, 105.6, 104.3, 40.6, 31.9, 29.4, 28.9, 26.7, 22.8, 14.2, 12.5. HRMS (ESI) m/z: $[M+H]^+$ calcd. for $C_{16}H_{24}NO^+$ 246.1852; found 246.1848.

2-hexyl-1-(4-methoxyphenyl)-5-methyl-1H-pyrrole (12)

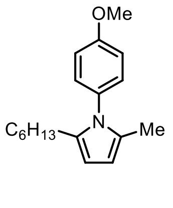

Prepared following GP1. The solution of **S3l** and PTSA was prepared in CH_3CN:EtOH 9:1. The crude was purified by flash chromatography on silica (Hexane/EtOAc 13:1) to afford the product as an orange oil (17.9 mg, 66% after isolation).
^{1}H NMR (400 MHz, $CDCl_3$) δ 7.13 (d, *J* = 9 Hz, 2H), 6.97 (d, *J* = 9 Hz, 2H), 5.97 – 5.83 (m, 2H), 3.87 (s, 3H), 2.32 (t, *J* = 8 Hz, 2H), 2.01 (s, 3H), 1.46 (quint, *J* = 8 Hz, 2H), 1.28 – 1.17 (m, 6H), 0.84 (t, *J* = 7 Hz, 3H). **^{13}C NMR (101 MHz, $CDCl_3$)** δ 159.0, 134.5, 131.9, 129.5, 129.0, 114.3, 105.3, 104.3, 55.6, 31.7, 29.2, 29.1, 27.2, 22.7, 14.2, 13.0. HRMS (ESI) m/z: $[M+H]^+$ calcd. for $C_{18}H_{26}NO^+$ 272.2009; found 272.2010.

2-(2-hexyl-5-methyl-1H-pyrrol-1-yl)-5-methylphenol (13)

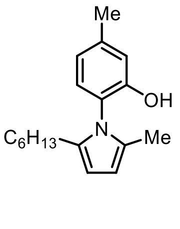

Prepared following GP1. The solution of **S3m** and PTSA was prepared in $CH_3CN:H_2O$ 9:1. The crude was purified by flash chromatography on silica (Hexane/EtOAc 9:1) to afford the product as red oil (13.5 mg, 50% after isolation). **^{1}H NMR (600 MHz, $CDCl_3$)** δ 6.99 (d, *J* = 8 Hz, 1H), 6.88 (d, *J* = 2 Hz, 1H), 6.79 (dd, *J* = 8, 2 Hz, 1H), 6.00 – 5.92 (m, 2H), 4.98 (s, 1H), 2.38 (s, 3H), 2.30 – 2.21 (m, 2H), 1.96 (s, 3H), 1.45 (quint, *J* = 7 Hz, 2H), 1.30 – 1.13 (m, 6H), 0.84 (t, *J* = 7 Hz, 3H). **^{13}C NMR (151 MHz, $CDCl_3$)** δ 152.5, 140.5, 134.4, 129.1, 129.0, 122.6, 121.6, 116.7, 106.8, 105.9, 31.7, 29.1, 29.0, 26.8, 22.6, 21.5, 14.2, 12.4. HRMS (ESI) m/z: $[M+H]^+$ calcd. for $C_{18}H_{26}NO^+$ 272.2009; found 272.2013.

8-(2-hexyl-5-methyl-1H-pyrrol-1-yl)-5-methoxyquinoline (14)

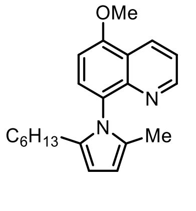

Prepared following GP1. The crude was purified by flash chromatography on silica (Hexane/ EtOAc 21:4) to afford the product as a yellow oil (21.4 mg, 66% after isolation).

^{1}H NMR (400 MHz, CDCl$_3$) δ 8.93 (dd, *J* = 4, 2 Hz, 1H), 8.63 (dd, *J* = 8, 2 Hz, 1H), 7.55 (d, *J* = 8 Hz, 1H), 7.41 (dd, *J* = 8, 4 Hz, 1H), 6.92 (d, *J* = 8 Hz, 1H), 6.05 – 5.98 (m, 2H), 4.08 (s, 3H), 2.20 (t, *J* = 8 Hz, 2H), 1.90 (s, 3H), 1.43 – 1.35 (m, 2H), 1.18 – 1.03 (m, 6H), 0.77 (t, *J* = 7 Hz, 3H). **^{13}C NMR (101 MHz, CDCl$_3$)** δ 155.2, 151.6, 146.1, 135.2, 130.9, 130.0, 129.9, 129.4, 121.4, 120.8, 105.3, 104.3, 103.5, 56.0, 31.6, 29.1, 28.9, 27.1, 22.6, 14.1, 12.8. HRMS (ESI) m/z: [M+H]$^+$ calcd. for $C_{21}H_{27}N_2O^+$ 323.2118; found 323.2116.

2-(2-cyclopropyl-5-methyl-1H-pyrrol-1-yl)ethan-1-ol (15)

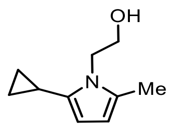

Prepared following GP1. The crude was purified by flash chromatography on silica (Hexane/EtOAc 3:1) to afford the product as a colourless oil (6.0 mg, 36% after isolation). **^{1}H NMR (400 MHz, CDCl$_3$)** δ 5.76 (d, *J* = 3 Hz, 1H), 5.69 (d, *J* = 3 Hz, 1H), 4.10 (t, *J* = 6 Hz, 2H), 3.87 (t, *J* = 6 Hz, 2H), 2.24 (s, 3H), 1.75 – 1.69 (m, 1H), 1.60 (s, 1H), 0.85 – 0.79 (m, 2H), 0.63 – 0.56 (m, 2H). **^{13}C NMR (101 MHz, CDCl$_3$)** δ 134.9, 128.7, 105.4, 103.8, 62.5, 45.8, 12.8, 7.5, 6.3. HRMS (ESI) m/z: [M+H]$^+$ calcd. for $C_{10}H_{16}NO^+$ 166.1226; found 166.1233.

2-(2-methyl-5-phenethyl-1H-pyrrol-1-yl)ethan-1-ol (16)

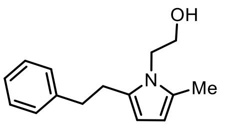

Prepared following GP1. The crude was purified by flash chromatography on silica (Hexane/EtOAc 4:1) to afford the product as a yellow oil (14.4 mg, 63% after isolation).

^{1}H NMR (600 MHz, CDCl$_3$) δ 7.31 (t, *J* = 7 Hz, 2H), 7.25 – 7.19 (m, 3H), 5.91 (d, *J* = 3 Hz, 1H), 5.86 (d, *J* = 3 Hz, 1H), 3.90 (t, *J* = 6 Hz, 2H), 3.77 – 3.71 (m, 2H), 3.01 – 2.94 (m, 2H), 2.89 – 2.83 (m, 2H), 2.25 (s, 3H), 1.63 (s, 1H). **^{13}C NMR (151 MHz, CDCl$_3$)** δ 142.0, 132.4, 128.6, 128.5, 128.3, 126.2, 105.9, 104.6, 62.4, 45.5, 35.3, 29.1, 12.8. HRMS (ESI) m/z: [M+H]$^+$ calcd. for $C_{15}H_{20}NO^+$ 230.1539; found 230.1536.

benzyl (2-(1-(2-hydroxyethyl)-5-methyl-1H-pyrrol-2-yl)ethyl)carbamate (17)

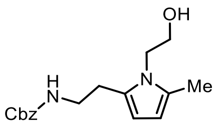

Prepared following GP1. The crude was purified by flash chromatography on silica (Hexane/EtOAc 1:1) to afford the product as a yellow oil (13.6 mg, 45% after isolation).

^{1}H NMR (600 MHz, CDCl$_3$) δ 7.38 – 7.31 (m, 5H), 5.82 (s, 2H), 5.08 (s, 3H), 3.92 (t, *J* = 5 Hz, 2H), 3.76 (t, *J* = 6 Hz, 2H), 3.44 (q, *J* = 7 Hz, 2H), 2.79 (t, *J* = 7 Hz, 2H), 2.33 (s, 1H), 2.23 (s, 3H). **^{13}C NMR (151 MHz, CDCl$_3$)** δ 156.7, 136.6, 129.1, 128.7, 128.6, 128.2, 128.2, 106.2, 105.6, 66.8, 62.3, 45.6, 40.6, 27.3, 12.7. HRMS (ESI) m/z: [M+H]$^+$ calcd. for $C_{17}H_{23}N_2O_3^+$ 303.1703; found 303.1707.

2-(2-(((tert-butyldimethylsilyl)oxy)methyl)-5-methyl-1H-pyrrol-1-yl)ethan-1-ol (18)

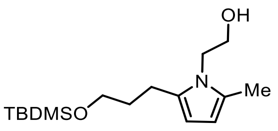

Prepared following GP1. The crude was purified by flash chromatography on silica (Hexane/ EtOAc 3:1) to afford the product as a colourless oil (18.9 mg, 64% after isolation).

^{1}H NMR (400 MHz, CDCl$_3$) δ 5.85 – 5.76 (m, 2H), 3.94 (t, *J* = 6 Hz, 2H), 3.78 (t, *J* = 6 Hz, 2H), 3.70 (t, *J* = 6 Hz, 2H), 2.61 (t, *J* = 8 Hz, 2H), 2.24 (s, 3H), 1.91 – 1.80 (m, 2H), 1.62 (s, 1H), 0.90 (s, 9H), 0.06 (s, 6H). **^{13}C NMR (101 MHz, CDCl$_3$)** δ 132.6, 128.1, 105.8, 104.4, 62.7, 62.4, 45.5, 32.1,

26.1, 23.1, 18.5, 12.8, -5.1. HRMS (ESI) calcd. for $C_{16}H_{32}NO_2Si^+$ 298.2197, found 298.2203.

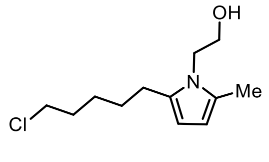

2-(2-(5-chloropentyl)-5-methyl-1H-pyrrol-1-yl)ethan-1-ol (19)

Prepared following GP1. The crude was purified by flash chromatography on silica (Hexane/ EtOAc 3:1) to afford the product as a yellow oil (16.1 mg, 70% after isolation).

^{1}H NMR (600 MHz, CDCl$_3$)) δ 5.82 (d, J = 3 Hz, 1H), 5.80 (d, J = 3 Hz, 1H), 3.93 (t, J = 6 Hz, 2H), 3.78 (t, J = 6 Hz, 2H), 3.55 (t, J = 7 Hz, 2H), 2.57 (t, J = 8 Hz, 2H), 2.24 (s, 3H), 1.83 (p, J = 7 Hz, 2H), 1.68 (quint, J = 8 Hz, 2H), 1.59 – 1.52 (m, 2H), 1.26 (s, 1H).**^{13}C NMR (151 MHz, CDCl$_3$)** δ 132.7, 128.1, 105.9, 104.5, 62.4, 45.5, 45.1, 32.6, 28.2, 27.0, 26.7, 12.7. HRMS (ESI) m/z: $[M+H]^+$ calcd. for $C_{12}H_{21}ClNO^+$ 230.1306; found 230.1309.

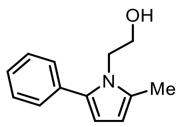

2-(2-methyl-5-phenyl-1H-pyrrol-1-yl)ethan-1-ol (20)

Prepared following GP1. The crude was purified by flash chromatography on silica (Hexane/EtOAc 20:1) to afford the product as a colourless oil (11.2 mg, 56% after isolation).

^{1}H NMR (600 MHz, CDCl$_3$) δ 7.41 – 7.35 (m, 4H), 7.33 – 7.27 (m, 1H), 6.12 (d, J = 3 Hz, 1H), 5.97 (d, J = 3 Hz, 1H), 4.10 (t, J = 6 Hz, 2H), 3.62 (t, J = 6 Hz, 2H), 2.34 (s, 3H), 1.38 (s, 1H).**^{13}C NMR (101 MHz, CDCl$_3$)** δ 134.2, 134.2, 130.6, 129.3, 128.6, 127.0, 108.7, 107.3, 62.4, 46.0, 13.1. Characterization data are in accordance with the literature. [12] HRMS (ESI) m/z: $[M+H]^+$ calcd. for $C_{13}H_{16}NO^+$ 202.1226; found 202.1226.

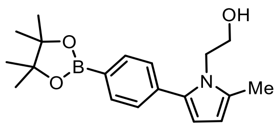

2-(2-methyl-5-(4-(4,4,5,5-tetramethyl-1,3,2-dioxaborolan-2-yl)phenyl)-1H-pyrrol-1-yl)ethan-1-ol (21)

Prepared following GP1. The crude was purified by flash chromatography on silica (Hexane/ EtOAc 6:1) to afford the product as a yellow oil (10.9 mg, 33% after isolation).

^{1}H NMR (400 MHz, CDCl$_3$) δ 7.83 (d, J = 8 Hz, 2H), 7.40 (d, J = 8 Hz, 2H), 6.15 (d, J = 3 Hz, 1H), 5.97 (d, J = 3 Hz, 1H), 4.13 (t, J = 6 Hz, 2H), 3.65 – 3.55 (m, 2H), 2.34 (s, 3H), 1.57 (s, 1H), 1.35 (s, 12H). **^{13}C NMR (101 MHz, CDCl$_3$)** δ 136.9, 135.4, 135.1, 134.0, 131.3, 128.2, 109.3, 107.5, 84.0, 62.5, 46.1, 25.0, 13.1. HRMS (ESI) calcd. for $C_{19}H_{27}BNO_3^+$ 328.2079, found 328.2073.

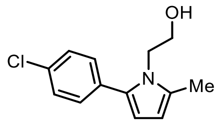

2-(2-(4-chlorophenyl)-5-methyl-1H-pyrrol-1-yl)ethan-1-ol (22)

Prepared following GP1. The crude was purified by flash chromatography on silica (Hexane/EtOAc 4:1) to afford the product as a colourless oil (9.7 mg, 41% after isolation).

^{1}H NMR (600 MHz, CDCl$_3$) δ 7.40 – 7.28 (m, 4H), 6.10 (d, J = 3 Hz, 1H), 5.96 (d, J = 3 Hz, 1H), 4.06 (t, J = 6 Hz, 2H), 3.62 (t, J = 6 Hz, 2H), 2.33 (s, 3H),1.46 (s, 1H). **^{13}C NMR (151 MHz, CDCl$_3$)** δ 133.0, 132.7, 131.0, 130.4, 129.6, 128.8, 109.0, 107.5, 62.3, 46.0, 13.0. HRMS (ESI) m/z: $[M+H]^+$ calcd. for $C_{13}H_{15}ClNO$ 236.0837; found 236.0836.

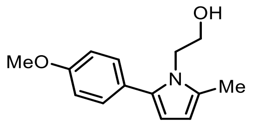

2-(2-(4-methoxyphenyl)-5-methyl-1H-pyrrol-1-yl)ethan-1-ol (23)

Prepared following GP1. The crude was purified by flash chromatography on silica (Hexane/EtOAc 6:1) to afford the product as a yellow oil (9.3 mg, 41% after isolation).

^{1}H NMR (400 MHz, CDCl$_3$) δ 7.33 – 7.28 (m, 2H), 6.95 – 6.90 (m, 2H), 6.05 (d, J = 3 Hz, 1H), 5.95 (d, J = 3 Hz, 1H), 4.05 (t, J = 6 Hz, 2H), 3.84 (s, 3H),

3.62 (t, J = 6 Hz, 2H), 2.33 (s, 3H), 1.50 (s, 1H). **^{13}C NMR (101 MHz, $CDCl_3$)** δ 158.9, 133.9, 130.7, 129.9, 126.7, 114.0, 108.1, 107.0, 62.4, 55.4, 45.9, 13.0. HRMS (ESI) m/z: $[M+H]^+$ calcd. for $C_{14}H_{18}NO_2^+$ 232.1332; found 232.1333.

2-(2-(furan-2-yl)-5-methyl-1H-pyrrol-1-yl)ethan-1-ol (24)

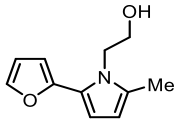

Prepared following GP1. The crude was purified by flash chromatography on silica (Hexane/EtOAc 6:1) to afford the product as a yellow oil (6.9 mg, 36% after isolation).

^{1}H NMR (600 MHz, $CDCl_3$) δ 7.41 (d, J = 2 Hz, 1H), 6.43 (dd, J = 3, 2 Hz, 1H), 6.34 (d, J = 3 Hz, 1H), 6.32 (d, J = 3 Hz, 1H), 5.93 (d, J = 3 Hz, 1H), 4.17 (t, J = 6 Hz, 2H), 3.84 (t, J = 6 Hz, 2H), 2.32 (s, 3H), 1.26 (s, 1H). **^{13}C NMR (151 MHz, $CDCl_3$)** δ 148.3, 141.3, 131.5, 123.8, 111.2, 109.1, 107.5, 106.2, 62.7, 46.9, 12.9. HRMS (ESI) m/z: $[M+H]^+$ calcd. for $C_{11}H_{14}NO_2^+$ 192.1019; found 192.1016.

methyl 2-hexyl-1-(2-hydroxyethyl)-5-methyl-1H-pyrrole-3-carboxylate (25)

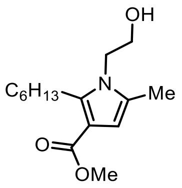

Prepared following GP1. The crude was purified by flash chromatography on silica (Hexane/EtOAc 12:1) to afford the product as a yellow oil (10.6 mg, 40% after isolation).

^{1}H NMR (600 MHz, $CDCl_3$) δ 6.26 – 6.24 (m, 1H), 3.96 (t, J = 6 Hz, 2H), 3.81 (t, J = 6 Hz, 2H), 3.76 (s, 3H), 2.95 – 2.89 (m, 2H), 2.21 (s, 3H), 1.52 (quint, J = 8 Hz, 2H), 1.38 (quint, J = 7 Hz, 2H), 1.34 – 1.26 (m, 4H), 0.90 – 0.86 (m, 3H). **^{13}C NMR (151 MHz, $CDCl_3$)** δ 165.9, 140.9, 127.9, 110.5, 108.2, 62.3, 50.7, 45.5, 31.8, 30.4, 29.6, 25.7, 22.8, 14.2, 12.6. HRMS (ESI) m/z: $[M+H]^+$ calcd. for $C_{15}H_{26}NO_3^+$ 268.1907; found 268.1904.

1-(4,5-dihexyl-1-(2-hydroxyethyl)-2-methyl-1H-pyrrol-3-yl)ethan-1-one (26)

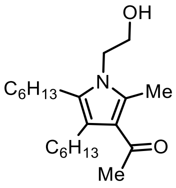

Prepared following GP1. The crude was purified by flash chromatography on silica (Hexane/ EtOAc 8:2) to afford the product as a yellow oil (13.1 mg, 39% after isolation).

^{1}H NMR (400 MHz, $CDCl_3$) δ 3.97 (t, J = 6 Hz, 2H), 3.80 (t, J = 6 Hz, 2H), 2.60 – 2.49 (m, 4H), 2.46 (s, 3H), 2.42 (s, 3H), 1.48 – 1.28 (m, 16H), 1.25 (s, 1H), 0.92 – 0.86 (m, 6H). **^{13}C NMR (101 MHz, $CDCl_3$)** δ 196.2, 134.1, 130.2, 121.5, 120.7, 62.2, 45.4, 32.5, 31.8, 31.7, 31.1, 31.0, 29.9, 29.8, 29.5, 26.1, 24.3, 22.8, 14.2, 14.2, 12.9. HRMS (ESI) m/z: $[M+H]^+$ calcd. for $C_{21}H_{38}NO_2^+$ 336.2897; found 336.2899.

2-hexyl-5-methylthiophene (27)

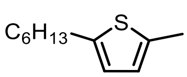

Prepared following GP2. The crude was purified by flash chromatography on silica (Hexane/EtOAc 24:1) to afford the product as a yellow oil (11.2 mg, 61% after isolation).

^{1}H NMR (400 MHz, $CDCl_3$) δ 6.54 (s, 2H), 2.73 (t, J = 8 Hz, 2H), 2.43 (s, 3H), 1.63 (quint, J = 8 Hz, 2H), 1.40 – 1.25 (m, 6H), 0.89 (t, J = 7 Hz, 3H). **^{13}C NMR (101 MHz, $CDCl_3$)** δ 143.8, 137.2, 124.7, 123.7, 31.9, 31.7, 30.3, 28.9, 22.7, 15.4, 14.2. HRMS (ESI) m/z: $[M+H]^+$ calcd. for $C_{11}H_{19}S^+$ 183.1202; found 183.1205.

2-(5-chloropentyl)-5-methylthiophene (28)

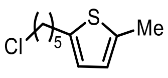

Prepared following GP2. The crude was purified by flash chromatography on silica (Hexane/EtOAc 24:1) to afford the product as a yellow oil (11.2 mg, 55% after isolation).

^{1}H NMR (600 MHz, $CDCl_3$) δ 6.56 – 6.52 (m, 2H), 3.53 (t, *J* = 7 Hz, 2H), 2.76 (t, *J* = 8 Hz, 2H), 2.43 (s, 3H), 1.86 – 1.75 (m, 2H), 1.67 (quint, *J* = 8 Hz, 2H), 1.54 – 1.47 (m, 2H). **^{13}C NMR (151 MHz, $CDCl_3$)** δ 143.1, 137.4, 124.7, 123.9, 45.1, 32.5, 31.1, 30.0, 26.5, 15.4. HRMS (ESI) m/z: $[M+H]^+$ calcd. for $C_{10}H_{16}ClS^+$ 203.0656; found 203.0655.

benzyl (2-(5-methylthiophen-2-yl)ethyl)carbamate (29)

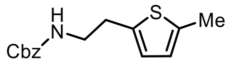

Prepared following GP2. The crude was purified by flash chromatography on silica (Hexane/EtOAc 6:1) to afford the product as a yellow oil (13.4 mg, 49% after isolation).

^{1}H NMR (600 MHz, $CDCl_3$) δ 7.39 – 7.30 (m, 5H), 6.59 (d, *J* = 3 Hz, 1H), 6.56 (d, *J* = 3 Hz, 1H), 5.10 (s, 2H), 4.88 (s, 1H), 3.45 (q, *J* = 6 Hz, 2H), 2.95 (t, *J* = 6 Hz, 2H), 2.43 (s, 3H).**^{13}C NMR (101 MHz, $CDCl_3$)** δ 156.4, 138.9, 138.6, 136.7, 128.7, 128.3,128.3, 125.4, 125.1, 66.8, 42.5, 30.6, 15.4. HRMS (ESI) m/z: $[M+H]^+$ calcd. for $C_{15}H_{18}NO_2S^+$ 276.1053; found 276.1055.

2-hexyl-4,5,6,7-tetrahydro-4,7-methanobenzo[b]thiophene (30)

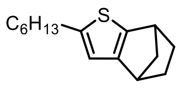

Prepared following GP2. The crude was purified by flash chromatography on silica (Hexane/EtOAc 49:1) to afford the product as yellow oil (14.1 mg, 60% after isolation).

^{1}H NMR (600 MHz, $CDCl_3$) δ 6.50 (s, 1H), 3.43 (s, 1H), 3.33 (s, 1H), 2.78 – 2.69 (m, 2H), 1.86 (dquint, *J* = 8, 2 Hz, 1H), 1.83 – 1.75 (m, 2H), 1.66 – 1.59 (m, 2H), 1.53 (dt, *J* = 8, 2 Hz, 1H), 1.38 – 1.25 (m, 6H), 1.00 – 0.93 (m, 2H), 0.88 (t, *J* = 7 Hz, 3H). **^{13}C NMR (151 MHz, $CDCl_3$)** δ 150.6, 146.7, 142.8, 117.7, 51.3, 42.9, 41.9, 32.1, 31.7, 30.8, 29.0, 28.0, 27.9, 22.7, 14.2. HRMS (ESI) m/z: $[M+H]^+$ calcd. for $C_{15}H_{23}S^+$ 235.1515; found 235.1513.

2-hexyl-4H-indeno[1,2-b]thiophene (31)

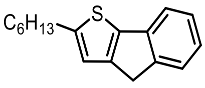

Prepared following GP2. The crude was purified by flash chromatography on silica (Hexane/EtOAc 49:1) to afford the product as yellow oil (11.4 mg, 45% after isolation).

^{1}H NMR (400 MHz, $CDCl_3$) δ 7.45 (d, *J* = 7 Hz, 1H), 7.40 (d, *J* = 8 Hz, 1H), 7.28 (t, *J* = 8 Hz, 1H), 7.14 (t, *J* = 7 Hz, 1H), 6.81 (s, 1H), 3.63 (s, 2H), 2.87 (t, *J* = 8 Hz, 2H), 1.72 (quint, *J* = 8 Hz, 2H), 1.46 – 1.25 (m, 6H), 0.93 – 0.87 (m, 3H). **^{13}C NMR (101 MHz, $CDCl_3$)** δ 149.0, 146.9, 145.5, 140.5, 139.6, 126.9, 125.0, 124.2, 120.1, 118.3, 34.4, 32.0, 31.8, 31.2, 28.9, 22.7, 14.2. HRMS (ESI) m/z: $[M+H]^+$ calcd. for $C_{17}H_{21}S^+$ 257.1358; found 257.1359.

2-(4-methoxyphenyl)-5-methylthiophene (32)

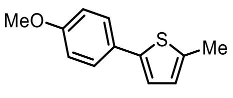

Prepared following GP2. The crude was purified by flash chromatography on silica (Hexane/EtOAc 59:1) to afford the product as a white solid (11.7 mg, 57% after isolation).

^{1}H NMR (600 MHz, $CDCl_3$) δ 7.48 (d, *J* = 9 Hz, 2H), 6.99 (d, *J* = 4 Hz, 1H), 6.90 (d, *J* = 9 Hz, 2H), 6.70 (dd, *J* = 4, 2 Hz, 1H), 3.83 (s, 3H), 2.50 (s, 3H). **^{13}C NMR (151 MHz, $CDCl_3$)** δ 159.0, 142.1, 138.6, 127.8, 126.9, 126.2, 122.0, 114.4, 55.5, 15.5. Characterization data are in accordance with the literature.[13] HRMS (ESI) m/z: $[M+H]^+$ calcd. for $C_{12}H_{13}OS^+$ 205.0682; found 205.0681.

m.p.: 92-94 °C (lit.[14] 94-95 °C).

2-(4-chlorophenyl)-5-methylthiophene (33)

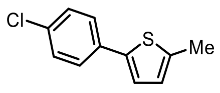

Prepared following GP2. The crude was purified by flash chromatography on silica (Cyclohexane) to afford the product as a white solid (9 mg, 43% after isolation).

^{1}H NMR (400 MHz, $CDCl_3$) δ 7.50 – 7.43 (m, 2H), 7.34 – 7.28 (m, 2H), 7.08 (d, *J* = 4 Hz, 1H), 6.72 (dq, *J* = 4, 1 Hz, 1H), 2.51 (d, *J* = 1 Hz, 3H). **^{13}C NMR (101 MHz, $CDCl_3$)** δ 140.8, 140.1, 133.4, 132.8, 129.1, 126.8, 126.5, 123.4, 15.6. Characterization data are in accordance with the literature.[15] HRMS (ESI) calcd. for $C_{11}H_{10}ClS^+$ 209.0186, found 209.0186.
m.p.: 106-108 °C.

3-methyl-2-pentylcyclopent-2-en-1-one (34)

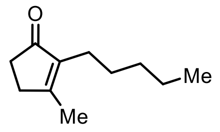

Prepared following GP3. The crude mixture was purified by flash chromatography on silica (cyclohexane/ethyl acetate 8:2) to afford the product as a yellowish oil (63 mg, 70% after isolation).
^{1}H NMR (400 MHz, $CDCl_3$) δ 2.47 – 2.44 (m, 2H), 2.35 – 2.30 (m, 2H), 2.13 (t, *J* = 8 Hz, 2H), 2.02 (d, *J* = 1 Hz, 3H), 1.39 – 1.16 (m, 6H), 0.84 (t, *J* = 7 Hz, 3H). **^{13}C NMR (101 MHz, $CDCl_3$)** δ 209.8, 170.2, 140.9, 34.4, 31.9, 31.6, 28.2, 23.1, 22.6, 17.3, 14.1. Characterization data are in accordance with the literature.[16] HRMS (ESI) calcd. for $C_{11}H_{19}O$ 167.1430, found 167.1428.

2-heptyl-3-methylcyclopent-2-en-1-one (35)

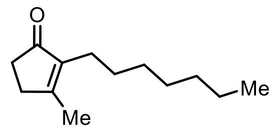

Prepared following GP3. The crude mixture was purified by flash chromatography on silica (cyclohexane/ethyl acetate 8:2) to afford the product as a white solid (90 mg, 86% after isolation).
^{1}H NMR (400 MHz, $CDCl_3$) δ 2.49 – 2.43 (m, 2H), 2.35 – 2.30 (m, 2H), 2.14 (t, *J* = 8 Hz, 2H), 2.02 (s, 3H), 1.42 – 1.14 (m, 10H), 0.84 (t, *J* = 7 Hz, 3H). **^{13}C NMR (101 MHz, $CDCl_3$)** δ 209.7, 170.0, 140.9, 34.4, 31.9, 31.6, 29.7, 29.2, 28.5, 23.1, 22.7, 17.3, 14.2. HRMS (ESI) m/z: $[M+H]^+$ calcd. for $C_{13}H_{23}O^+$ 195.1743; found 195.1742.
m.p.: 45 – 47°C.

2-benzyl-3-methylcyclopent-2-en-1-one (36)

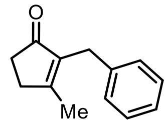

Prepared following GP3. The crude mixture was purified by flash chromatography on silica (cyclohexane/ethyl acetate 8:2) to afford the product as a yellowish oil (93 mg, 99% after isolation).
^{1}H NMR (400 MHz, $CDCl_3$) δ 7.35 – 7.18 (m, 5H), 3.60 (s, 2H), 2.59 – 2.57 (m, 2H), 2.49 – 2.41 (m, 2H), 2.13 (s, 3H).**^{13}C NMR (101 MHz, $CDCl_3$)** δ 209.0, 171.3, 139.7, 139.6, 128.5, 128.5, 126.1, 34.3, 31.8, 29.0, 17.6. Characterization data are in accordance with the literature.[17] HRMS (ESI) m/z: $[M+H]^+$ calcd. for $C_{13}H_{15}O^+$ 187.1117; found 187.1116.

3-cyclopropylcyclopent-2-en-1-one (37)

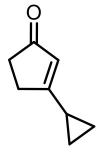

Prepared following GP3. The crude mixture was purified by flash chromatography on silica (cyclohexane/ethyl acetate 8:2) to afford the product as a yellowish oil (35 mg, 57% after isolation).
^{1}H NMR (400 MHz, $CDCl_3$) δ 5.91 (t, *J* = 2 Hz, 1H), 2.46 – 2.41 (m, 2H), 2.40 – 2.35 (m, 2H), 1.88 – 1.81 (m, 1H), 1.10 – 1.02 (m, 2H), 0.88 – 0.82 (m, 2H). **^{13}C NMR (101 MHz, $CDCl_3$)** δ 209.3, 185.5, 127.3, 35.0, 28.6, 14.6, 10.0. Characterization data are in accordance with the literature.[18] HRMS (ESI) m/z: $[M+H]^+$ calcd. for $C_8H_{11}O^+$ 123.0804; found 123.0804.

3-isopropylcyclopent-2-en-1-one (38)

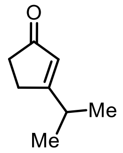

Prepared following GP3. The crude mixture was purified by flash chromatography on silica (petrol ether/ethyl acetate 8:2) to afford the product as a yellowish oil (37 mg, 60% after isolation).
^{1}H NMR (400 MHz, $CDCl_3$) δ 5.91 (q, *J* = 2 Hz, 1H), 2.66 – 2.56 (m, 3H), 2.40 – 2.36 (m, 2H), 1.16 (d, *J* = 7 Hz, 6H). **^{13}C NMR (101 MHz, $CDCl_3$)** δ 210.4, 188.6, 127.9, 35.4, 32.2, 29.5, 21.0. Characterization data are in accordance with the literature.[19] HRMS (ESI) m/z: $[M+H]^+$ calcd. for $C_8H_{13}O^+$ 125.0961; found 125.0958.

3-(4-methoxyphenyl)cyclopent-2-en-1-one (39)

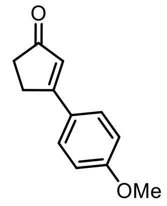

Prepared following GP3. The crude mixture was purified by flash chromatography on silica (petrol ether/ethyl acetate 8:2) to afford the product as a white solid (43 mg, 46% after isolation).

^{1}H NMR (400 MHz, CDCl$_3$) δ 7.61 (d, *J* = 9 Hz, 2H), 6.95 (m, 2H), 6.47 (t, *J* = 2 Hz, 1H), 3.86 (s, 3H), 3.03 – 2.98 (m, 2H), 2.59 – 2.53 (m, 2H). **^{13}C NMR (101 MHz, CDCl$_3$)** δ 209.4, 173.7, 162.2, 128.7, 126.9, 125.6, 114.4, 55.6, 35.4, 28.7. Characterization data are in accordance with the literature.[20] HRMS (ESI) m/z: [M+H]$^+$ calcd. for $C_{12}H_{13}O_2^+$ 189.0910; found 189.0907.

m.p.: 141 – 143°C (lit.[20] 140 – 144°C).

3-(4-chlorophenyl)cyclopent-2-en-1-one (40)

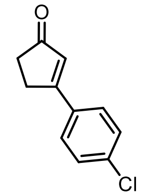

Prepared following GP3. The crude mixture was purified by flash chromatography on silica (cyclohexane/ethyl acetate 8:2) to afford the product as a yellowish solid (36 mg, 37% after isolation).

^{1}H NMR (400 MHz, CDCl$_3$) δ 7.60 – 7.54 (m, 2H), 7.44 – 7.39 (m, 2H), 6.54 (t, *J* = 2 Hz, 1H), 3.03 – 2.97 (m, 2H), 2.62 – 2.54 (m, 2H). **^{13}C NMR (101 MHz, CDCl$_3$)** δ 209.1, 172.4, 137.4, 132.7, 129.3, 128.2, 128.0, 35.4, 28.7. Characterization data are in accordance with the literature.[21] HRMS (ESI) m/z: [M+H]$^+$ calcd. for $C_{11}H_{10}ClO^+$ 193.0415; found 193.0414.

m.p.: 95 – 97°C.

3-(thiophen-2-yl)cyclopent-2-en-1-one (41)

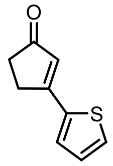

Prepared following GP3. The crude mixture was purified by flash chromatography on silica (cyclohexane/ethyl acetate 8:2) to afford the product as a white solid (51 mg, 62% after isolation).

^{1}H NMR (400 MHz, CDCl$_3$) δ 7.54 (dd, *J* = 5, 1 Hz, 1H), 7.45 (dd, *J* = 4, 1 Hz, 1H), 7.13 (dd, *J* = 5, 4 Hz, 1H), 6.37 (t, *J* = 2 Hz, 1H), 3.07 – 3.02 (m, 2H), 2.59 – 2.54 (m, 2H). **^{13}C NMR (101 MHz, CDCl$_3$)** δ 208.6, 166.7, 138.9, 130.3, 128.6, 128.5, 126.0, 35.2, 29.5. Characterization data are in accordance with the literature.[22] HRMS (ESI) m/z: [M+H]$^+$ calcd. for $C_9H_9OS^+$ 165.0369; found 165.0367.

2-benzyl-3-ethylcyclopent-2-en-1-one (42)

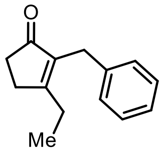

Prepared following GP3 from ethyl vinyl ketone (**S2f**) and hydrocinnamaldehyde (**S1c**). The crude mixture (1:1 isomeric mixture of products **42** and **42'**) was purified by flash chromatography on silica (cyclohexane/ethyl acetate 8:2) to afford the product as a yellowish oil (50 mg, 50% after isolation).

^{1}H NMR (400 MHz, CDCl$_3$) δ 7.35 – 7.18 (m, 5H), 3.61 (s, 2H), 2.64 – 2.44 (m, 6H), 1.15 (t, *J* = 8 Hz, 3H). **^{13}C NMR (101 MHz, CDCl$_3$)** δ 209.5, 176.4, 139.8, 139.0, 128.5, 128.5, 126.1, 34.3, 29.0, 28.8, 24.6, 11.7. HRMS (ESI) m/z: [M+H]$^+$ calcd. for $C_{14}H_{17}O^+$ 201.1274; found 201.1274.

2-methyl-3-phenethylcyclopent-2-en-1-one (42')

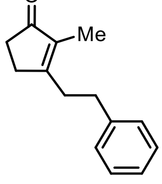

Prepared following GP3 from ethyl vinyl ketone (**S2f**) and hydrocinnamaldehyde (**S1c**). The crude mixture (1:1 isomeric mixture of products **42** and **42'**) was purified by flash chromatography on silica (cyclohexane/ethyl acetate 8:2) to afford the product as a yellowish oil (50 mg, 50% after isolation).

^{1}H NMR (400 MHz, CDCl$_3$) δ 7.32 – 7.13 (m, 5H), 2.84 (dd, *J* = 9, 6 Hz, 2H), 2.73 (dd, *J* = 9, 6 Hz, 2H), 2.50 – 2.45 (m, 2H), 2.39 – 2.32 (m, 2H), 1.59 (t, *J* = 2 Hz, 3H). **^{13}C NMR (101 MHz, CDCl$_3$)** δ 210.1, 172.3, 140.8, 136.9, 128.7, 128.3, 126.5, 34.3, 33.5, 33.2, 29.5, 8.0. Characterization data are in accordance with the literature.[23] HRMS (ESI) m/z: [M+H]$^+$ calcd. for $C_{14}H_{17}O^+$ 201.1274; found 201.1275.

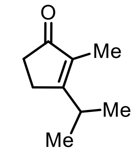

3-isopropyl-2-methylcyclopent-2-en-1-one (43)

Prepared following GP3. The crude mixture was purified by flash chromatography on silica (cyclohexane/ethyl acetate 8:2) to afford the product as a yellowish oil (64 mg, 92% after isolation).

^{1}H NMR (400 MHz, CDCl$_3$) δ 3.04 – 2.97 (m, 1H), 2.49 – 2.44 (m, 2H), 2.35 – 2.30 (m, 2H), 1.67 (t, *J* = 2 Hz, 3H), 1.10 (d, *J* = 7 Hz, 6H). **^{13}C NMR (101 MHz, CDCl$_3$)** δ 210.6, 178.8, 134.7, 33.9, 29.5, 24.9, 20.3, 8.0. Characterization data are in accordance with the literature.[24] HRMS (ESI) m/z: $[M+H]^+$ calcd. for $C_9H_{15}O^+$ 139.1117; found 139.1117.

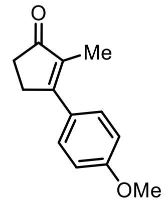

3-(4-methoxyphenyl)-2-methylcyclopent-2-en-1-one (44)

Prepared following GP3. The crude mixture was purified by flash chromatography on silica (cyclohexane/ethyl acetate 8:2) to afford the product as a yellowish oil (75 mg, 74% after isolation).

^{1}H NMR (400 MHz, CDCl$_3$) δ 7.55 – 7.49 (m, 2H), 7.00 – 6.94 (m, 2H), 3.85 (d, *J* = 1 Hz, 3H), 2.90 – 2.85 (m, 2H), 2.53 – 2.46 (m, 2H), 1.97 (t, *J* = 2 Hz, 3H). **^{13}C NMR (101 MHz, CDCl$_3$)** δ 209.8, 165.9, 160.7, 134.9, 129.4, 128.9, 114.1, 55.5, 34.0, 29.0, 10.2. Characterization data are in accordance with the literature.[25] HRMS (ESI) m/z: $[M+H]^+$ calcd. for $C_{13}H_{15}O_2^+$ 203.1067; found 203.1067.

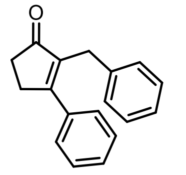

2-benzyl-3-phenylcyclopent-2-en-1-one (45)

Prepared following GP3. The crude mixture was purified by flash chromatography on silica (petrol ether/ethyl acetate 8:2) to afford the product as a yellowish oil (70 mg, 56% after isolation).

^{1}H NMR (400 MHz, CDCl$_3$) δ 7.33 – 7.24 (m, 5H), 7.13 – 6.98 (m, 5H), 3.62 (s, 2H), 2.89 – 2.79 (m, 2H), 2.51 – 2.41 (m, 2H). **^{13}C NMR (101 MHz, CDCl$_3$)** δ 209.3, 169.1, 139.4, 139.3, 136.4, 129.8, 128.8, 128.6, 128.4, 127.4, 126.2, 34.3, 30.1, 29.8. Characterization data are in accordance with the literature.[26] HRMS (ESI) m/z: $[M+H]^+$ calcd. for $C_{18}H_{17}O^+$ 249.1274; found 249.1274.

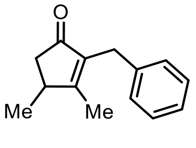

2-benzyl-3,4-dimethylcyclopent-2-en-1-one (46)

Prepared following GP3. The crude mixture was purified by flash chromatography on silica (petrol ether/ethyl acetate 8:2) to afford the product as a yellowish oil (98 mg, 98% after isolation).

^{1}H NMR (400 MHz, CDCl$_3$) δ 7.34 – 7.19 (m, 5H), 3.59 (d, *J* = 2 Hz, 2H), 2.86 – 2.78 (m, 1H), 2.70 (dd, *J* = 19, 7 Hz, 1H), 2.12 – 2.04 (m, 4H), 1.25 (d, *J* = 7 Hz, 3H). **^{13}C NMR (101 MHz, CDCl$_3$)** δ 208.0, 175.3, 139.5, 139.1, 128.4, 128.4, 126.0, 43.1, 37.4, 29.0, 19.1, 15.2. HRMS (ESI) m/z: $[M+H]^+$ calcd. for $C_{14}H_{17}O^+$ 201.1274; found 201.1273.

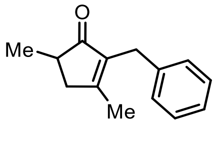

2-benzyl-3,5-dimethylcyclopent-2-en-1-one (47)

Prepared following GP3. The crude mixture was purified by flash chromatography on silica (hexane/ethyl acetate 8:2) to afford the product as a yellowish oil (72 mg, 72% after isolation).

^{1}H NMR (400 MHz, CDCl$_3$) δ 7.32 – 7.14 (m, 5H), 3.57 (s, 2H), 2.83 – 2.76 (m, 1H), 2.46 – 2.38 (m, 1H), 2.18 – 2.12 (m, 1H), 2.08 (s, 3H) 1.20 (d, *J* = 8 Hz, 3H). **^{13}C NMR (101 MHz, CDCl$_3$)** δ 211.5, 169.5, 139.7, 138.4, 128.5, 128.5, 126.0, 40.9, 39.6, 29.1, 17.4, 16.6. HRMS (ESI) m/z: $[M+H]^+$ calcd. for $C_{14}H_{17}O^+$ 201.1274; found 201.1271.

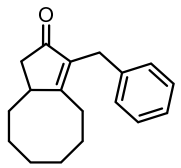

3-benzyl-1,4,5,6,7,8,9,9a-octahydro-2H-cyclopenta[8]annulen-2-one (48)

Prepared following GP3. The crude mixture was purified by flash chromatography on silica (hexane/ethyl acetate 8:2) to afford the product as a yellowish oil (72 mg, 57% after isolation).

^{1}H NMR (400 MHz, $CDCl_3$) δ 7.29 – 7.09 (m, 5H), 3.60 – 3.47 (m, 2H), 2.79 – 2.70 (m, 1H), 2.57 – 2.50 (m, 1H), 2.38 – 2.10 (m, 3H), 1.85 – 1.76 (m, 1H), 1.64 – 1.35 (m, 8H), 1.26 – 1.14 (m, 1H). **^{13}C NMR (101 MHz, $CDCl_3$)** δ 208.4, 180.7, 139.4, 139.4, 128.6, 128.4, 126.0, 42.2, 40.2, 29.3, 29.2, 28.3, 28.1, 26.1, 25.6, 23.8. HRMS (ESI) m/z: $[M+H]^+$ calcd. for $C_{18}H_{23}O^+$ 255.1743; found 255.1743.

2-(6-hydroxy-6-methylheptan-2-yl)-3-methylcyclopent-2-en-1-one (49)

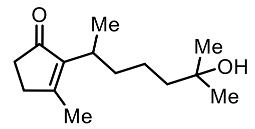

Prepared following GP3. The crude mixture was purified by flash chromatography on silica (cyclohexane/ethyl acetate 8:2) to afford the product as a yellowish oil (98 mg, 82% after isolation).

^{1}H NMR (400 MHz, $CDCl_3$) δ 2.61 (m, 1H), 2.45 (m, 2H), 2.34 – 2.25 (m, 2H), 2.04 (t, *J* = 1.0 Hz, 3H), 1.73 – 1.62 (m, 1H), 1.53 – 1.32 (m, 4H), 1.32 – 1.16 (m, 3H), 1.15 (s, 6H), 1.12 (d, *J* = 7.1 Hz, 3H). **^{13}C NMR (101 MHz, $CDCl_3$)** δ 209.72, 169.91, 143.70, 71.03, 43.91, 34.85, 34.67, 31.84, 30.25, 29.34, 29.32, 22.88, 18.70, 17.66. HRMS (ESI) m/z: $[M+H]^+$ calcd. for $C_{14}H_{24}NaO_2^+$ 247.1669; found 247.1668.

3-methyl-2-(pent-2-en-1-yl)cyclopent-2-en-1-one (50)

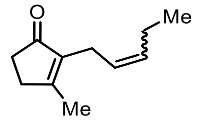

Prepared following GP3. The crude mixture was purified by flash chromatography on silica (hexane/ethyl acetate 8:2) to afford the product as a yellowish oil (64 mg, 78% after isolation, 1.8:1 Z/E stereoisomeric mixture). The Z/E stereochemistry of product **50** has been assigned through ^{1}H-NOESY experiment.

^{1}H NMR (400 MHz, $CDCl_3$) δ 5.45 – 5.17 (m, 4H), 2.90 (d, *J* = 7 Hz, 2H, isomer *Z*, major), 2.84 (d, *J* = 6 Hz, 2H, isomer *E*, minor), 2.48 – 2.45 (m, 4H), 2.35 – 2.32 (m, 4H), 2.16 – 2.08 (m, 2H, isomer *Z*, major), 2.03 (d, *J* = 3 Hz, 6H), 1.97 – 1.90 (m, 2H, isomer *E*, minor), 0.95 (t, *J* = 7 Hz, 3H, isomer *Z*, major), 0.90 (t, *J* = 7 Hz, 3H, isomer *E*, minor). **^{13}C NMR (101 MHz, isomer *Z*, major from the mixture, $CDCl_3$)** δ 209.0, 170.4, 139.5, 132.4, 125.1, 34.3, 31.7, 26.1, 21.2, 17.3, 14.2. **^{13}C NMR (101 MHz, isomer *E*, minor from the mixture, $CDCl_3$)** δ 209.1, 170.9, 139.0, 133.0, 125.0, 34.3, 31.7, 25.4, 20.6, 17.3, 13.7. Characterization data of **(*Z*)-50** are in accordance with the literature.[27] HRMS (ESI) m/z: $[M+H]^+$ calcd. for $C_{11}H_{17}O^+$ 165.1247; found 165.1246.

8. Additional comments on the Hunsdiecker condensation (HC)

The cyclization of 1,4-diketones to afford cyclopentenones, *i.e.* the Hunsdiecker Condensation - HC - ,[28-29] is a kinetically controlled reaction,[30] which follows a (disfavoured, yet the only viable) 5-(enolendo)-exo-trig cyclization mode,[31] according to the classification offered by the Baldwin rules.[32]

For unsymmetrical diketones, there are multiple non-equivalent deprotonation sites α-to the carbonyl groups present, potentially leading to different cyclization pathways. Only some of the corresponding enolates, however, are productive for the formation of the desired cyclopentenone derivatives, as reported in Scheme S1. In particular, only the deprotonation of methylene (CH_2) or methyl (CH_3) groups can lead to a successful HC, while deprotonation at a methine (CH) one cannot, in accordance with the general textbook rules for aldol condensations (in the latter case the dehydration step is not possible). From the experimental point of view, in the present work we observe that the deprotonation of a methylene group (CH_2) selectively leads to the cyclopentenone derivative of interest, even if the deprotonation of a methyl group (CH_3) competes. However, the latter pathway, namely the deprotonation of a terminal methyl group (CH_3) becomes viable and leads selectively to the corresponding cyclopentenone derivative, event in the presence of a methine (CH) group next to the carbonyl.

Scheme S1. Possible cyclization pathways of 1,4-diketones

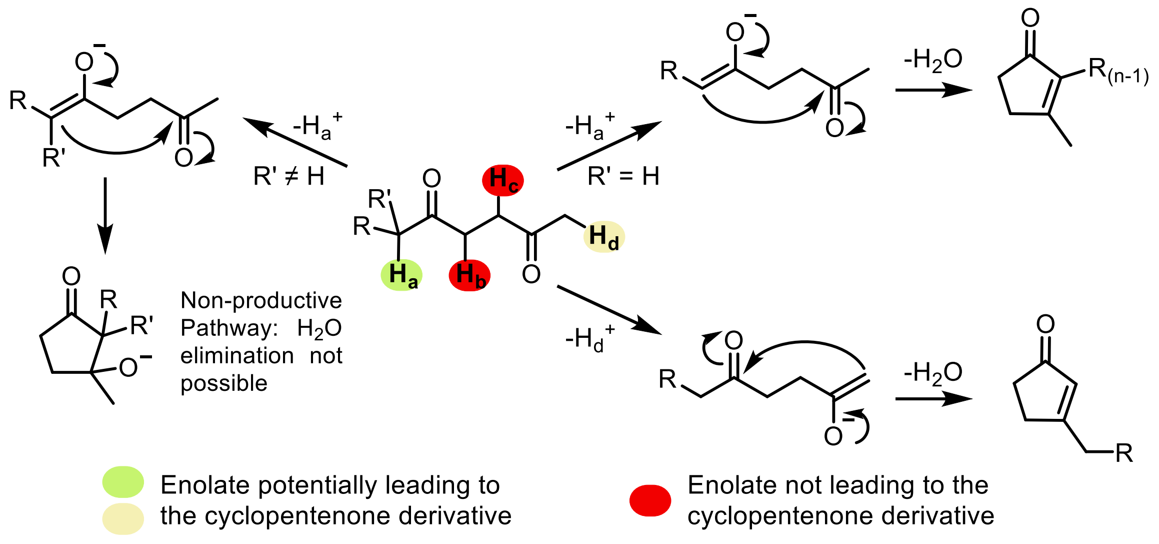

9. References

[1] S. Protti, D. Ravelli, M. Fagnoni, A. Albini, *Chem. Commun.* **2009**, Dec 2110.1039/b917732a, 7351-7353.

[2] O. Lifchits, M. Mahlau, C. M. Reisinger, A. Lee, C. Farès, I. Polyak, G. Gopakumar, W. Thiel, B. List, *J. Am. Chem. Soc.* **2013**, *135*, 6677-6693.

[3] M. Dieckmann, M. Kretschmer, P. Li, S. Rudolph, D. Herkommer, D. Menche, *Angew. Chem. Int. Ed.* **2012**, *51*, 5667-5670.

[4] H. Yu, B. Yu, H. Zhang, H. Huang, *Org. Lett.* **2021**, *23*, 3891-3896.

[5] S. Heindl, M. Riomet, J. Matyasovsky, M. Lemmerer, N. Malzer, N. Maulide, *Angew. Chem. Int. Ed.* **2021**, *60*, 19123-19127.

[6] S. Castellano, C. Milite, A. Feoli, M. Viviano, A. Mai, E. Novellino, A. Tosco, G. Sbardella, *ChemMedChem* **2015**, *10*, 144-157.

[7] A. Bugarin, K. D. Jones, B. T. Connell, *Chem. Commun.* **2010**, *46*, 1715-1717.

[8] P. Szcześniak, S. Buda, L. Lefevre, O. Staszewska-Krajewska, J. Mlynarski, *Eur. J. Org. Chem.* **2019**, *2019*, 6973-6982.

[9] H. Nakahira, I. Ryu, M. Ikebe, Y. Oku, A. Ogawa, N. Kambe, N. Sonoda, S. Murai, *J. Org. Chem.* **1992**, *57*, 17-28.

[10] T. M. Masson, S. D. A. Zondag, J. H. A. Schuurmans, T. Noël, *React. Chem. Eng.* **2024**, *9*, 2218-2225.

[11] S. Esposti, D. Dondi, M. Fagnoni, A. Albini, *Angew. Chem. Int. Ed.* **2007**, *46*, 2531-2534.

[12] L. Akelis, J. Rousseau, R. Juskenas, J. Dodonova, C. Rousseau, S. Menuel, D. Prevost, S. Tumkevičius, E. Monflier, F. Hapiot, *Eur. J. Org. Chem.* **2016**, *2016*, 31-35.

[13] J. Yao, Y. Xiao, H. Li, X. Yang, J. Du, Y. Yin, L. Feng, W. Duan, L. Yu, *Org. Lett.* **2024**, *26*, 7307-7312.

[14] G. A. Molander, S. L. J. Trice, S. M. Kennedy, *J. Org. Chem.* **2012**, *77*, 8678-8688.

[15] A. Ohno, T. Sato, T. Mase, Y. Uozumi, Y. M. A. Yamada, *Adv. Synth. Catal.* **2020**, *362*, 4687-4698.

[16] V. J. Geiger, G. Lefèvre, I. Fleischer, *Chem. Eur. J.* **2022**, *28*, e202202212.

[17] J. Mathew, *J. Org. Chem.* **1991**, *56*, 713-716.

[18] E. Piers, J. Banville, C. K. Lau, I. Nagakura, *Can. J. Chem.* **1982**, *60*, 2965-2975.

[19] P. Verma, R. R. Pallerla, A. Rolig, P. M. Pihko, *J. Org. Chem.* **2024**, *89*, 6987-6990.

[20] J. Lefarth, A. G. Griesbeck, *J. Org. Chem.* **2022**, *87*, 8028-8033.

[21] D. Aynetdinova, R. Jacques, K. E. Christensen, T. J. Donohoe, *Chem. Eur. J.* **2023**, *29*, e202203732.

[22] Q. Lang, H. Yang, G. Gu, Q. Feng, J. Wen, X. Zhang, *Chin. J. Chem .* **2021**, *39*, 933-936.

[23] S. Chiba, Y.-J. Xu, Y.-F. Wang, *J. Am. Chem. Soc.* **2009**, *131*, 12886-12887.

[24] K. Antczak, J. F. Kingston, S. J. Alward, A. G. Fallis, *Can. J. Chem.* **1984**, *62*, 829-837.

[25] Y. Jang, V. N. G. Lindsay, *Org. Lett.* **2020**, *22*, 8872-8876.

[26] R. C. Larock, E. K. Yum, H. Yang, *Tetrahedron* **1994**, *50*, 305-321.

[27] Y. Nakahara, M. Matsui, *Agricultural and Biological Chemistry* **1975**, *39*, 1887-1888.

[28] H. Hunsdiecker, *Ber. Dtsch. Chem. Ges.* **1942**, *75*, 447-454.

[29] Z. Wang, *Hunsdiecker Condensation,* in *Comprehensive Organic Name Reactions and Reagents*, 10.1002/9780470638859.conrr337, John Wiley & Sons, Inc., **2010**, pp. 1508-1510.

[30] P. M. McCurry, Jr., R. K. Singh, *J. Org. Chem.* **1974**, *39*, 2316-2317.

[31] J. E. Baldwin, M. J. Lusch, *Tetrahedron* **1982**, *38*, 2939-2947.

[32] K. Gilmore, R. K. Mohamed, I. V. Alabugin, *WIREs Computational Molecular Science* **2016**, *6*, 487-514.

10. Copy of NMR spectra

10.1 Starting Materials

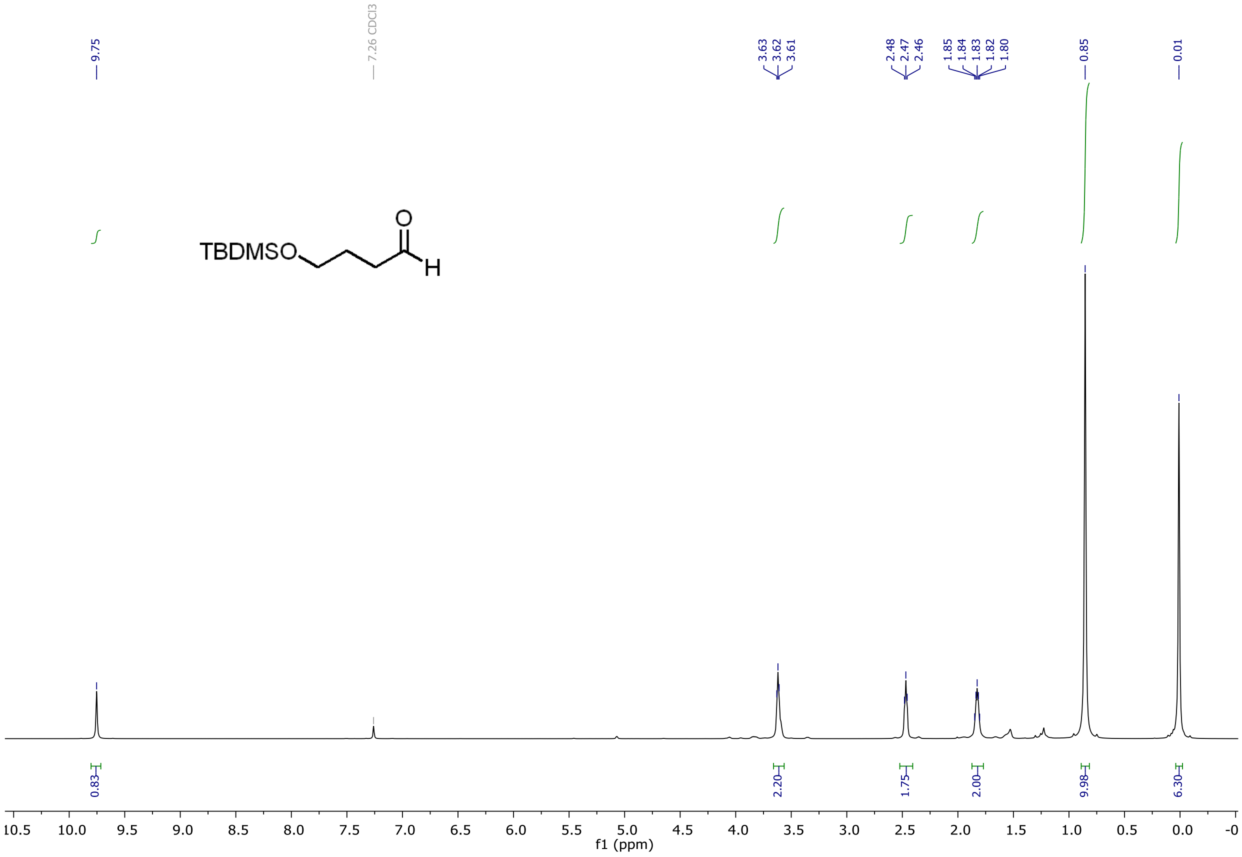

^{1}**H NMR (600 MHz, CDCl$_3$)** of ***4-((tert-butyldimethylsilyl)oxy)butanal (S1e)***

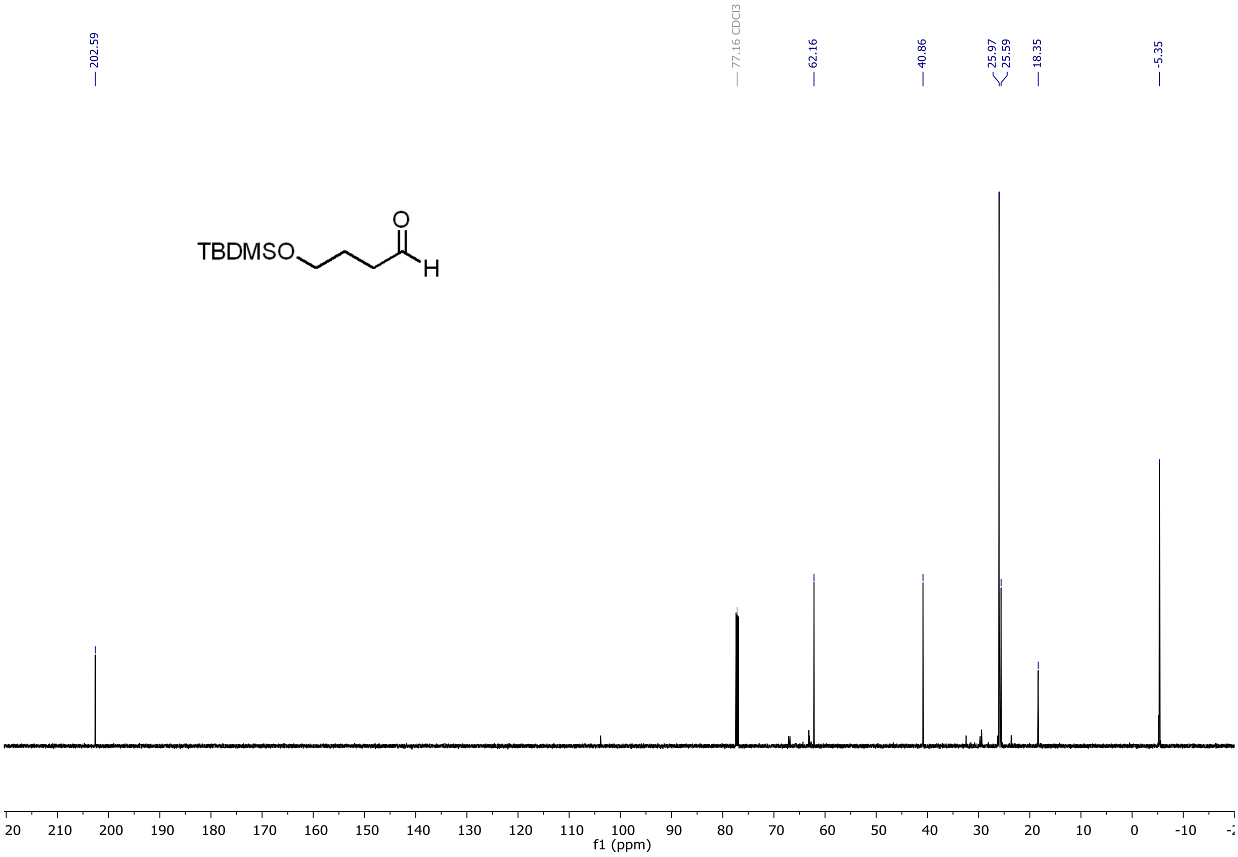

^{13}**C NMR (151 MHz, CDCl$_3$)** of ***4-((tert-butyldimethylsilyl)oxy)butanal (S1e)***

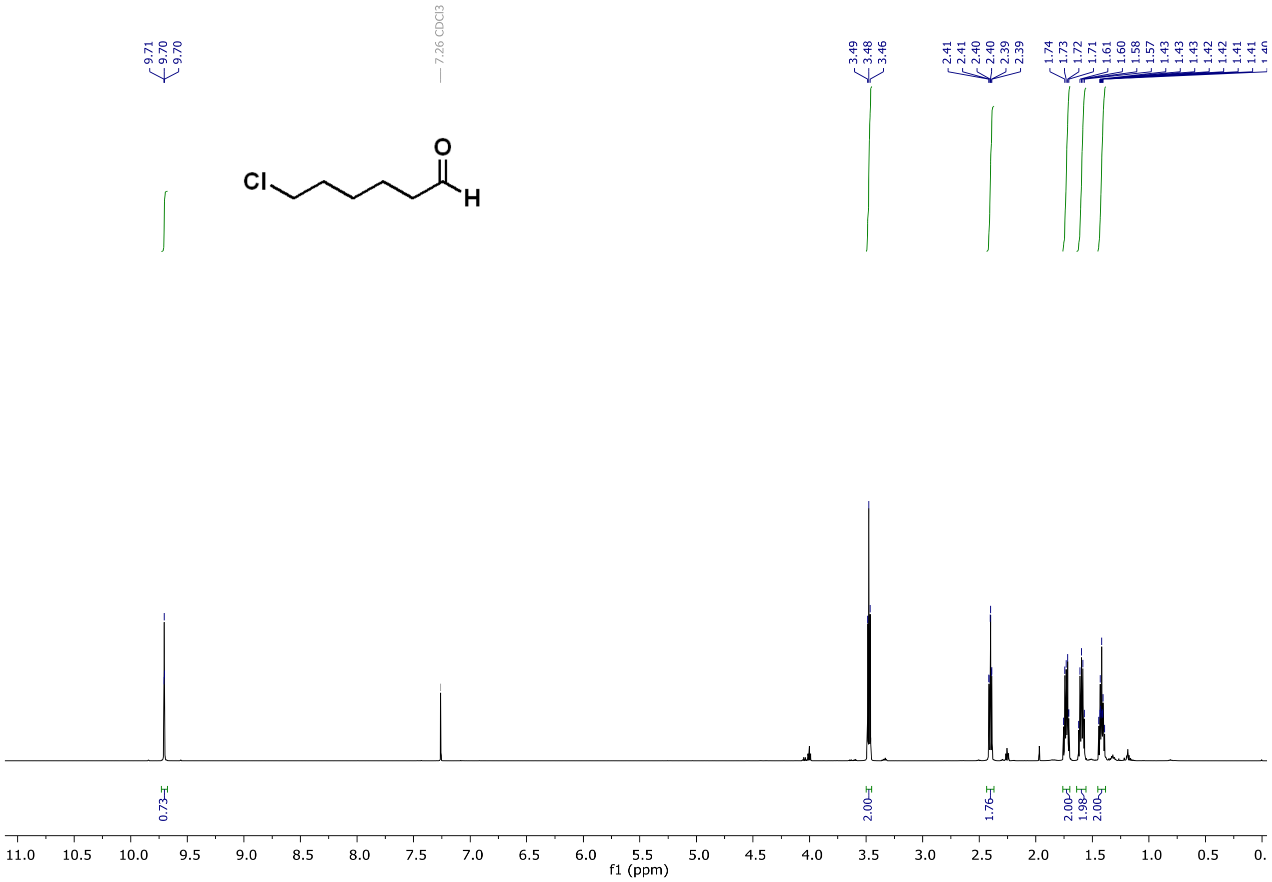

^{1}H NMR (600 MHz, $CDCl_3$) of *6-chlorohexanal (S1f)*

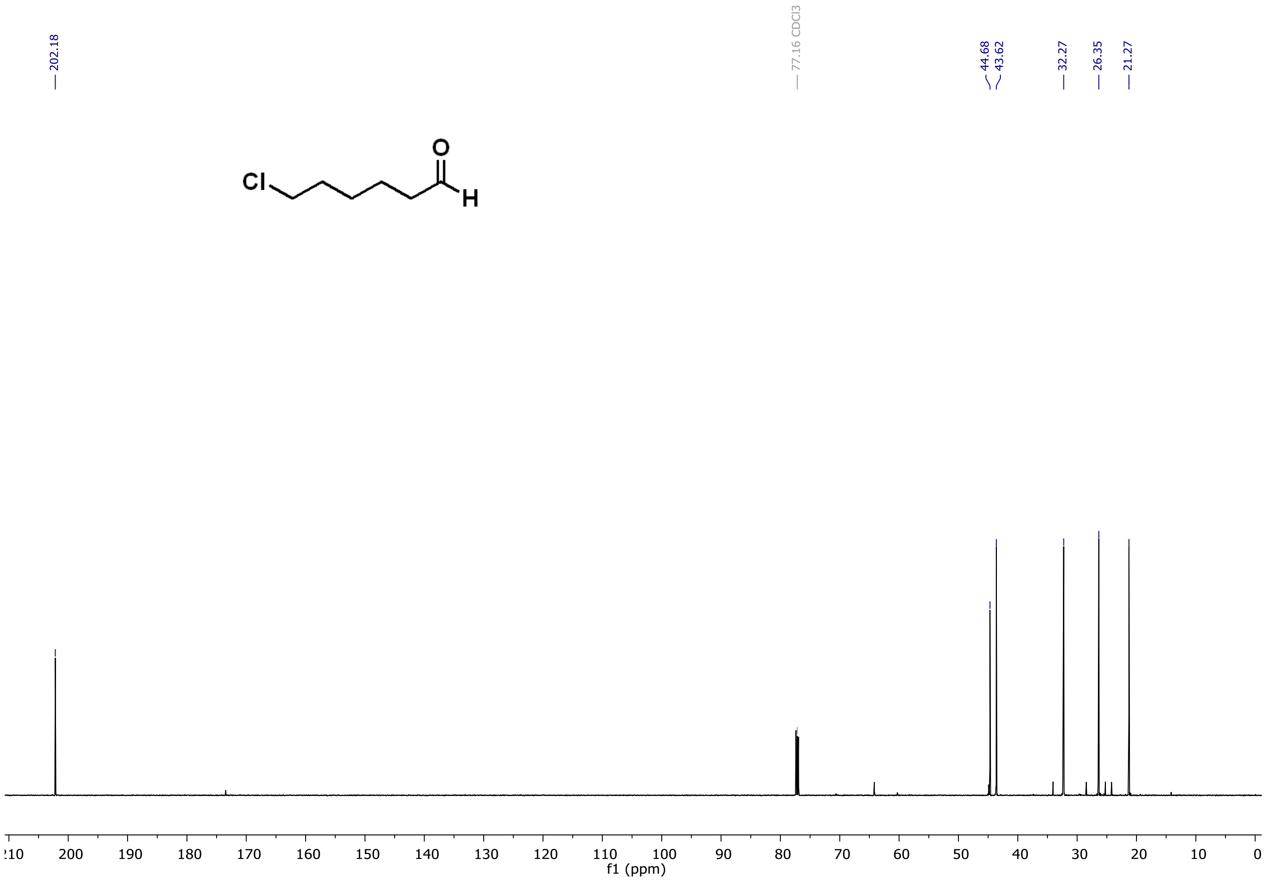

^{13}C NMR (151 MHz, $CDCl_3$) of *6-chlorohexanal (S1f)*

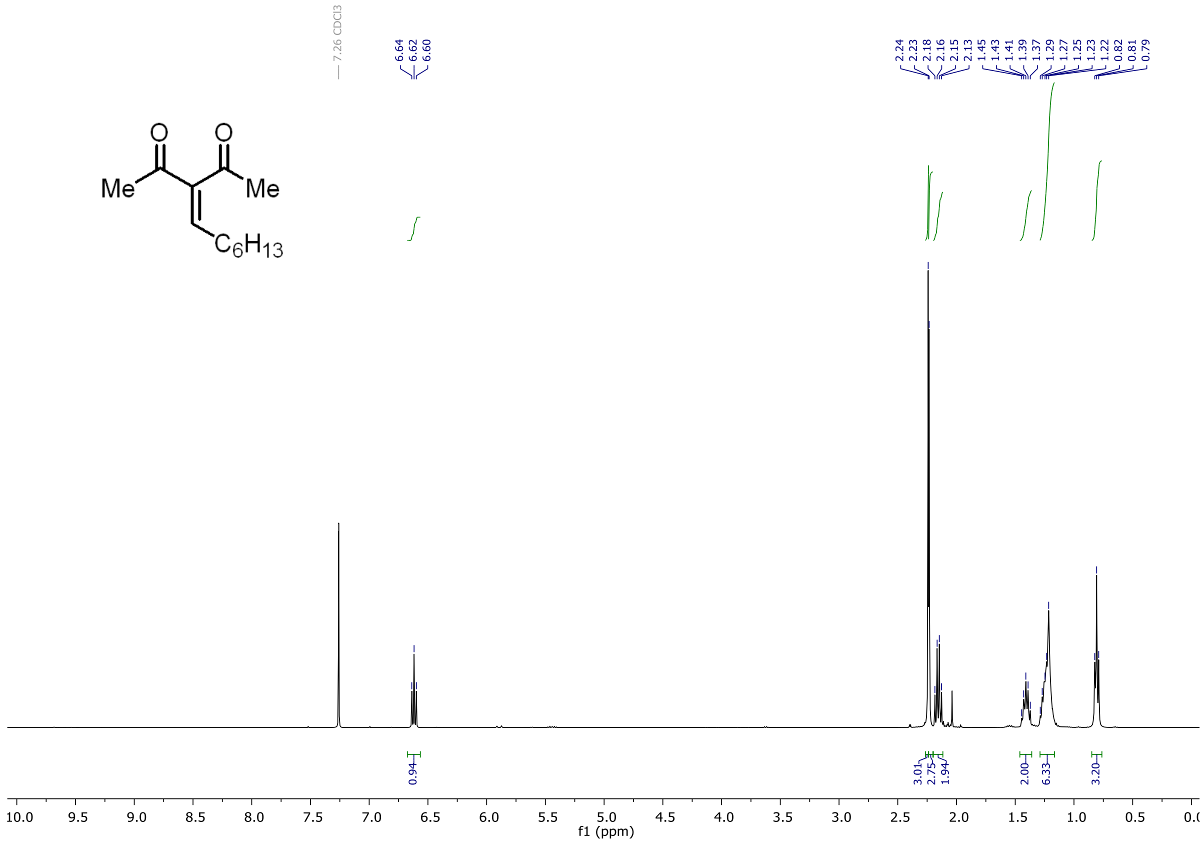

^1H NMR (400 MHz, $CDCl_3$) of ***3-heptylidenepentane-2,4-dione (S2c)***

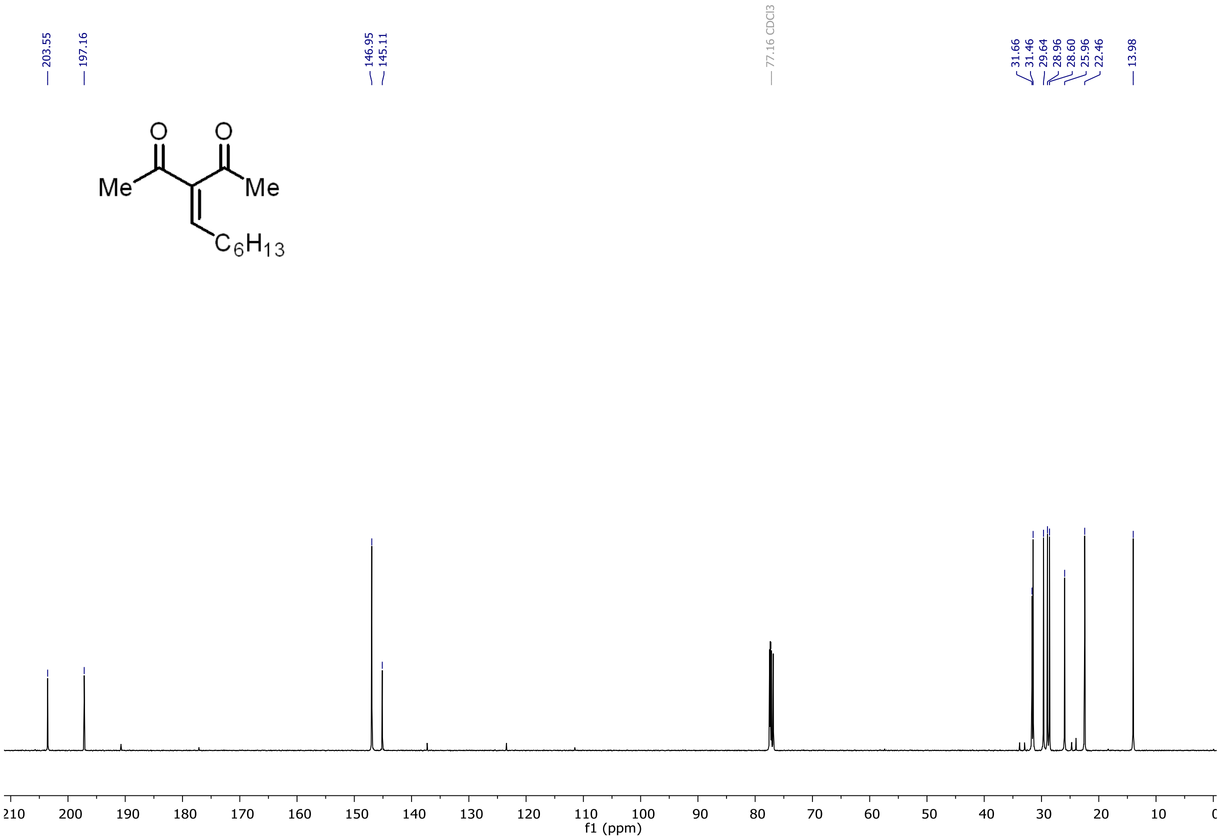

^{13}C NMR (101 MHz, $CDCl_3$) of ***3-heptylidenepentane-2,4-dione (S2c)***

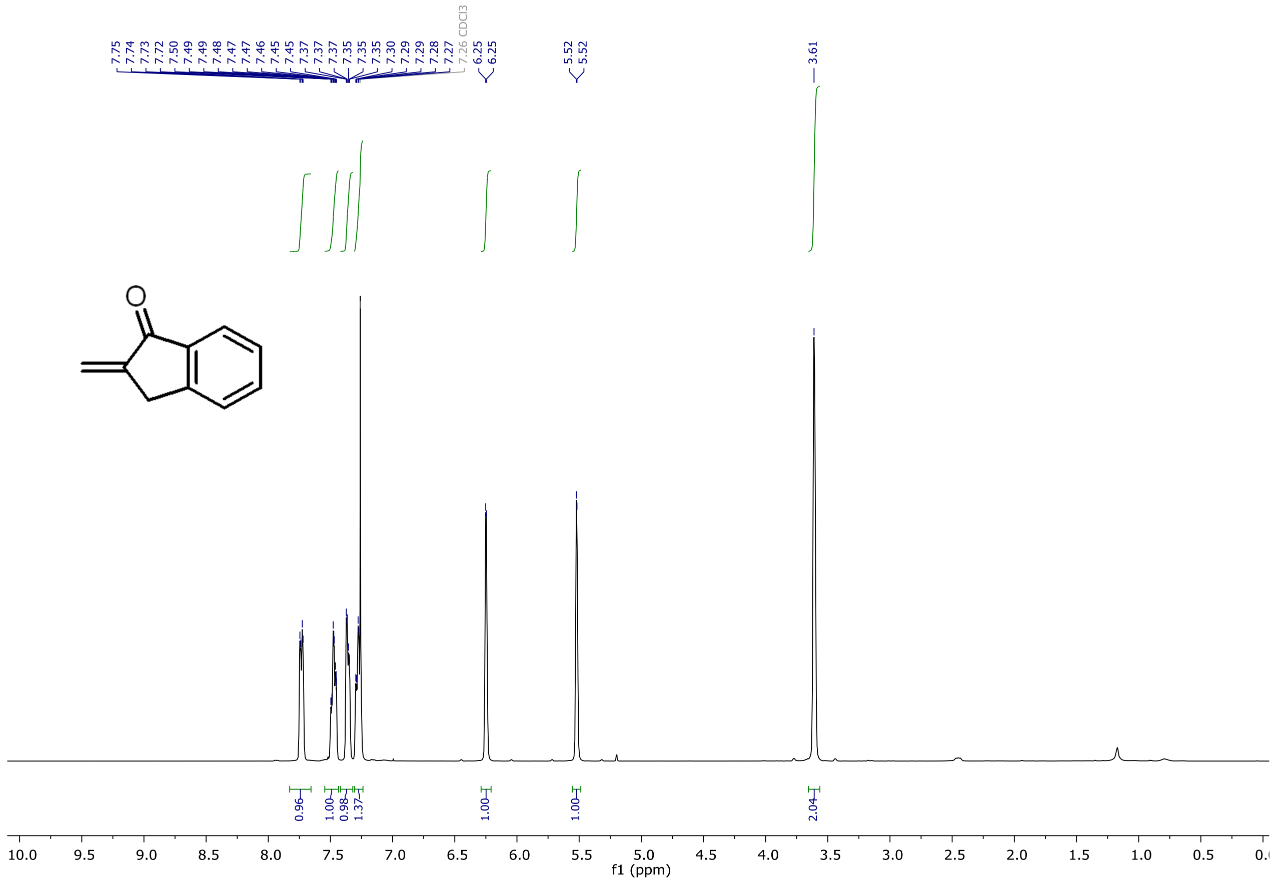

^{1}H NMR (400 MHz, CDCl$_3$) of ***2-methylene-2,3-dihydro-1H-inden-1-one (S2e)***

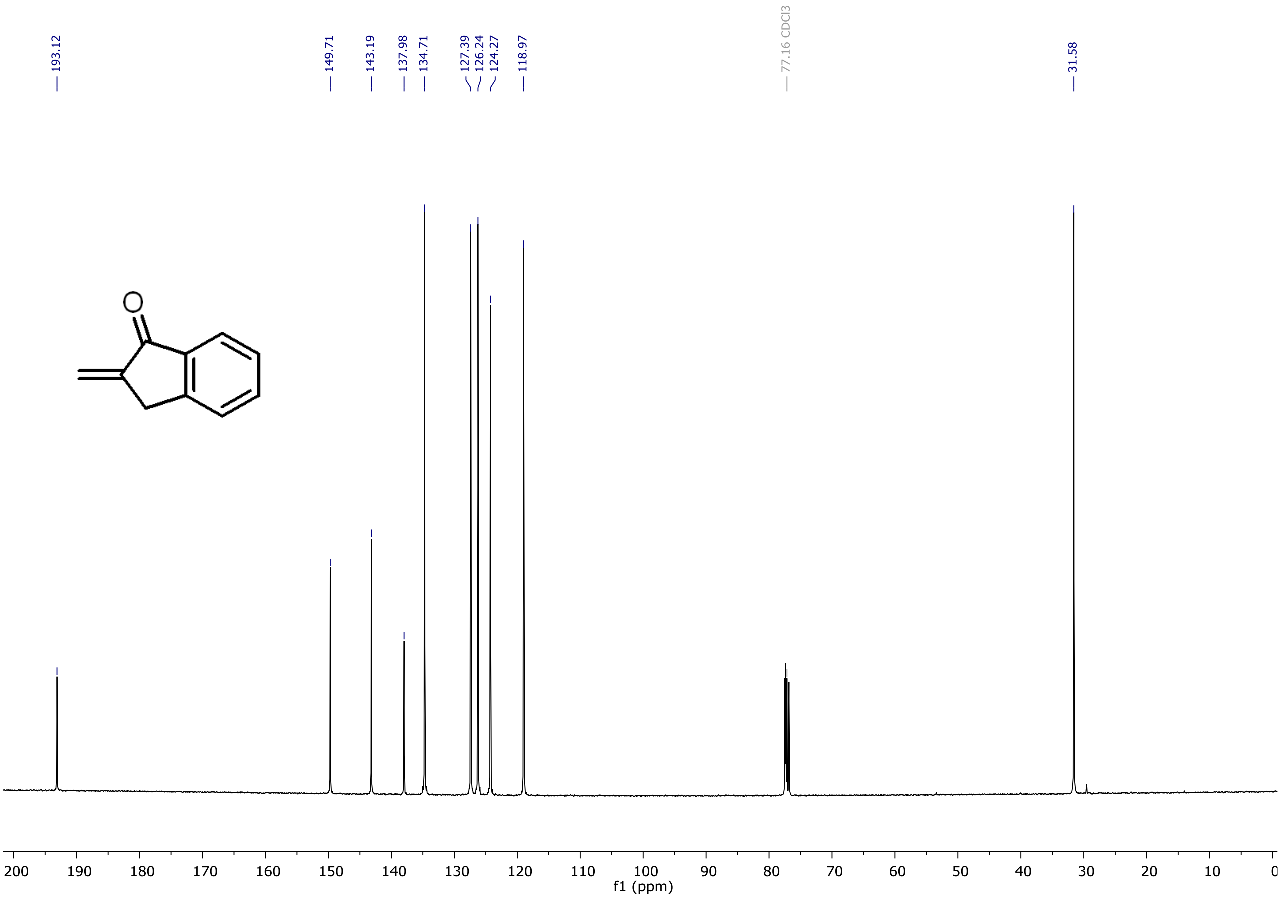

^{13}C NMR (101 MHz, CDCl$_3$) of ***2-methylene-2,3-dihydro-1H-inden-1-one (S2e)***

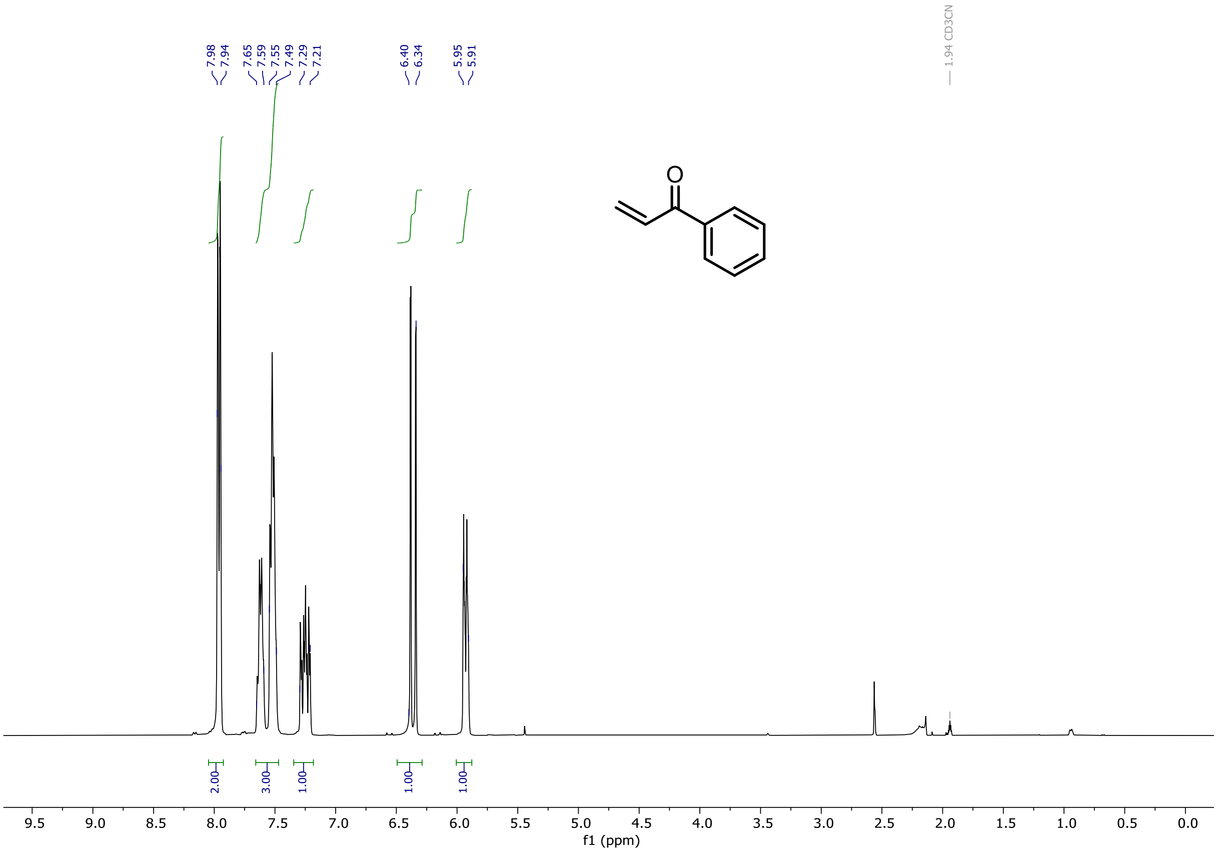

^{1}H NMR (400 MHz, CDCl$_3$) of ***1-phenylprop-2-en-1-one (S2g)***

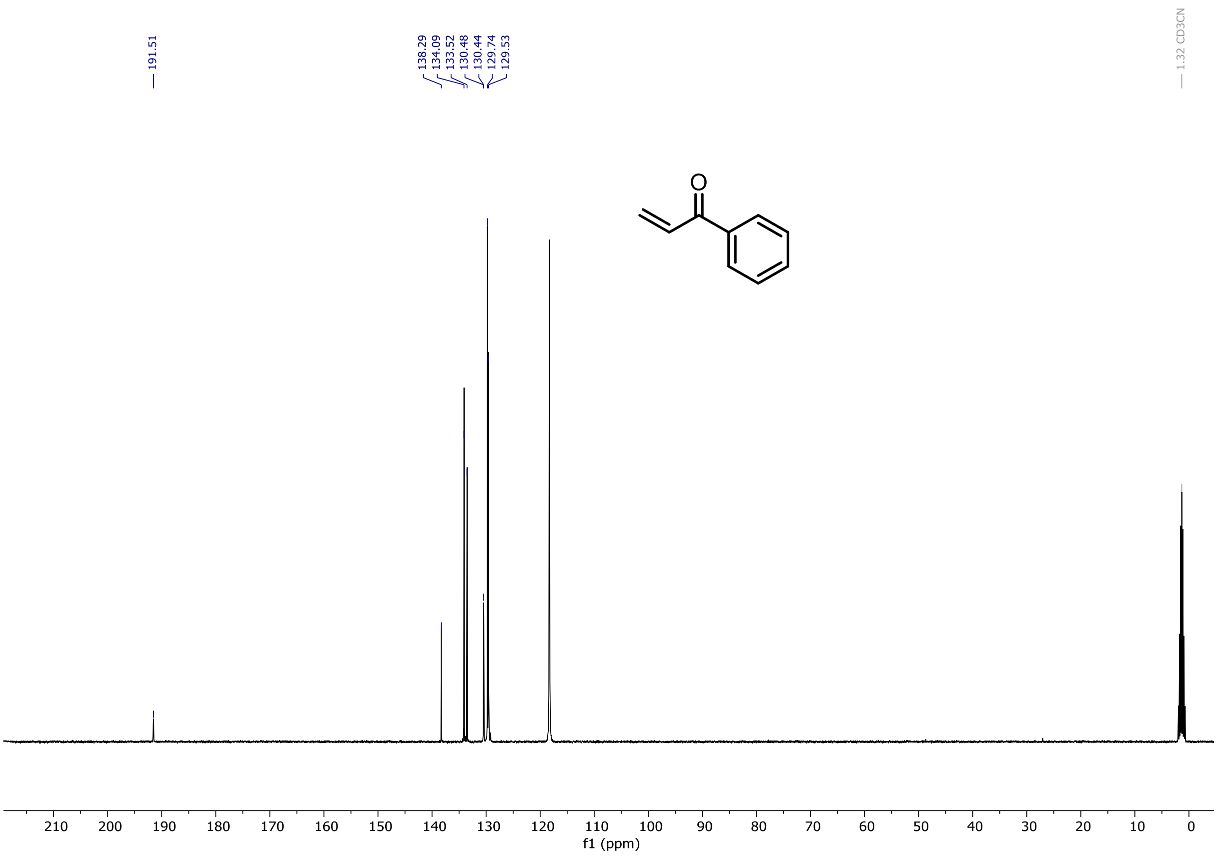

^{13}C NMR (101 MHz, CDCl$_3$) of ***1-phenylprop-2-en-1-one (S2g)***

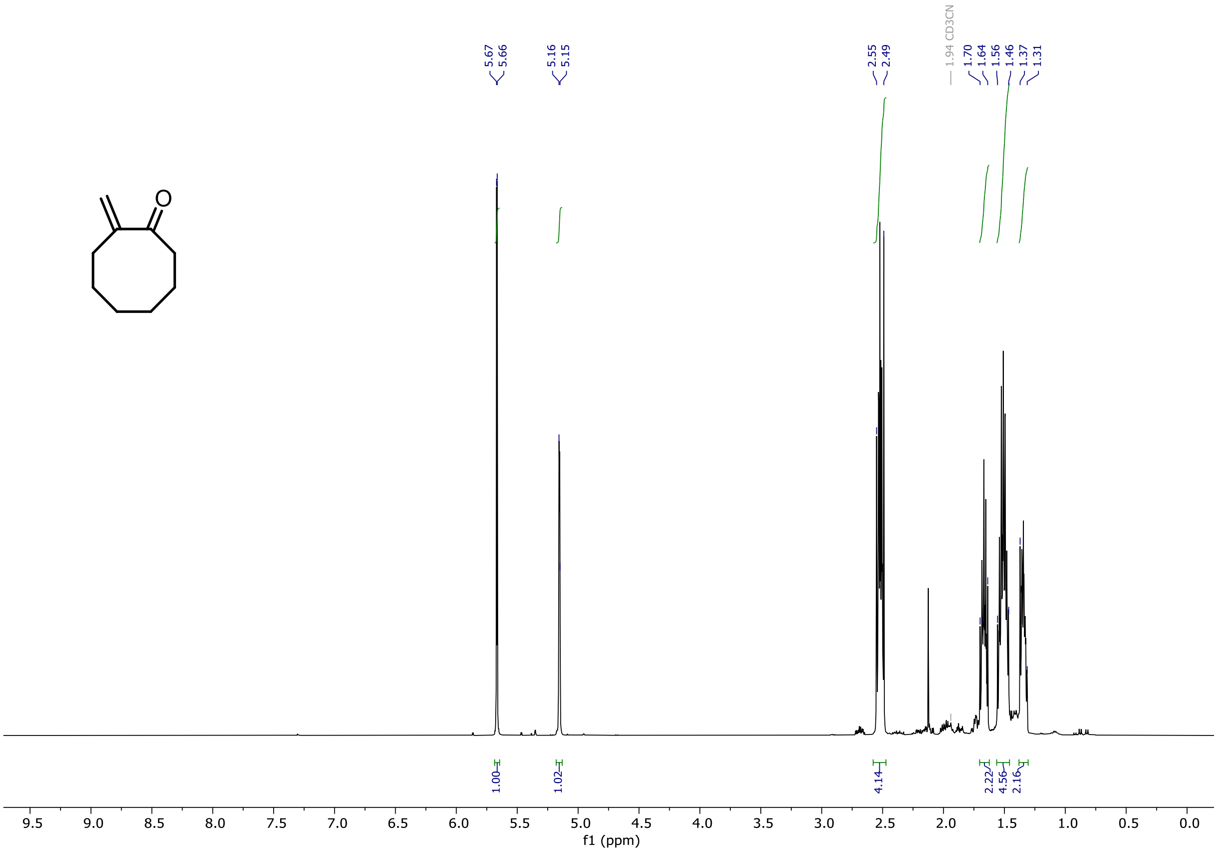

^{1}H NMR (400 MHz, $CDCl_3$) of ***2-methylenecyclooctan-1-one (S2j)***

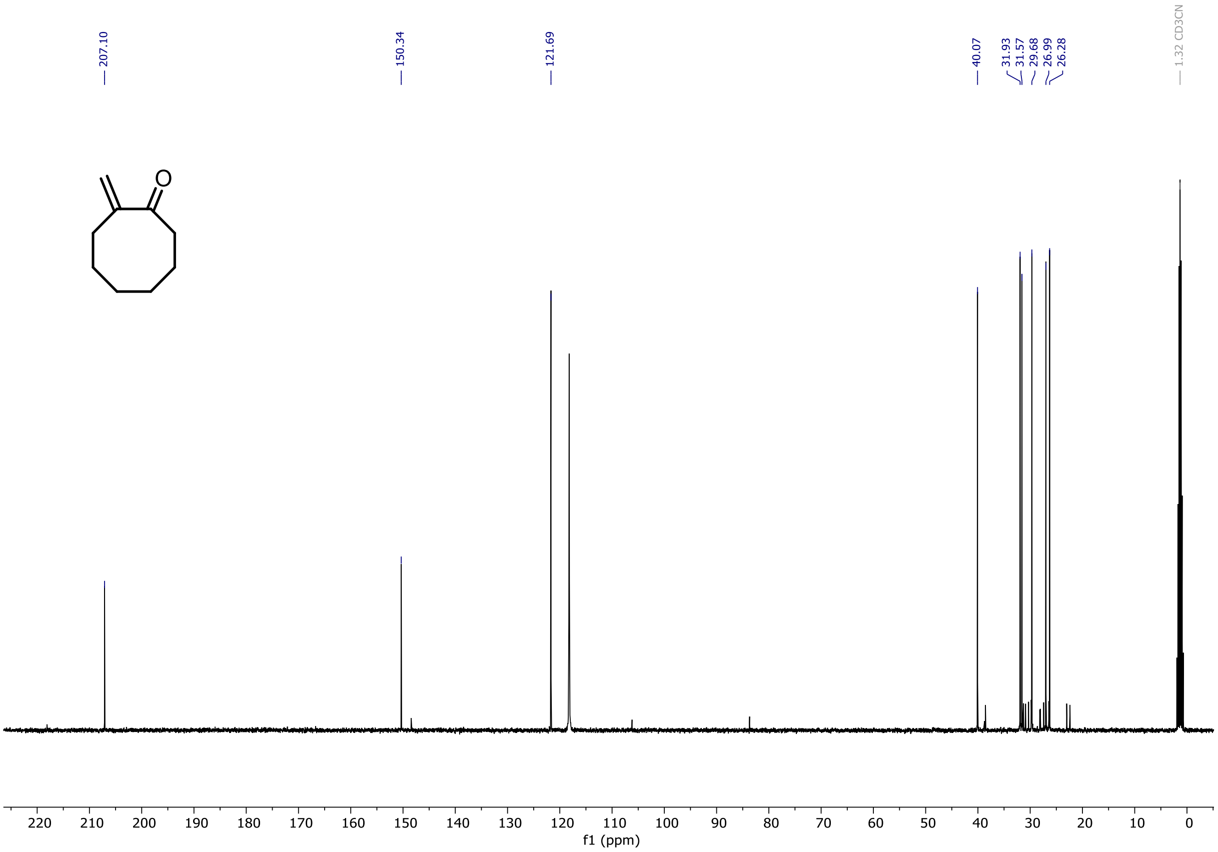

^{13}C NMR (101 MHz, $CDCl_3$) of ***2-methylenecyclooctan-1-one (S2j)***

10.2 Pyrroles

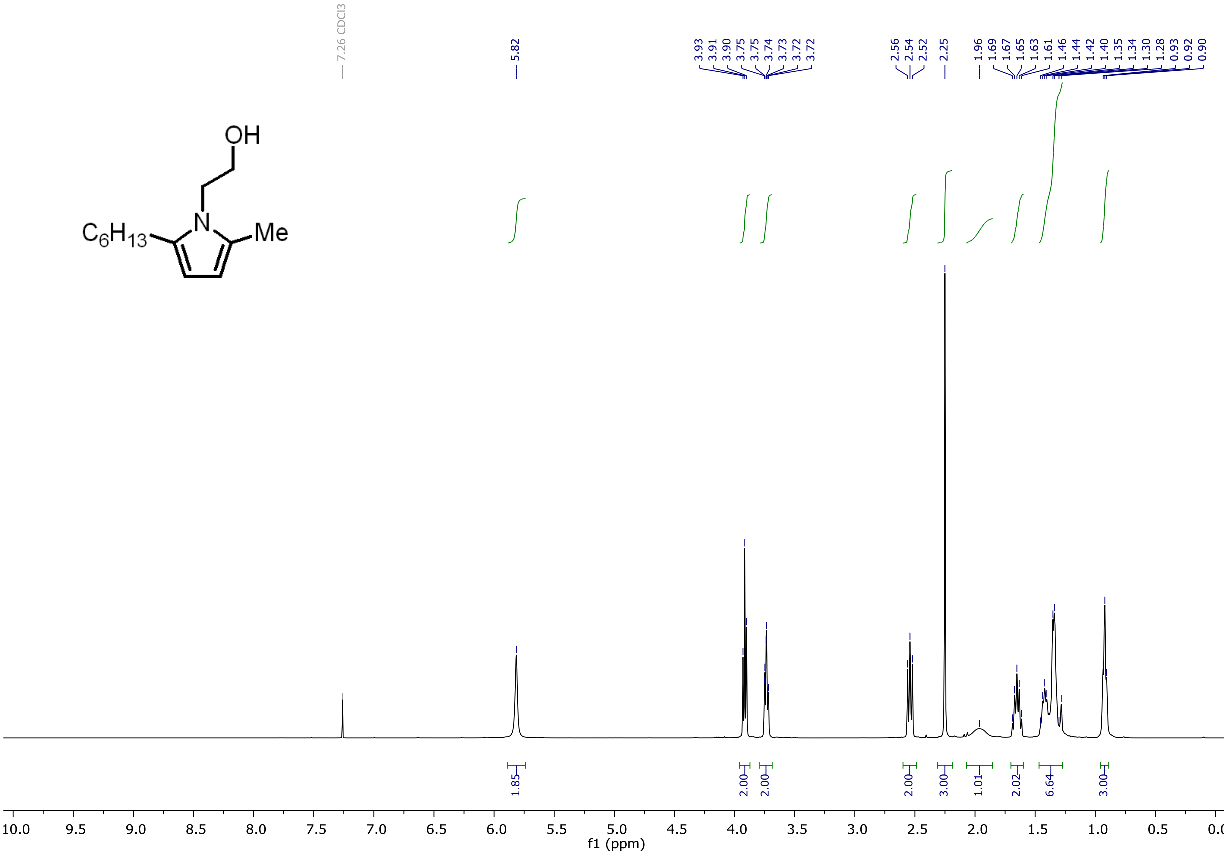

^{1}H NMR (400 MHz, $CDCl_3$) of ***2-(2-hexyl-5-methyl-1H-pyrrol-1-yl)ethan-1-ol (1)***

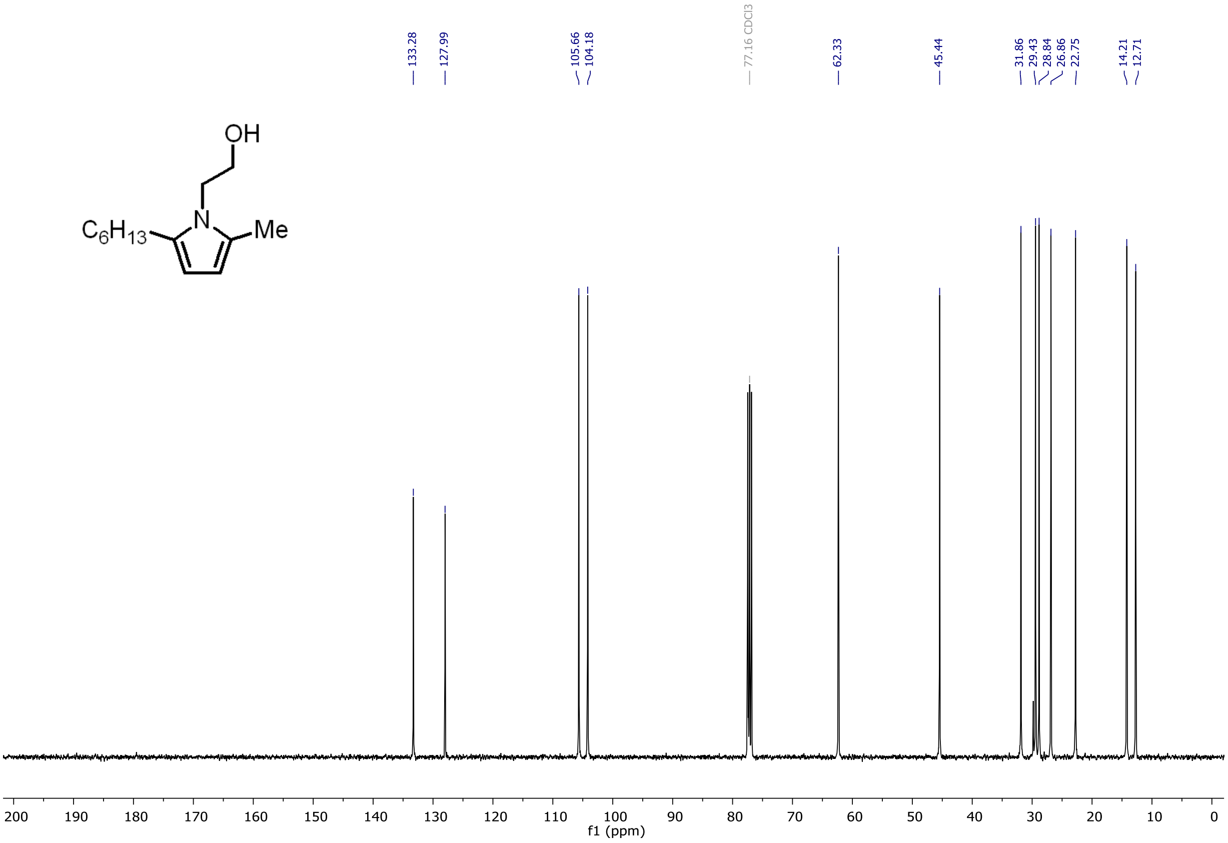

^{13}C NMR (101 MHz, $CDCl_3$) of ***2-(2-hexyl-5-methyl-1H-pyrrol-1-yl)ethan-1-ol (1)***

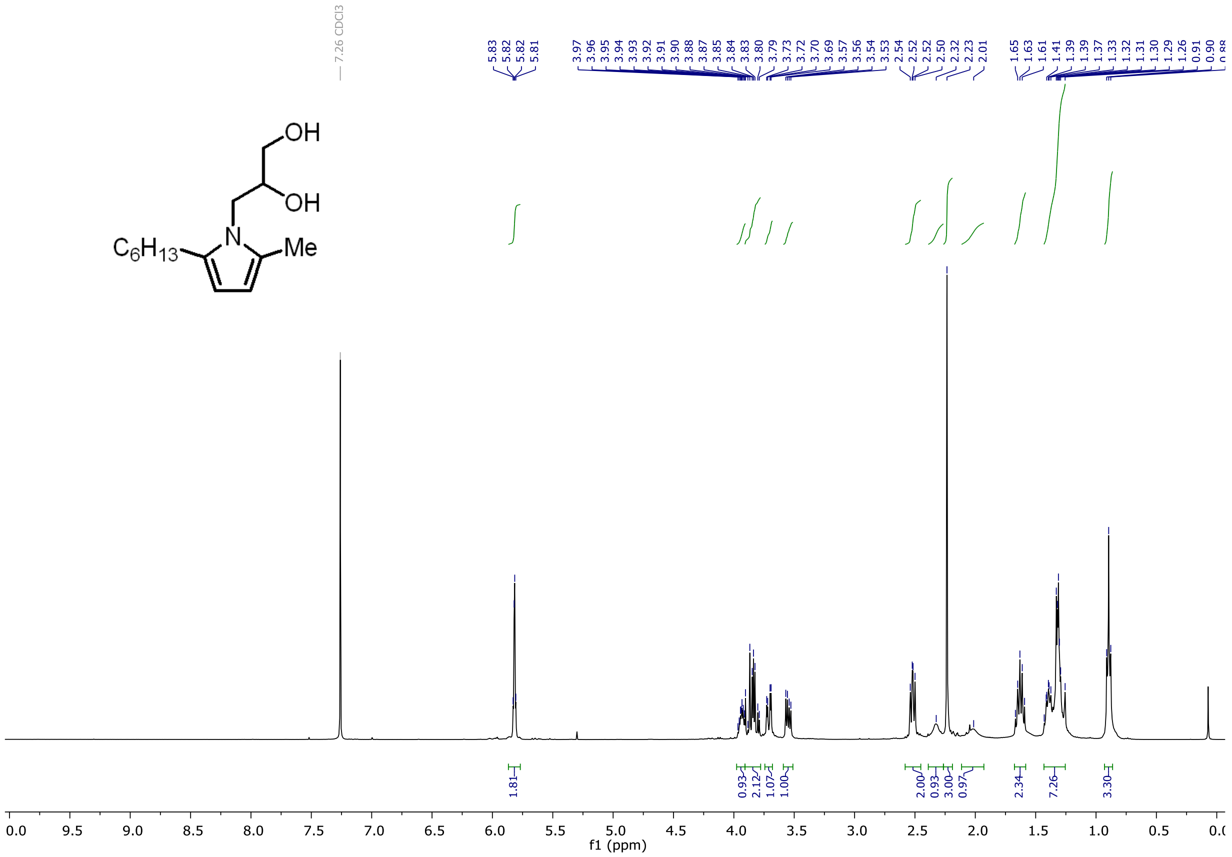

^{1}H NMR (400 MHz, CDCl$_3$) of ***3-(2-hexyl-5-methyl-1H-pyrrol-1-yl)propane-1,2-diol (2)***

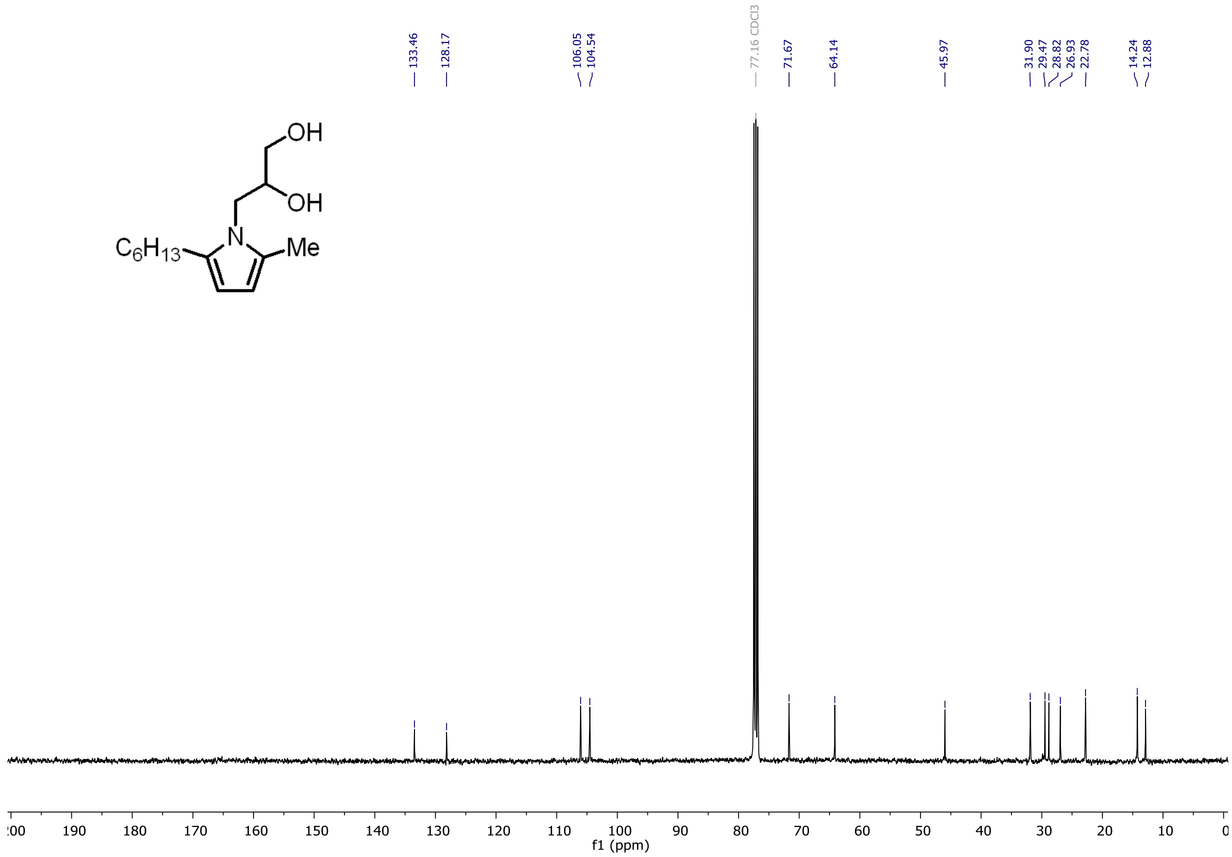

^{13}C NMR (101 MHz, CDCl$_3$) of ***3-(2-hexyl-5-methyl-1H-pyrrol-1-yl)propane-1,2-diol (2)***

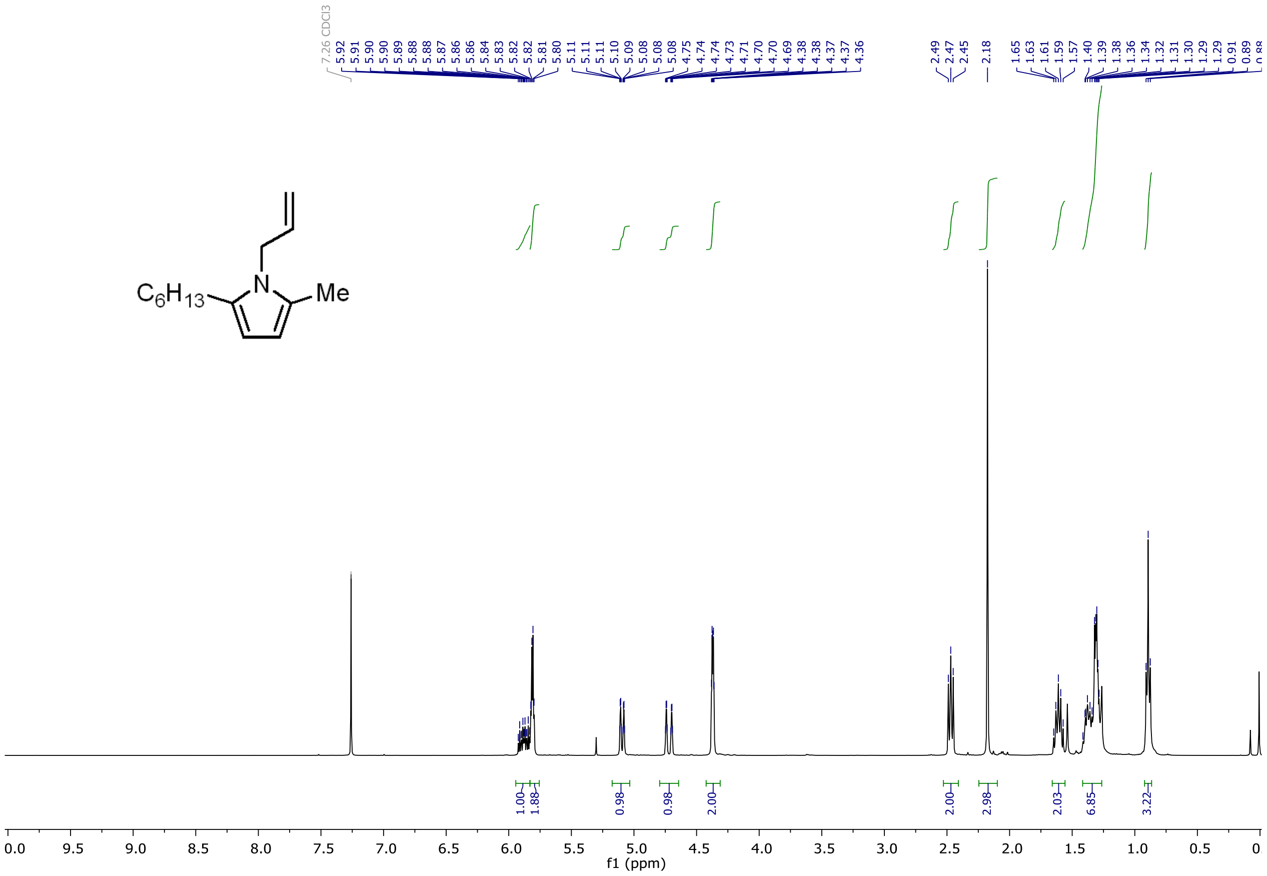

^{1}H NMR (400 MHz, $CDCl_3$) of ***1-allyl-2-hexyl-5-methyl-1H-pyrrole (3)***

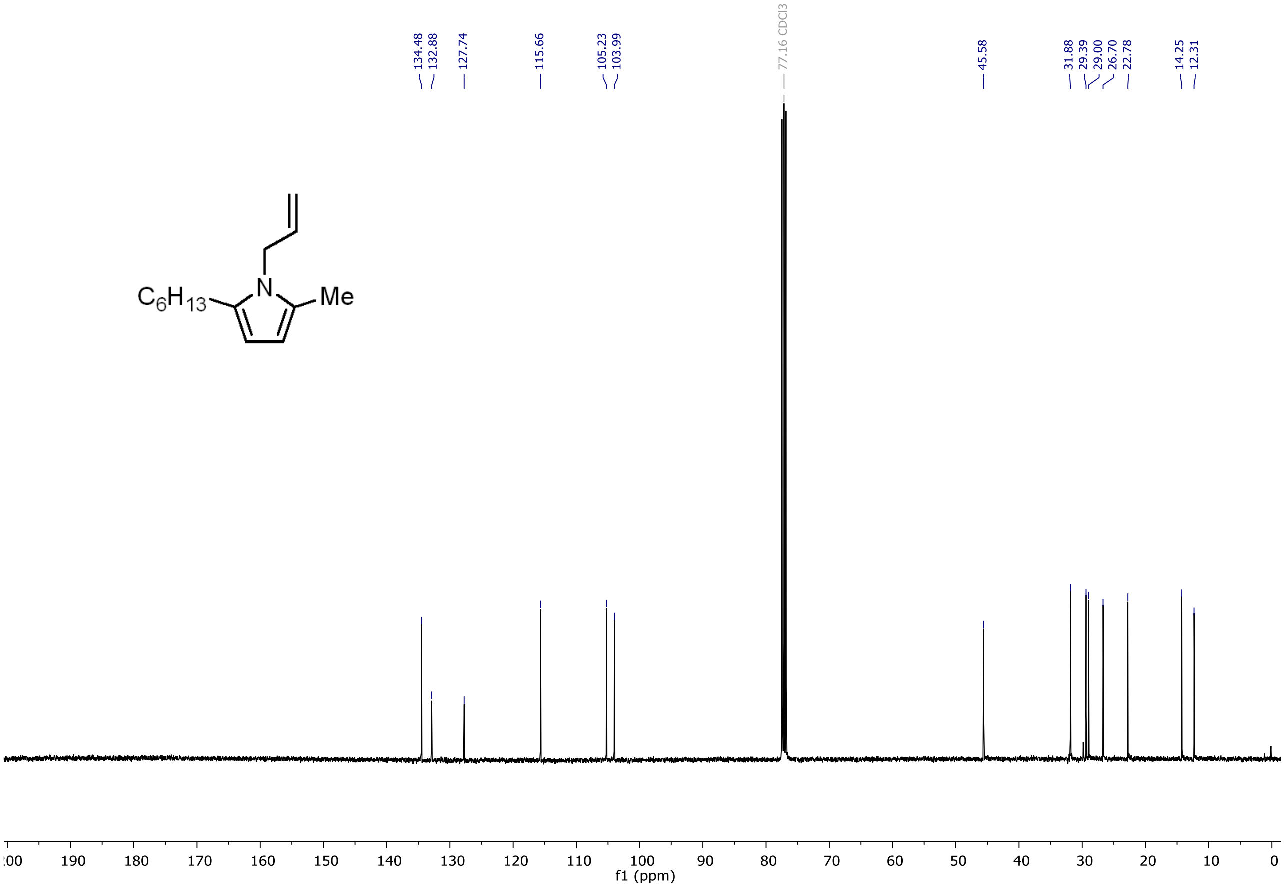

^{13}C NMR (101 MHz, $CDCl_3$) of ***1-allyl-2-hexyl-5-methyl-1H-pyrrole (3)***

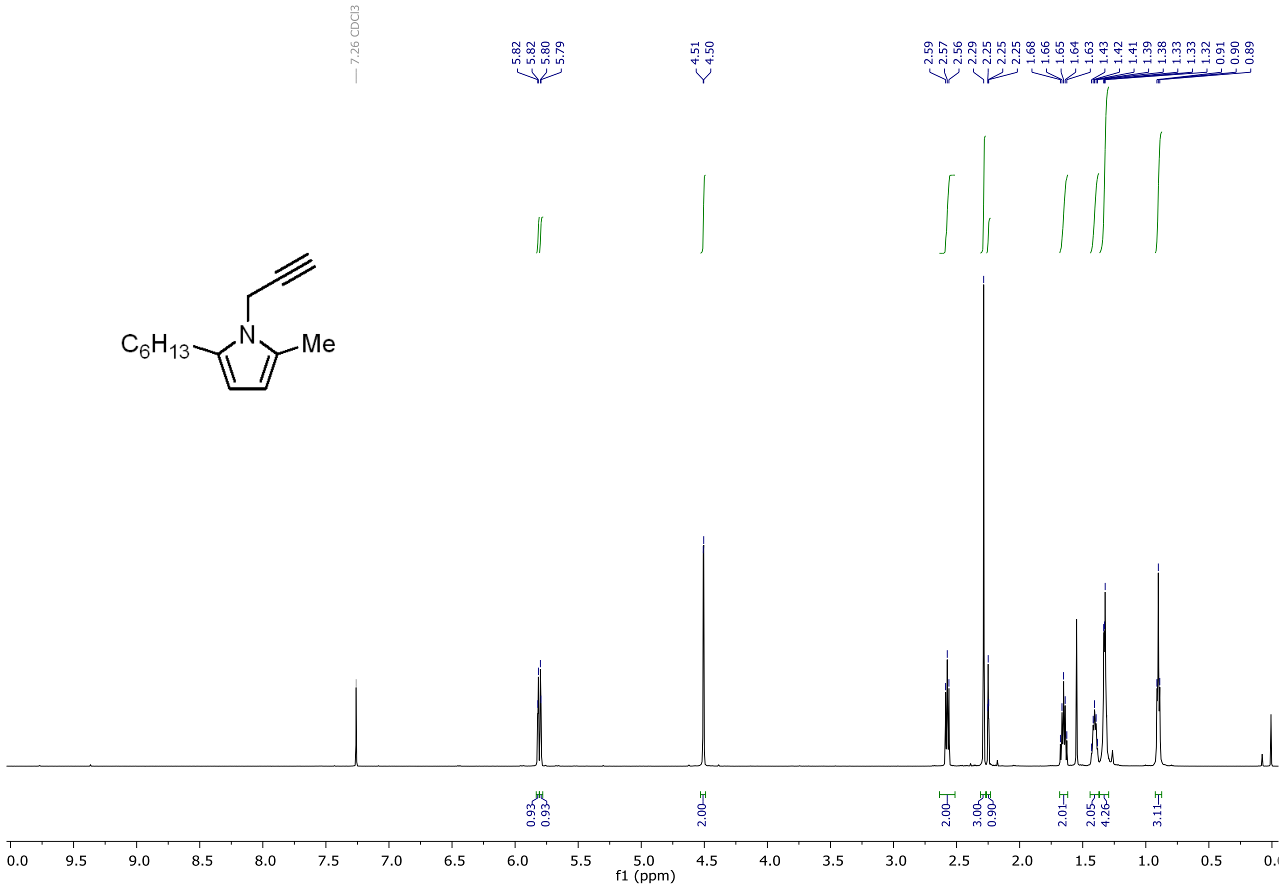

^{1}**H NMR (600 MHz, CDCl$_3$)** of **2-hexyl-5-methyl-1-(prop-2-yn-1-yl)-1H-pyrrole** ***(4)***

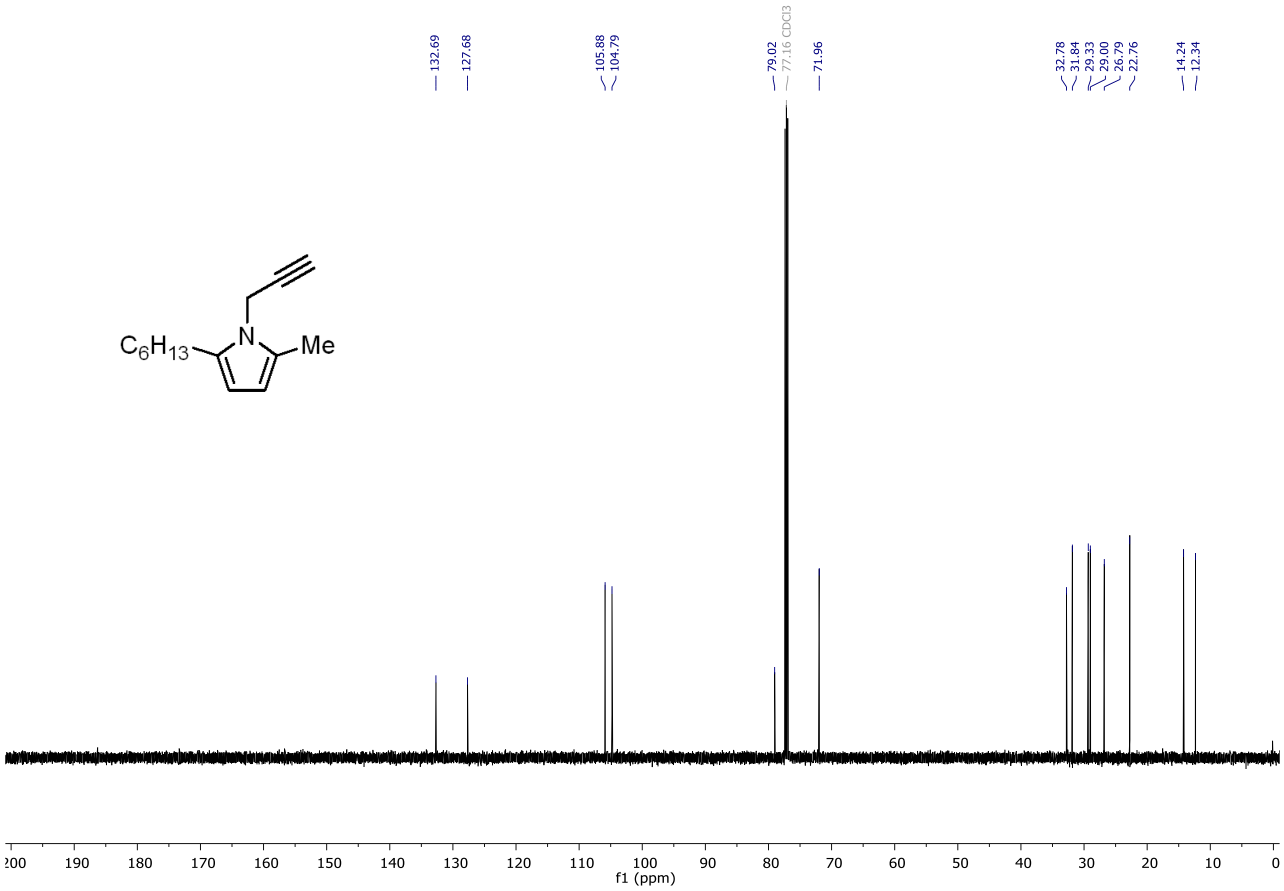

^{13}**C NMR (151 MHz, CDCl$_3$)** of ***2-hexyl-5-methyl-1-(prop-2-yn-1-yl)-1H-pyrrole (4)***

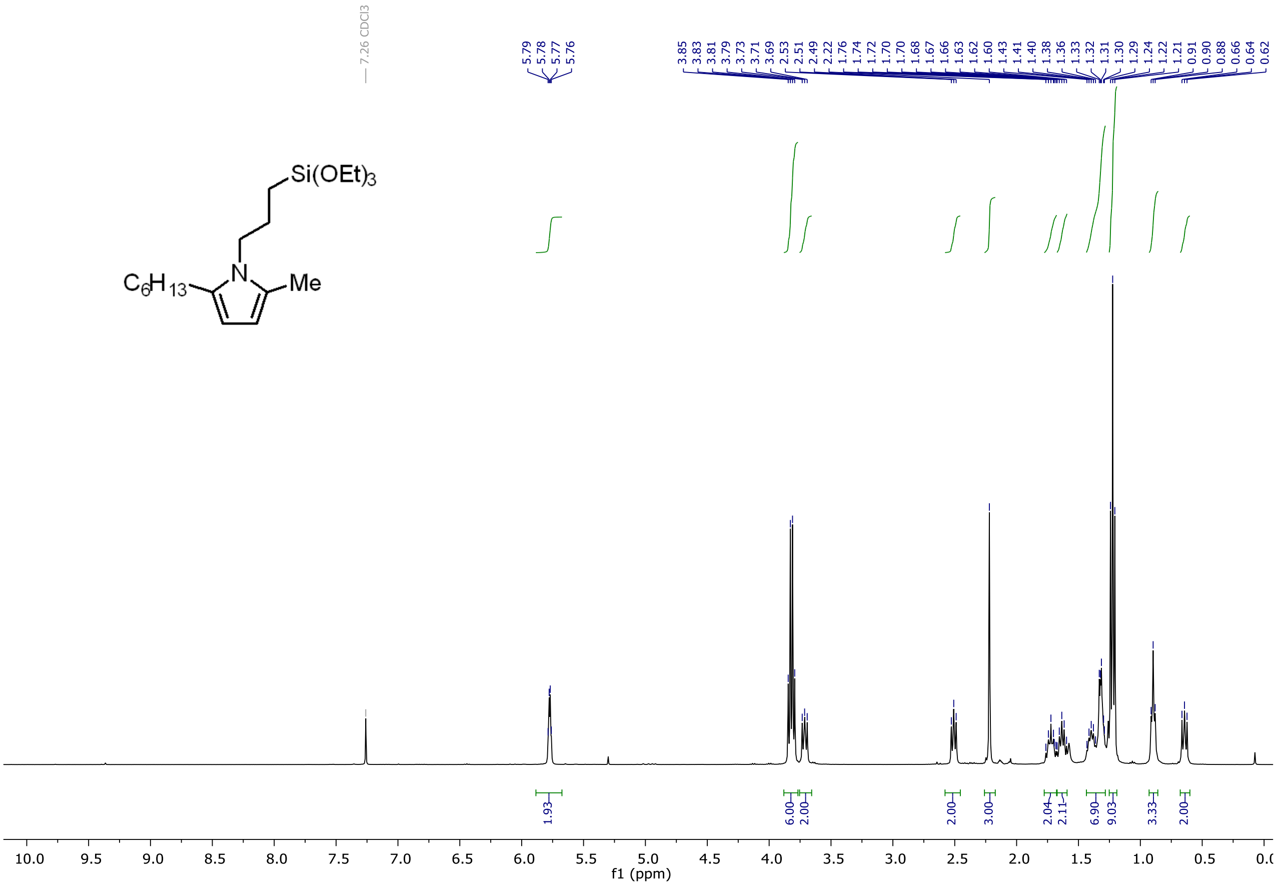

^{1}H NMR (400 MHz, $CDCl_3$) of ***2-hexyl-5-methyl-1-(3-(triethoxysilyl)propyl)-1H-pyrrole (5)***

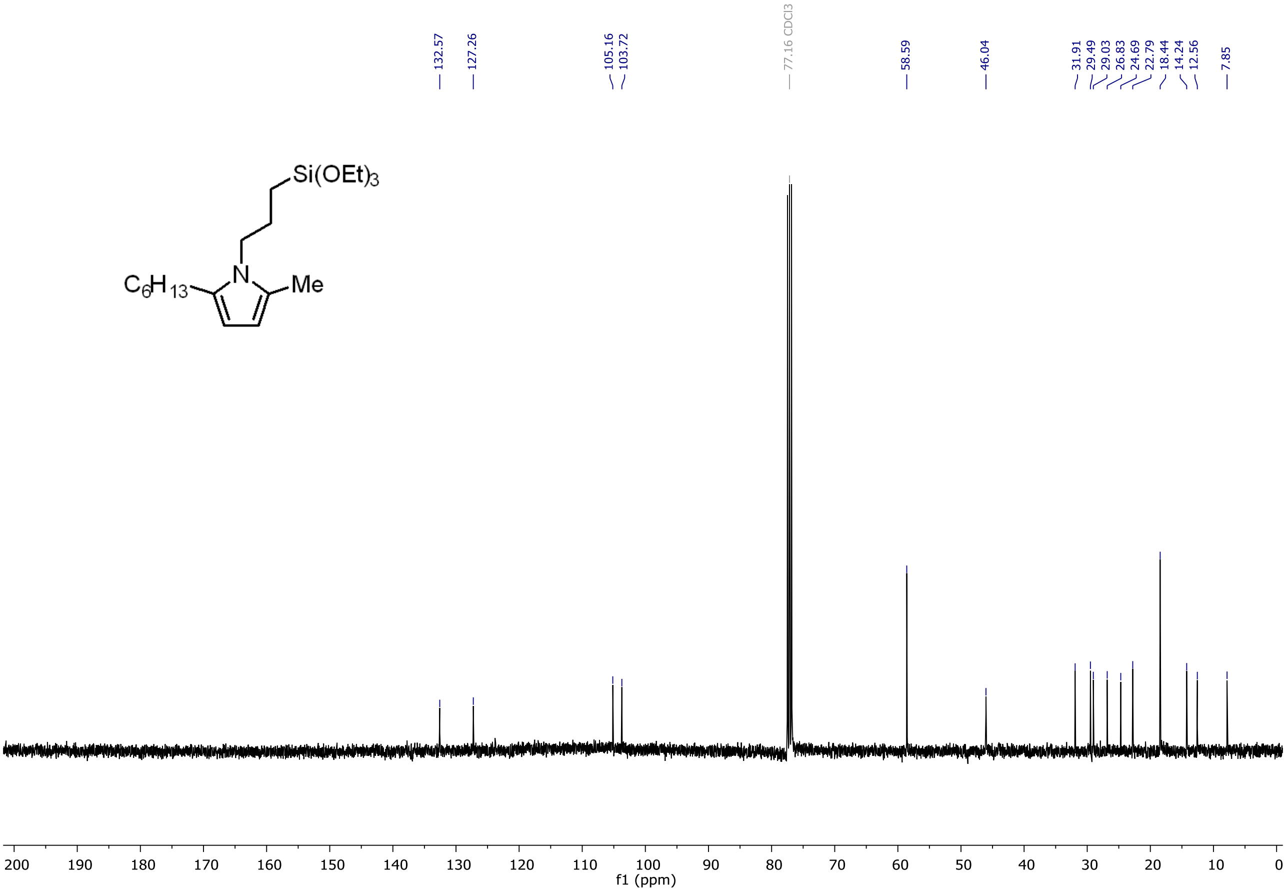

^{13}C NMR (101 MHz, $CDCl_3$) of ***2-hexyl-5-methyl-1-(3-(triethoxysilyl)propyl)-1H-pyrrole (5)***

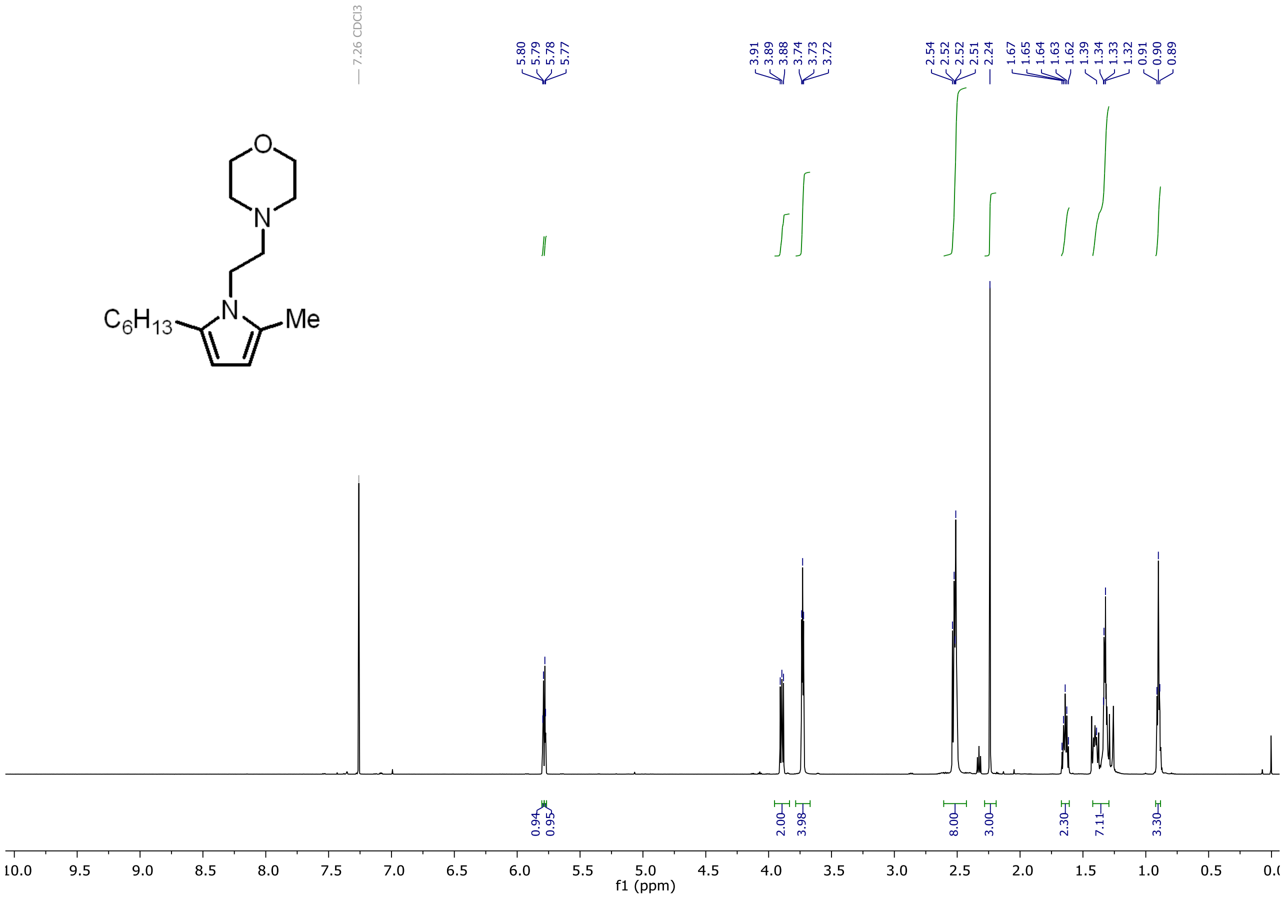

^{1}H NMR (600 MHz, CDCl$_3$) of ***4-(2-(2-hexyl-5-methyl-1H-pyrrol-1-yl)ethyl)morpholine (6)***

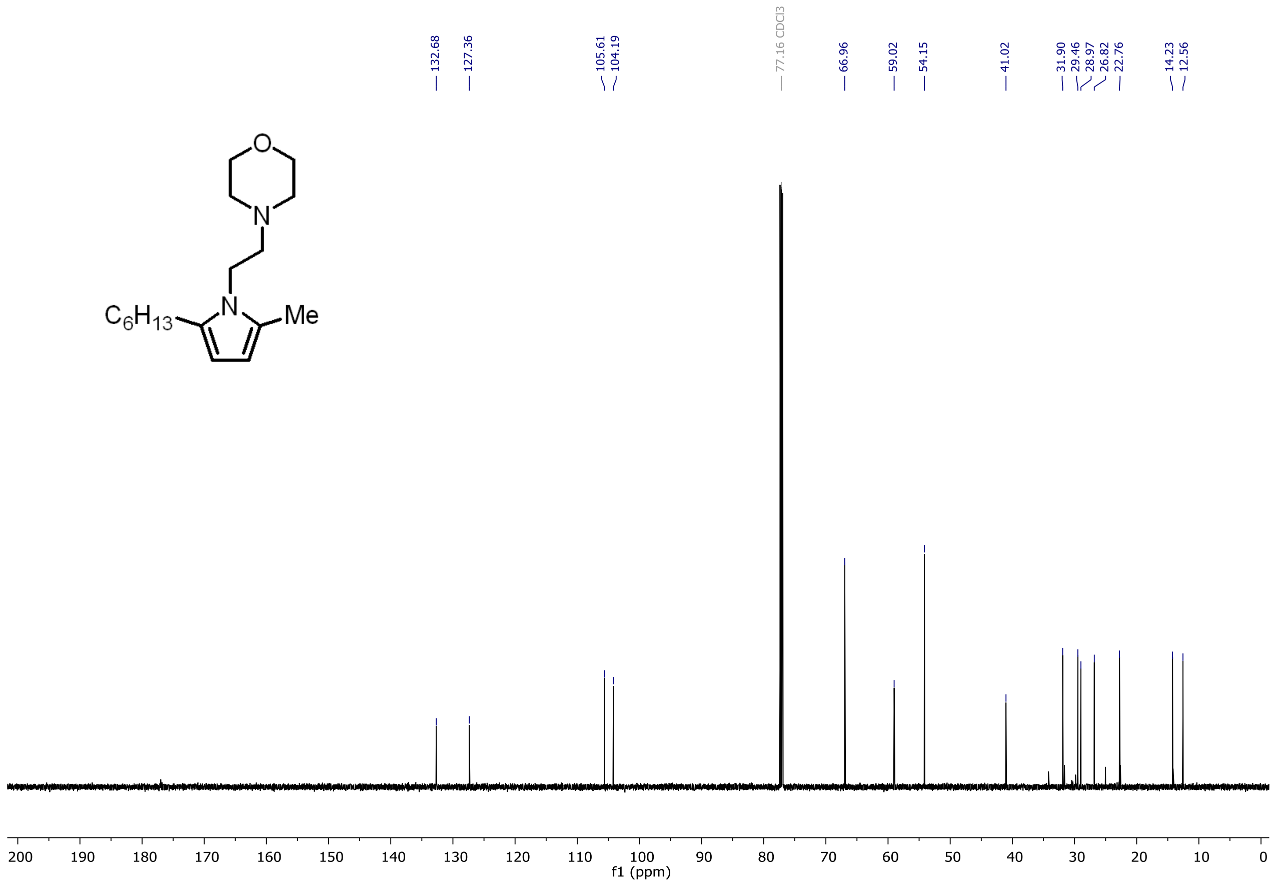

^{13}C NMR (151 MHz, CDCl$_3$) of ***4-(2-(2-hexyl-5-methyl-1H-pyrrol-1-yl)ethyl)morpholine (6)***

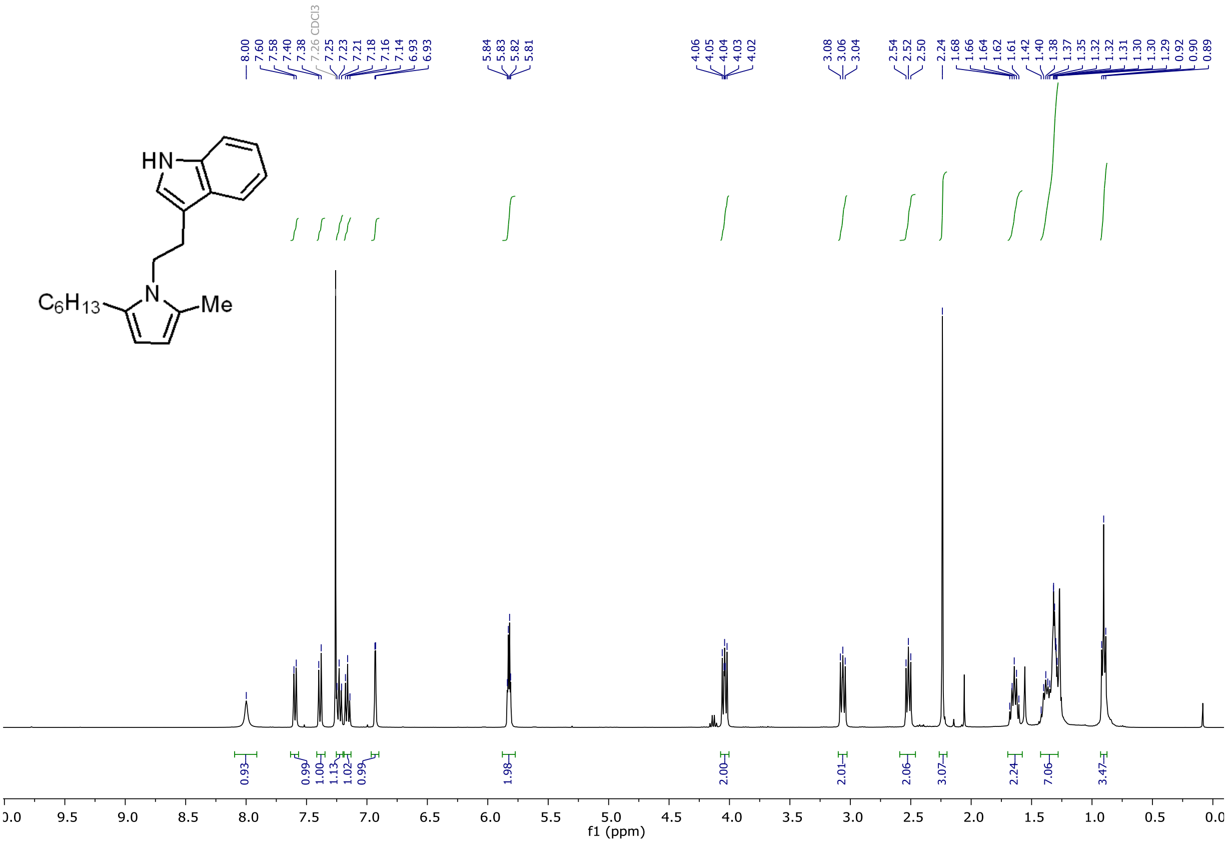

^{1}H NMR (400 MHz, CDCl$_3$) of ***3-(2-(2-hexyl-5-methyl-1H-pyrrol-1-yl)ethyl)-1H-indole (7)***

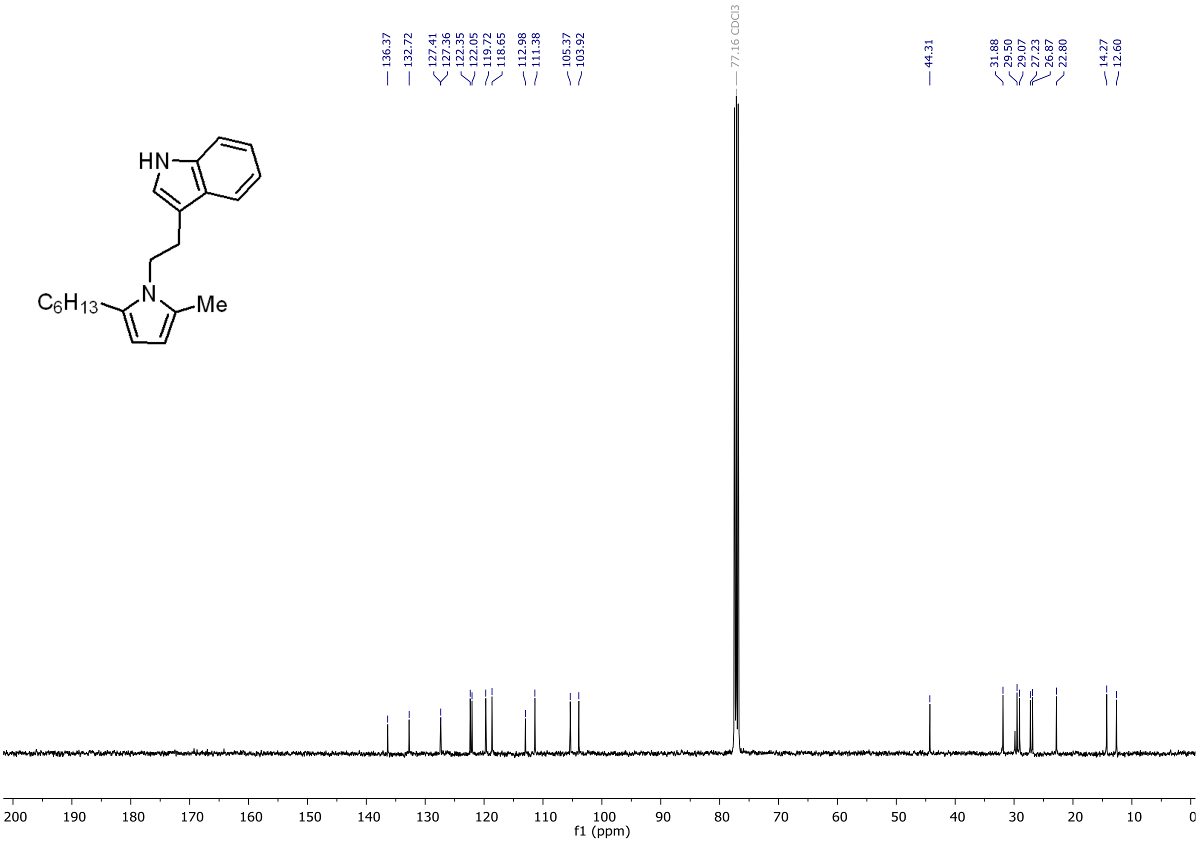

^{13}C NMR (101 MHz, CDCl$_3$) of ***3-(2-(2-hexyl-5-methyl-1H-pyrrol-1-yl)ethyl)-1H-indole (7)***

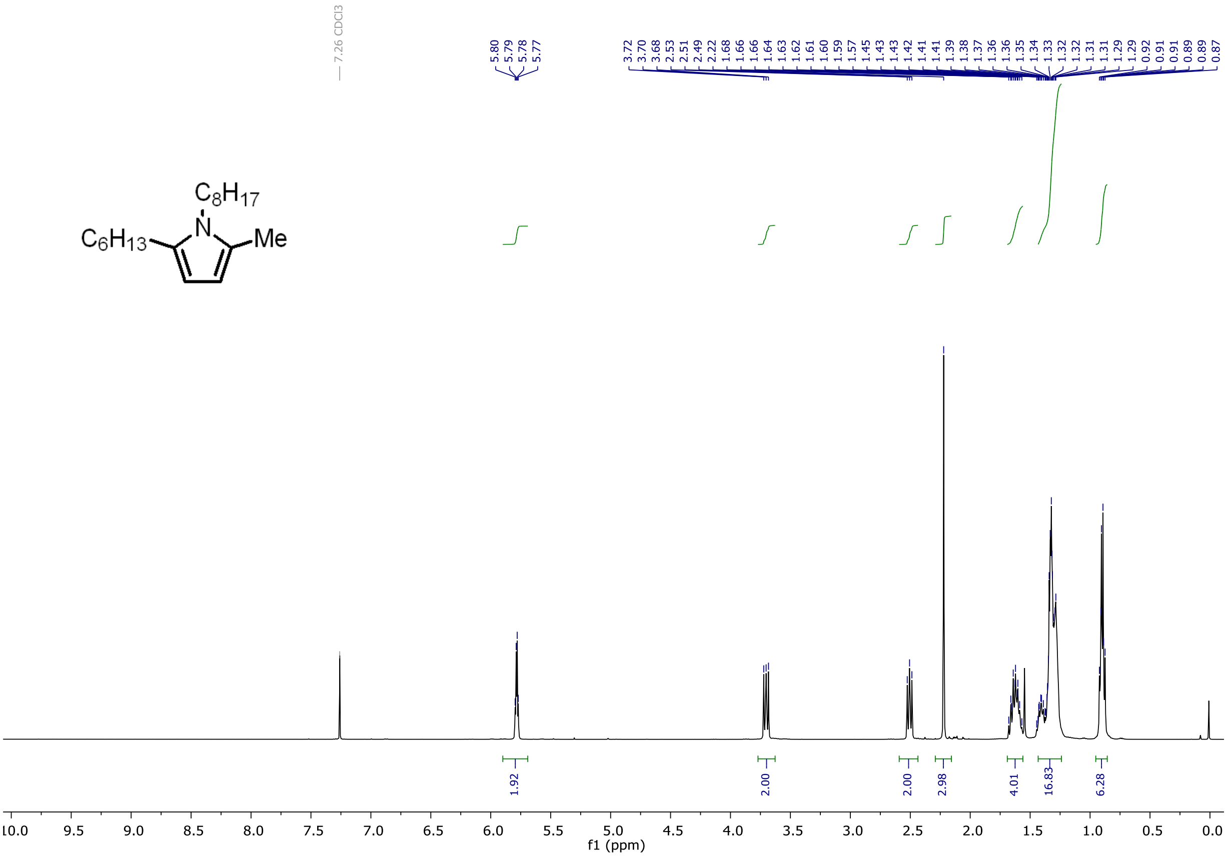

^{1}H NMR (400 MHz, $CDCl_3$) of ***2-hexyl-5-methyl-1-octyl-1H-pyrrole (8)***

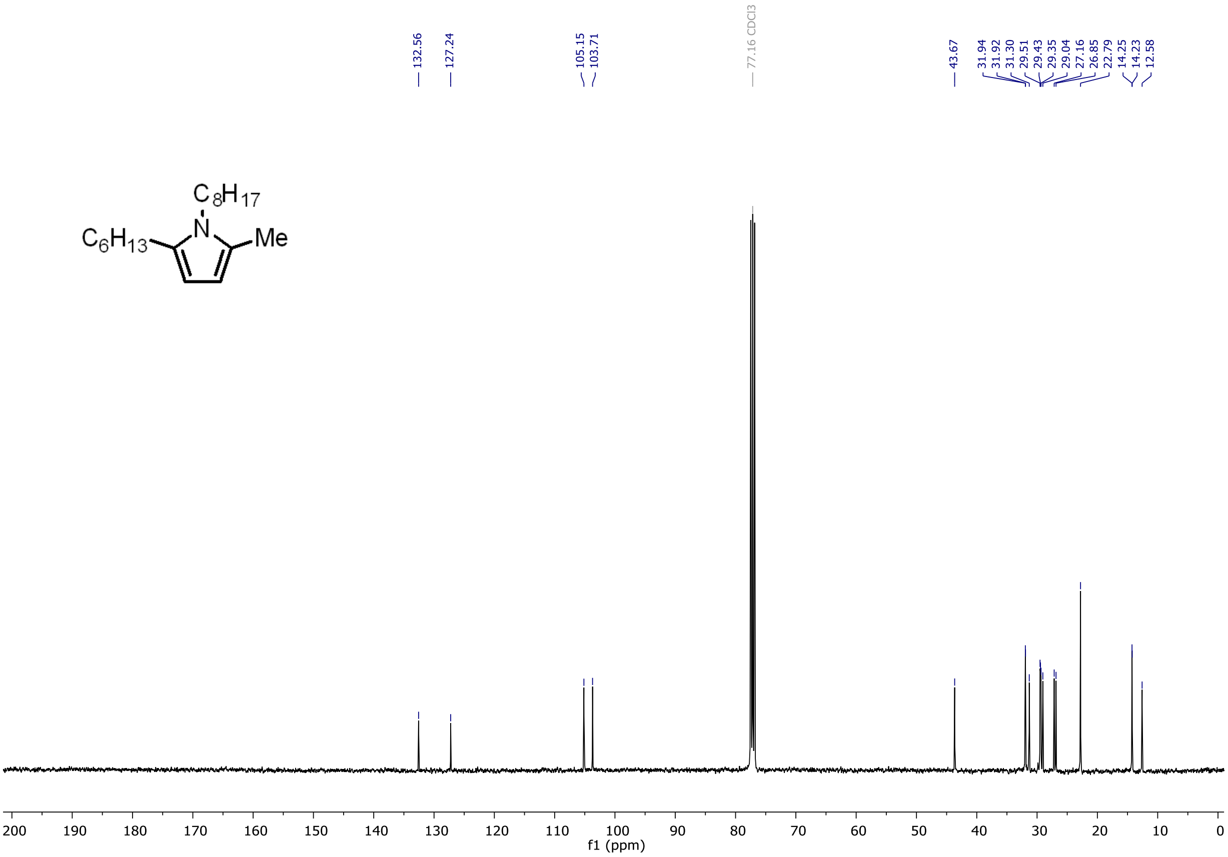

^{13}C NMR (101 MHz, $CDCl_3$) of ***2-hexyl-5-methyl-1-octyl-1H-pyrrole (8)***

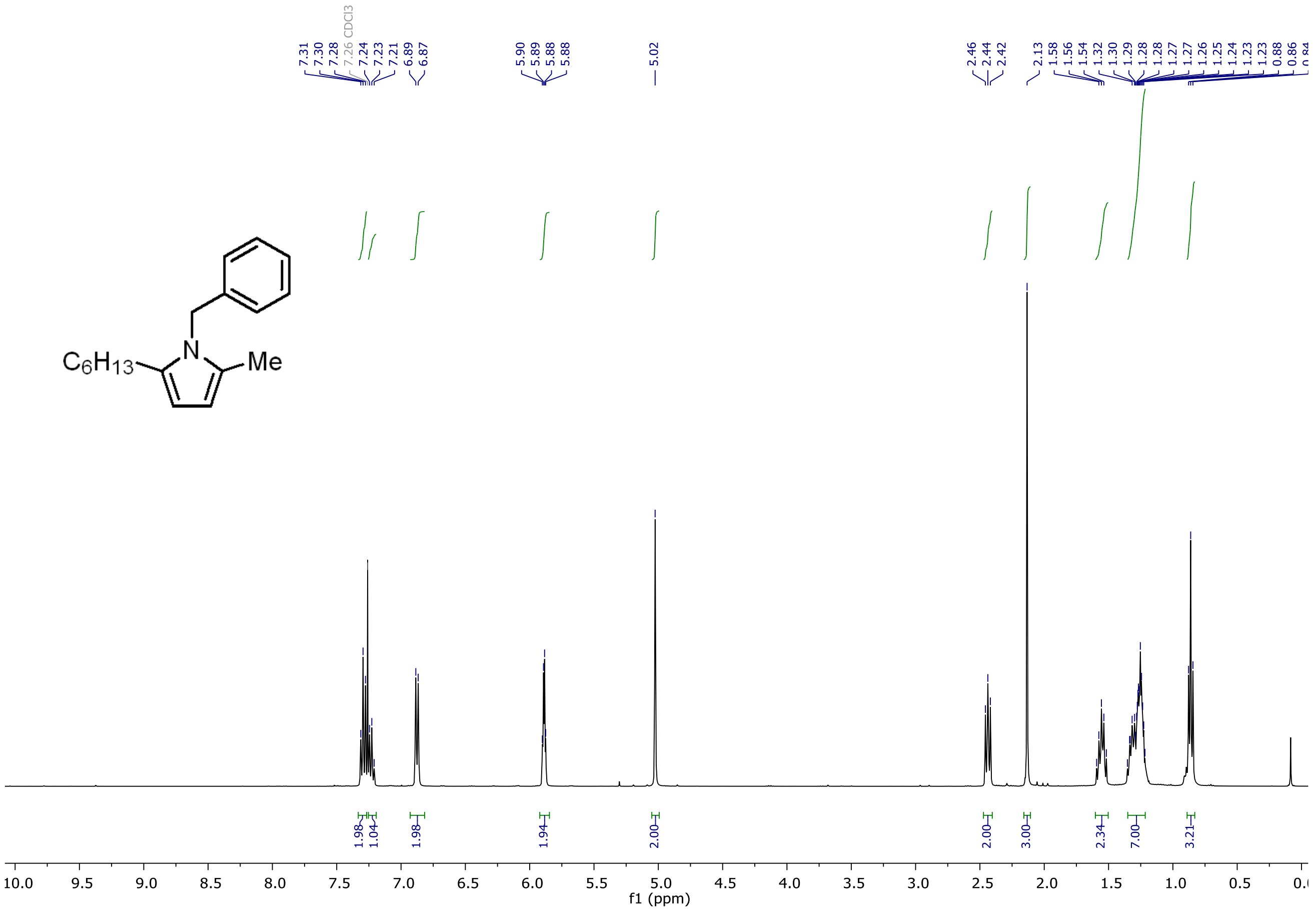

^{1}H NMR (400 MHz, $CDCl_3$) of ***1-benzyl-2-hexyl-5-methyl-1H-pyrrole (9)***

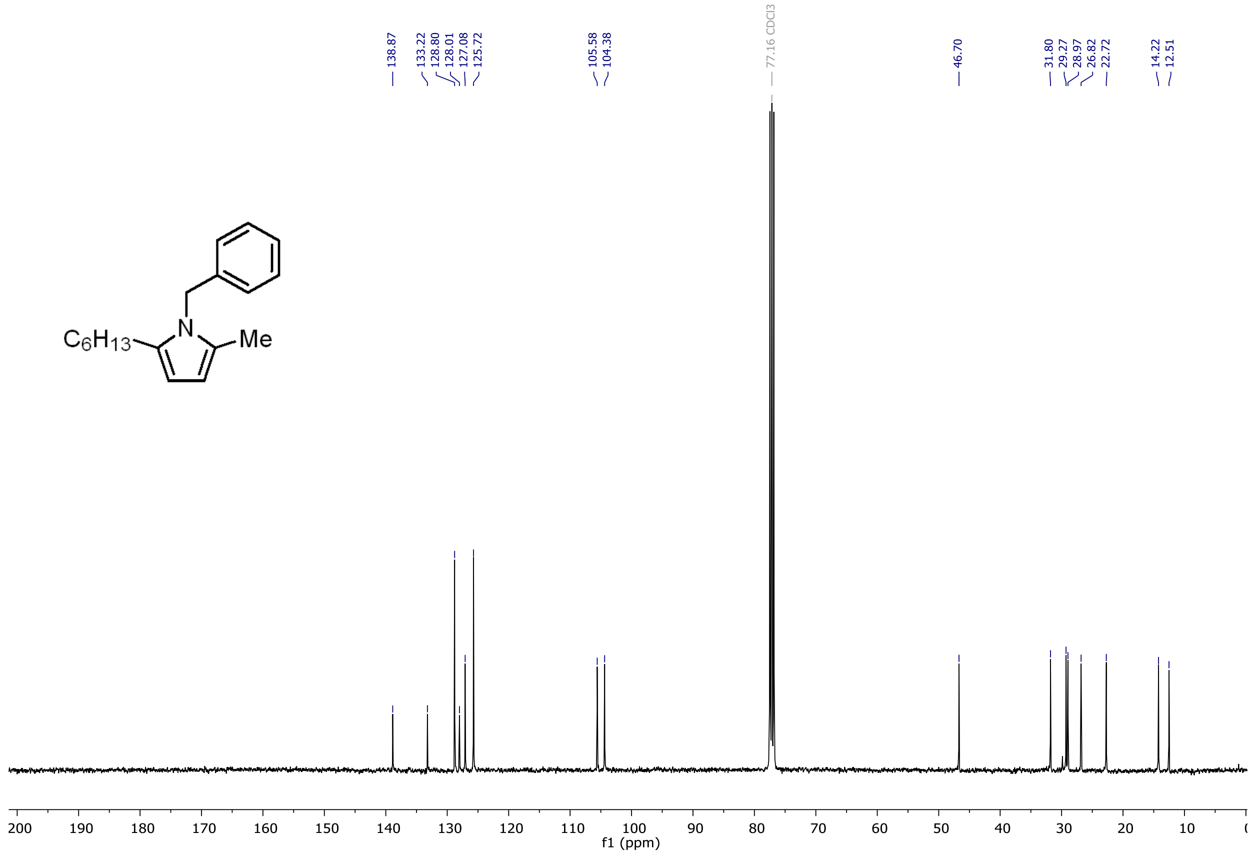

^{13}C NMR (101 MHz, $CDCl_3$) of ***1-benzyl-2-hexyl-5-methyl-1H-pyrrole (9)***

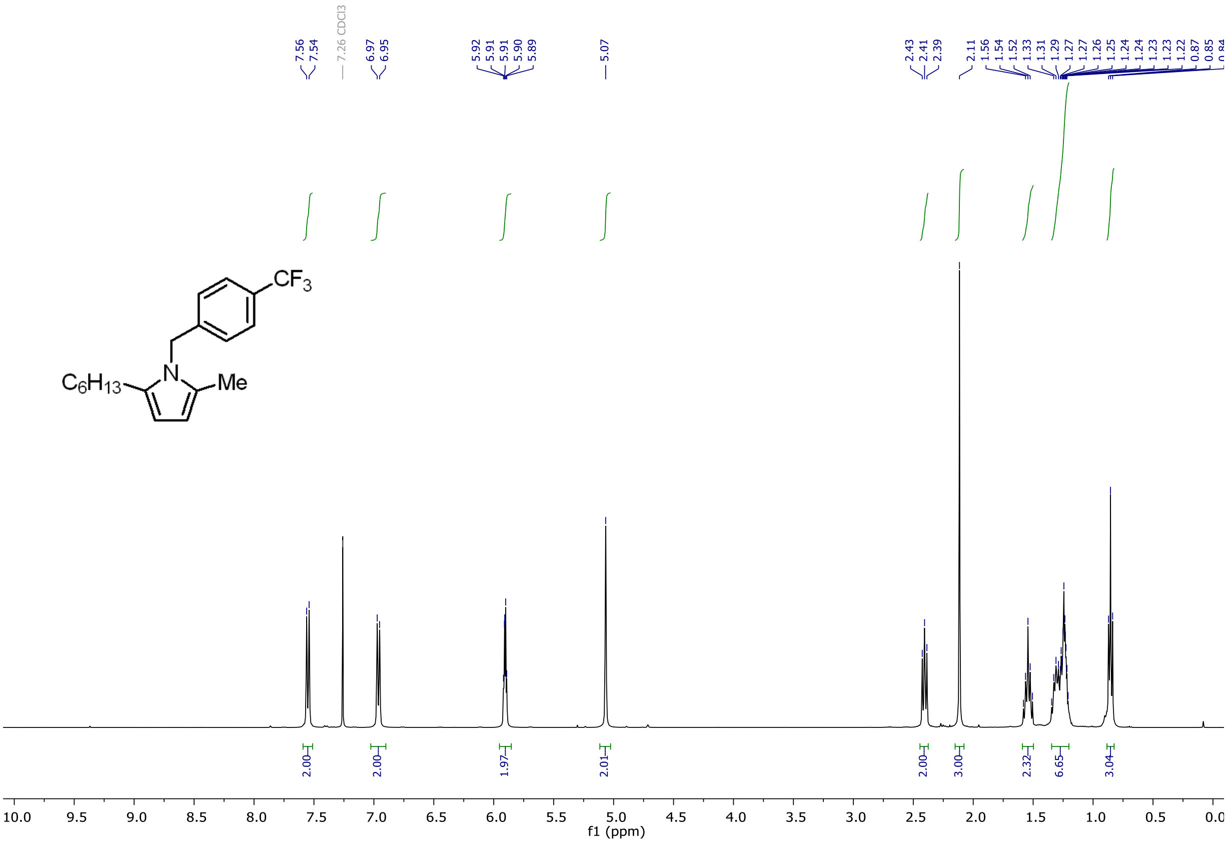

^{1}H NMR (400 MHz, $CDCl_3$) of ***2-hexyl-5-methyl-1-(4-(trifluoromethyl)benzyl)-1H-pyrrole (10)***

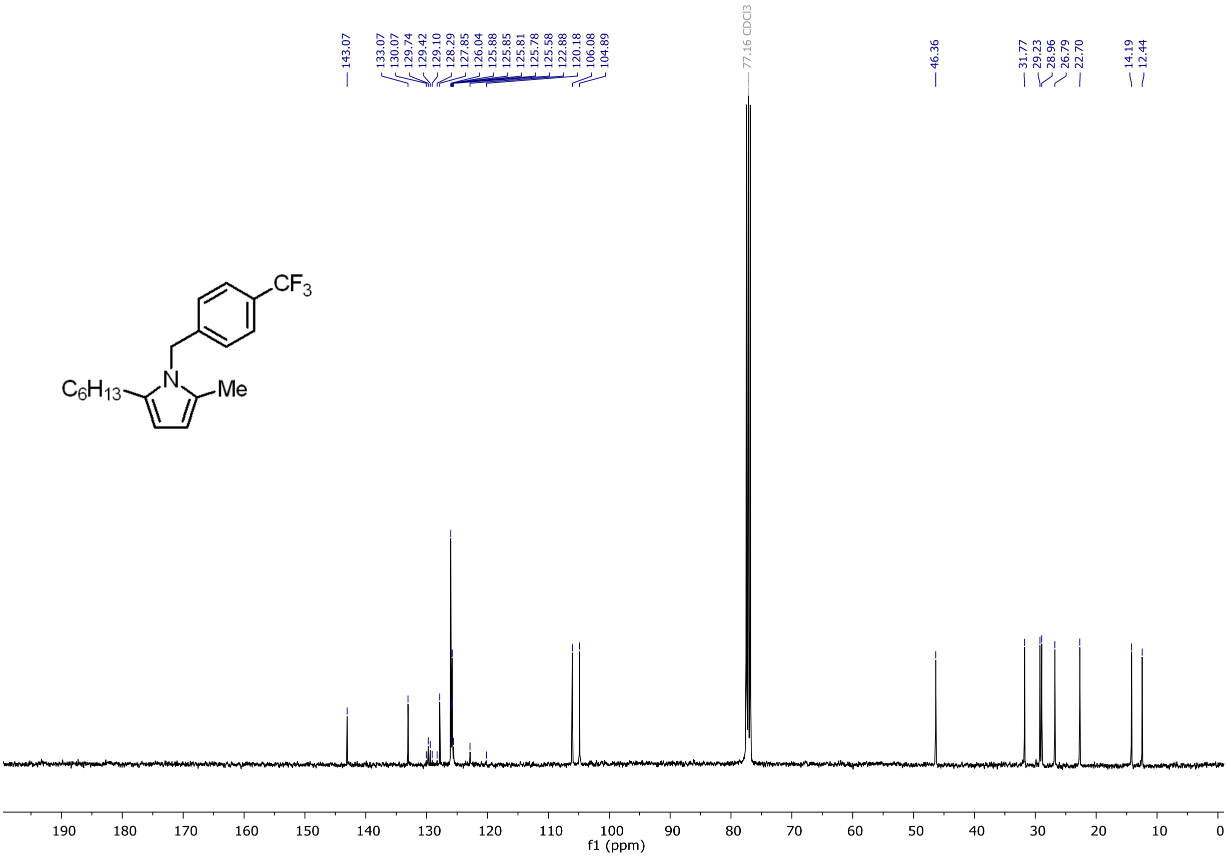

^{13}C NMR (101 MHz, $CDCl_3$) of ***2-hexyl-5-methyl-1-(4-(trifluoromethyl)benzyl)-1H-pyrrole (10)***

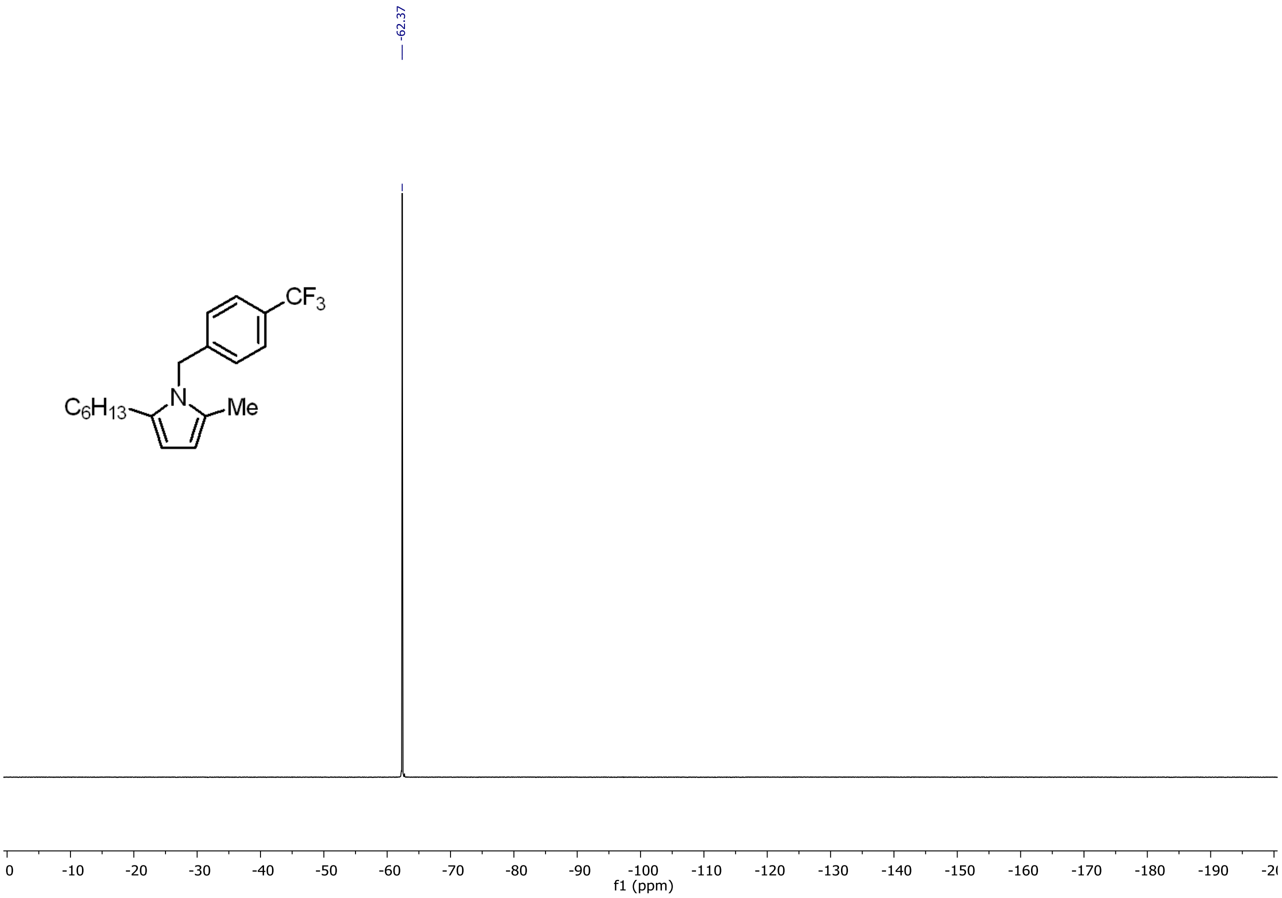

^{14}F NMR (565 MHz, $CDCl_3$) of ***2-hexyl-5-methyl-1-(4-(trifluoromethyl)benzyl)-1H-pyrrole (10)***

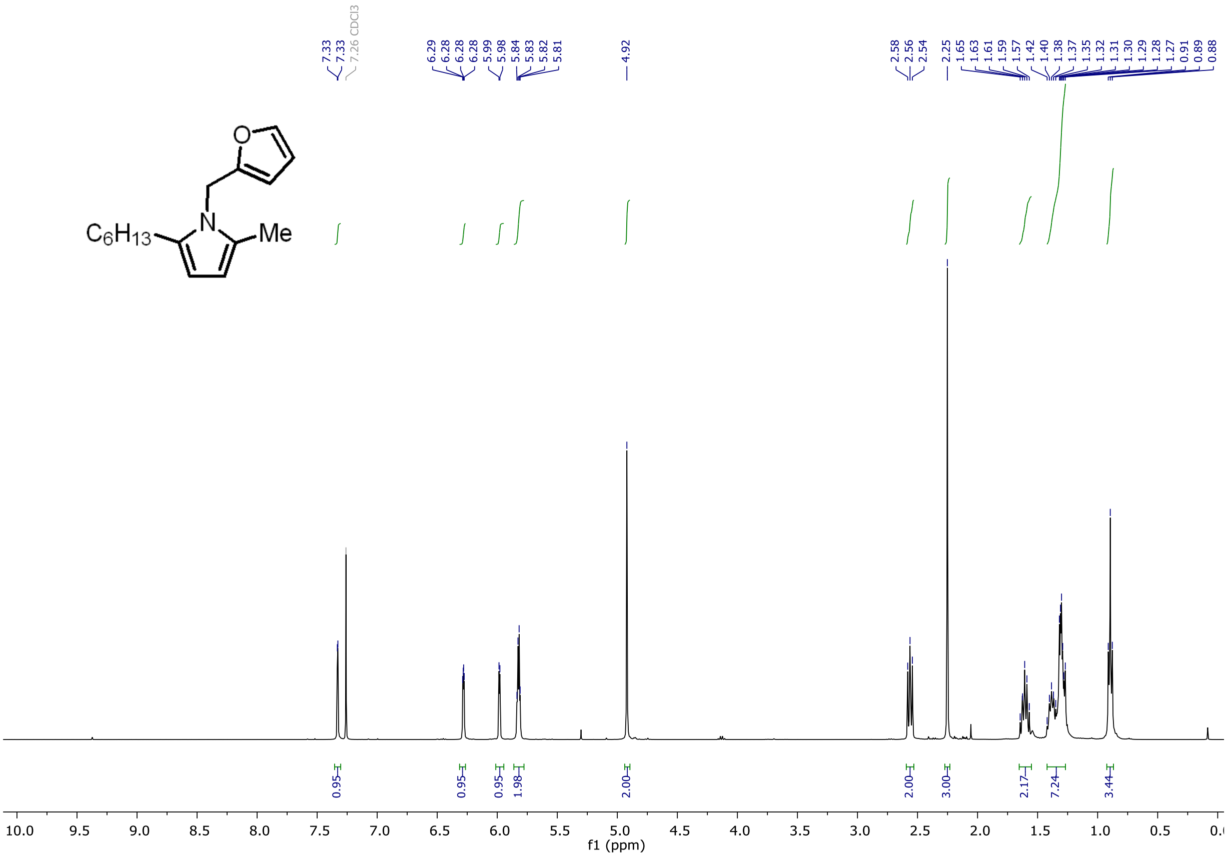

^{1}**H NMR (400 MHz, CDCl$_3$)** of ***1-(furan-2-ylmethyl)-2-hexyl-5-methyl-1H-pyrrole (11)***

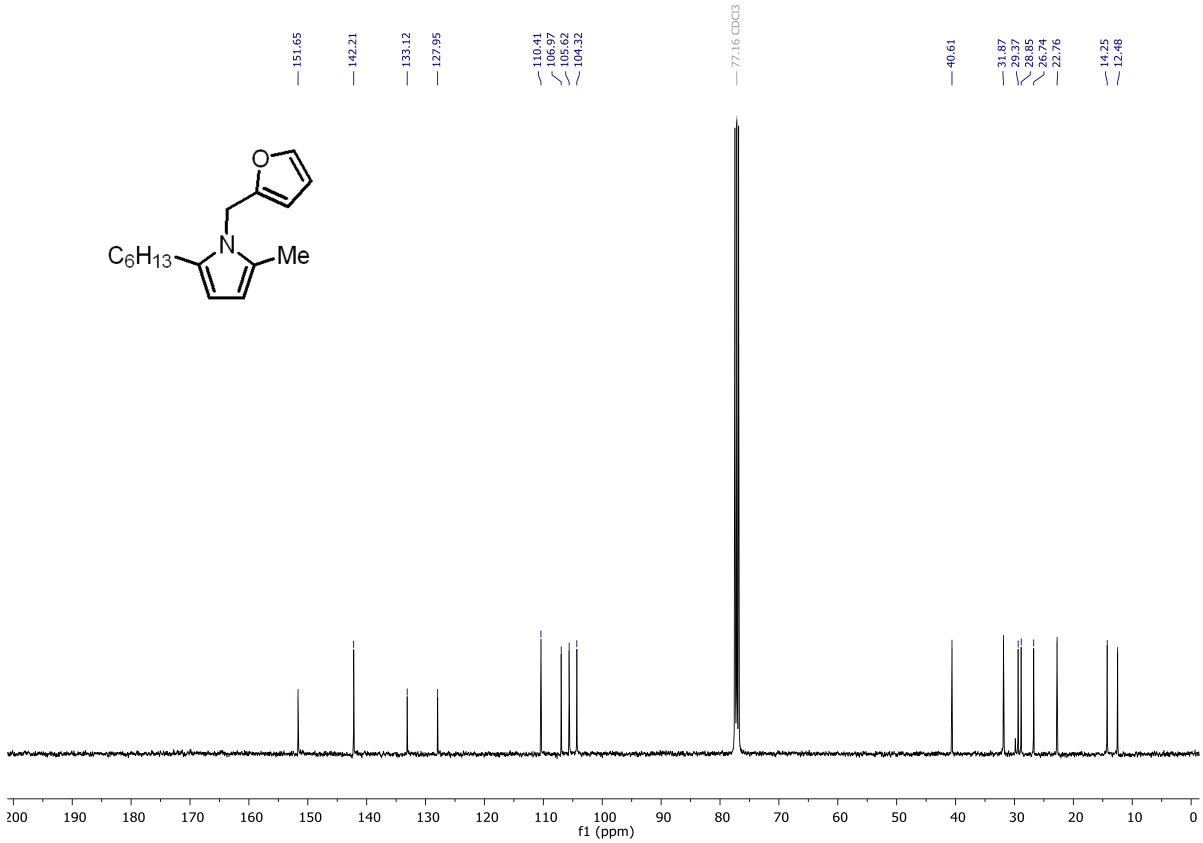

^{13}**C NMR (101 MHz, CDCl$_3$)** of ***1-(furan-2-ylmethyl)-2-hexyl-5-methyl-1H-pyrrole (11)***

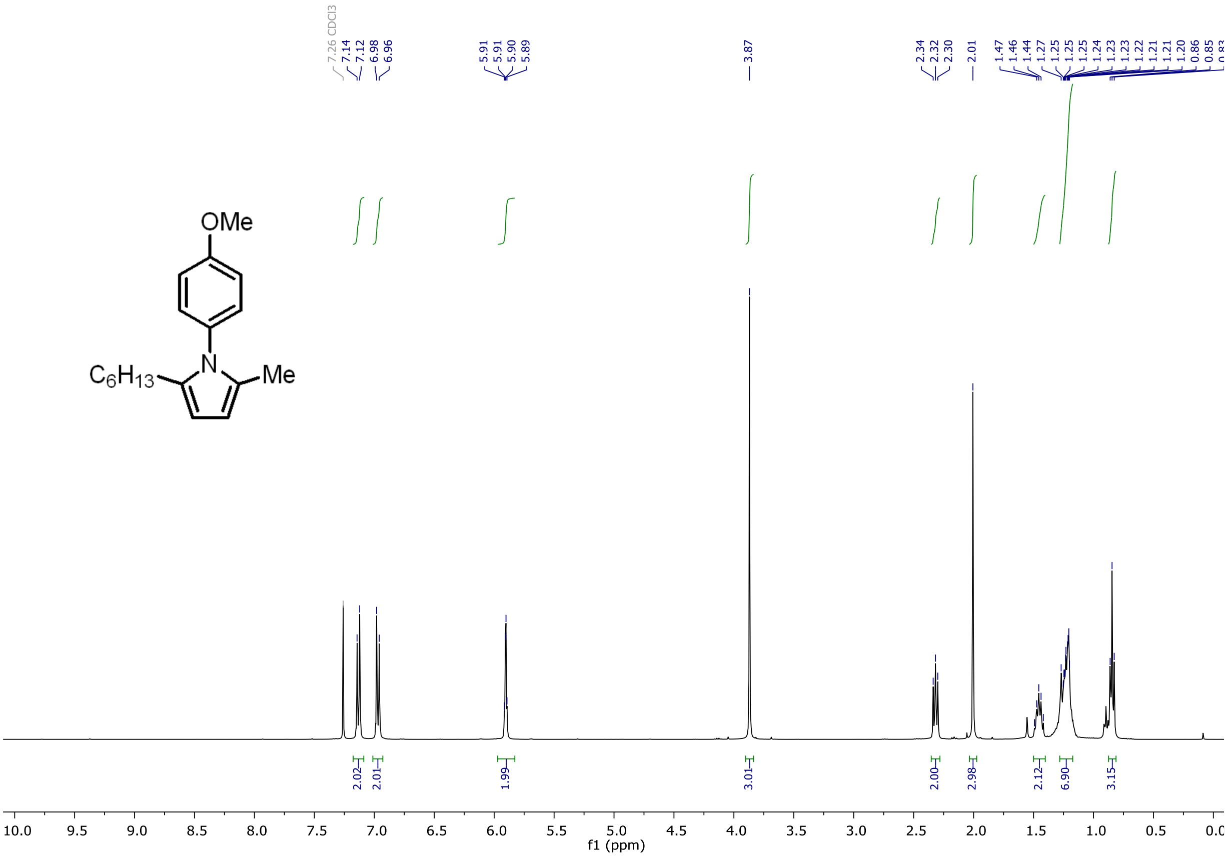

^{1}H NMR (400 MHz, $CDCl_3$) of ***2-hexyl-1-(4-methoxyphenyl)-5-methyl-1H-pyrrole (12)***

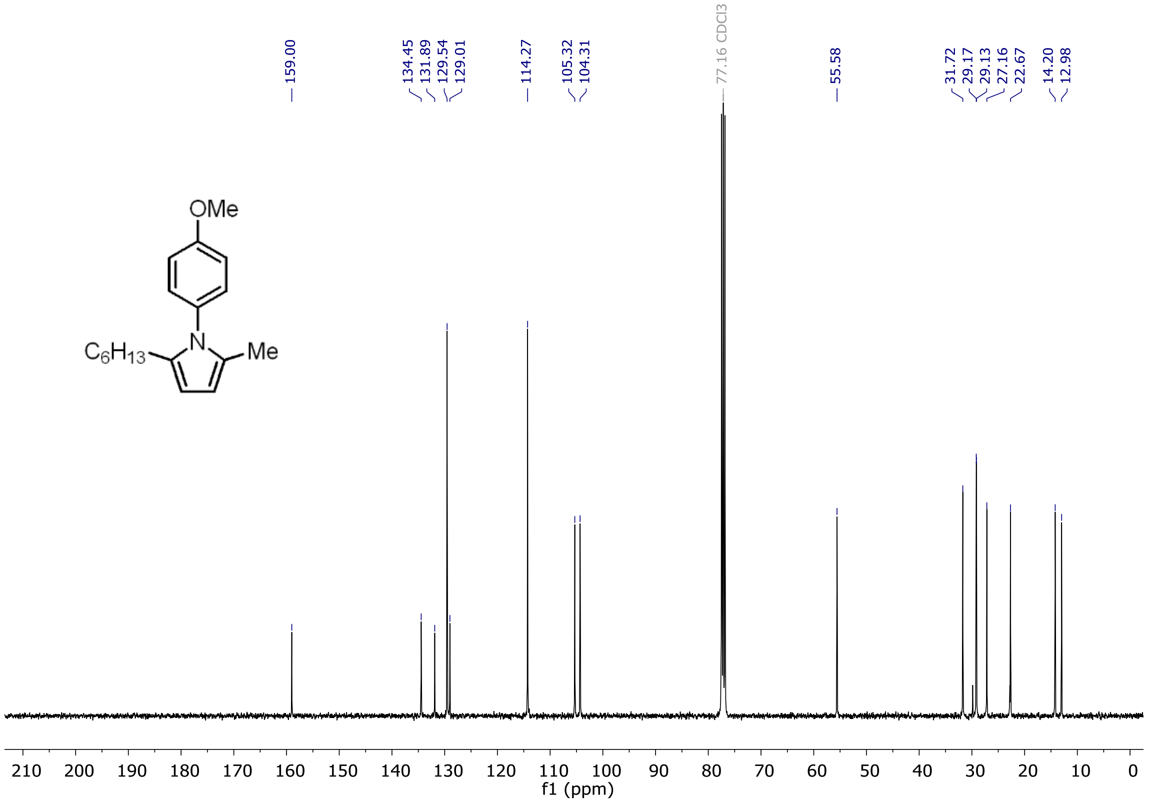

^{13}C NMR (101 MHz, $CDCl_3$) of ***2-hexyl-1-(4-methoxyphenyl)-5-methyl-1H-pyrrole (12)***

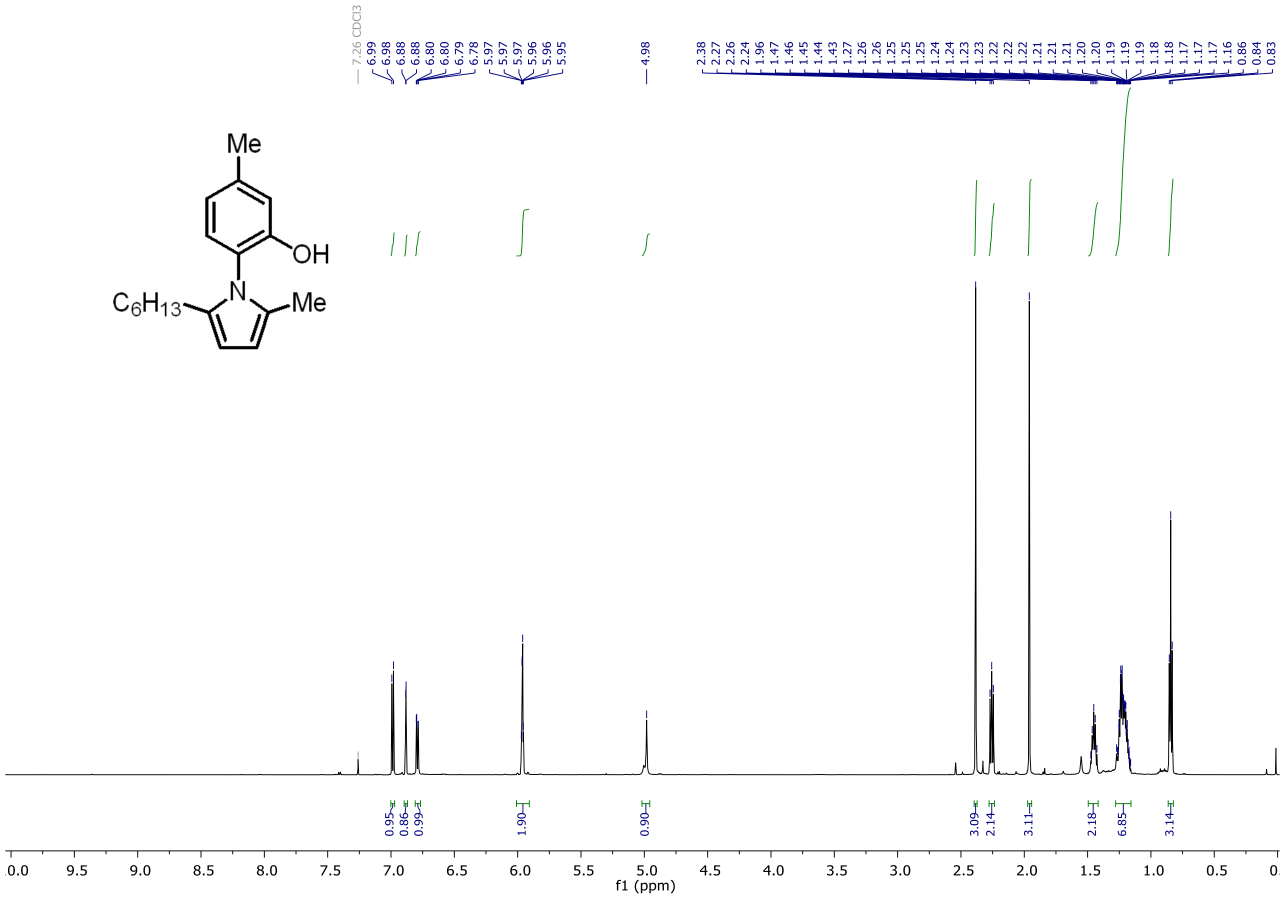

^{1}H NMR (600 MHz, CDCl$_3$) of ***2-(2-hexyl-5-methyl-1H-pyrrol-1-yl)-5-methylphenol (13)***

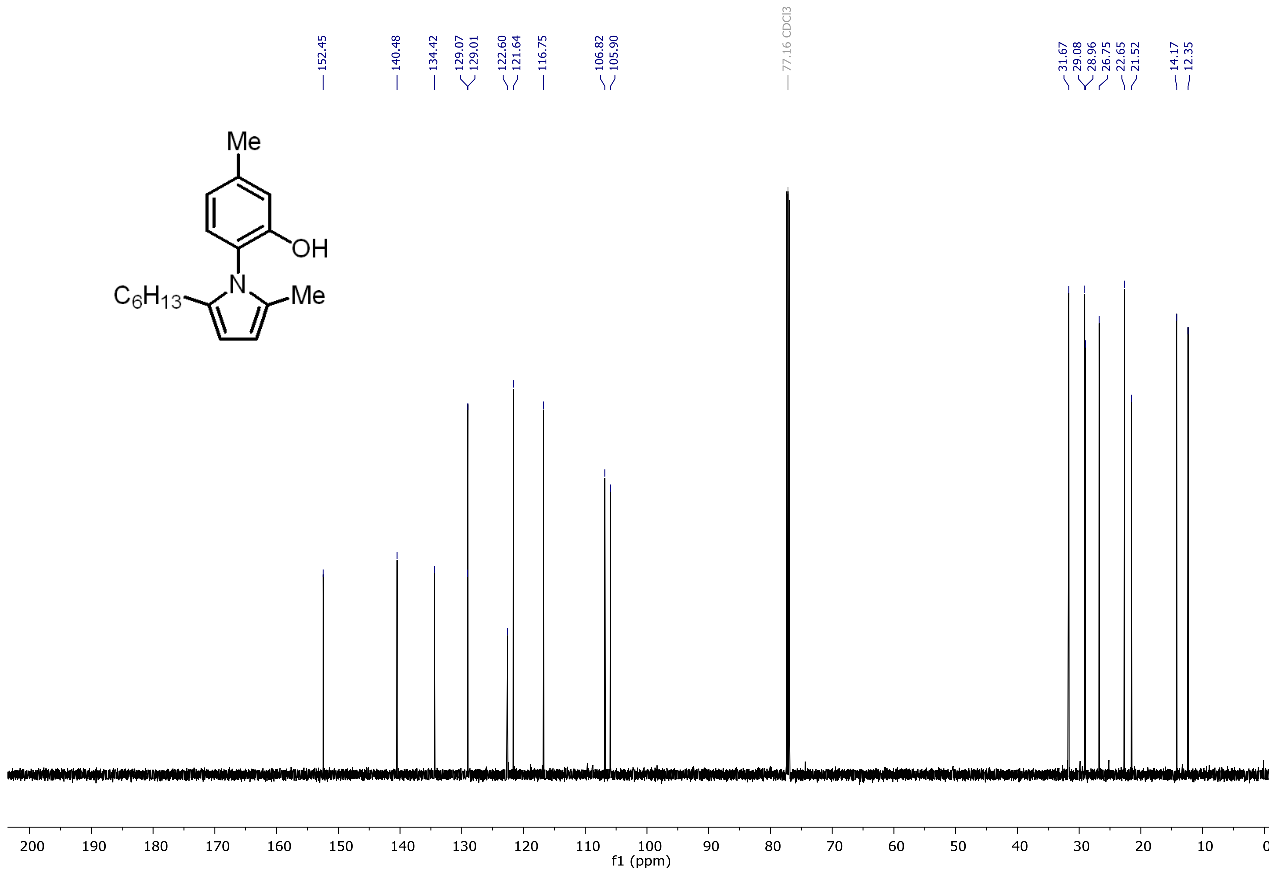

^{13}C NMR (151 MHz, CDCl$_3$) of ***2-(2-hexyl-5-methyl-1H-pyrrol-1-yl)-5-methylphenol (13)***

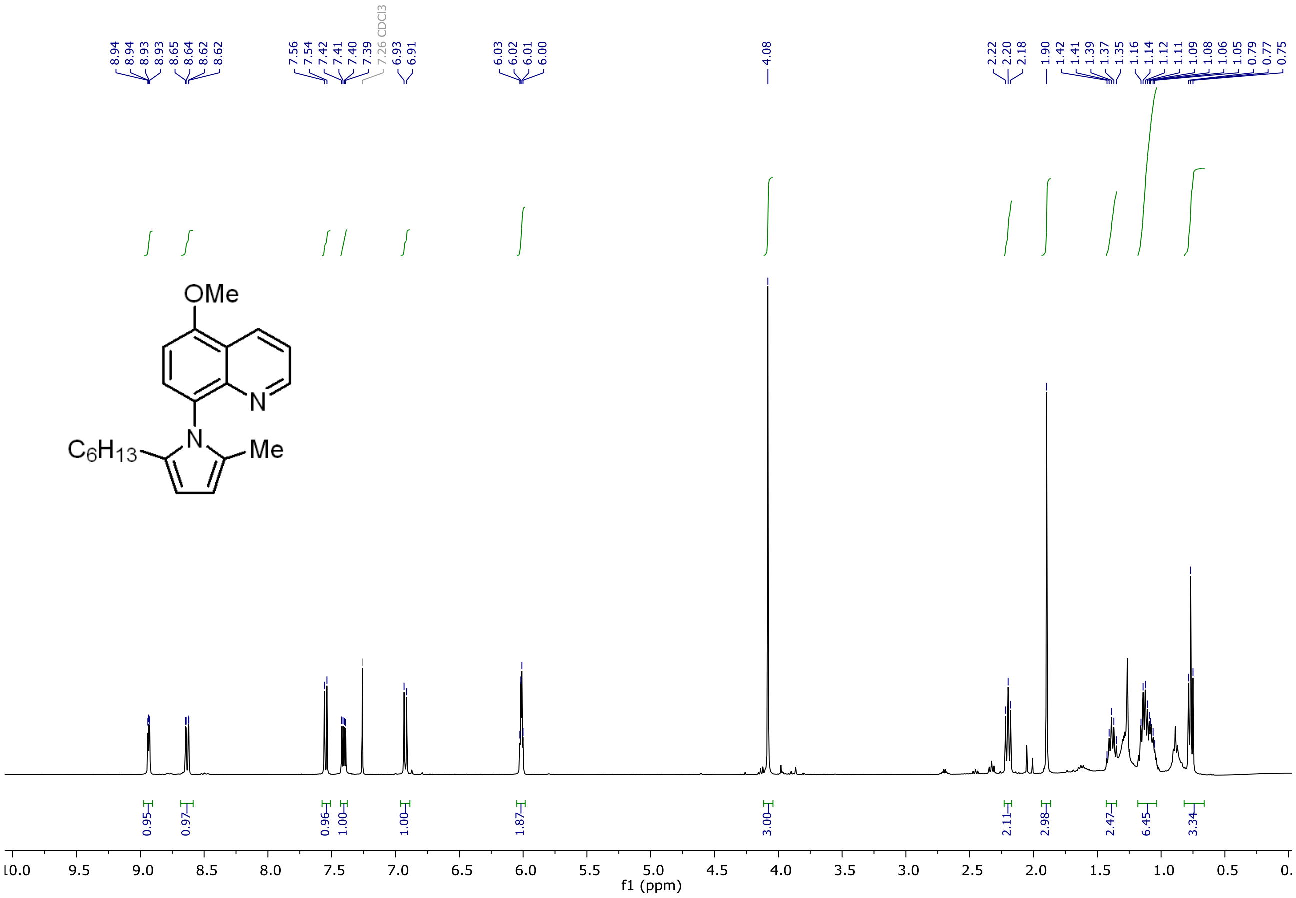

^{1}H NMR (400 MHz, CDCl$_3$) of ***8-(2-hexyl-5-methyl-1H-pyrrol-1-yl)-5-methoxyquinoline (14)***

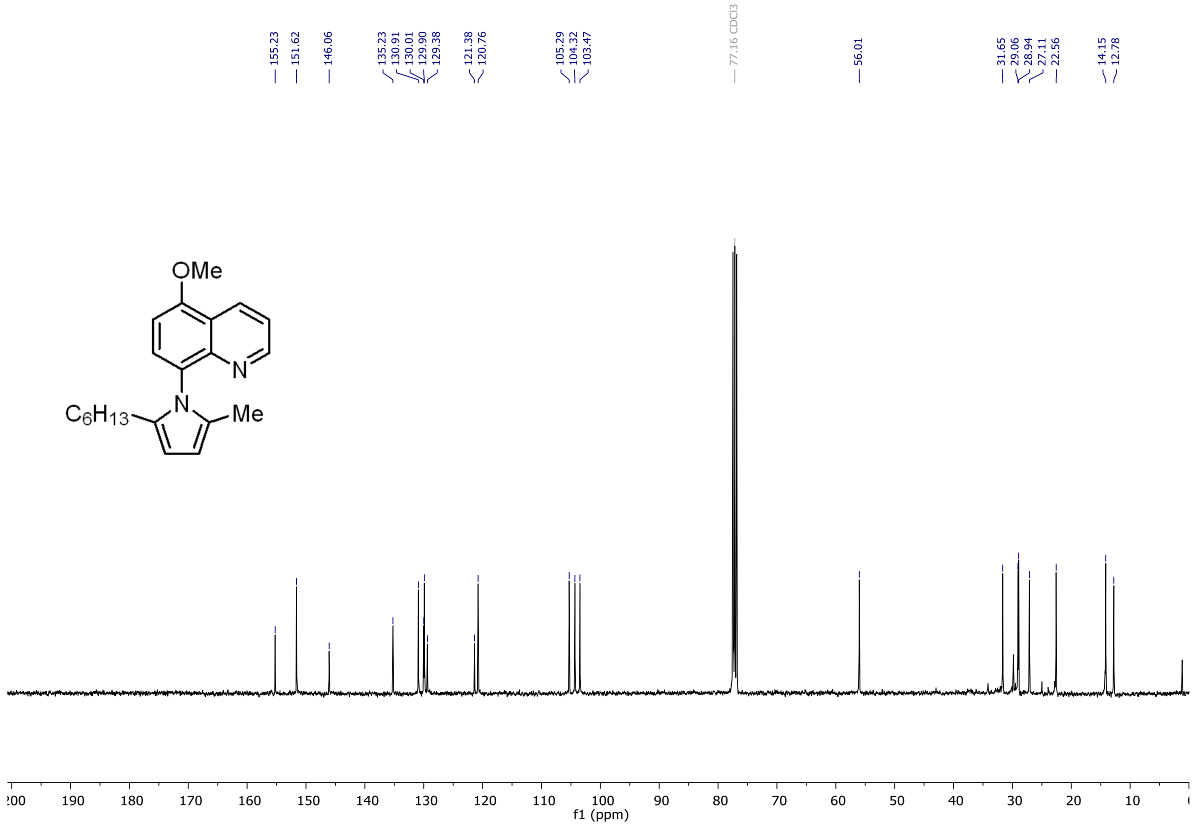

^{13}C NMR (101 MHz, CDCl$_3$) of ***8-(2-hexyl-5-methyl-1H-pyrrol-1-yl)-5-methoxyquinoline (14)***

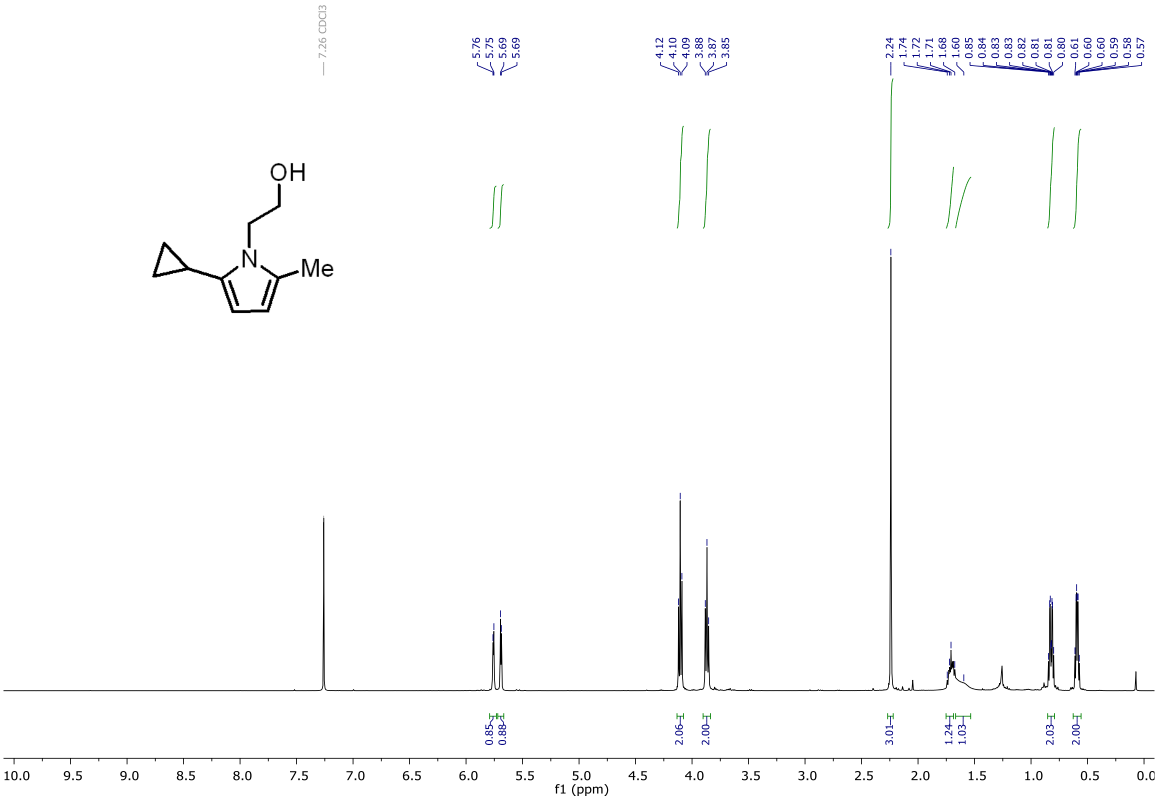

^{1}H NMR (400 MHz, $CDCl_3$) of ***2-(2-cyclopropyl-5-methyl-1H-pyrrol-1-yl)ethan-1-ol (15)***

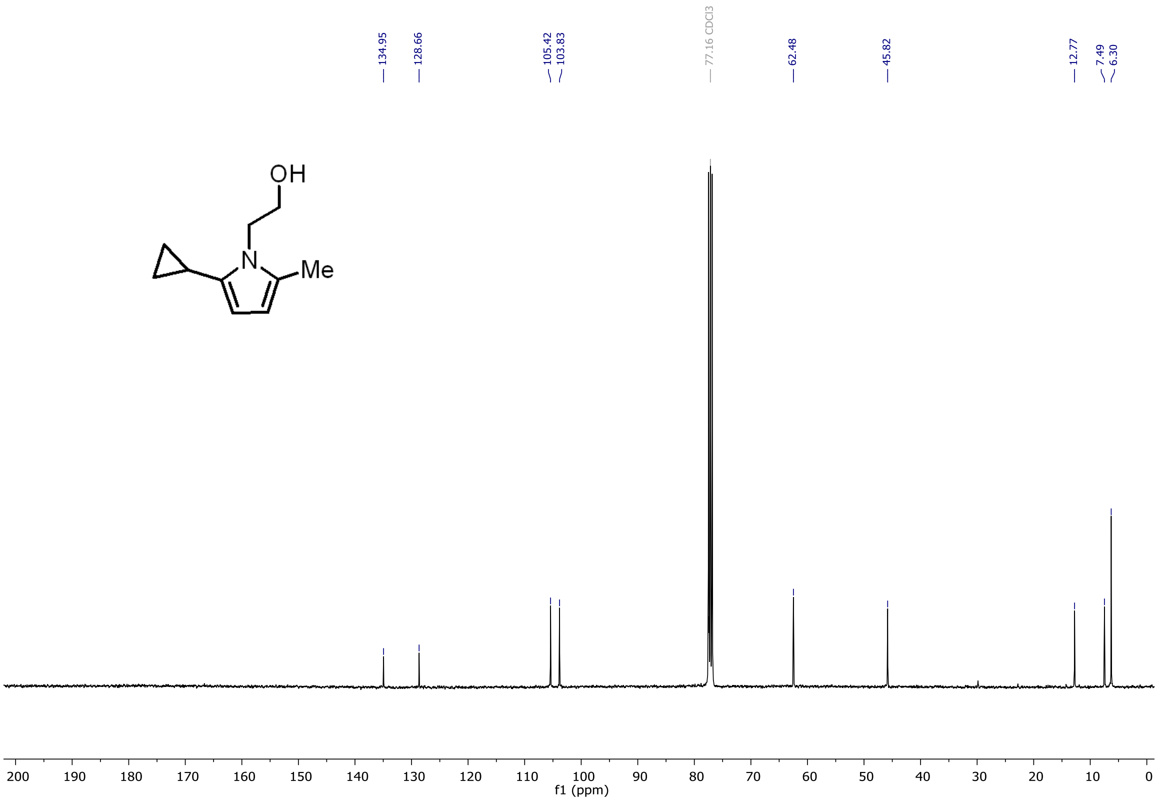

^{13}C NMR (101 MHz, $CDCl_3$) of ***2-(2-cyclopropyl-5-methyl-1H-pyrrol-1-yl)ethan-1-ol (15)***

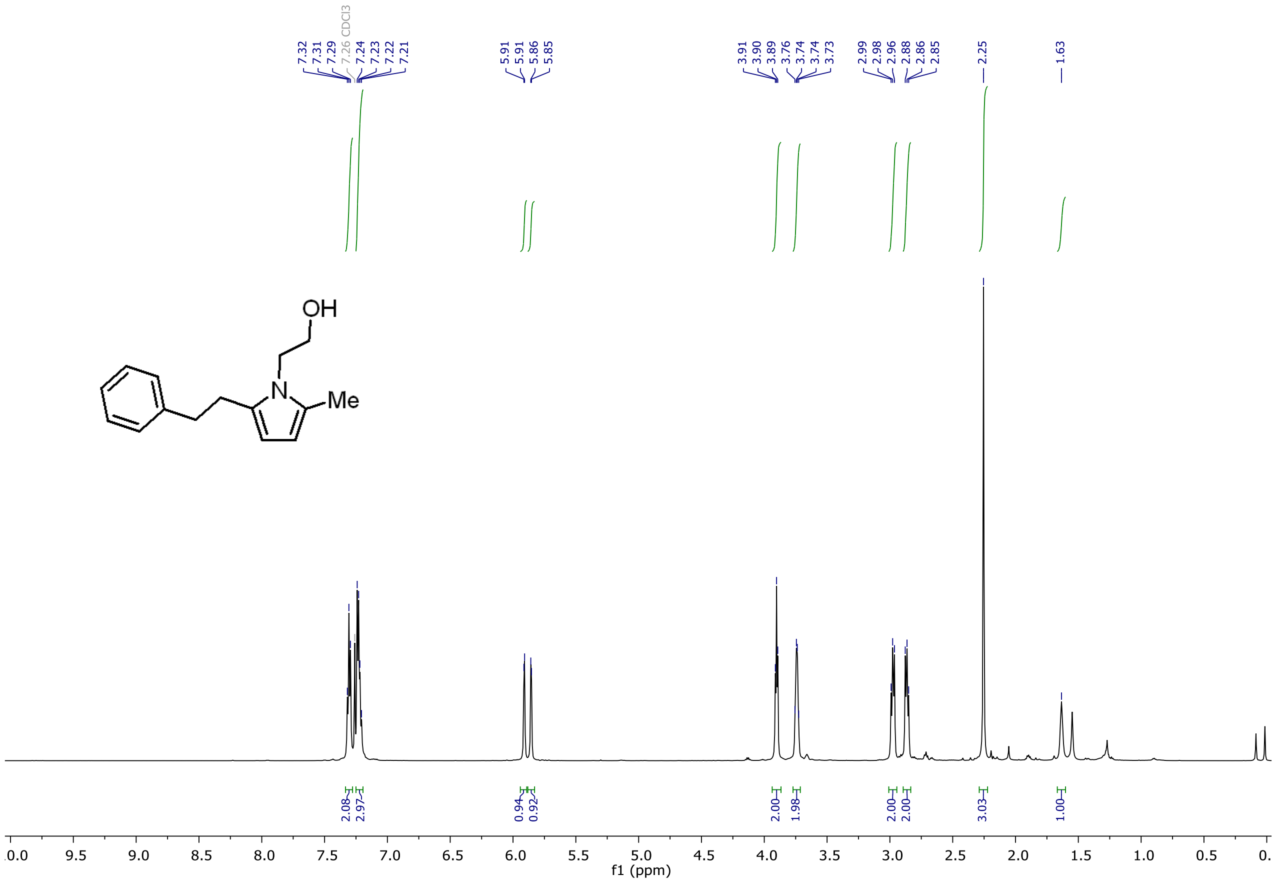

^{1}H NMR (600 MHz, CDCl$_3$) of ***2-(2-methyl-5-phenethyl-1H-pyrrol-1-yl)ethan-1-ol (16)***

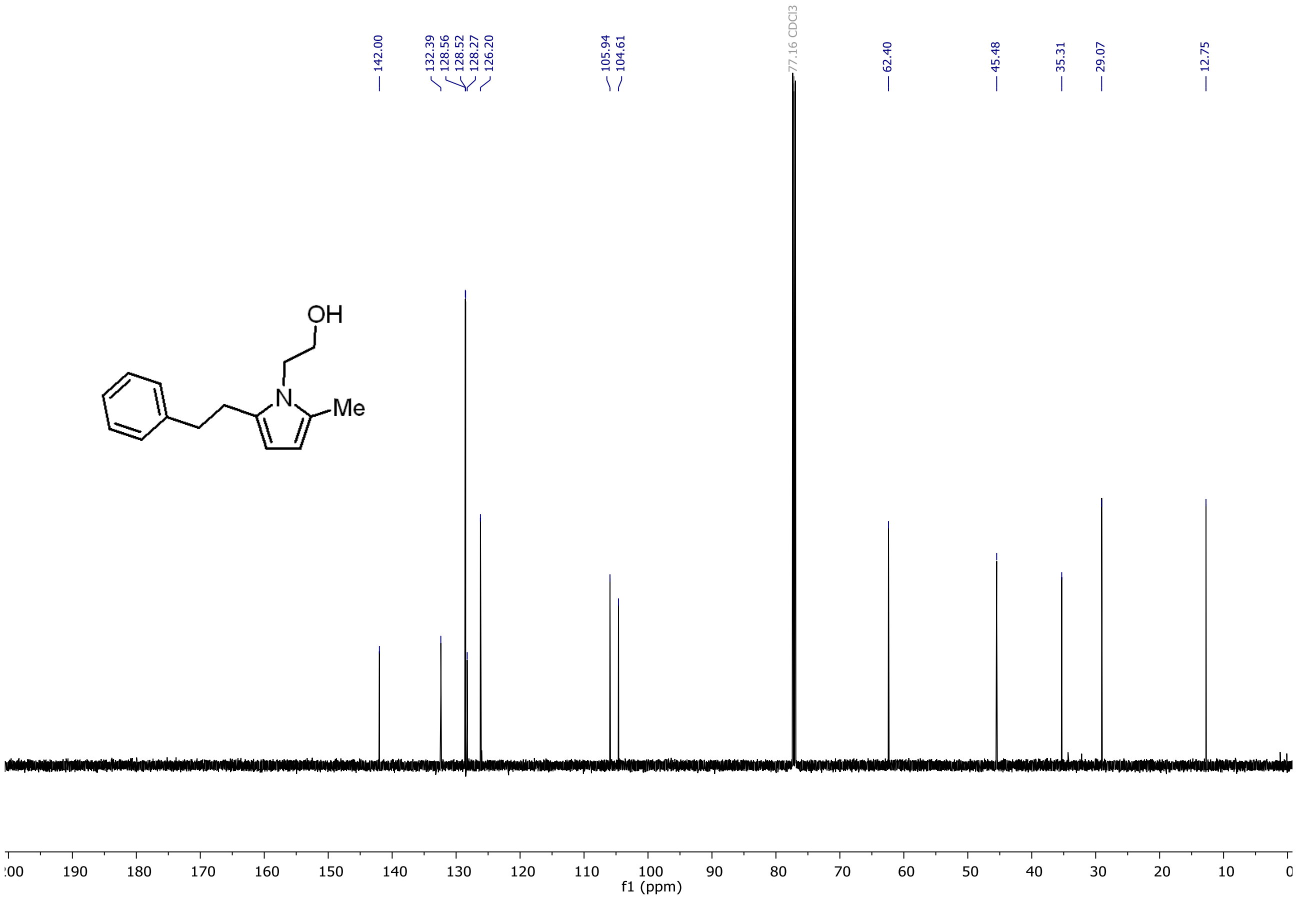

^{13}C NMR (151 MHz, CDCl$_3$) of ***2-(2-methyl-5-phenethyl-1H-pyrrol-1-yl)ethan-1-ol (16)***

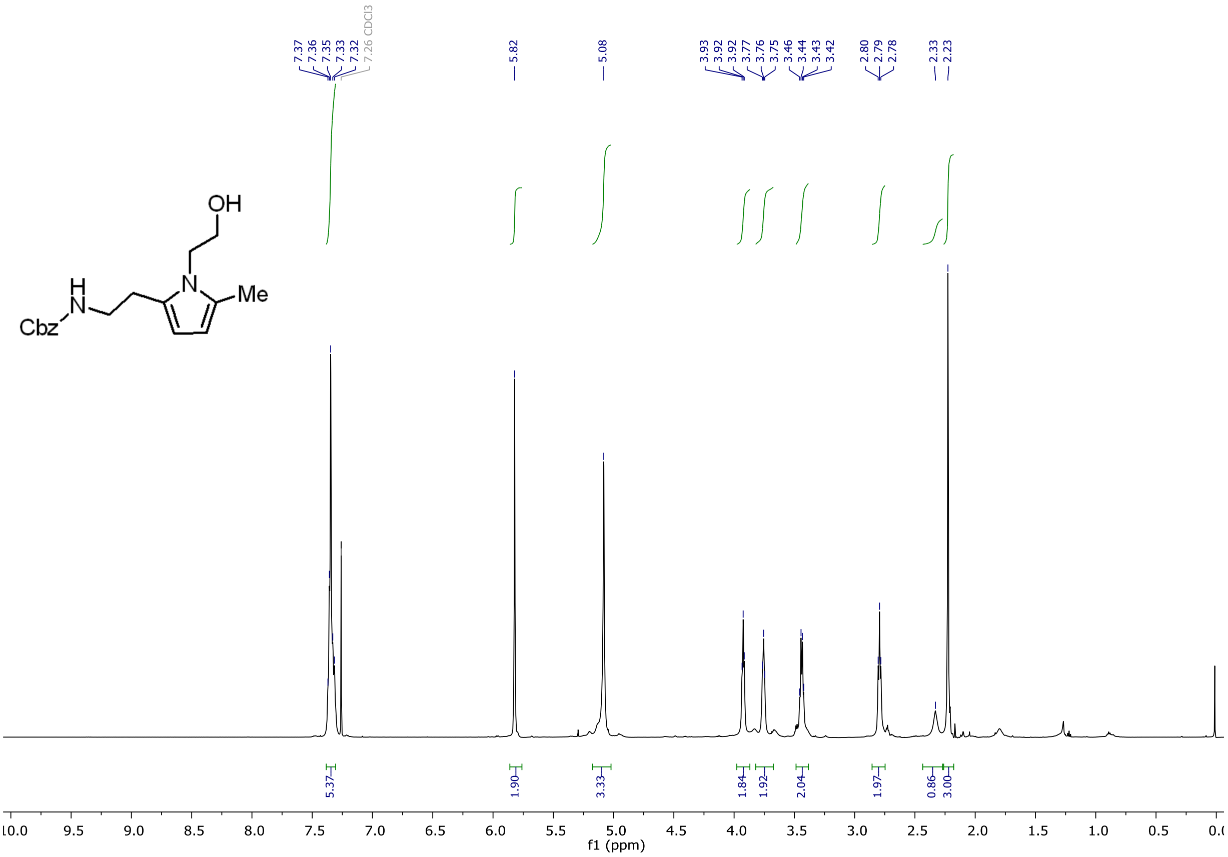

^{1}H NMR (600 MHz, CDCl$_3$) of ***benzyl (2-(1-(2-hydroxyethyl)-5-methyl-1H-pyrrol-2-yl)ethyl)carbamate (17)***

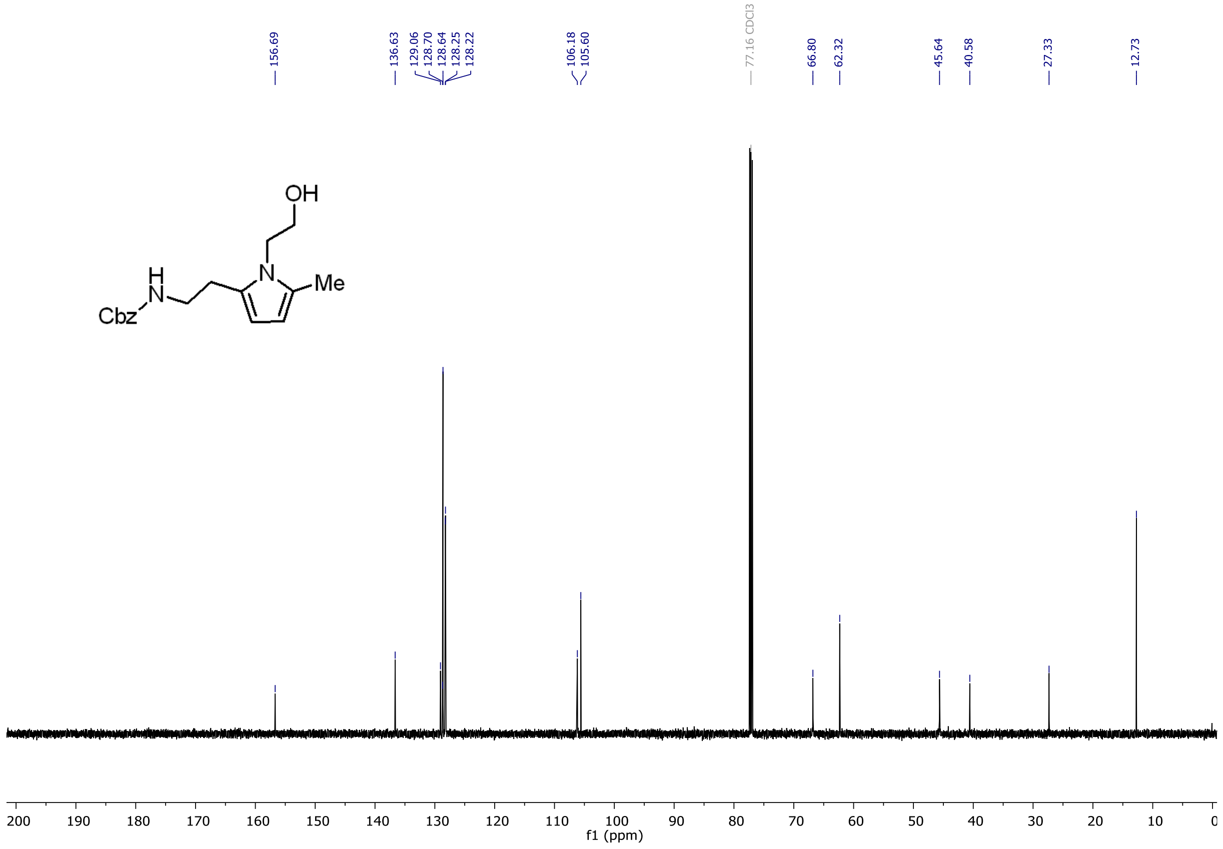

^{13}C NMR (151 MHz, CDCl$_3$) of ***benzyl (2-(1-(2-hydroxyethyl)-5-methyl-1H-pyrrol-2-yl)ethyl)carbamate (17)***

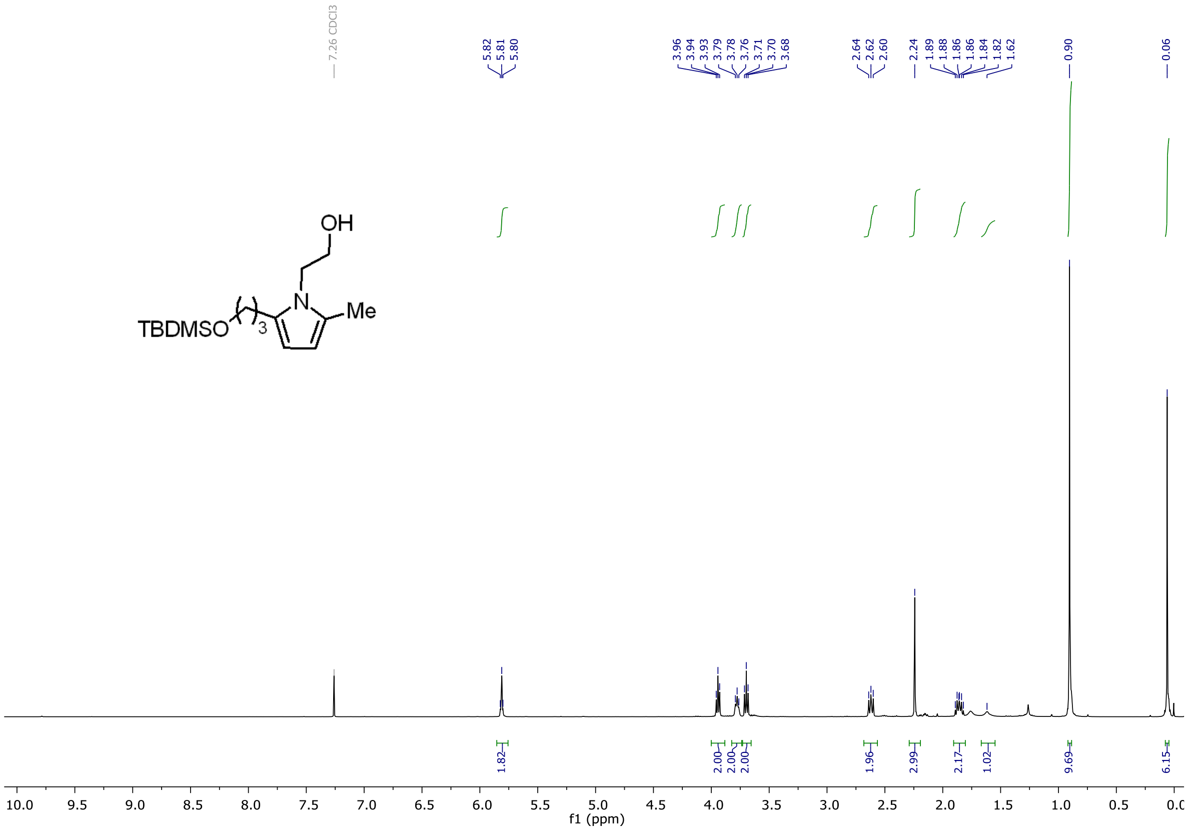

^{1}H NMR (400 MHz, $CDCl_3$) of ***2-(2-(((tert-butyldimethylsilyl)oxy)methyl)-5-methyl-1H-pyrrol-1-yl)ethan-1-ol (18)***

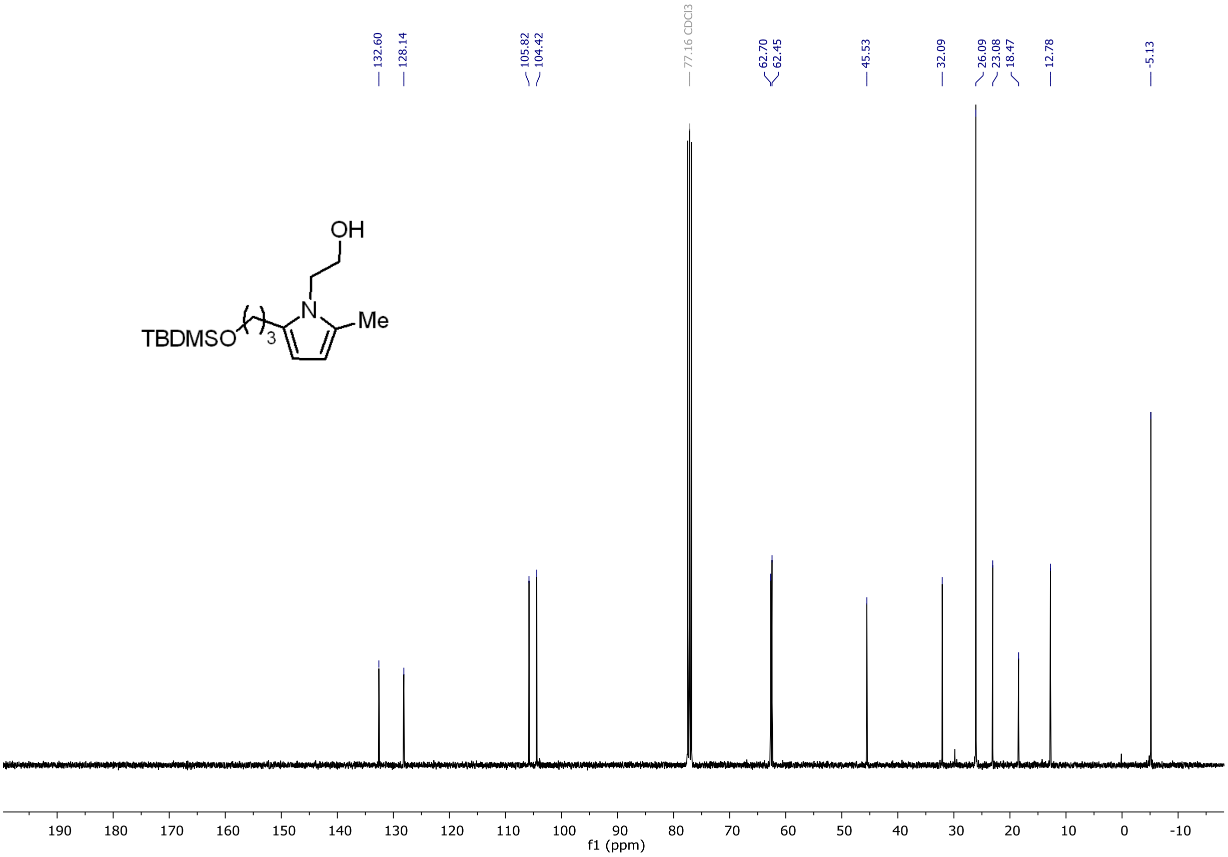

^{13}C NMR (101 MHz, $CDCl_3$) of ***2-(2-(((tert-butyldimethylsilyl)oxy)methyl)-5-methyl-1H-pyrrol-1-yl)ethan-1-ol (18)***

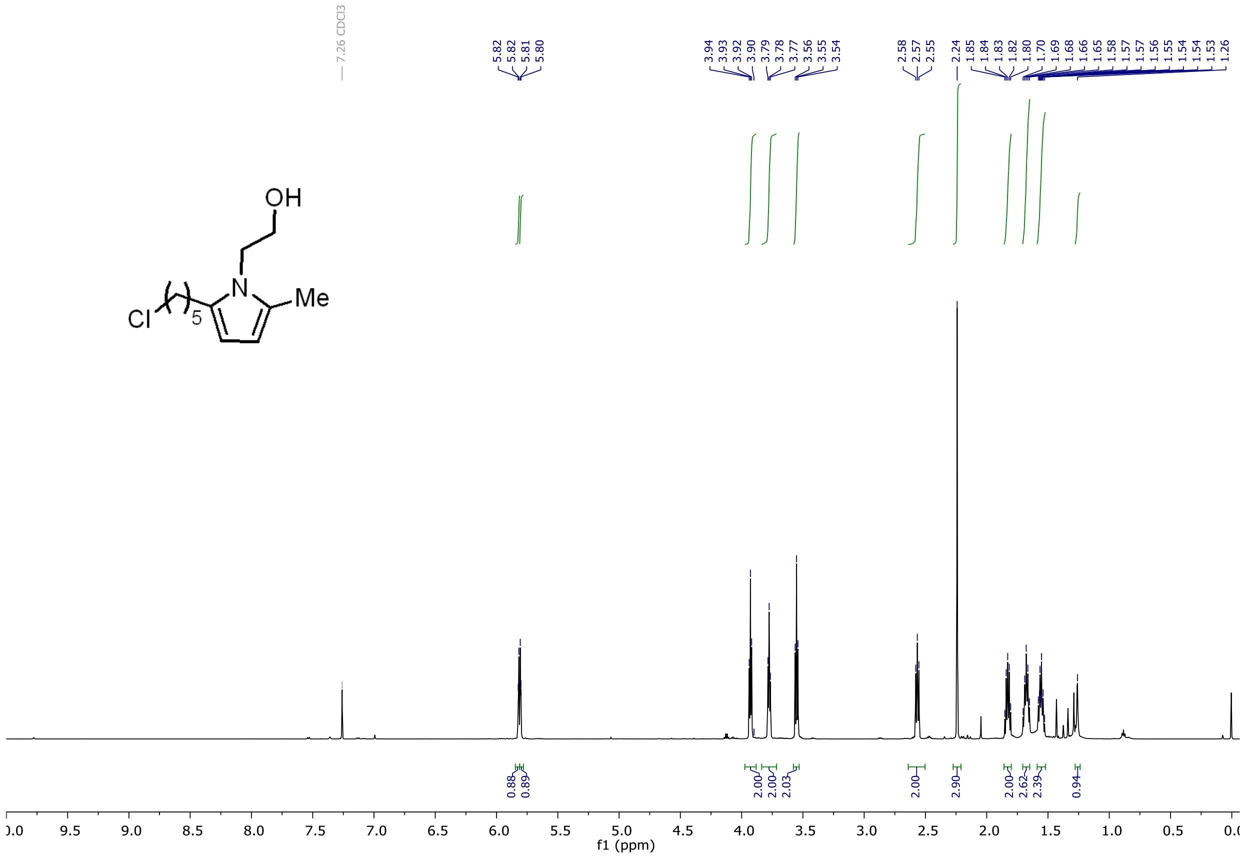

^{1}H NMR (600 MHz, $CDCl_3$) of ***2-(2-(5-chloropentyl)-5-methyl-1H-pyrrol-1-yl)ethan-1-ol (19)***

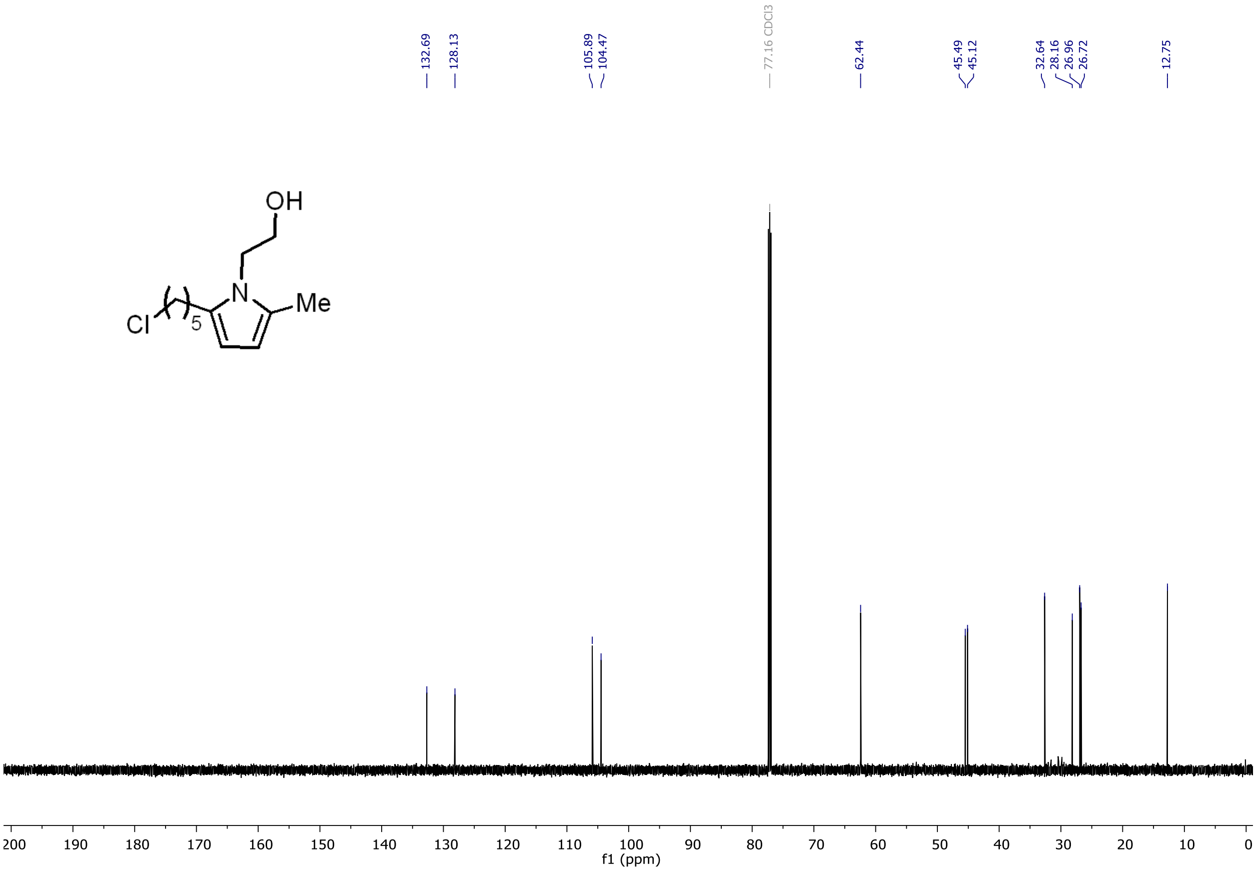

^{13}C NMR (151 MHz, $CDCl_3$) of ***2-(2-(5-chloropentyl)-5-methyl-1H-pyrrol-1-yl)ethan-1-ol (19)***

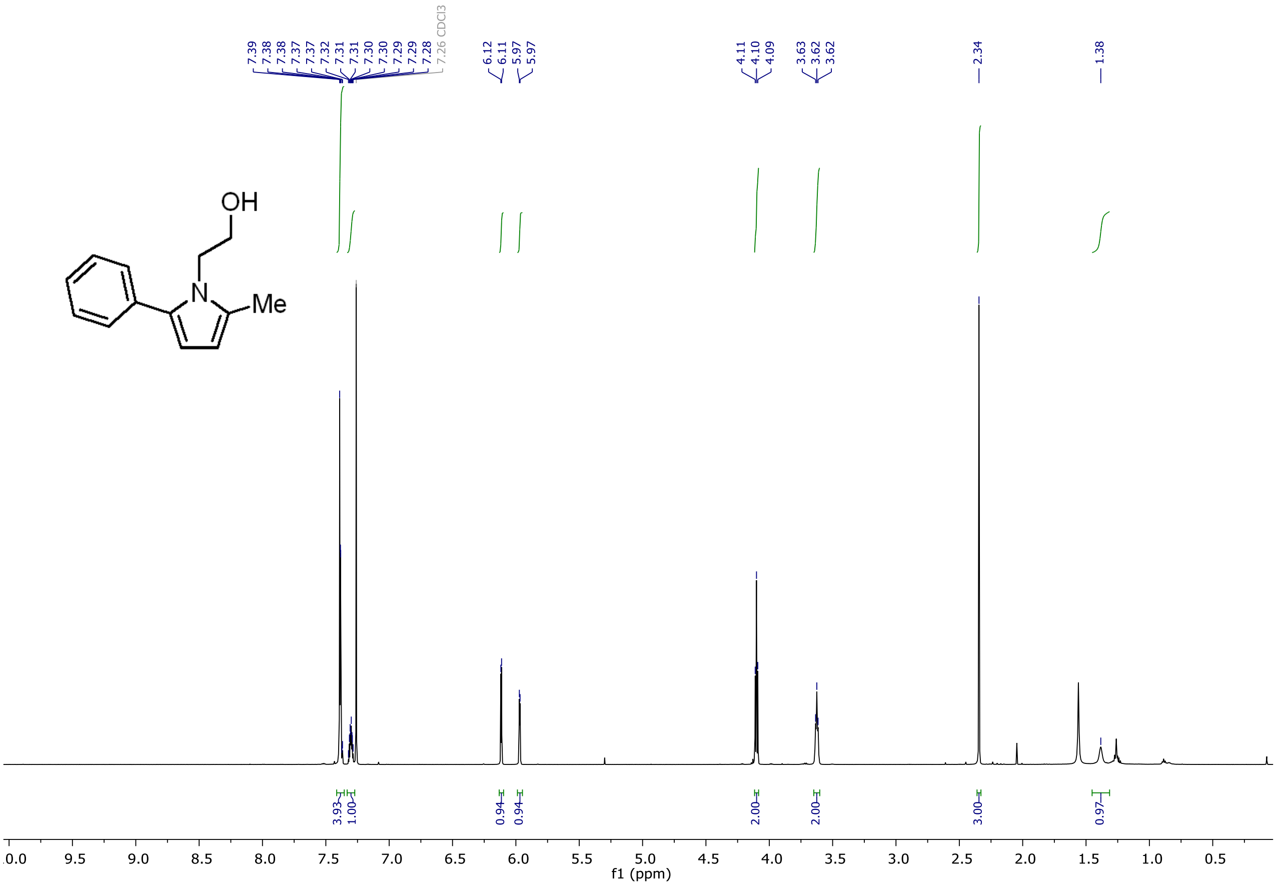

^{1}H NMR (600 MHz, $CDCl_3$) of ***2-(2-methyl-5-phenyl-1H-pyrrol-1-yl)ethan-1-ol (20)***

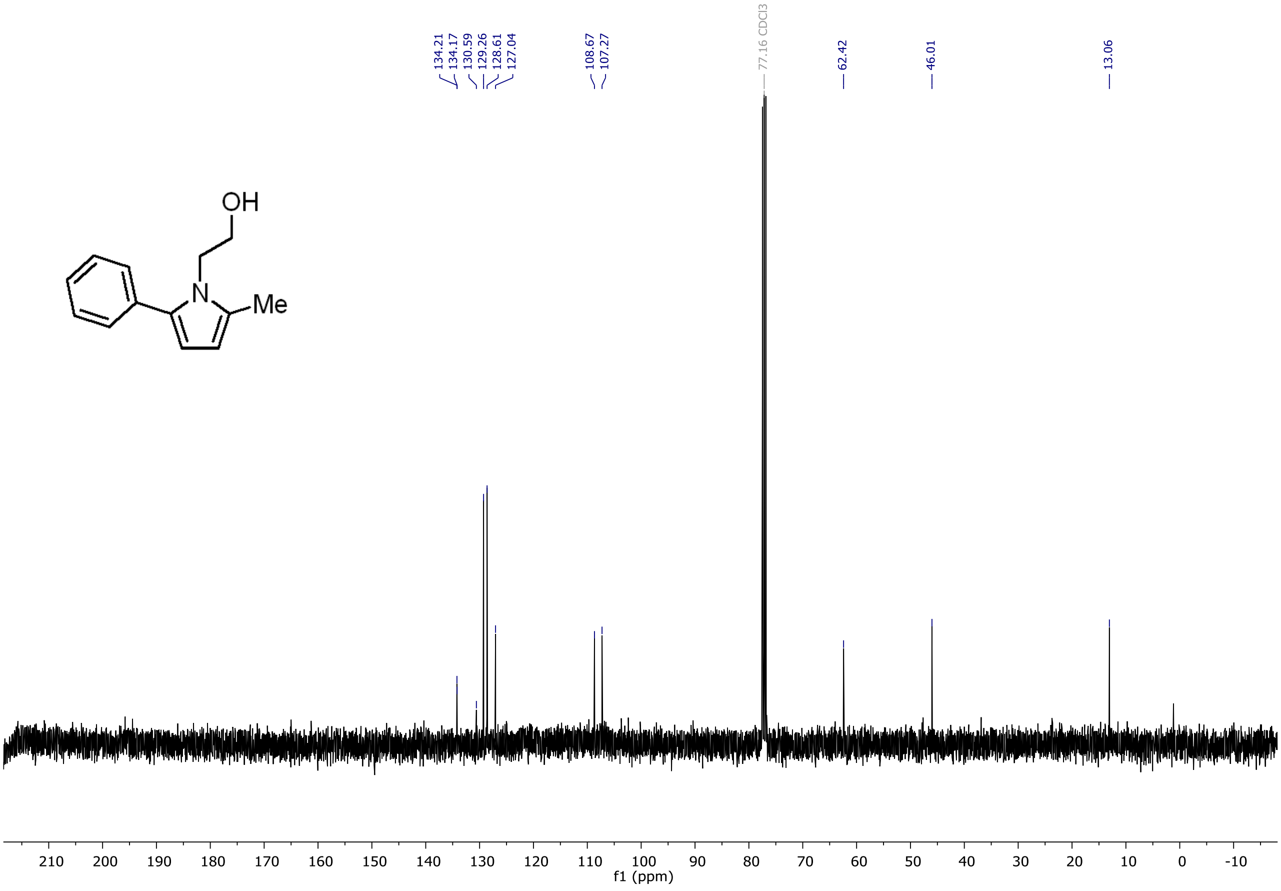

^{13}C NMR (101 MHz, $CDCl_3$) of ***2-(2-methyl-5-phenyl-1H-pyrrol-1-yl)ethan-1-ol (20)***

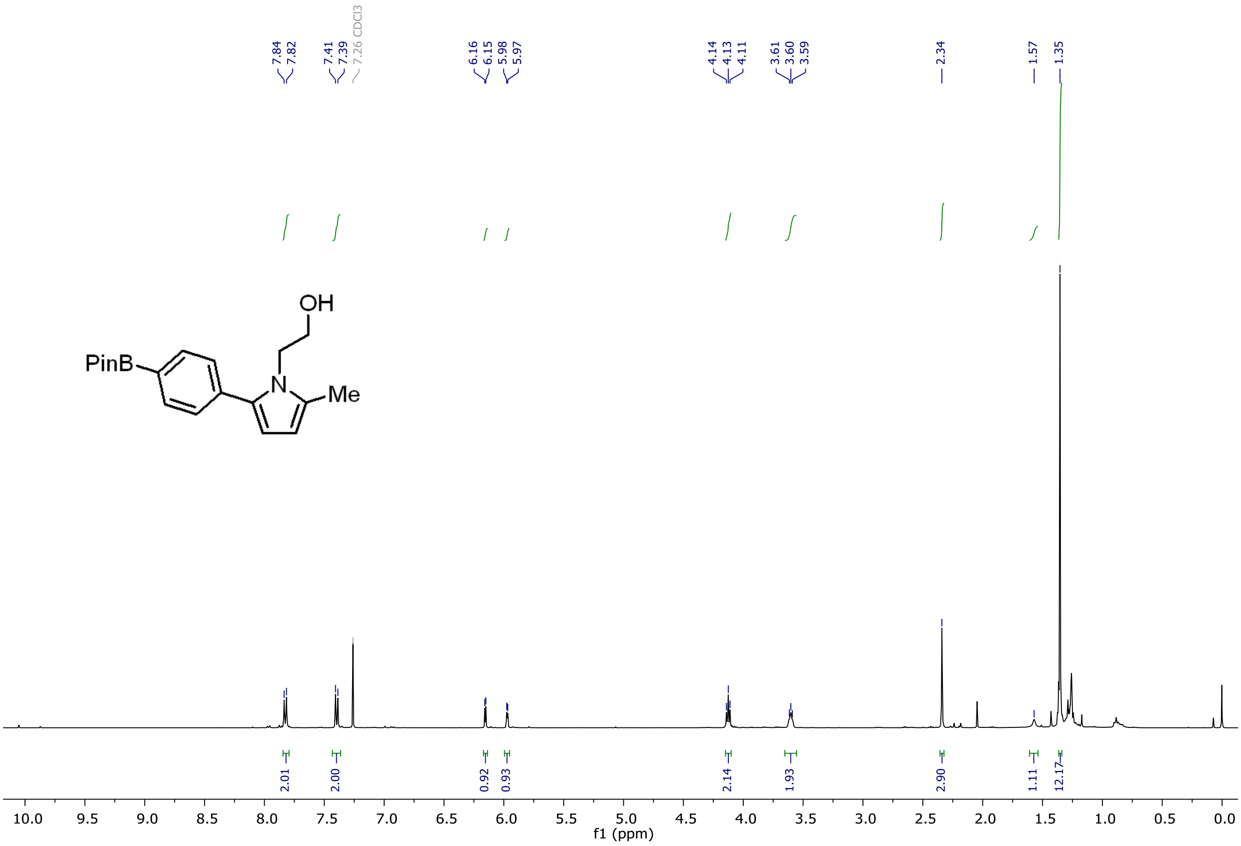

^{1}H NMR (400 MHz, $CDCl_3$) of ***2-(2-methyl-5-(4-(4,4,5,5-tetramethyl-1,3,2-dioxaborolan-2-yl)phenyl)-1H-pyrrol-1-yl)ethan-1-ol (21)***

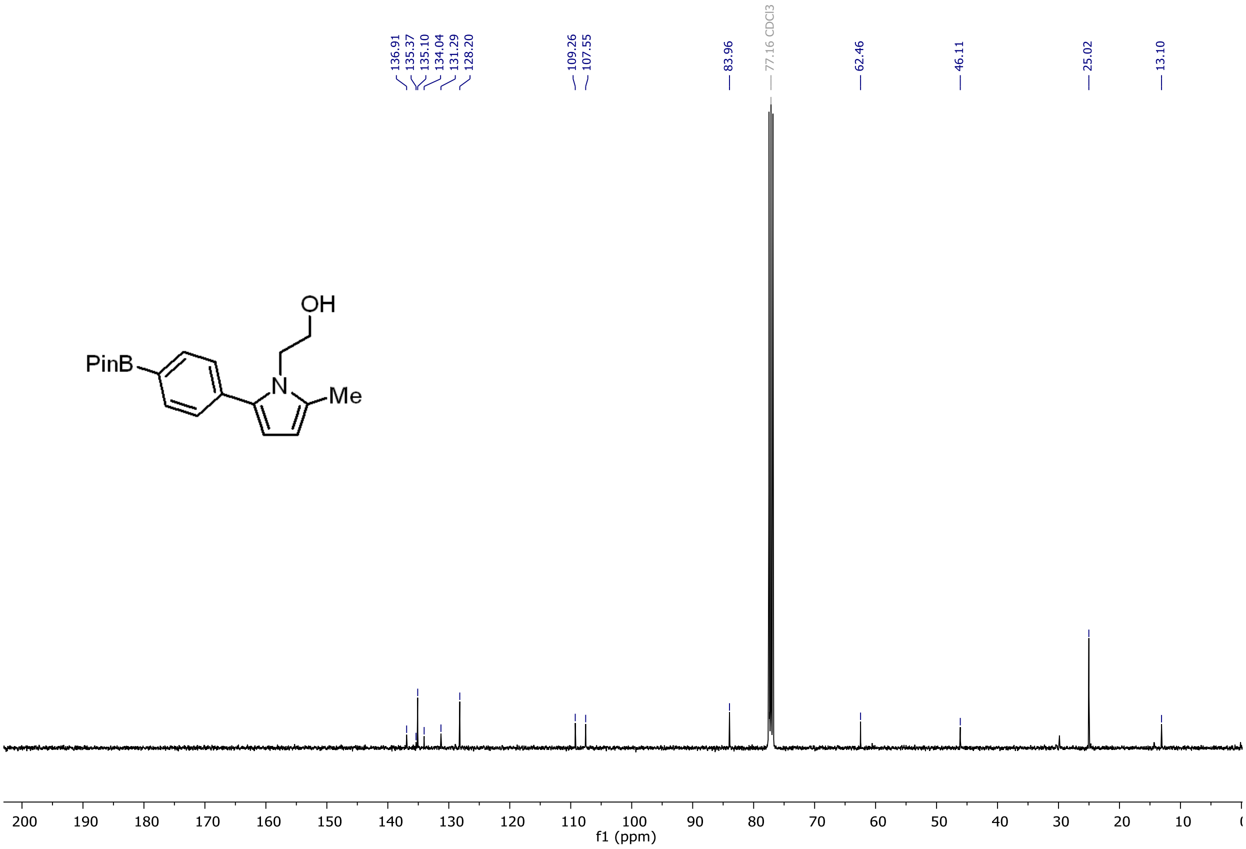

^{13}C NMR (101 MHz, $CDCl_3$) of ***2-(2-methyl-5-(4-(4,4,5,5-tetramethyl-1,3,2-dioxaborolan-2-yl)phenyl)-1H-pyrrol-1-yl)ethan-1-ol (21)***

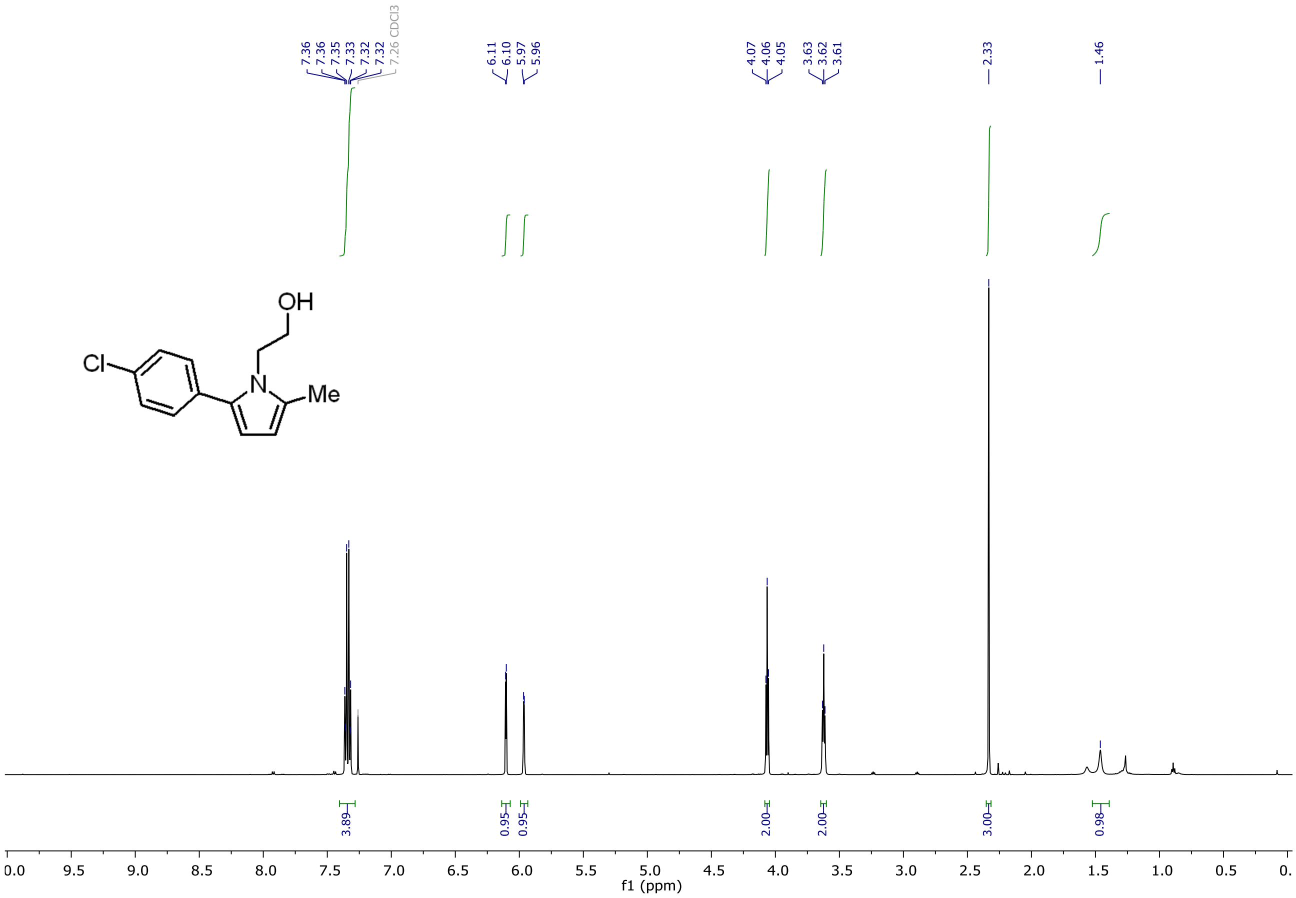

^{1}H NMR (600 MHz, CDCl$_3$) of ***2-(2-(4-chlorophenyl)-5-methyl-1H-pyrrol-1-yl)ethan-1-ol (22)***

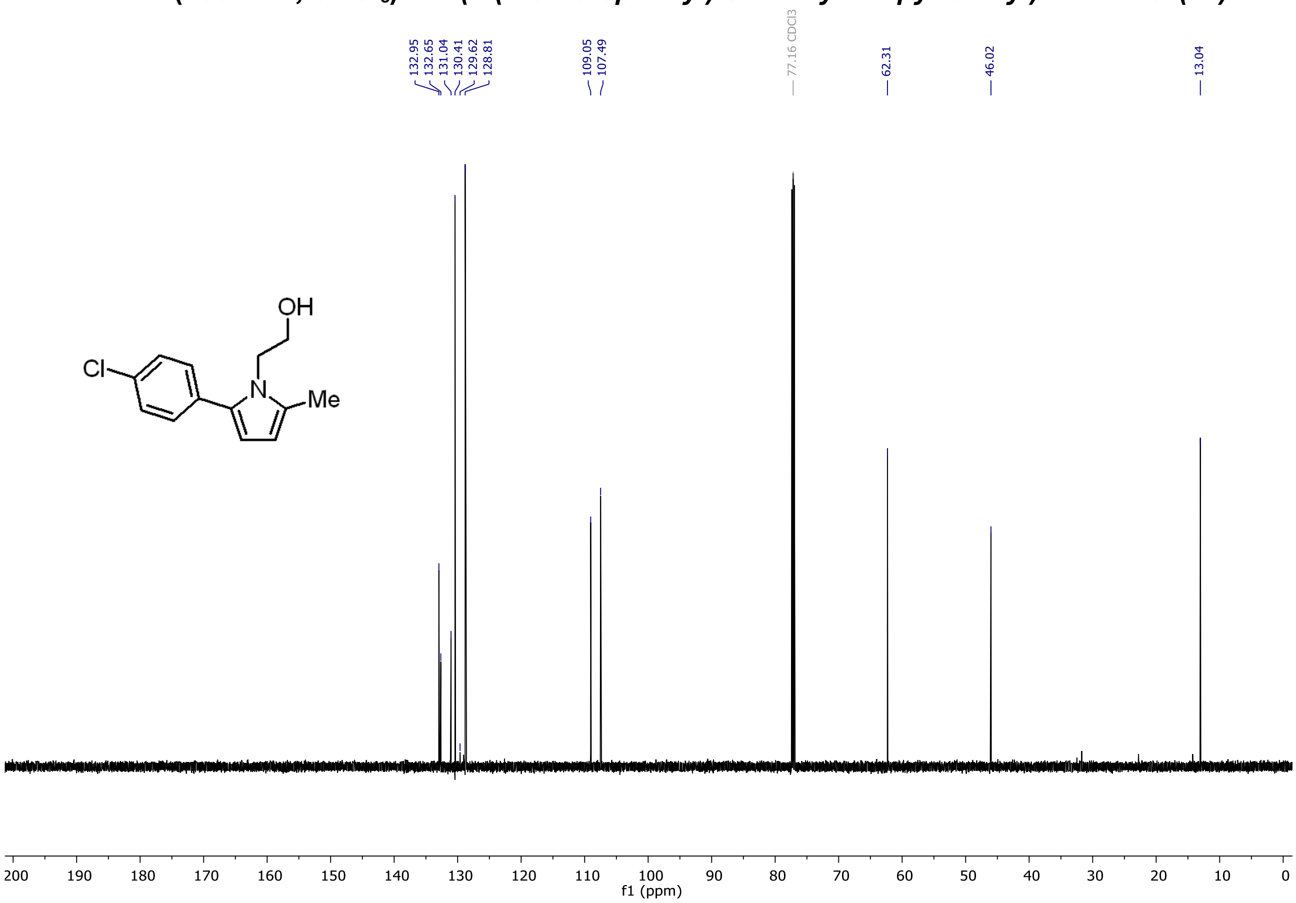

^{13}C NMR (151 MHz, CDCl$_3$) of ***2-(2-(4-chlorophenyl)-5-methyl-1H-pyrrol-1-yl)ethan-1-ol (22)***

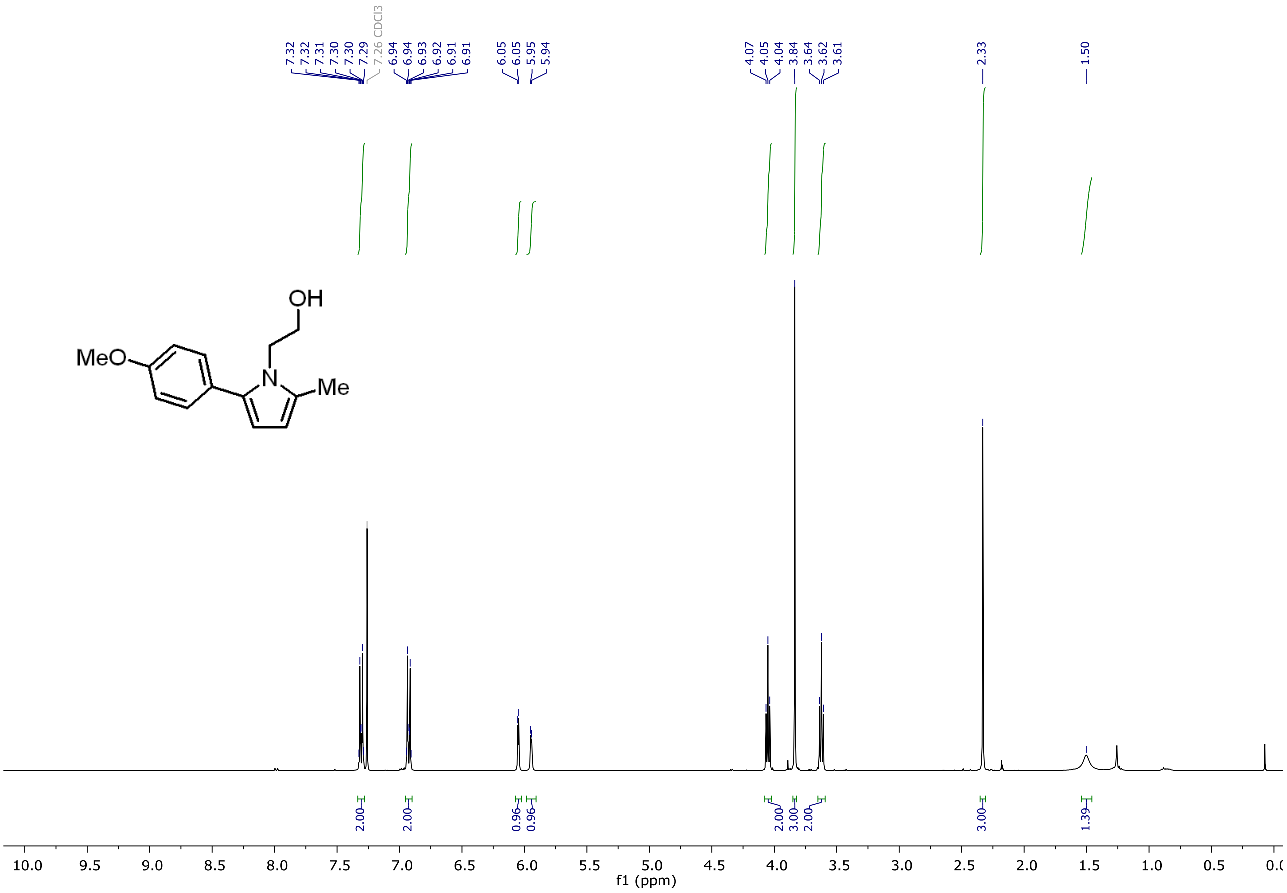

^{1}H NMR (400 MHz, $CDCl_3$) of *2-(2-(4-methoxyphenyl)-5-methyl-1H-pyrrol-1-yl)ethan-1-ol (23)*

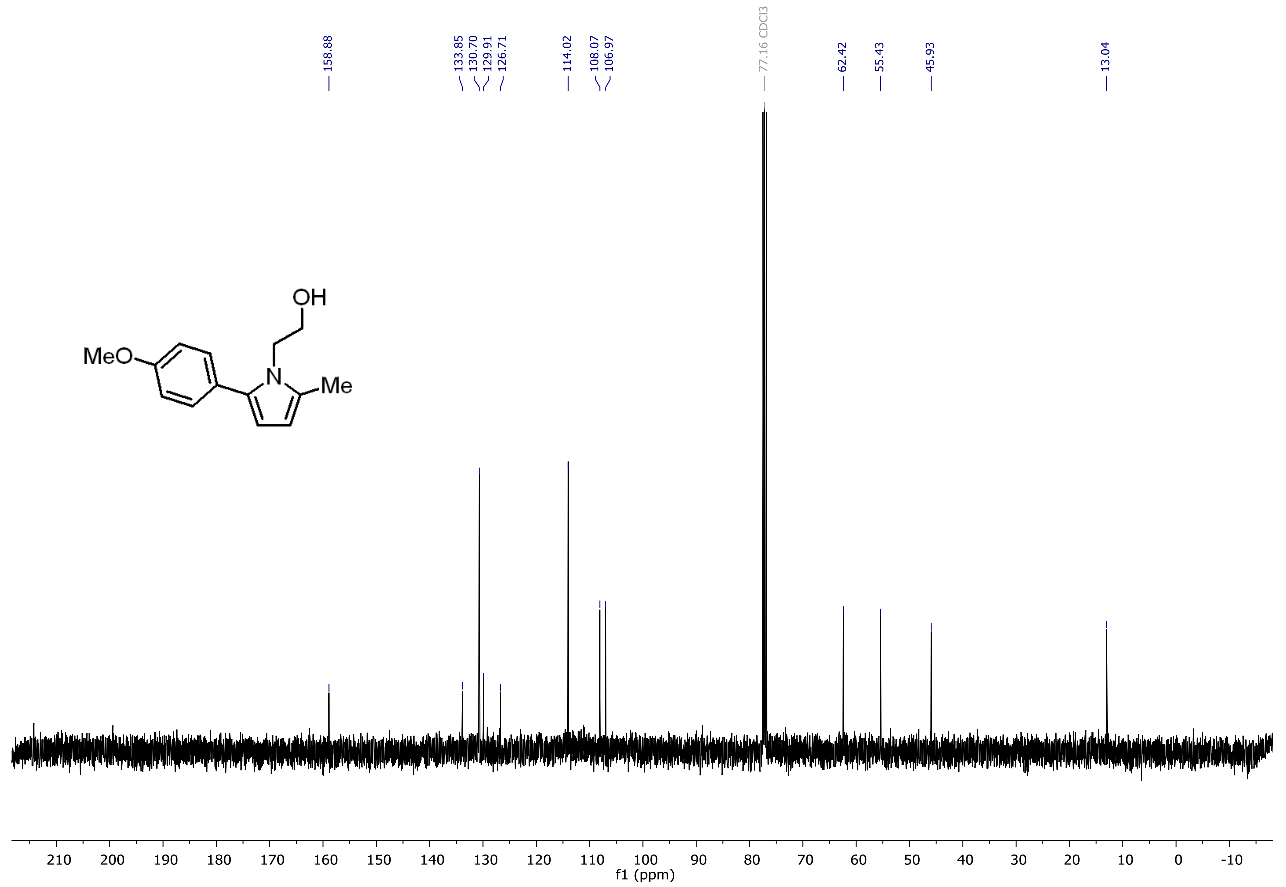

^{13}C NMR (101 MHz, $CDCl_3$) of *2-(2-(4-methoxyphenyl)-5-methyl-1H-pyrrol-1-yl)ethan-1-ol (23)*

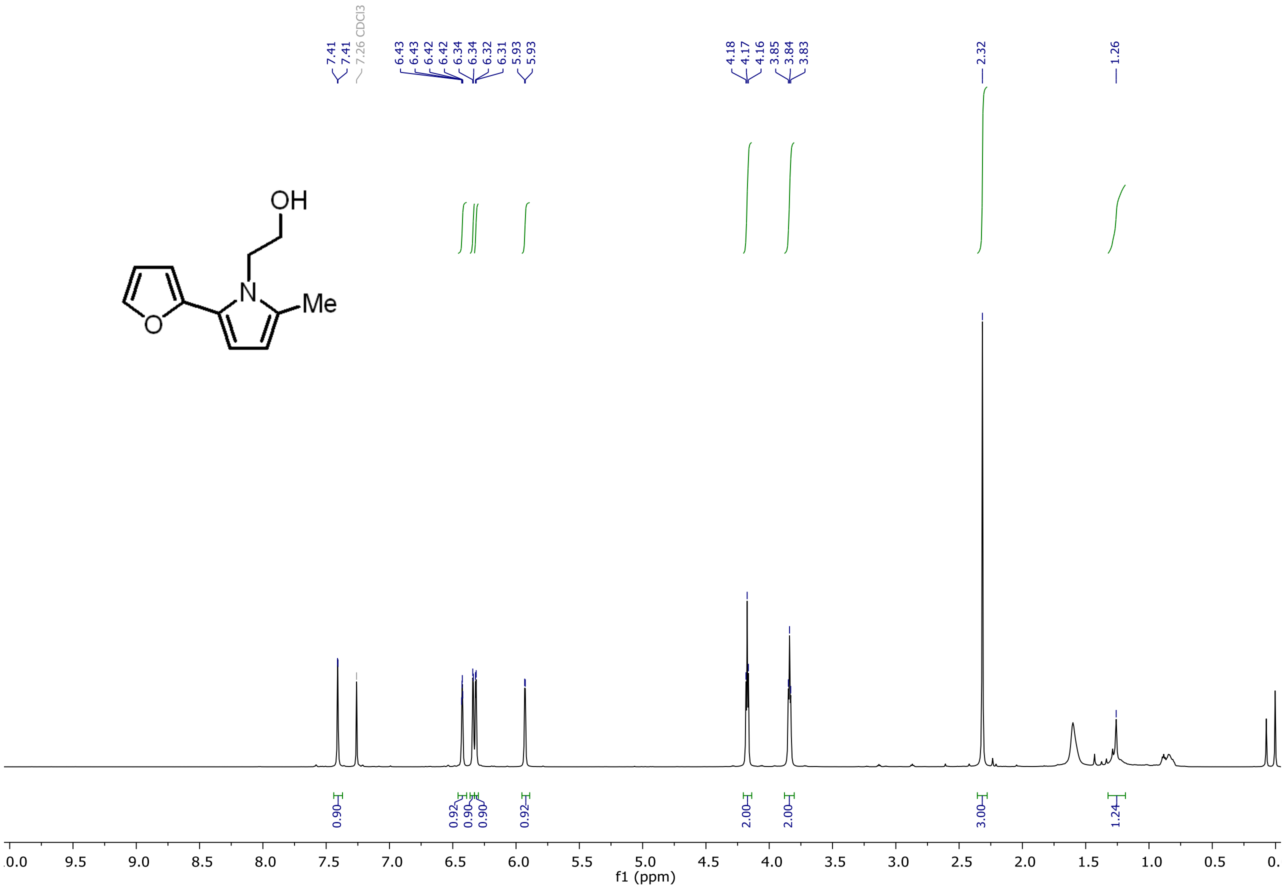

^{1}H NMR (600 MHz, $CDCl_3$) of ***2-(2-(furan-2-yl)-5-methyl-1H-pyrrol-1-yl)ethan-1-ol (24)***

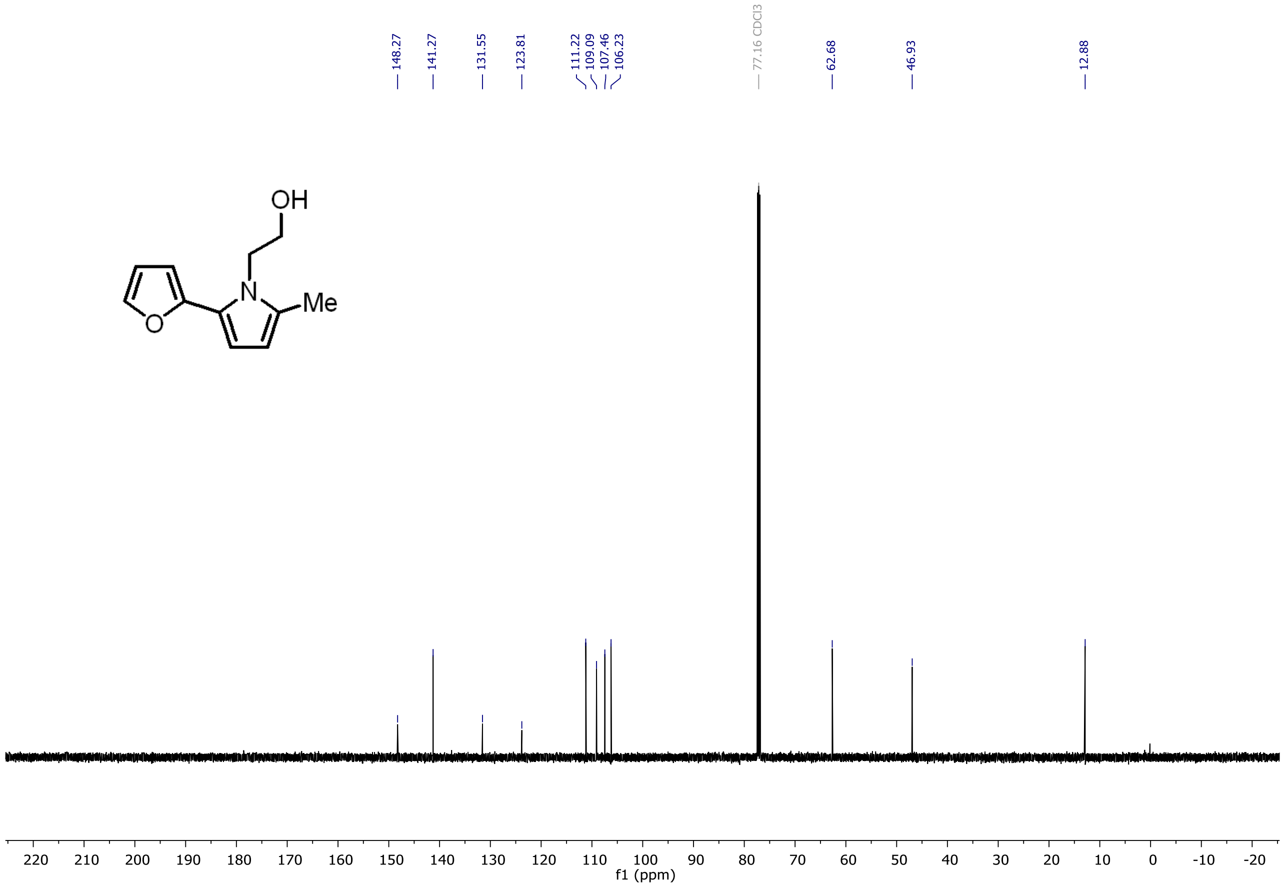

^{13}C NMR (151 MHz, $CDCl_3$) of ***2-(2-(furan-2-yl)-5-methyl-1H-pyrrol-1-yl)ethan-1-ol (24)***

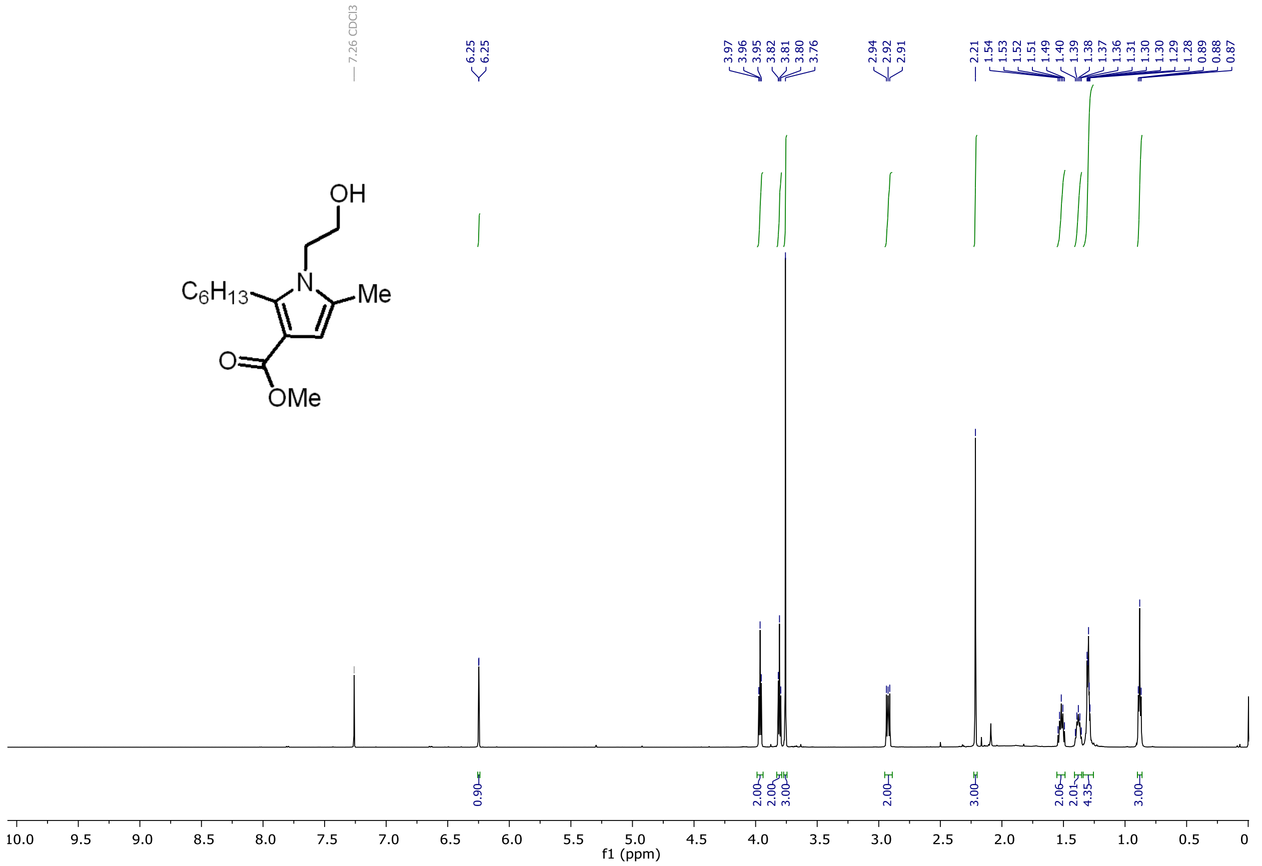

^{1}H NMR (600 MHz, $CDCl_3$) of ***methyl 2-hexyl-1-(2-hydroxyethyl)-5-methyl-1H-pyrrole-3-carboxylate (25)***

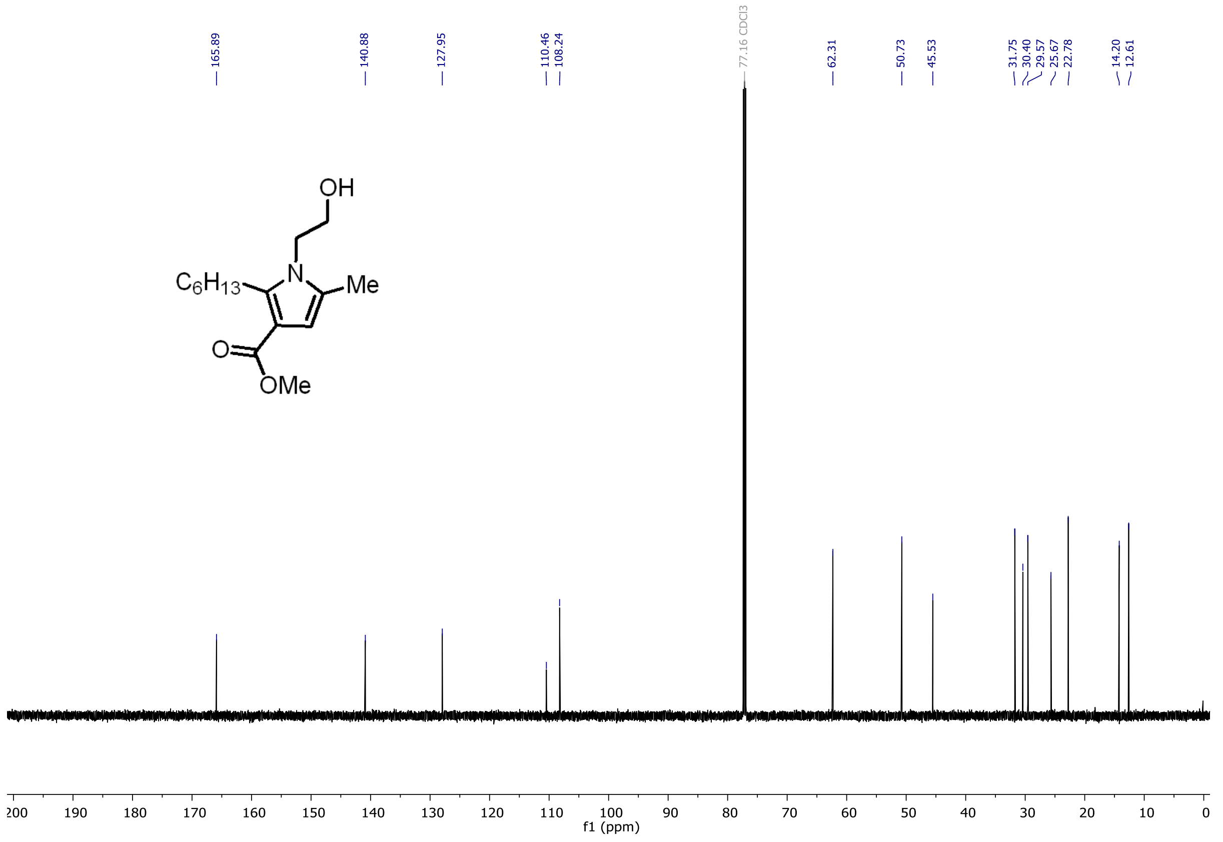

^{13}C NMR (151 MHz, $CDCl_3$) of ***methyl 2-hexyl-1-(2-hydroxyethyl)-5-methyl-1H-pyrrole-3-carboxylate (25)***

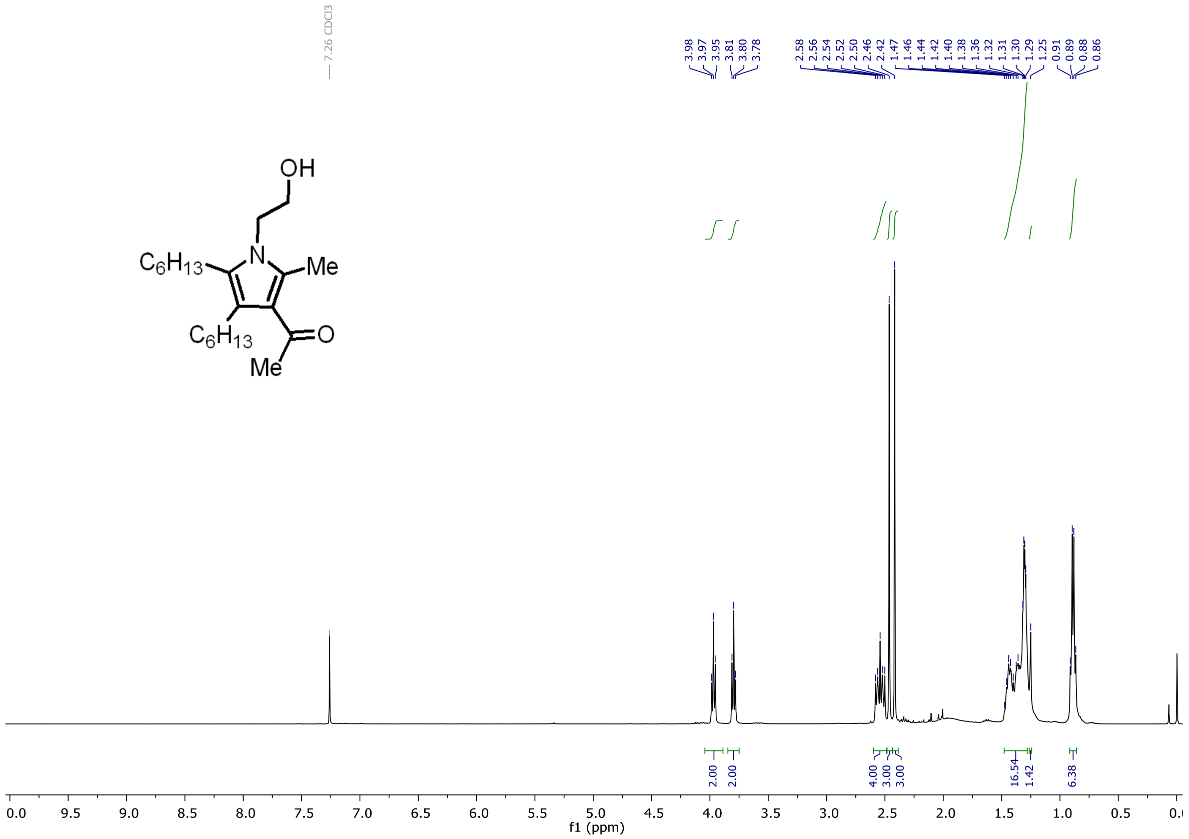

^{1}H NMR (400 MHz, CDCl$_3$) of ***1-(4,5-dihexyl-1-(2-hydroxyethyl)-2-methyl-1H-pyrrol-3-yl)ethan-1-one (26)***

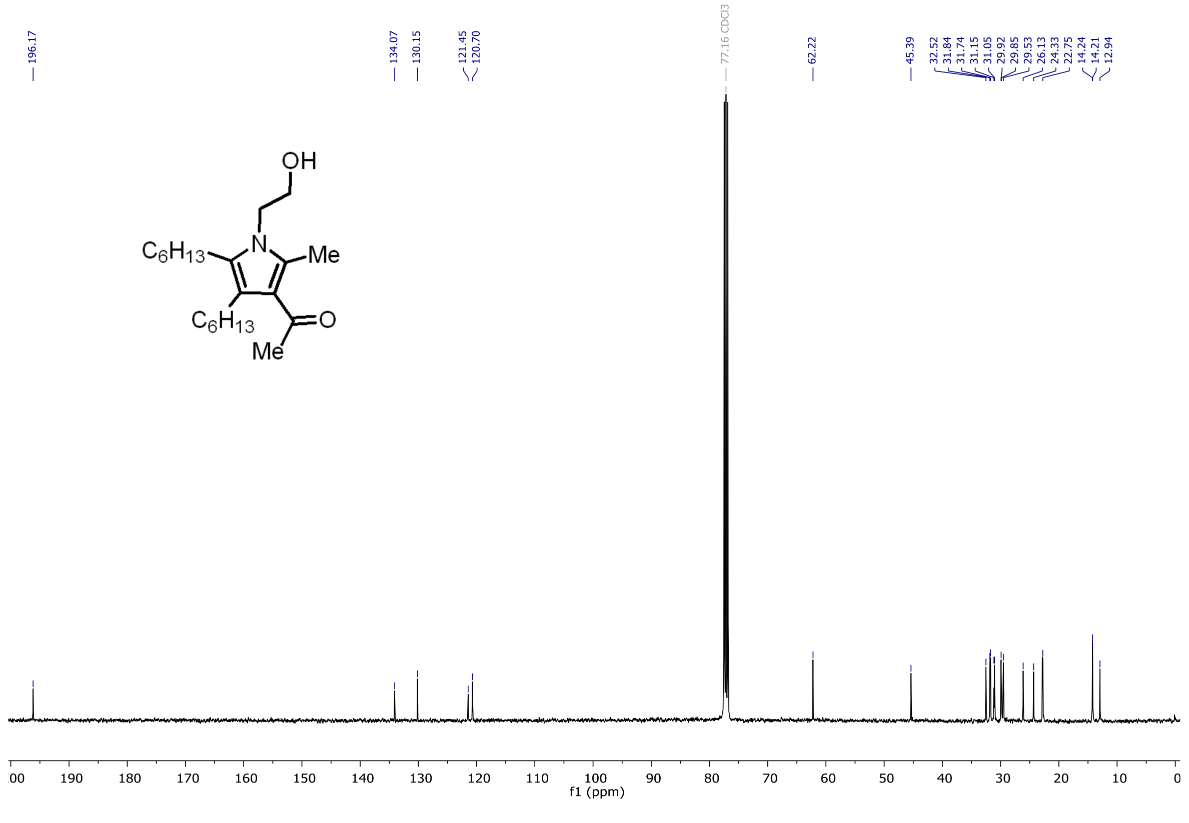

^{13}C NMR (101 MHz, CDCl$_3$) of ***1-(4,5-dihexyl-1-(2-hydroxyethyl)-2-methyl-1H-pyrrol-3-yl)ethan-1-one (26)***

10.3 Thiophenes

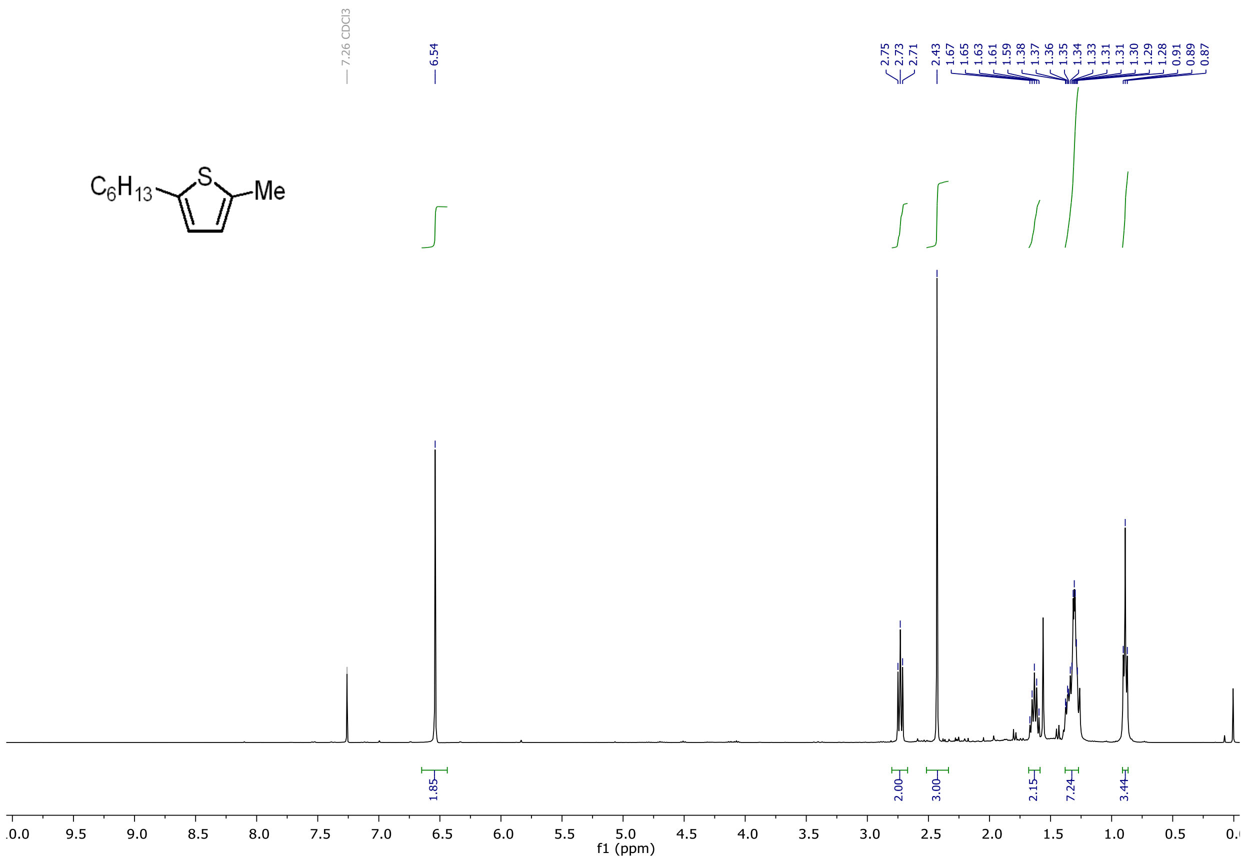

^{1}H NMR (400 MHz, $CDCl_3$) of ***2-hexyl-5-methylthiophene (27)***

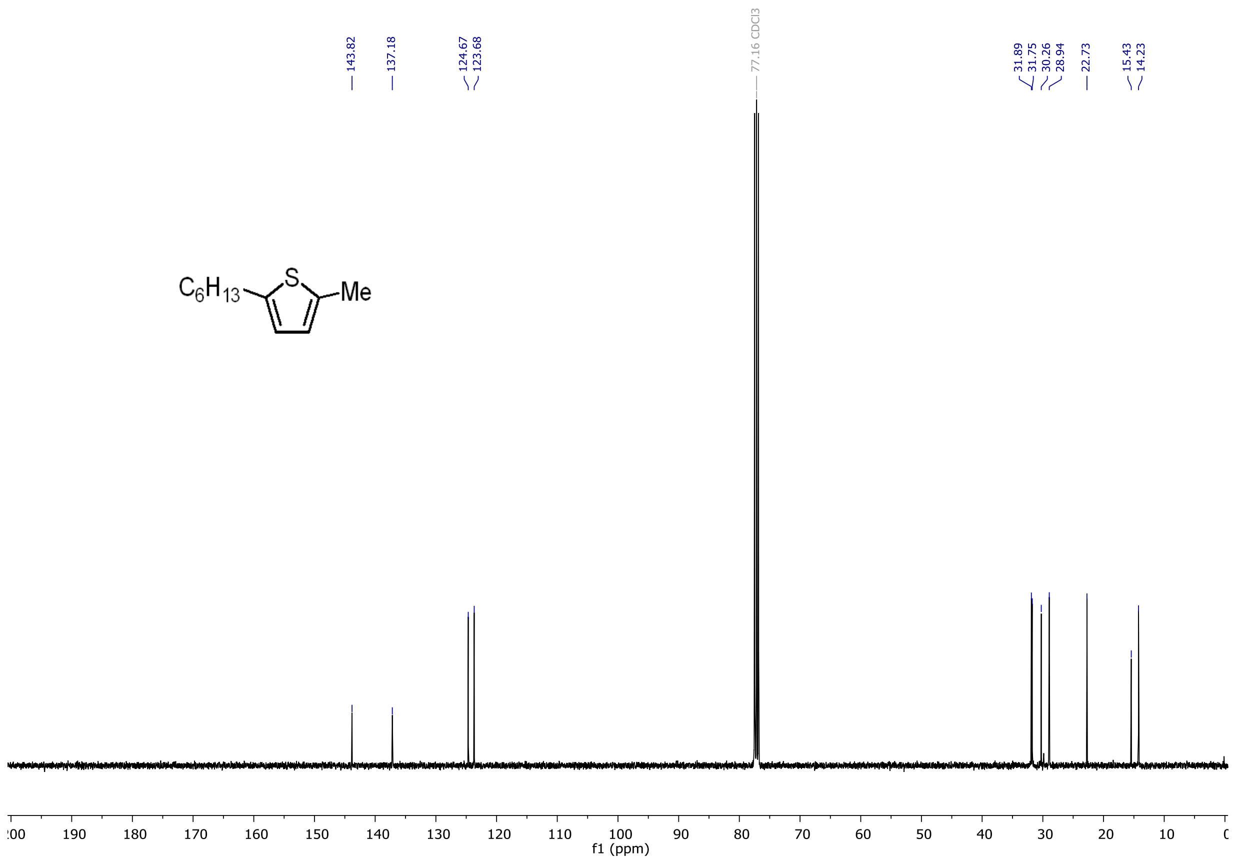

^{13}C NMR (101 MHz, $CDCl_3$) of ***2-hexyl-5-methylthiophene (27)***

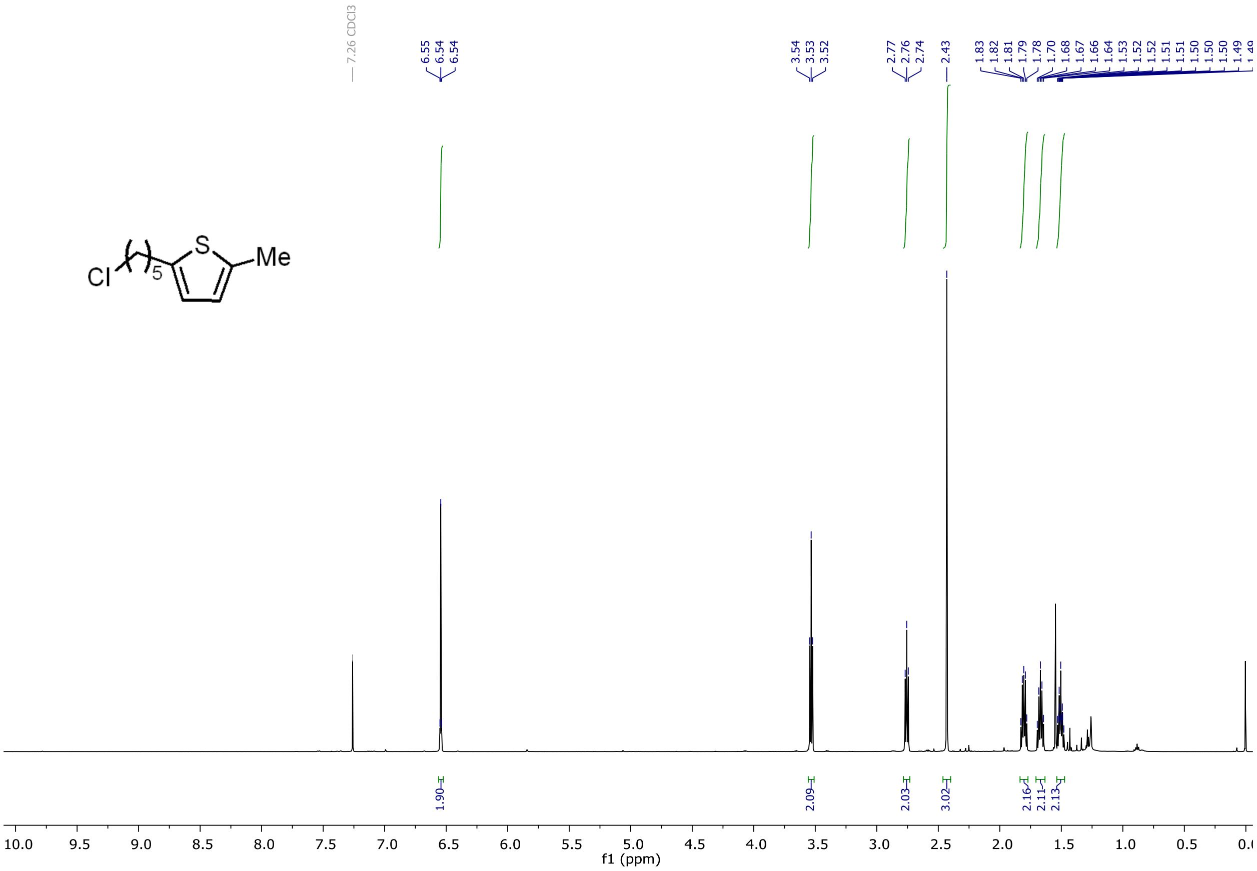

^{1}H NMR (600 MHz, $CDCl_3$) of ***2-(5-chloropentyl)-5-methylthiophene (28)***

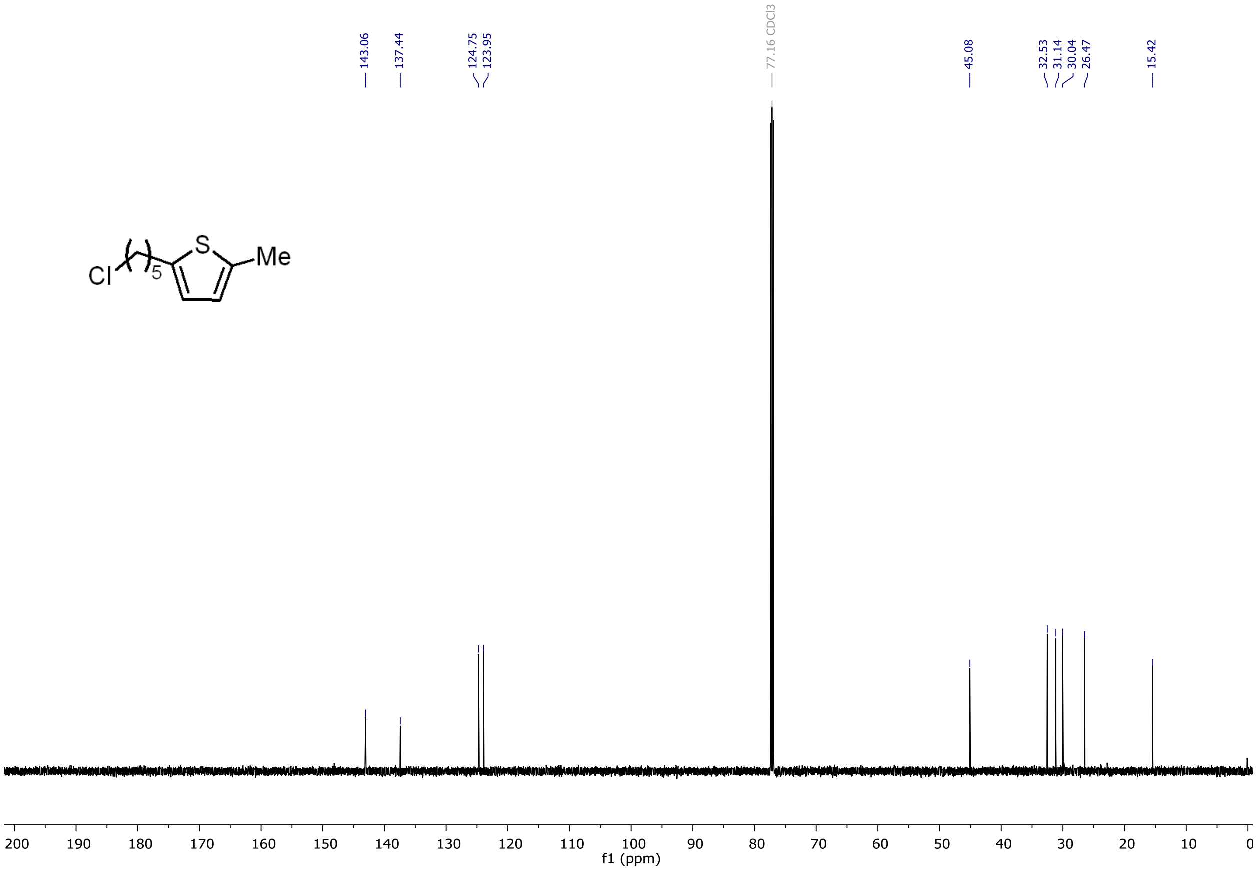

^{13}C NMR (151 MHz, $CDCl_3$) of ***2-(5-chloropentyl)-5-methylthiophene (28)***

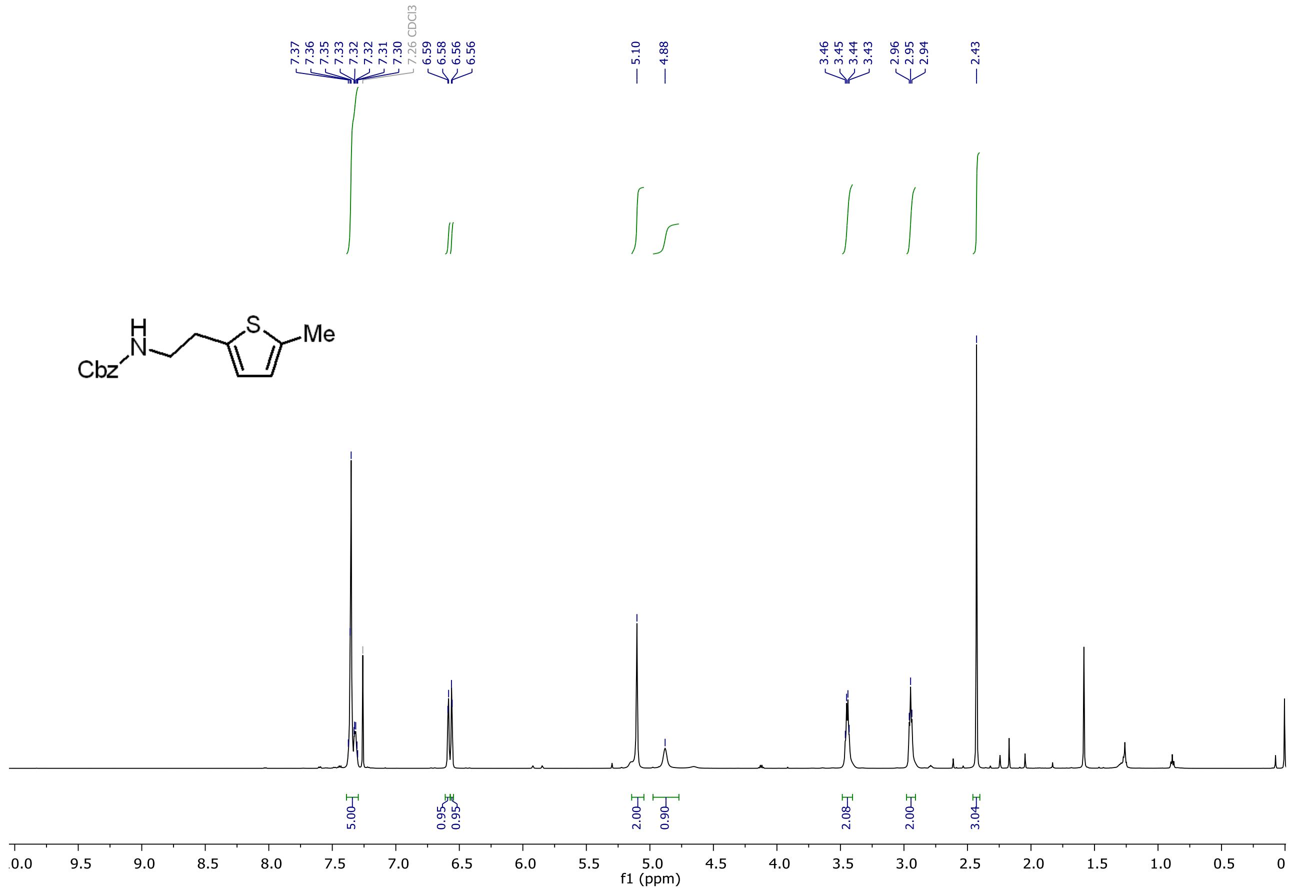

^{1}H NMR (600 MHz, CDCl$_3$) of ***benzyl (2-(5-methylthiophen-2-yl)ethyl)carbamate (29)***

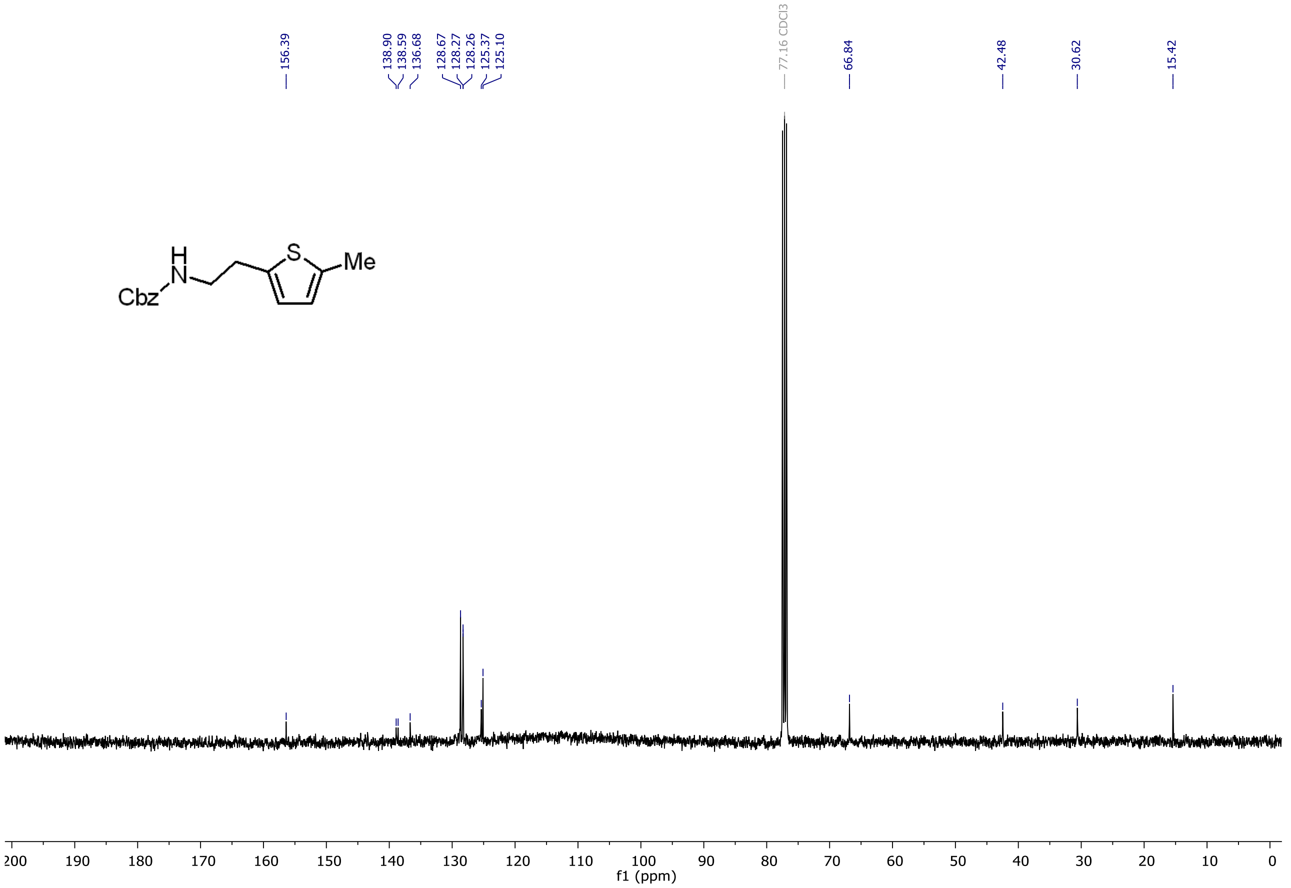

^{13}C NMR (101 MHz, CDCl$_3$) of ***benzyl (2-(5-methylthiophen-2-yl)ethyl)carbamate (29)***

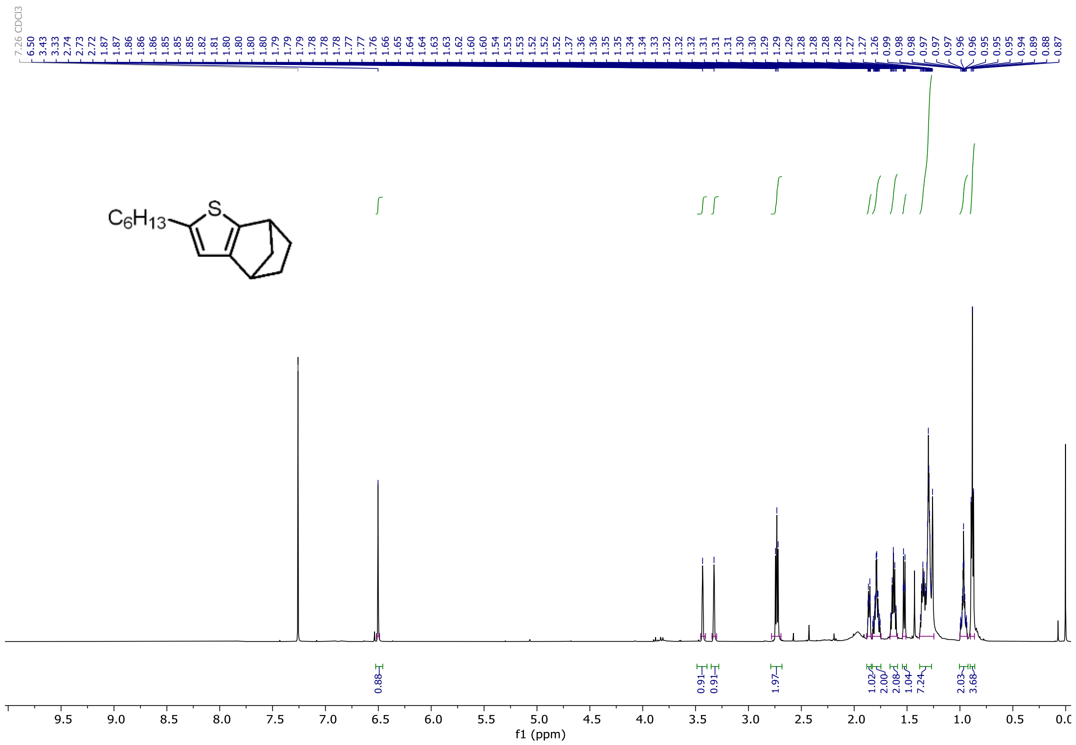

^{1}H NMR (600 MHz, $CDCl_3$) of ***2-hexyl-4,5,6,7-tetrahydro-4,7-methanobenzo[b]thiophene (30)***

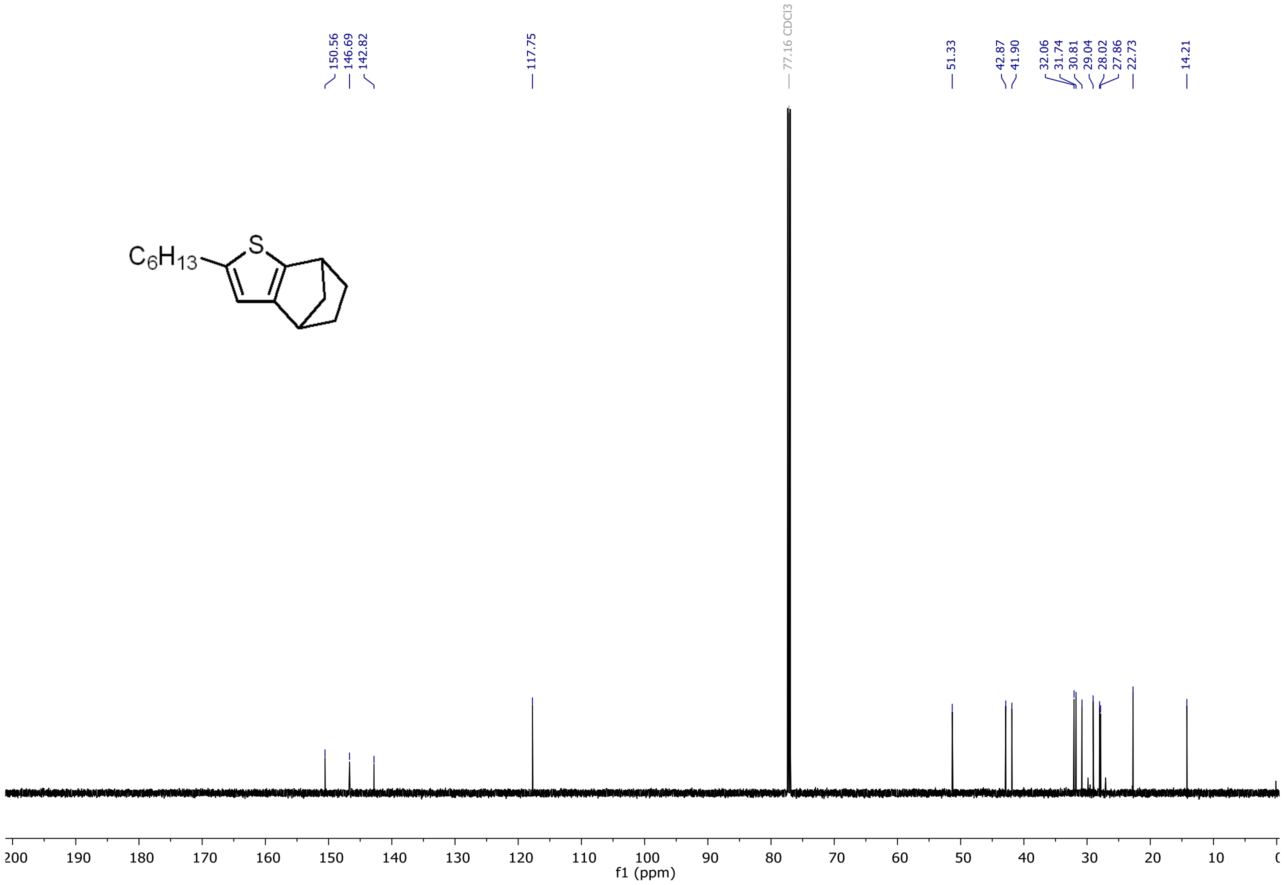

^{13}C NMR (151 MHz, $CDCl_3$) of ***2-hexyl-4,5,6,7-tetrahydro-4,7-methanobenzo[b]thiophene (30)***

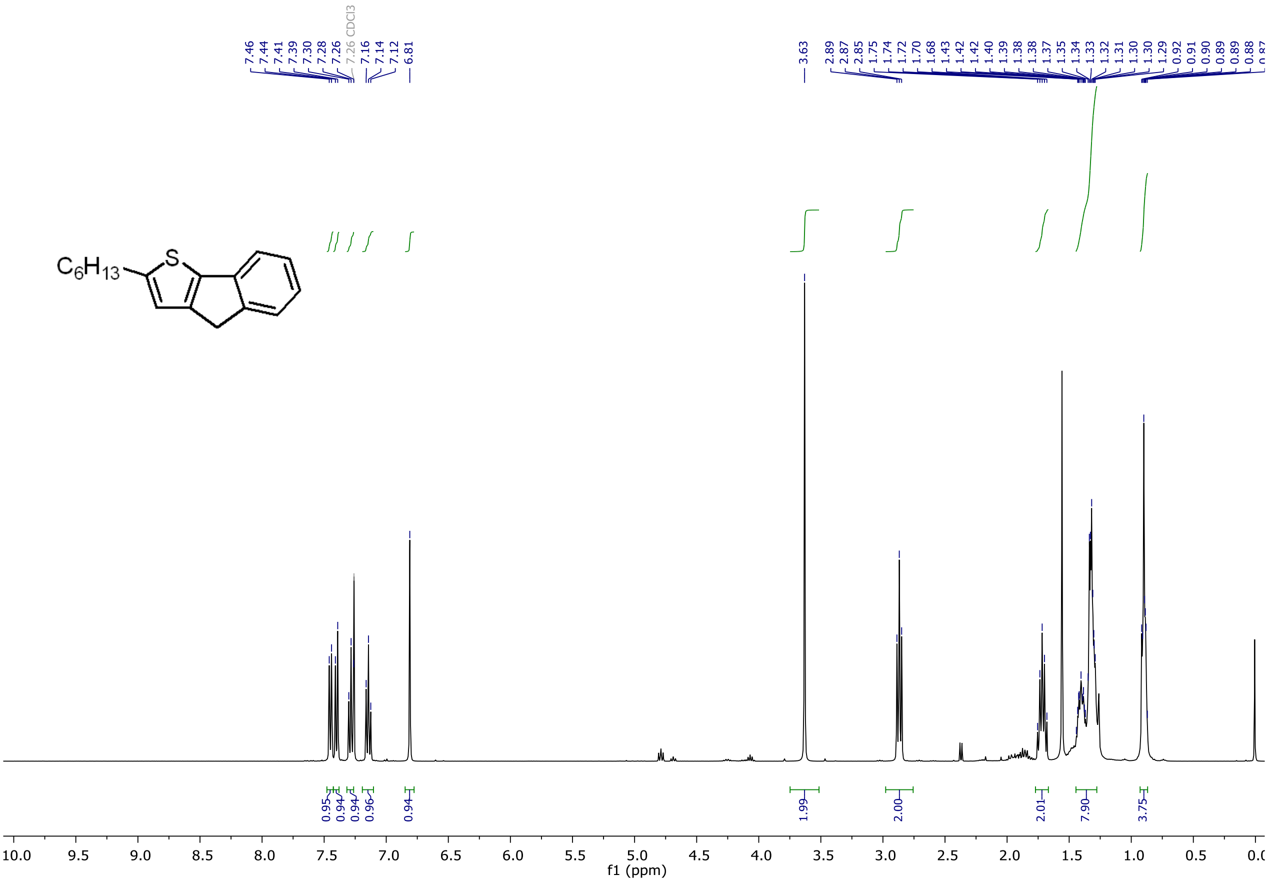

^{1}H NMR (400 MHz, CDCl$_3$) of ***2-hexyl-4H-indeno[1,2-b]thiophene (31)***

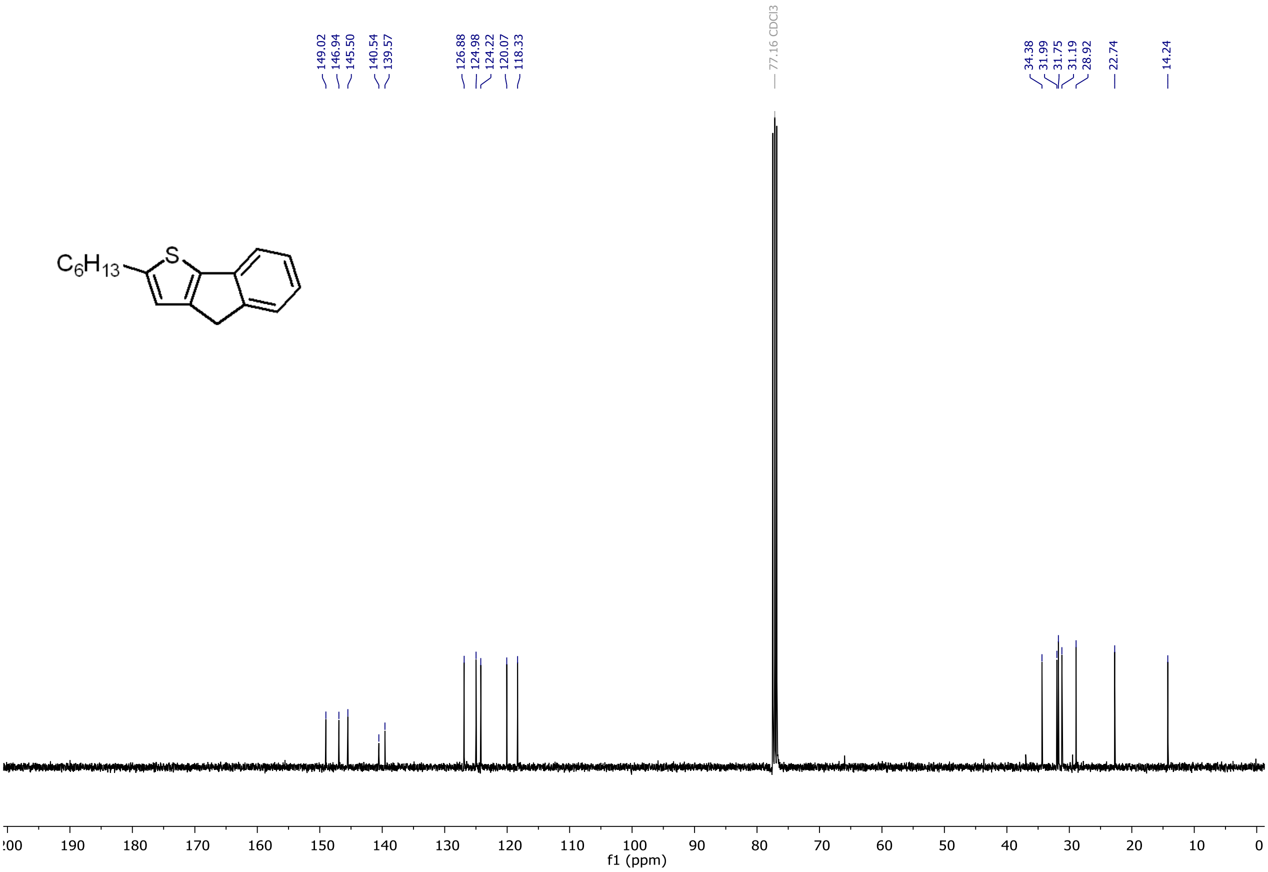

^{13}C NMR (101 MHz, CDCl$_3$) of ***2-hexyl-4H-indeno[1,2-b]thiophene (31)***

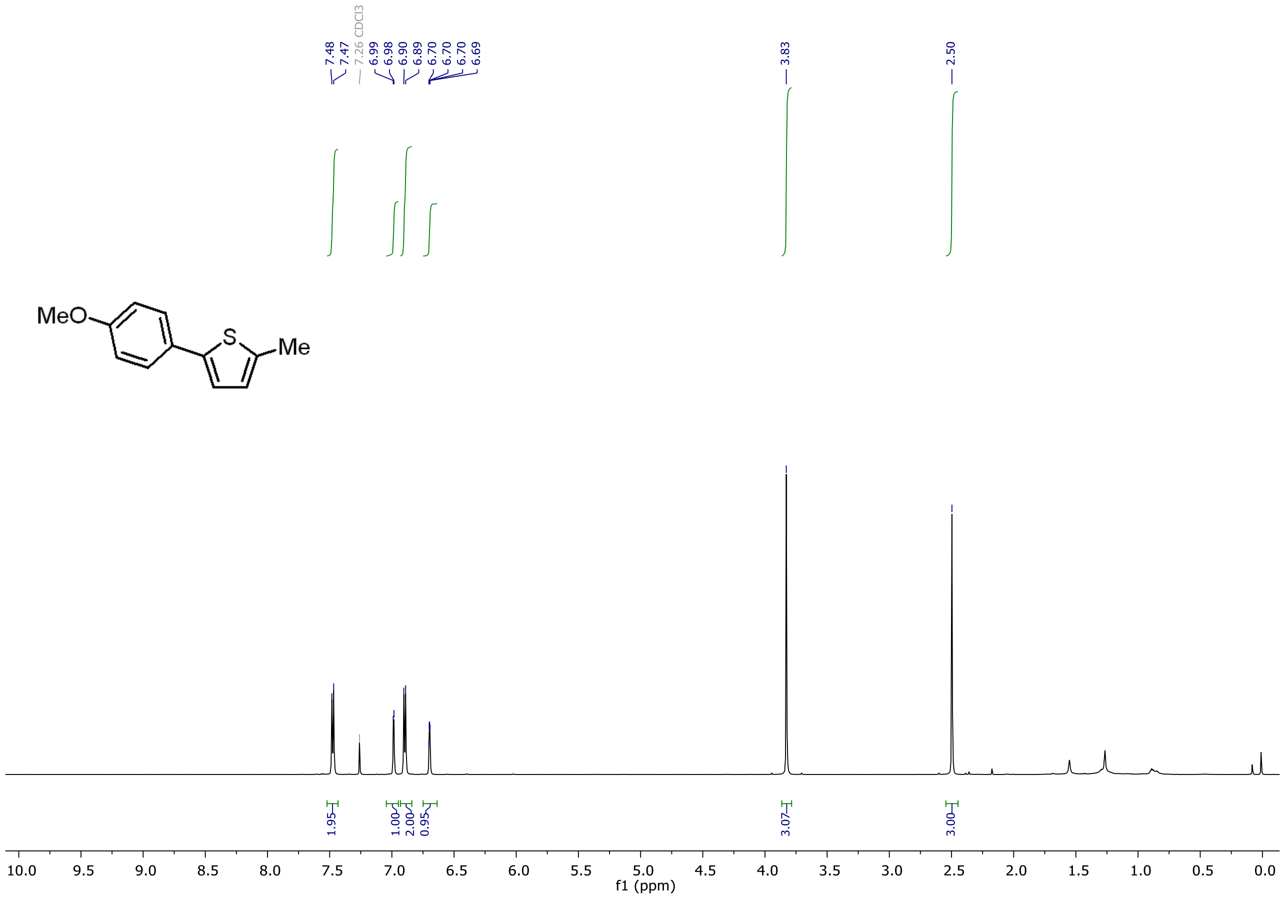

^{1}H NMR (600 MHz, $CDCl_3$) of ***2-(4-methoxyphenyl)-5-methylthiophene (32)***

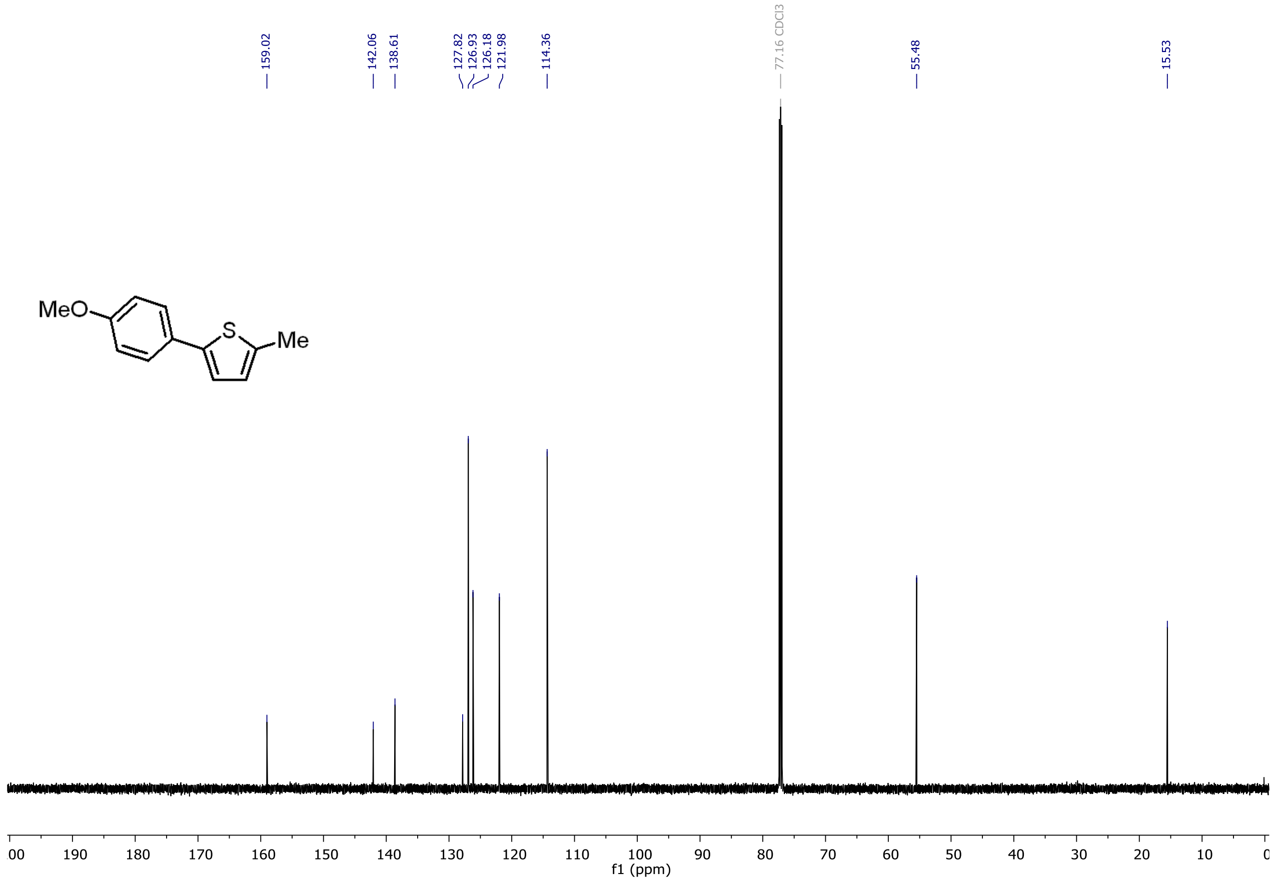

^{13}C NMR (151 MHz, $CDCl_3$) of ***2-(4-methoxyphenyl)-5-methylthiophene (32)***

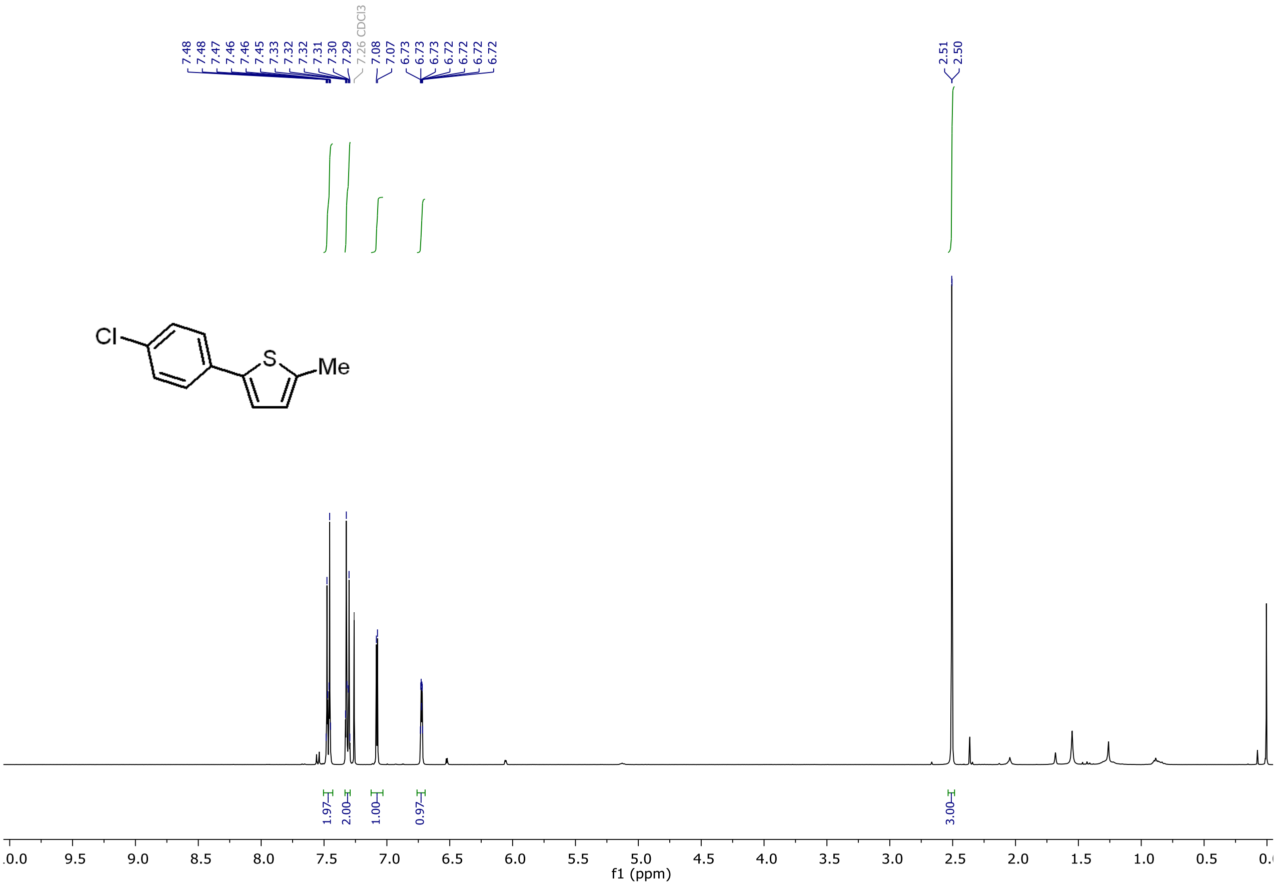

^{1}H NMR (400 MHz, $CDCl_3$) of ***2-(4-chlorophenyl)-5-methylthiophene (33)***

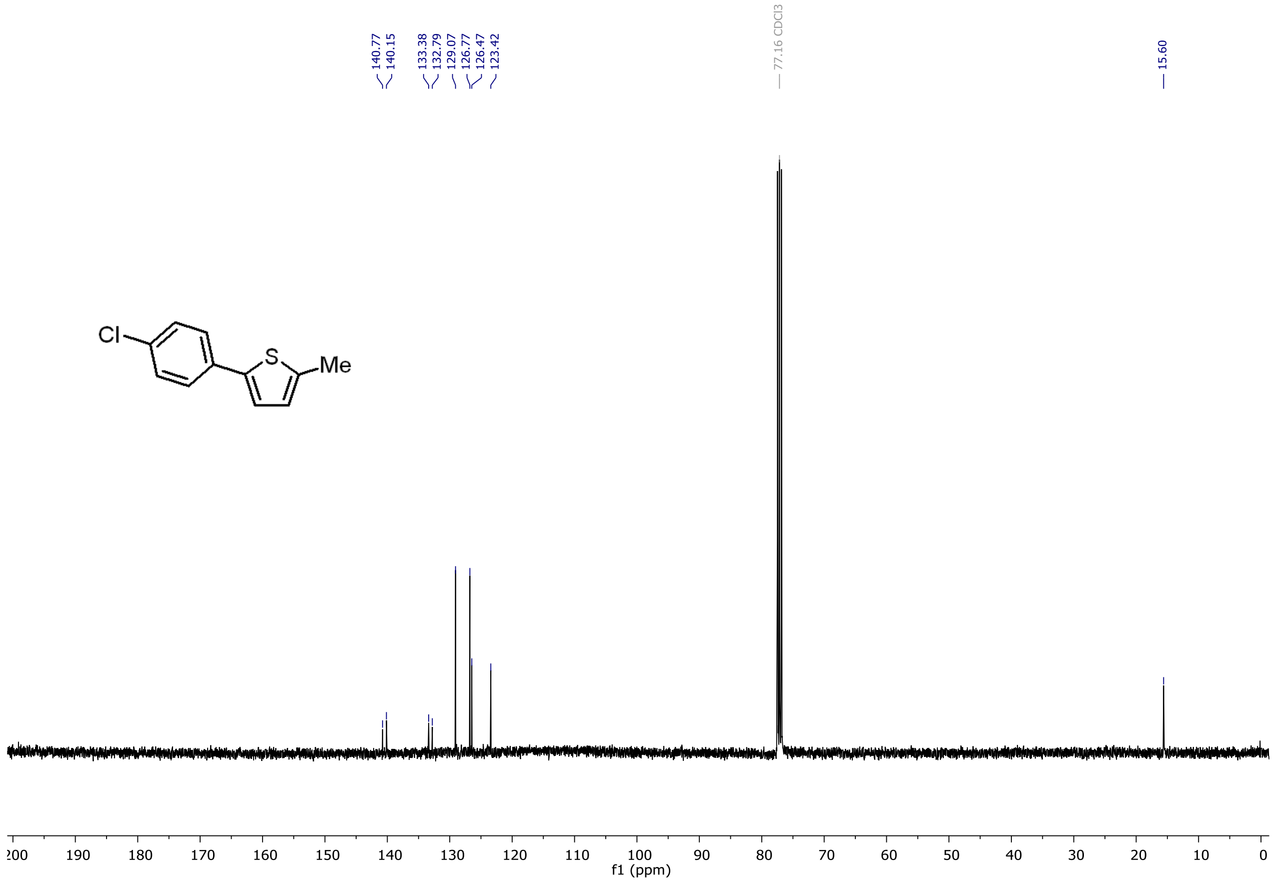

^{13}C NMR (101 MHz, $CDCl_3$) of ***2-(4-chlorophenyl)-5-methylthiophene (33)***

10.4 Cyclopentenones

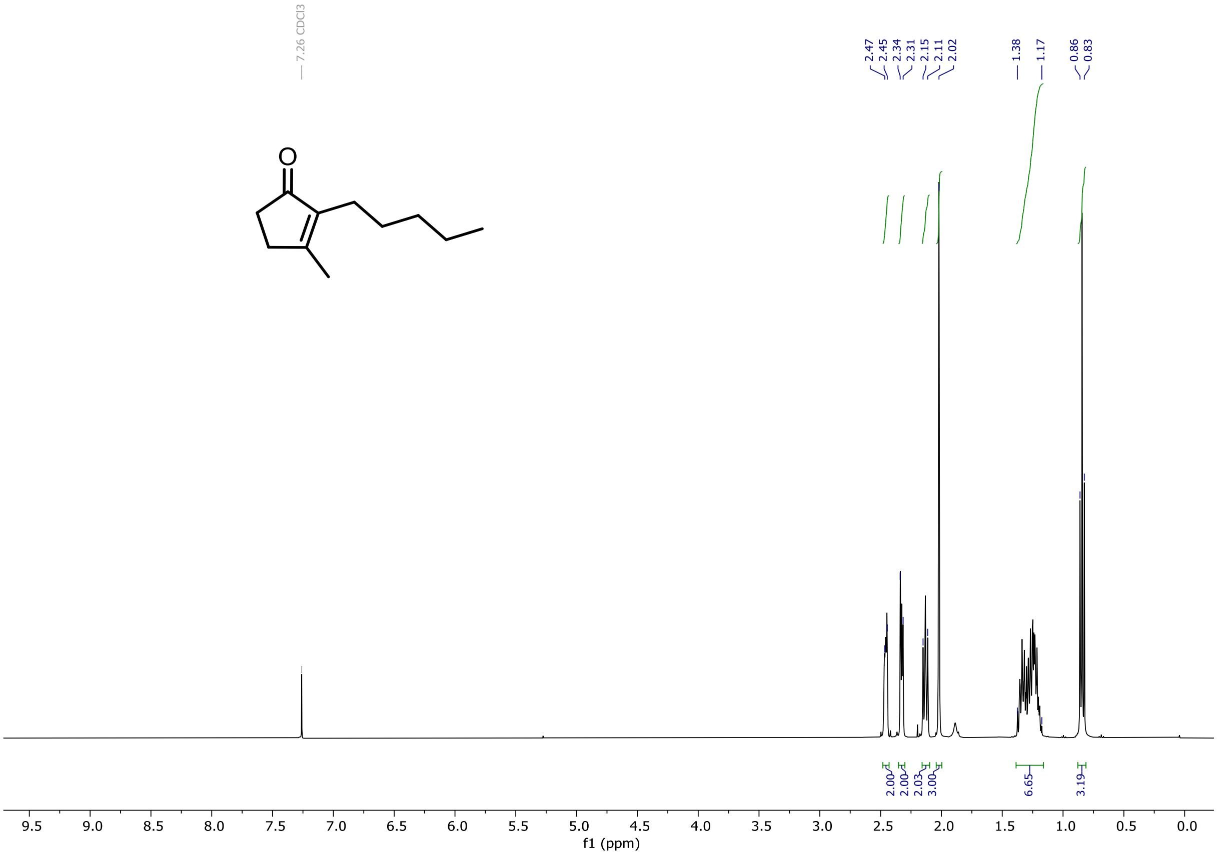

^{1}H NMR (400 MHz, CDCl$_3$) of ***2-hexyl-3-methylcyclopent-2-en-1-one (34)***

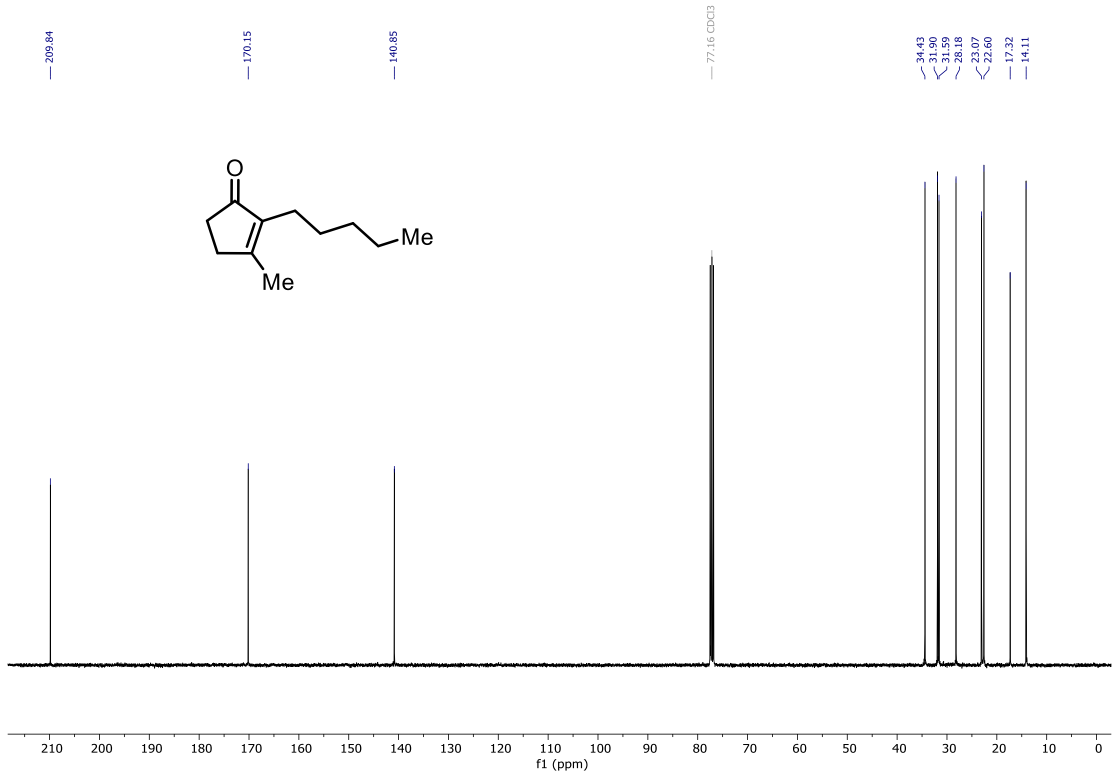

^{13}C NMR (101 MHz, CDCl$_3$) of ***2-hexyl-3-methylcyclopent-2-en-1-one (34)***

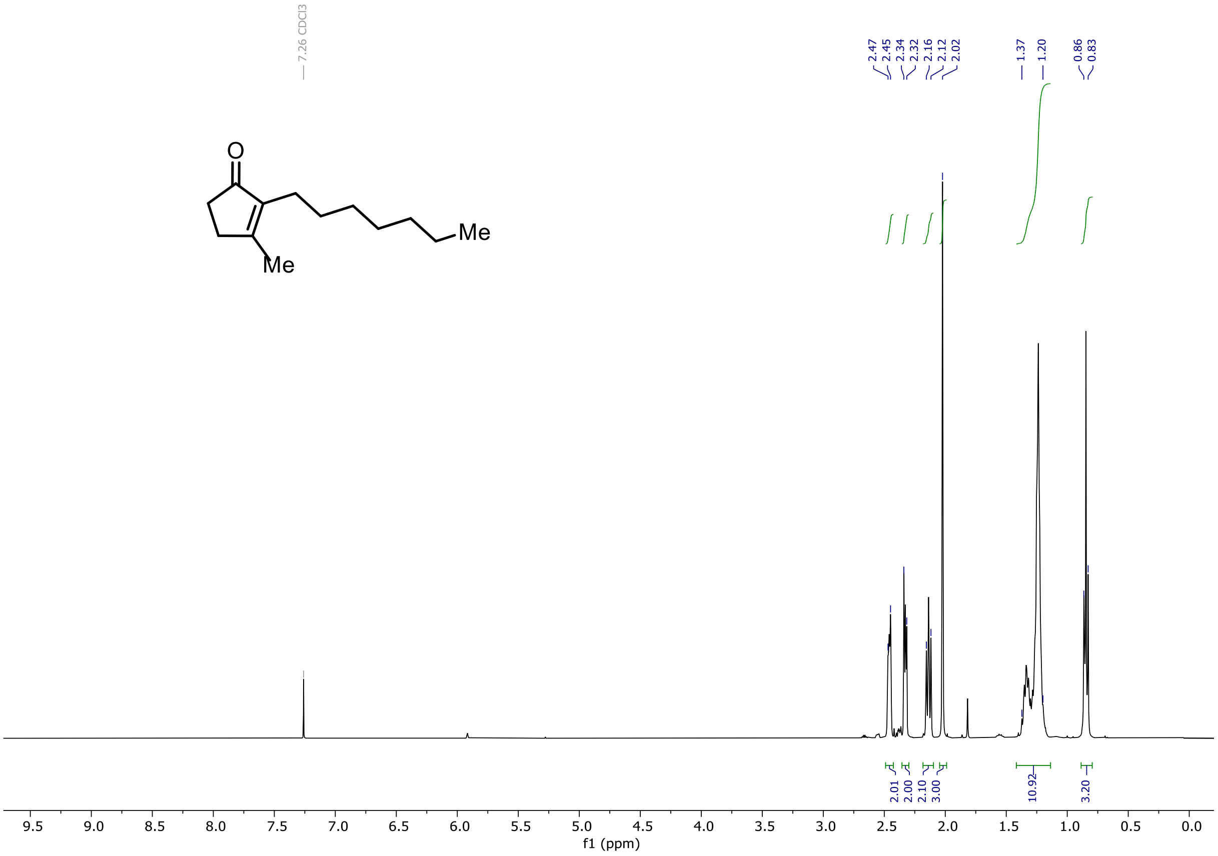

^{1}H NMR (400 MHz, CDCl$_3$) of ***3-methyl-2-octylcyclopent-2-en-1-one (35)***

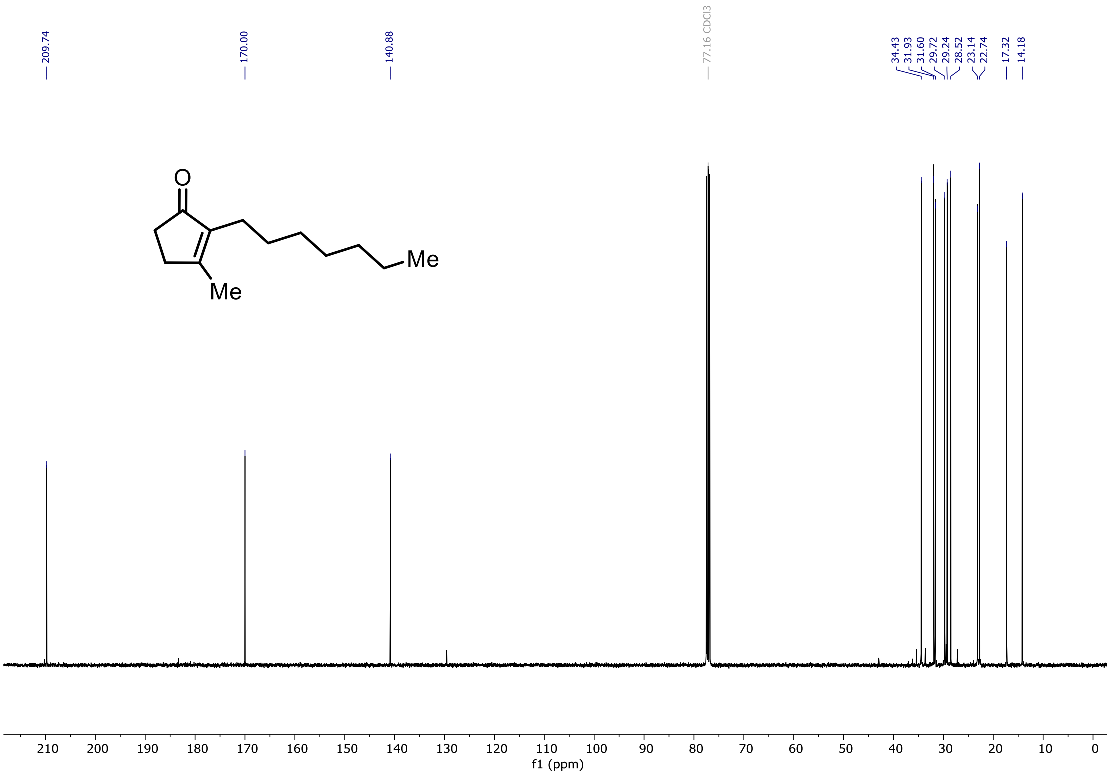

^{13}C NMR (101 MHz, CDCl$_3$) of ***3-methyl-2-octylcyclopent-2-en-1-one (35)***

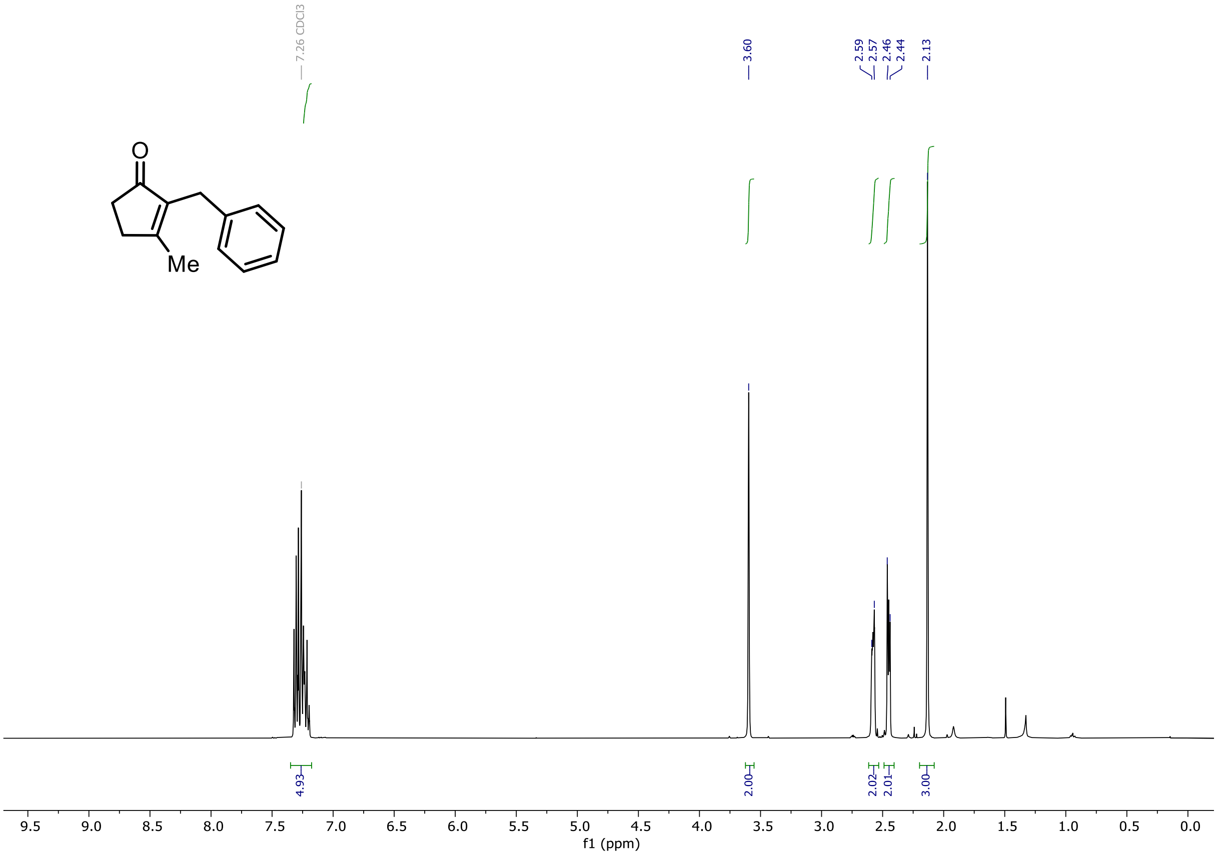

^{1}H NMR (400 MHz, $CDCl_3$) of ***2-benzyl-3-methylcyclopent-2-en-1-one (36)***

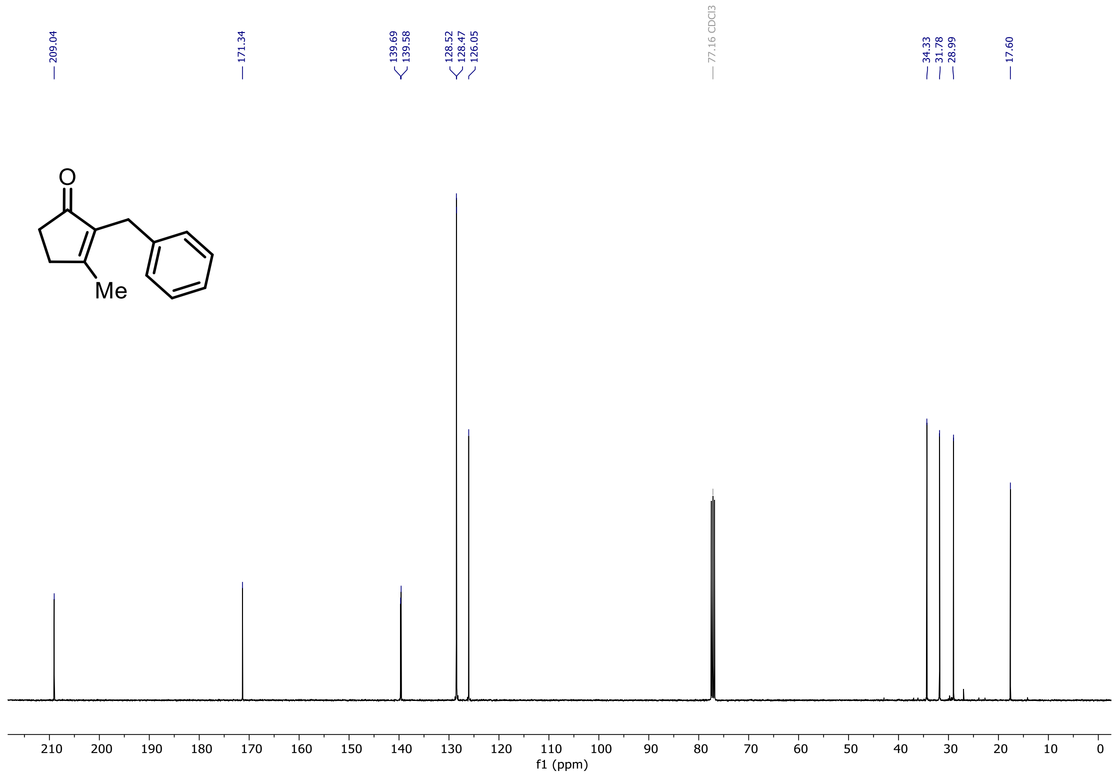

^{13}C NMR (101 MHz, $CDCl_3$) of ***2-benzyl-3-methylcyclopent-2-en-1-one (36)***

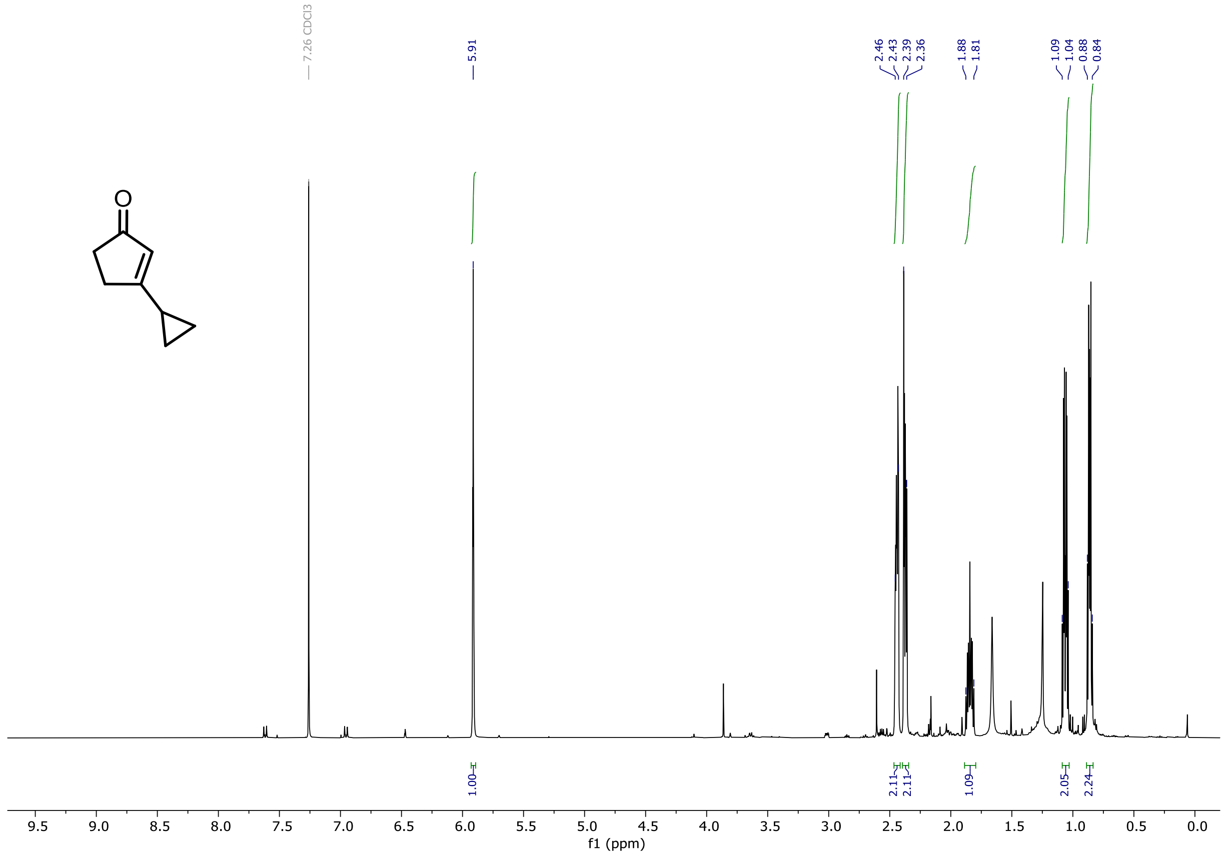

^{1}H NMR (400 MHz, $CDCl_3$) of ***3-cyclopropylcyclopent-2-en-1-one (37)***

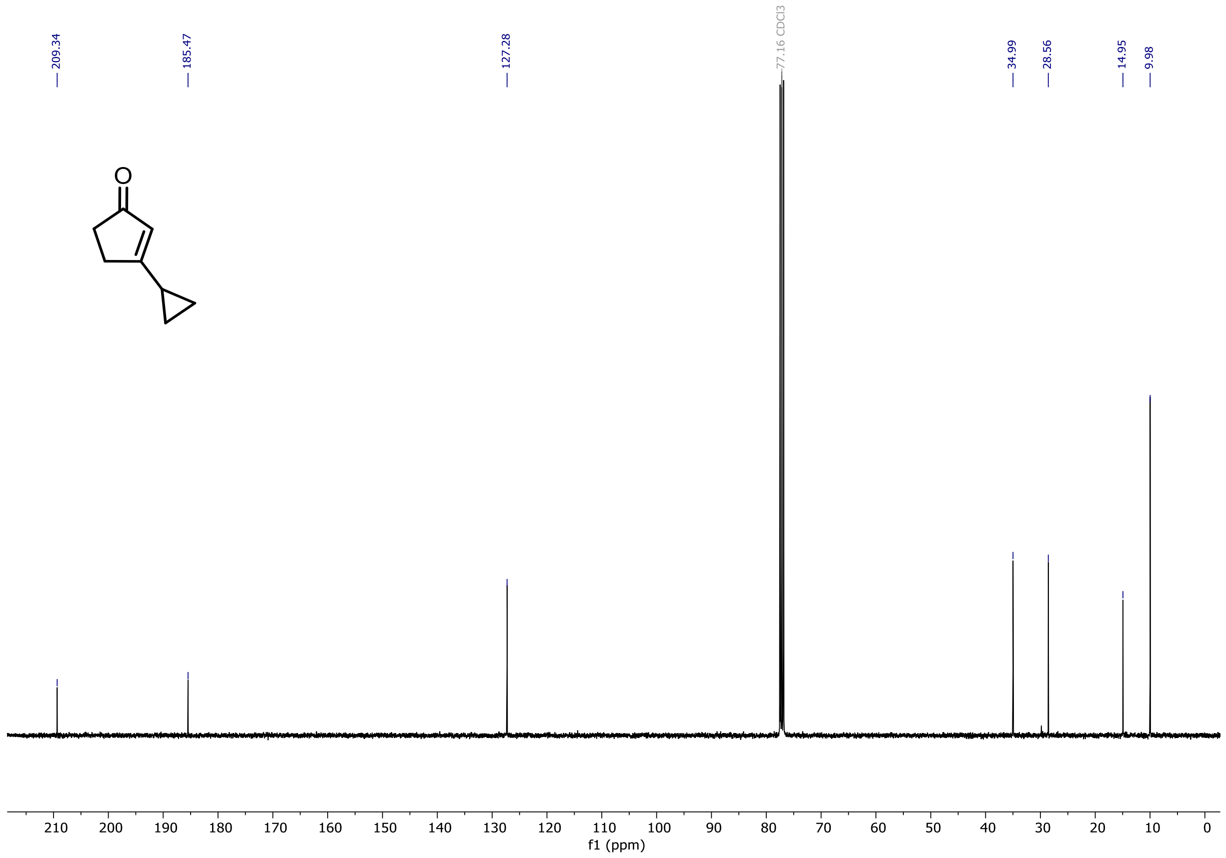

^{13}C NMR (101 MHz, $CDCl_3$) of ***3-cyclopropylcyclopent-2-en-1-one (37)***

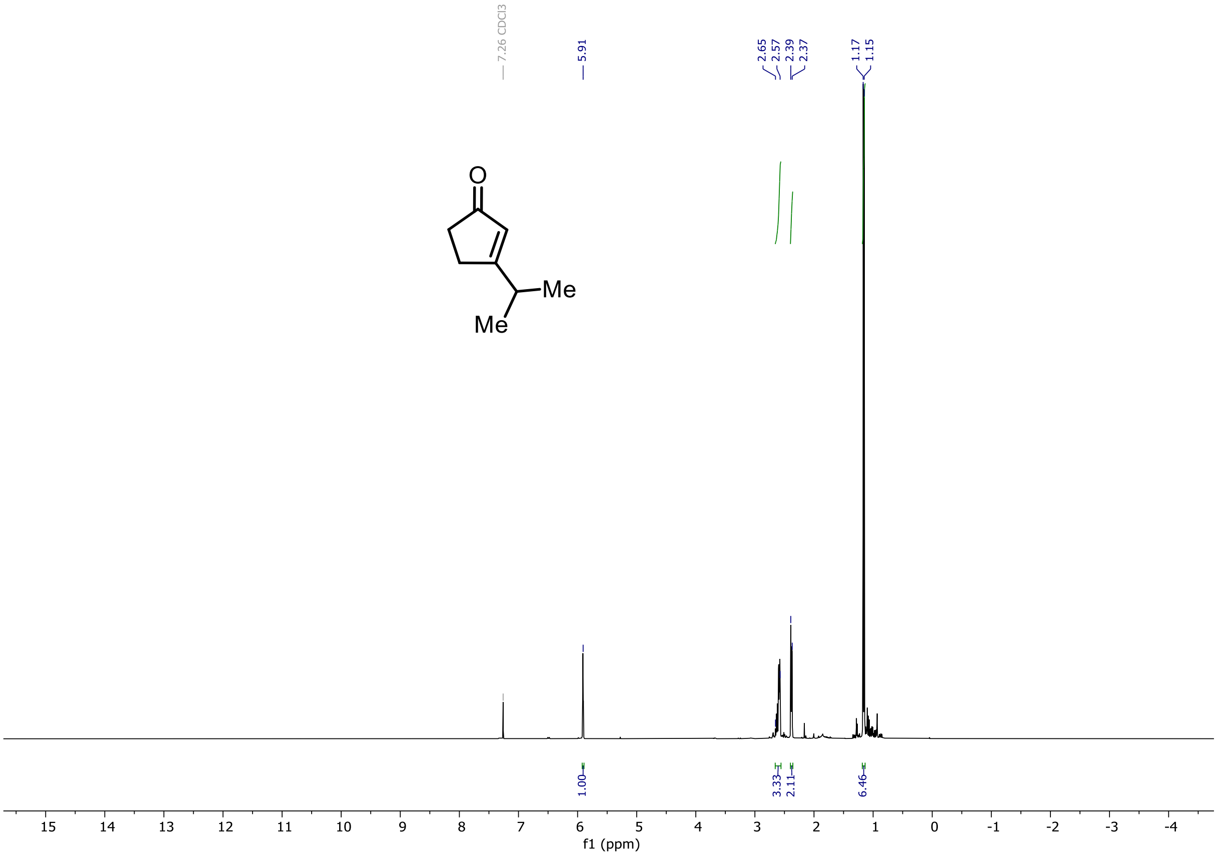

^{1}H NMR (400 MHz, $CDCl_3$) of ***3-isopropylcyclopent-2-en-1-one (38)***

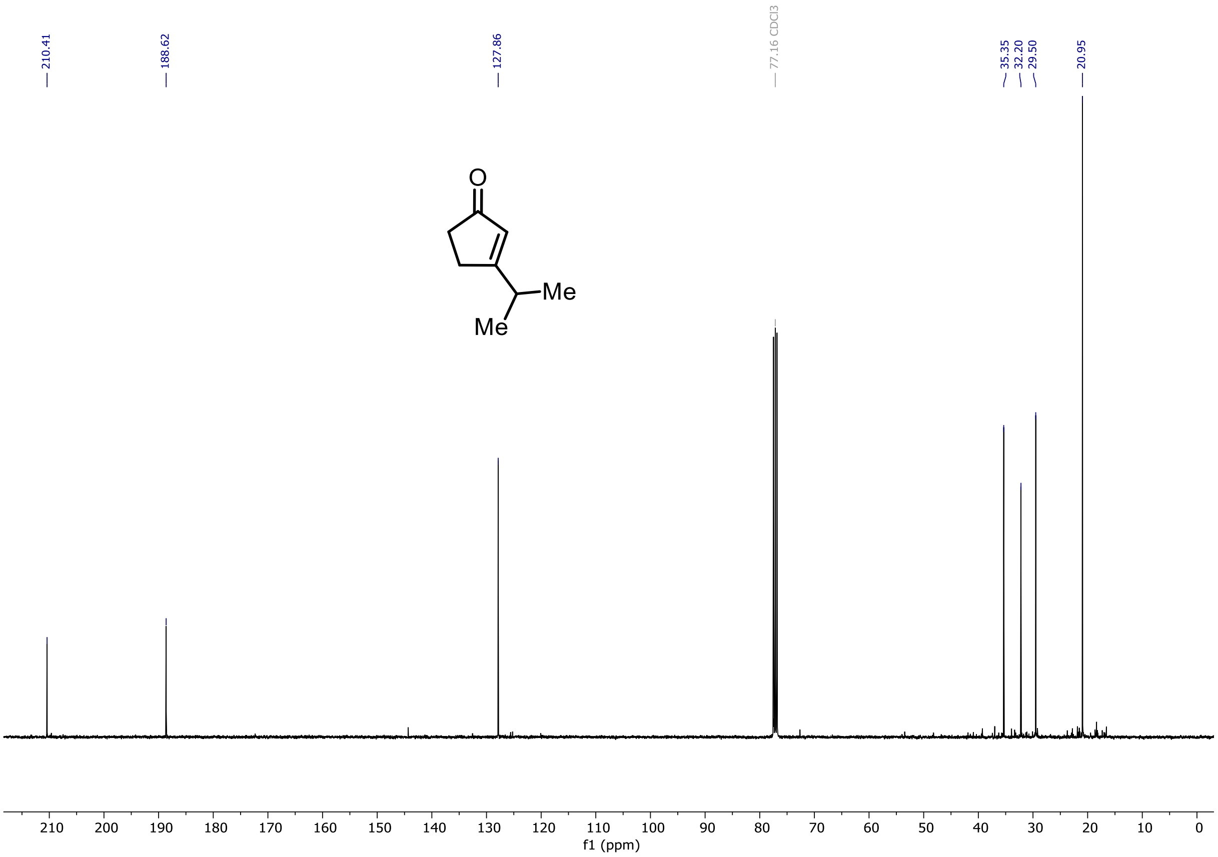

^{13}C NMR (101 MHz, $CDCl_3$) of ***3-isopropylcyclopent-2-en-1-one (38)***

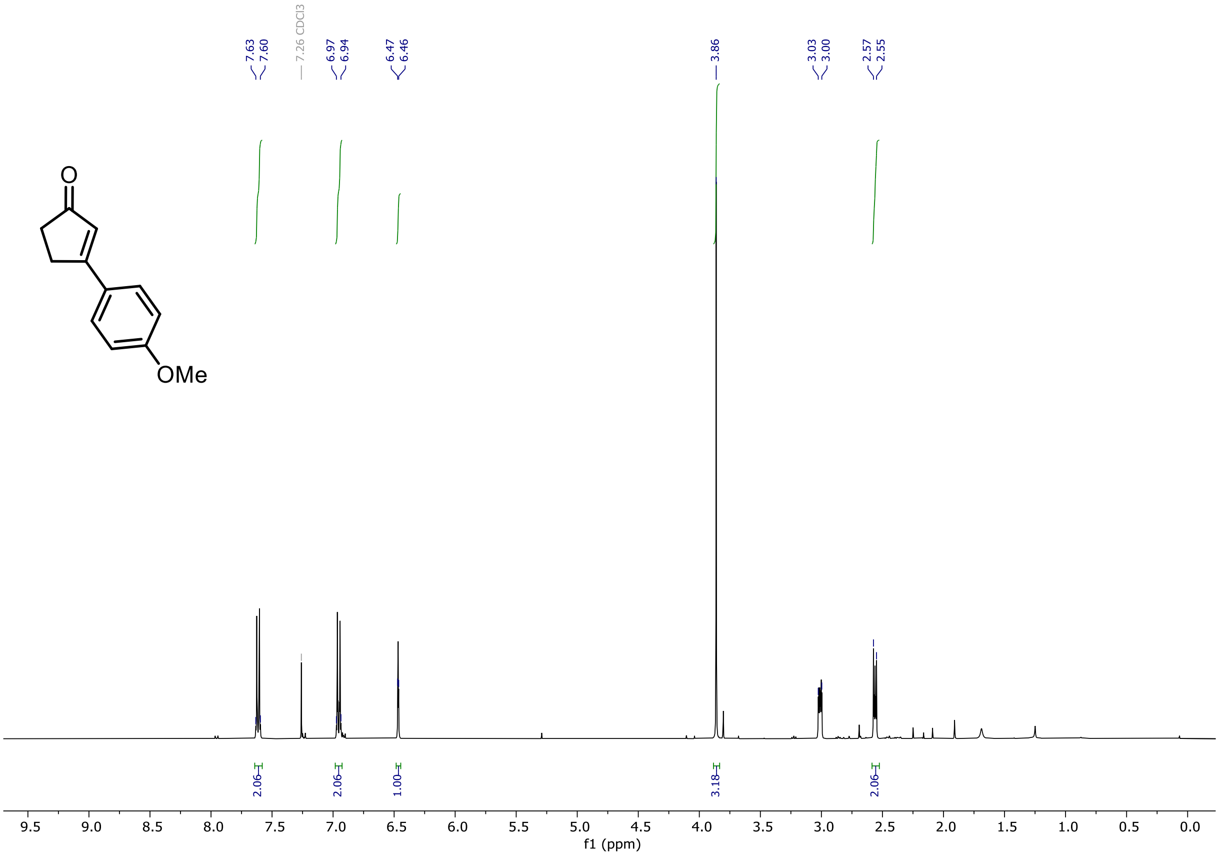

^{1}H NMR (400 MHz, $CDCl_3$) of ***3-(4-methoxyphenyl)cyclopent-2-en-1-one (39)***

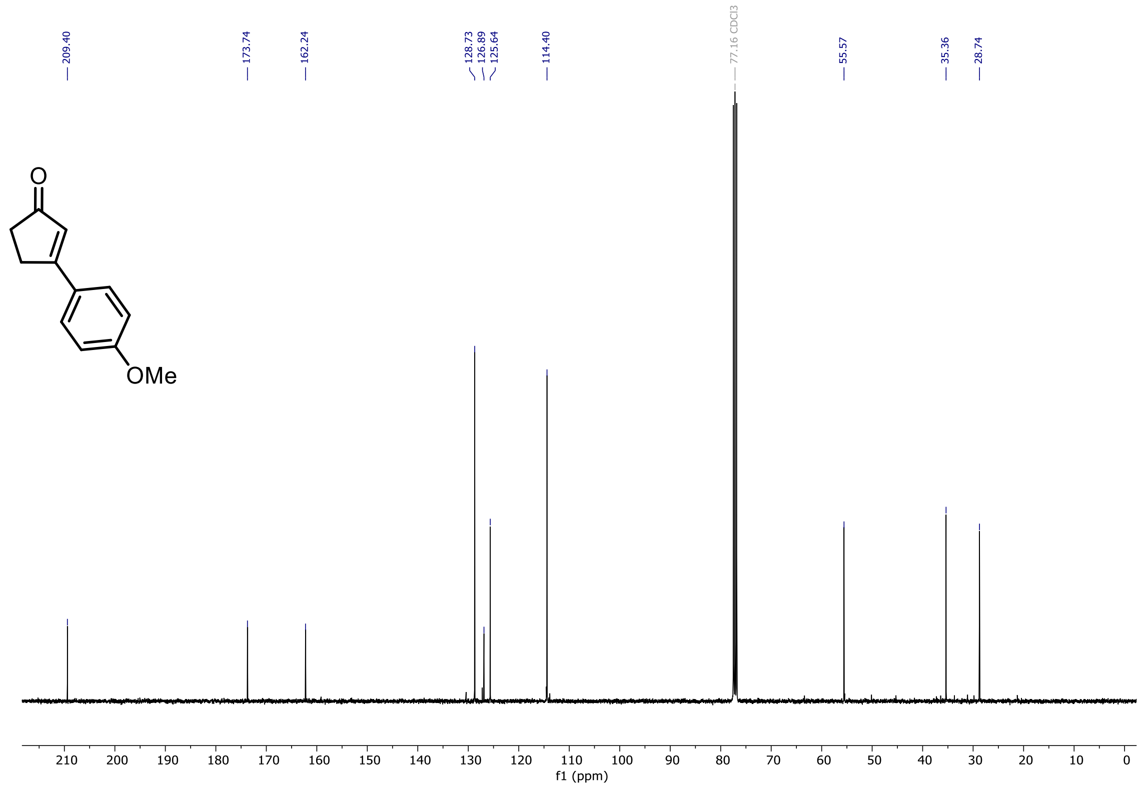

^{13}C NMR (101 MHz, $CDCl_3$) of ***3-(4-methoxyphenyl)cyclopent-2-en-1-one (39)***

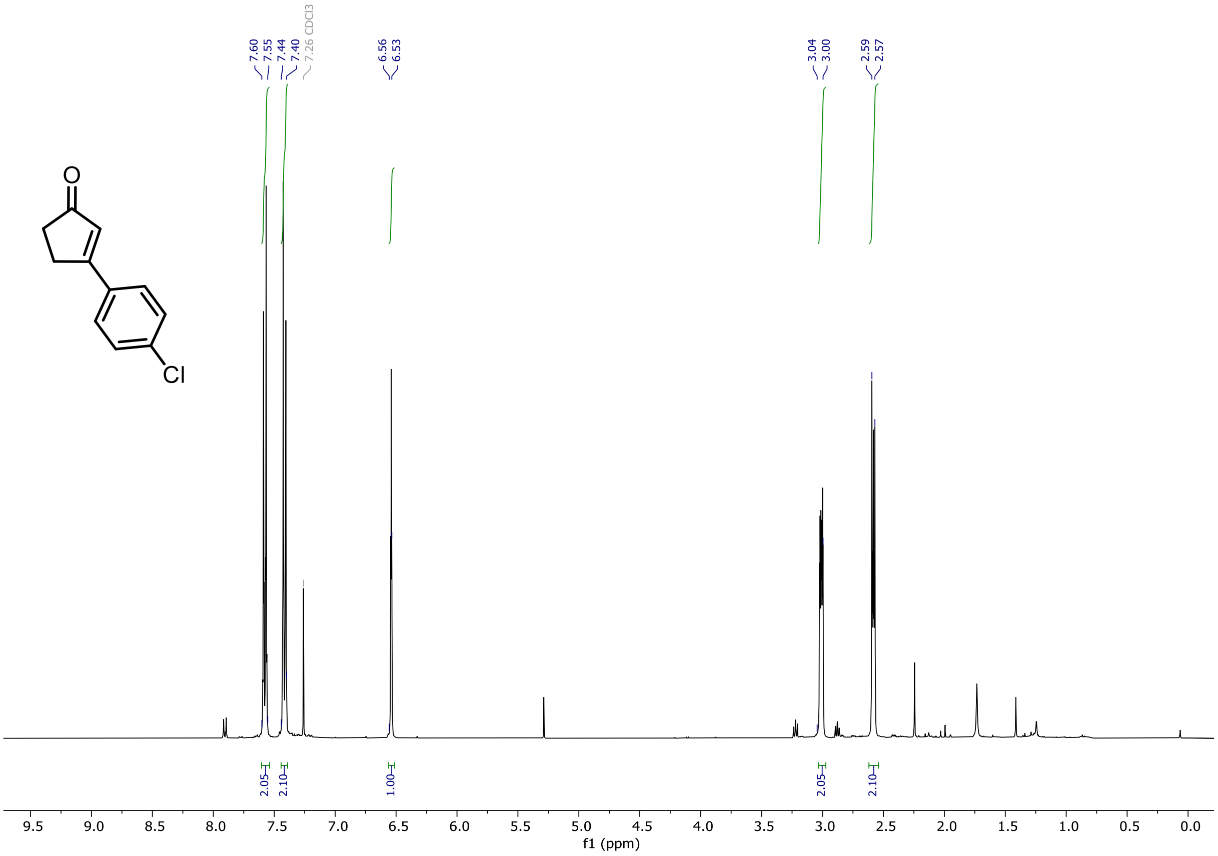

^{1}H NMR (400 MHz, $CDCl_3$) of ***3-(4-chlorophenyl)cyclopent-2-en-1-one (40)***

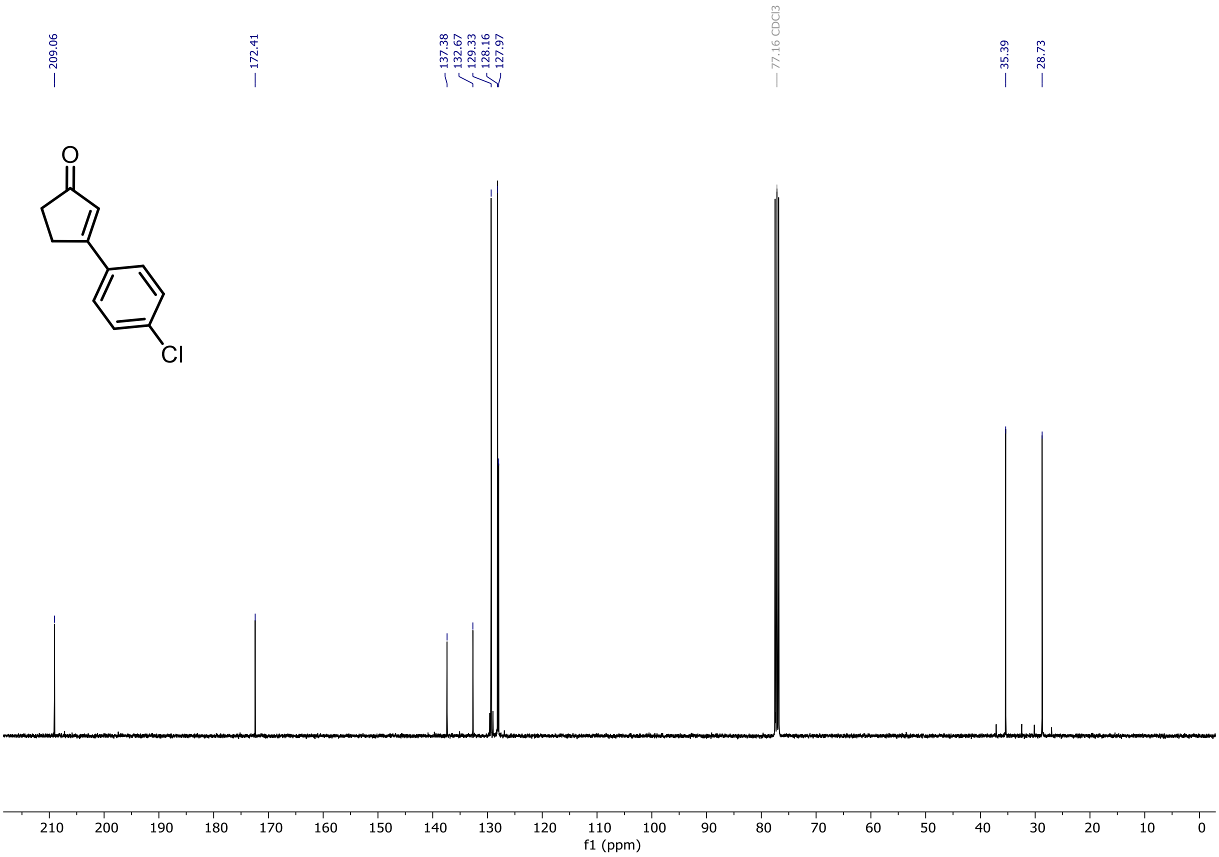

^{13}C NMR (101 MHz, $CDCl_3$) of ***3-(4-chlorophenyl)cyclopent-2-en-1-one (40)***

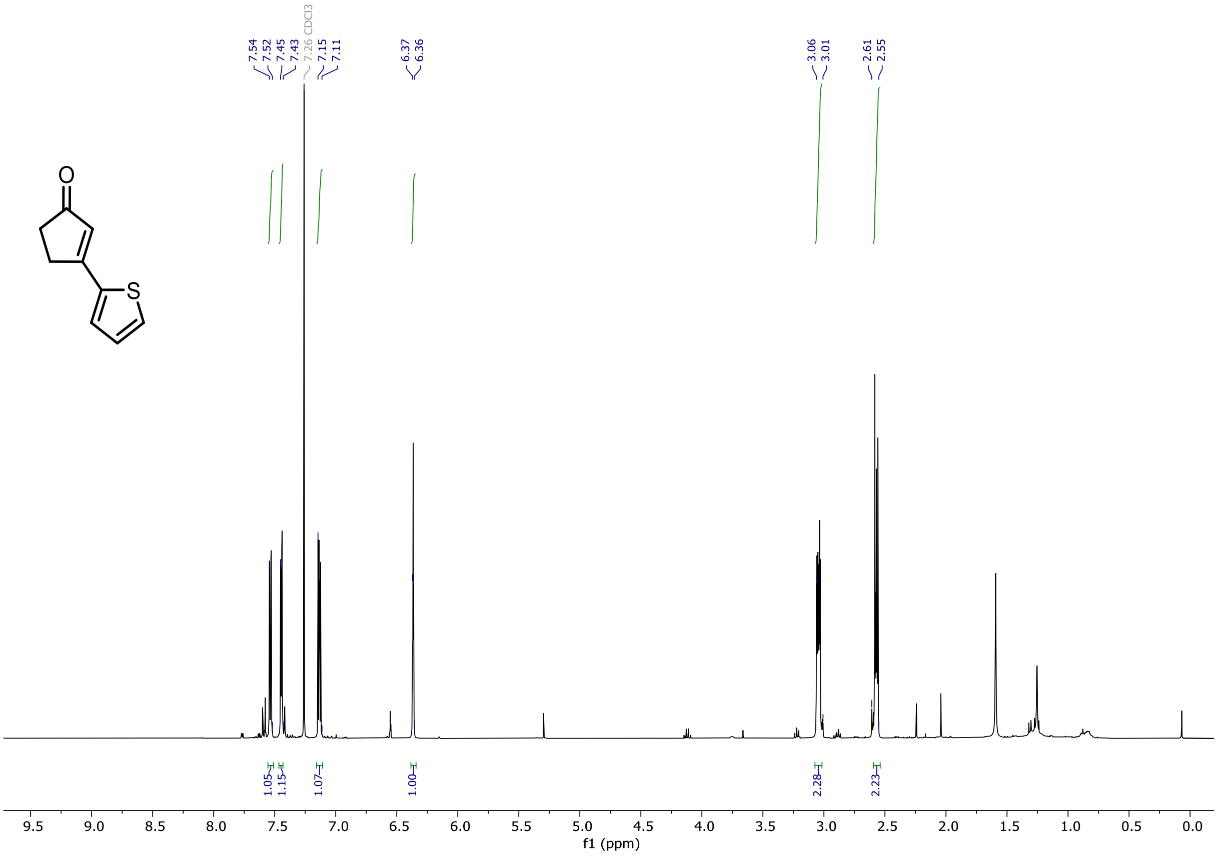

^{1}**H NMR (400 MHz, CDCl$_3$)** of ***3-(thiophen-2-yl)cyclopent-2-en-1-one (41)***

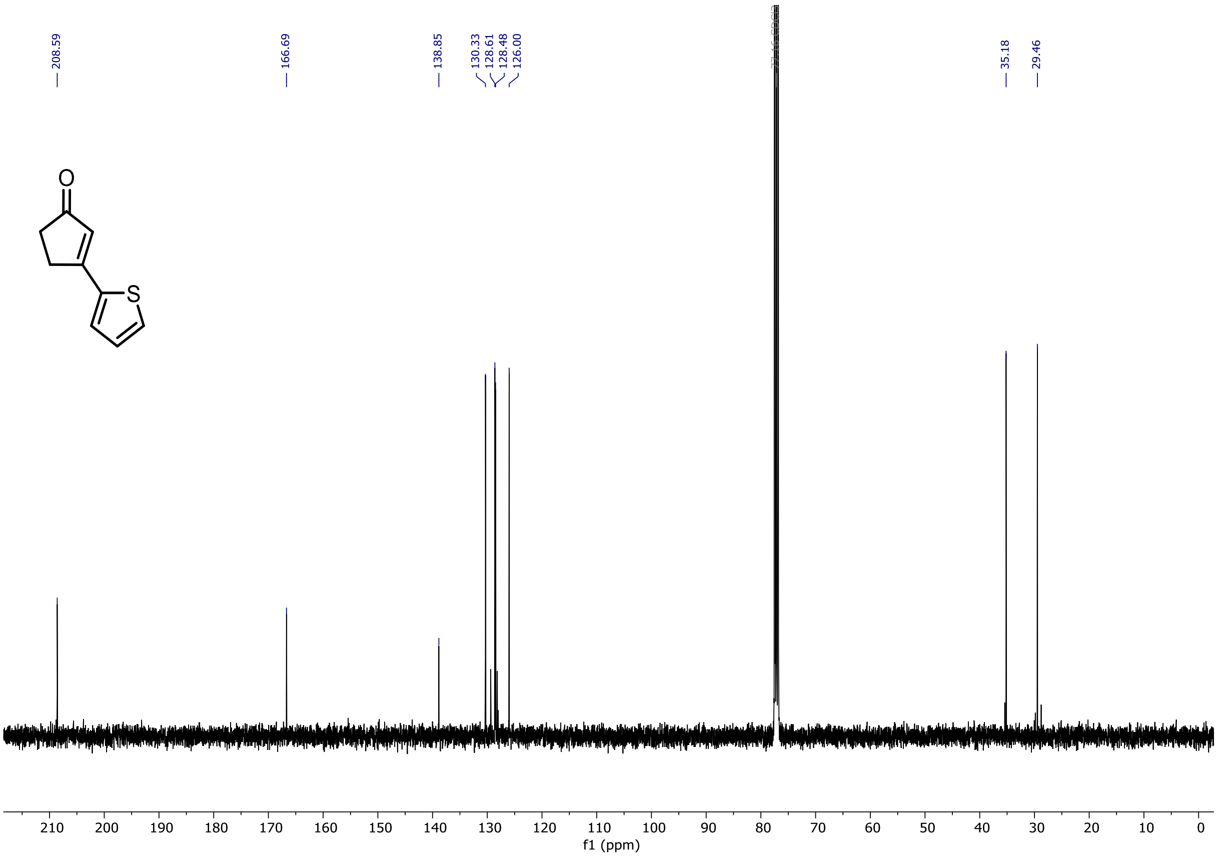

^{13}**C NMR (101 MHz, CDCl$_3$)** of ***3-(thiophen-2-yl)cyclopent-2-en-1-one (41)***

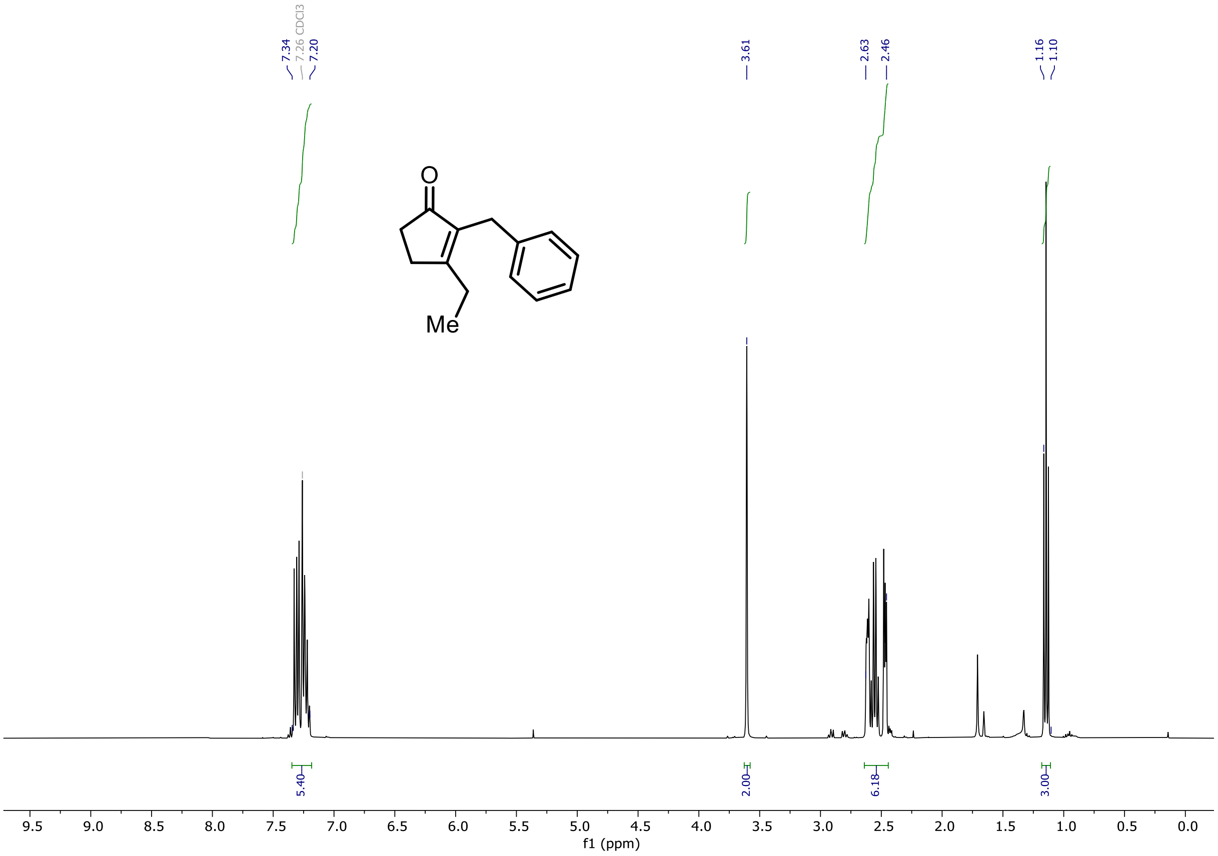

^{1}H NMR (400 MHz, $CDCl_3$) of ***2-benzyl-3-ethylcyclopent-2-en-1-one*** **(*42*)**

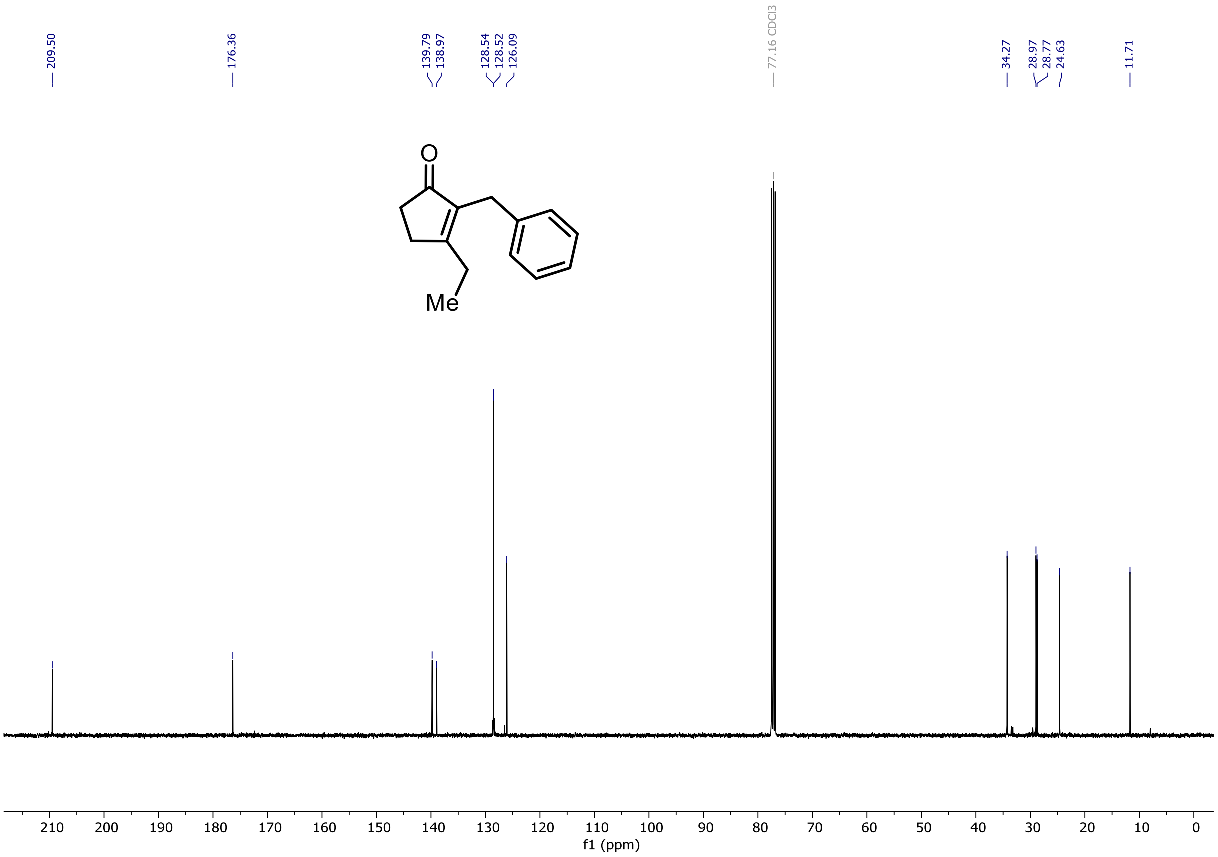

^{13}C NMR (101 MHz, $CDCl_3$) of ***2-benzyl-3-ethylcyclopent-2-en-1-one (42)***

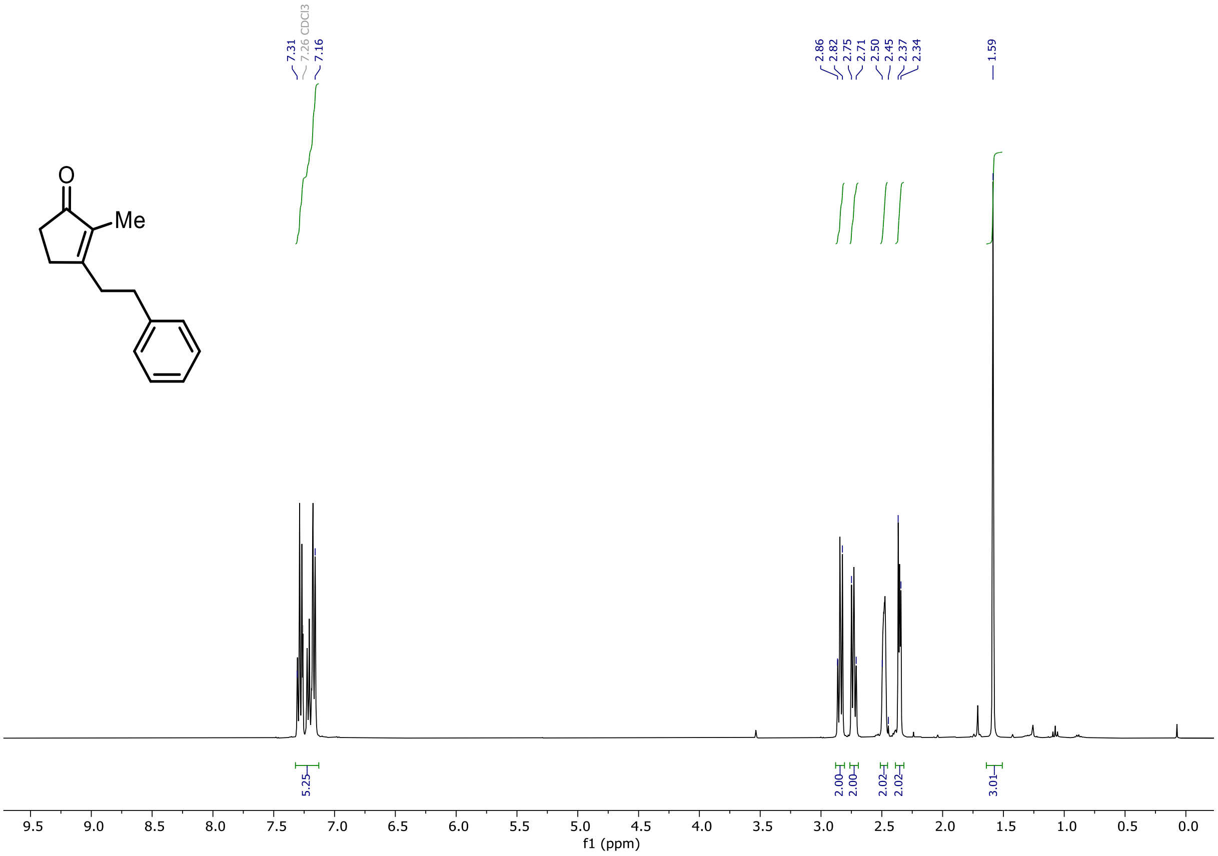

^{1}H NMR (400 MHz, $CDCl_3$) of ***2-methyl-3-phenethylcyclopent-2-en-1-one (42')***

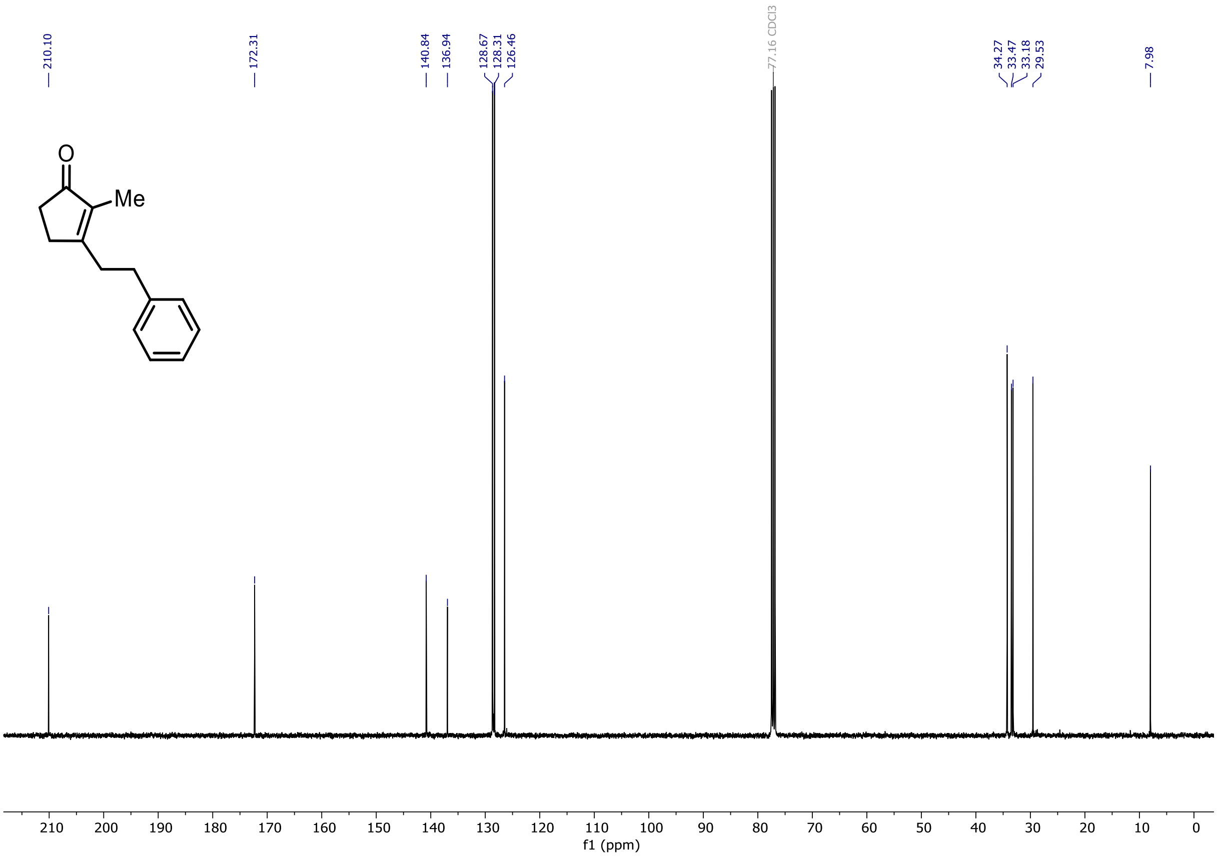

^{13}C NMR (101 MHz, $CDCl_3$) of ***2-methyl-3-phenethylcyclopent-2-en-1-one (42')***

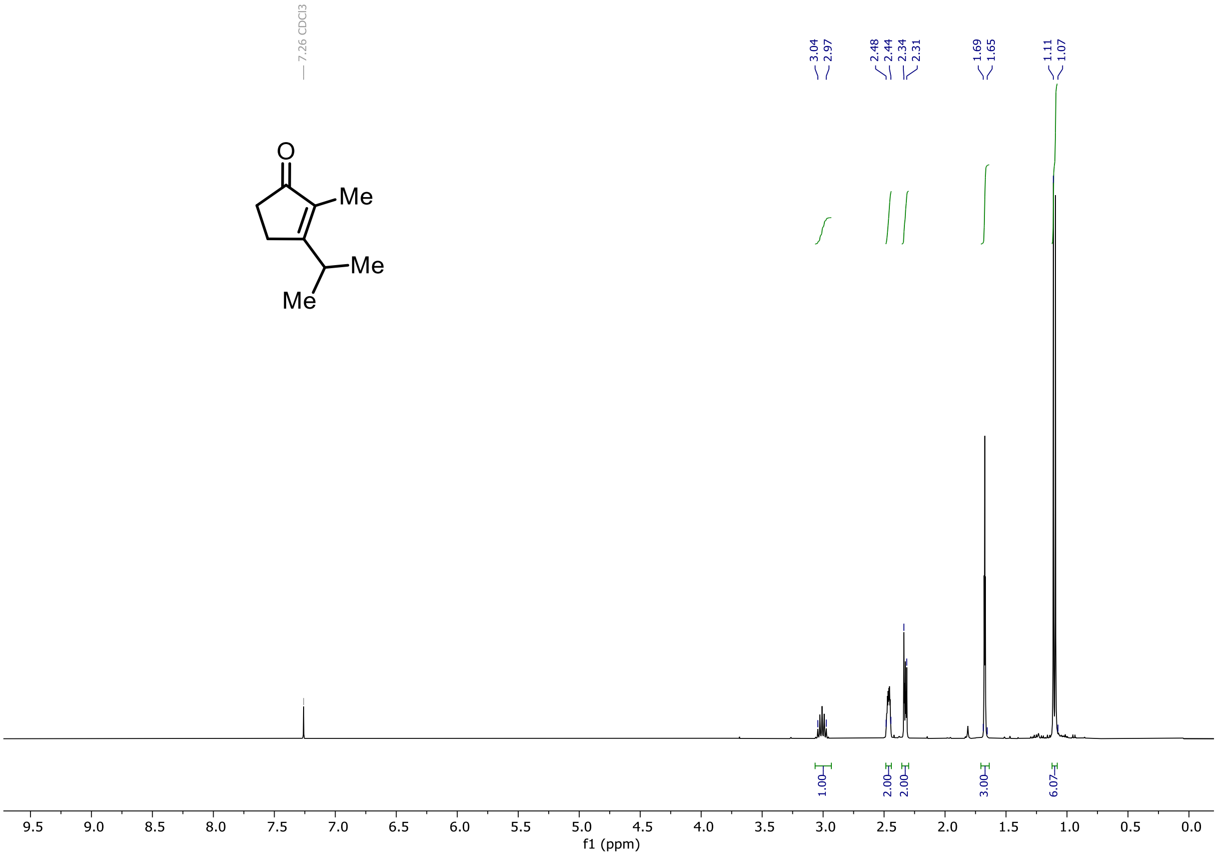

^{1}H NMR (400 MHz, $CDCl_3$) of ***3-isopropyl-2-methylcyclopent-2-en-1-one (43)***

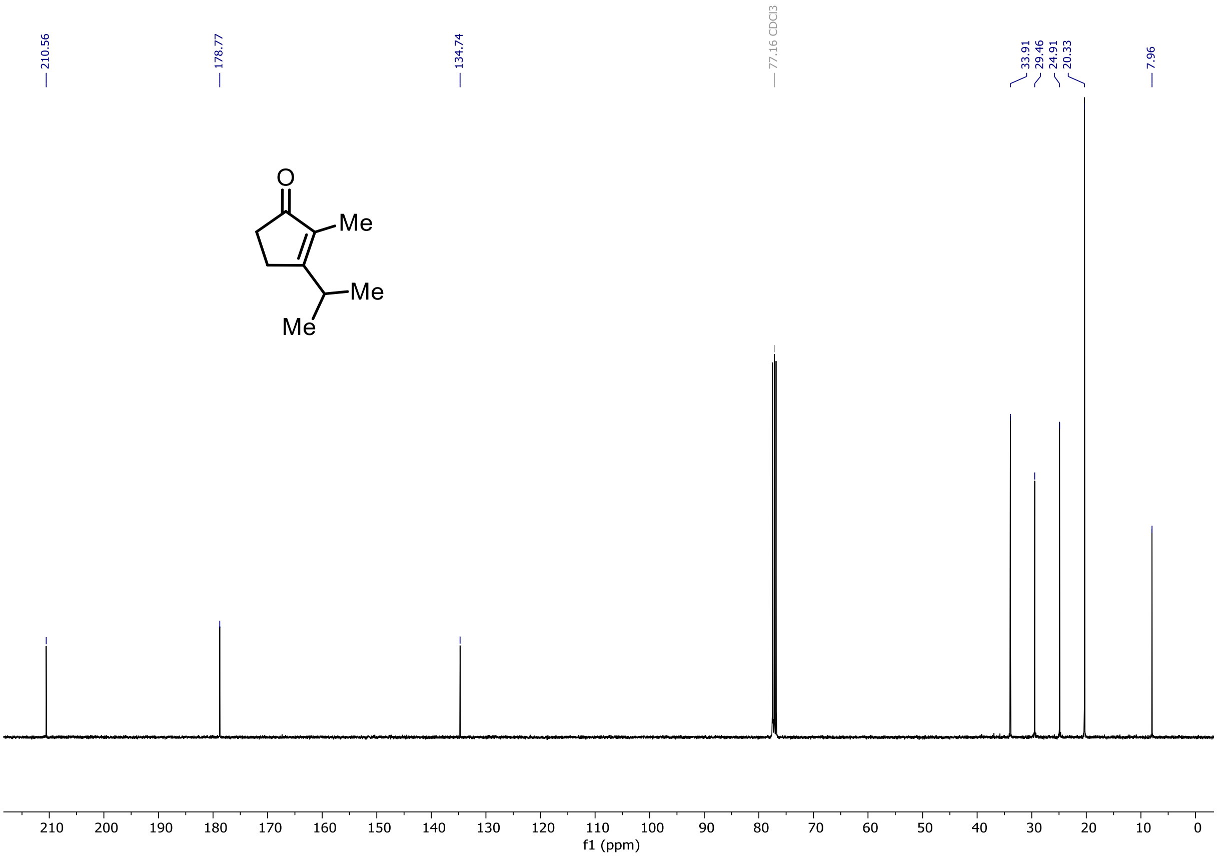

^{13}C NMR (101 MHz, $CDCl_3$) of ***3-isopropyl-2-methylcyclopent-2-en-1-one (43)***

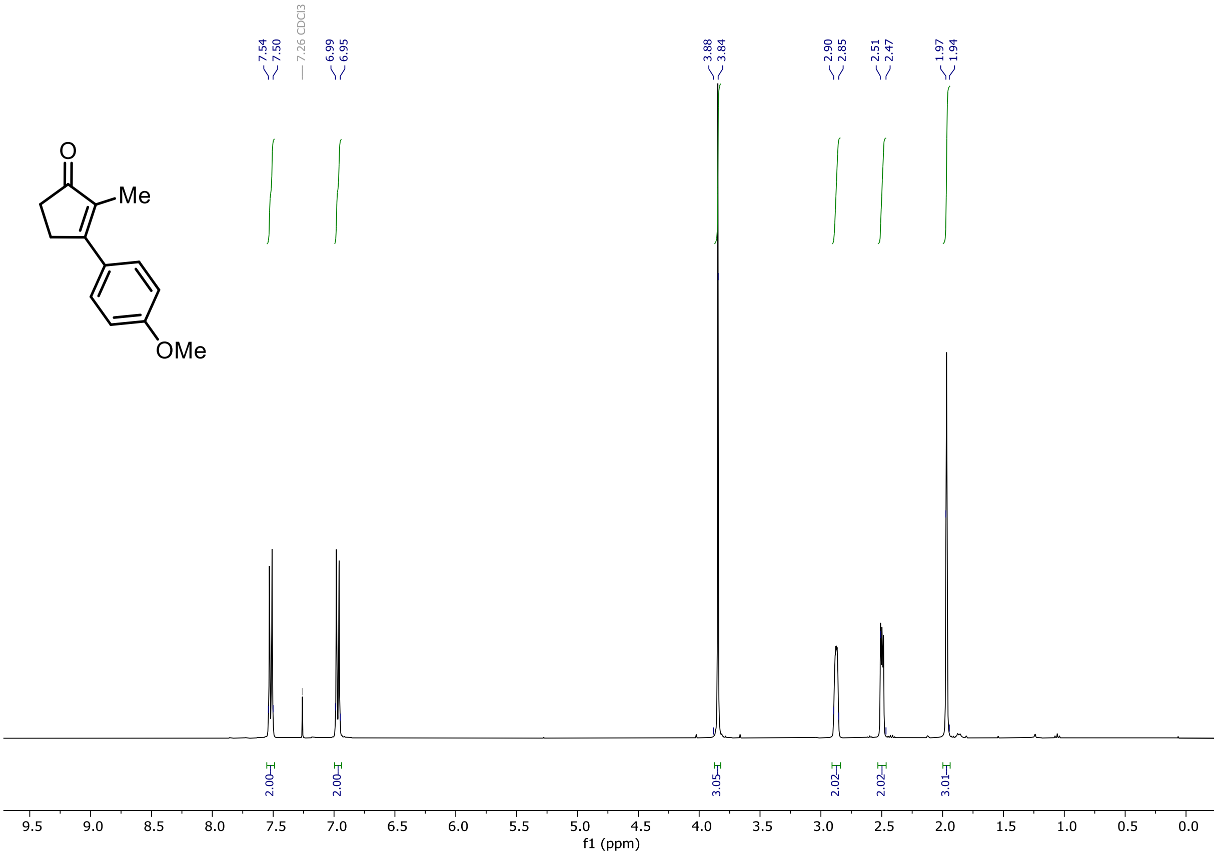

^{1}H NMR (400 MHz, $CDCl_3$) of ***3-(4-methoxyphenyl)-2-methylcyclopent-2-en-1-one (44)***

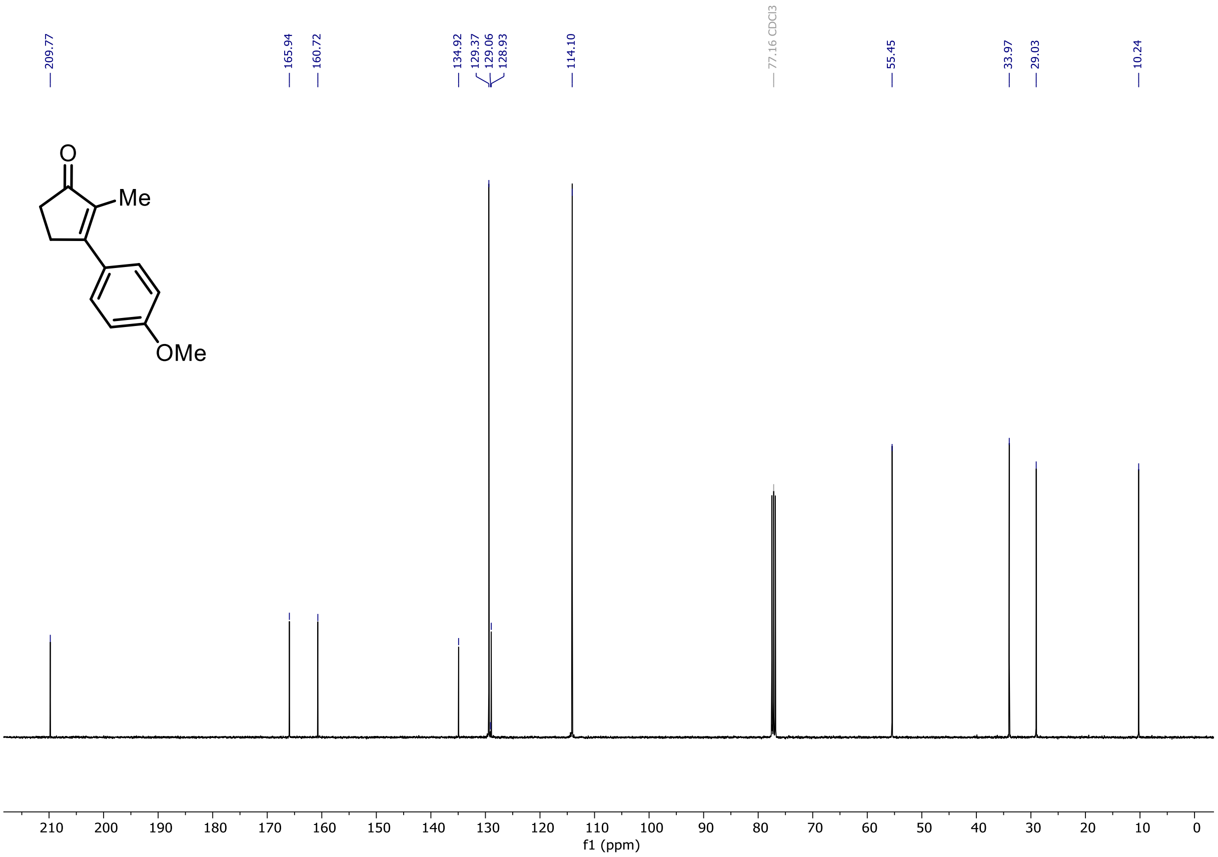

^{13}C NMR (101 MHz, $CDCl_3$) of ***3-(4-methoxyphenyl)-2-methylcyclopent-2-en-1-one (44)***

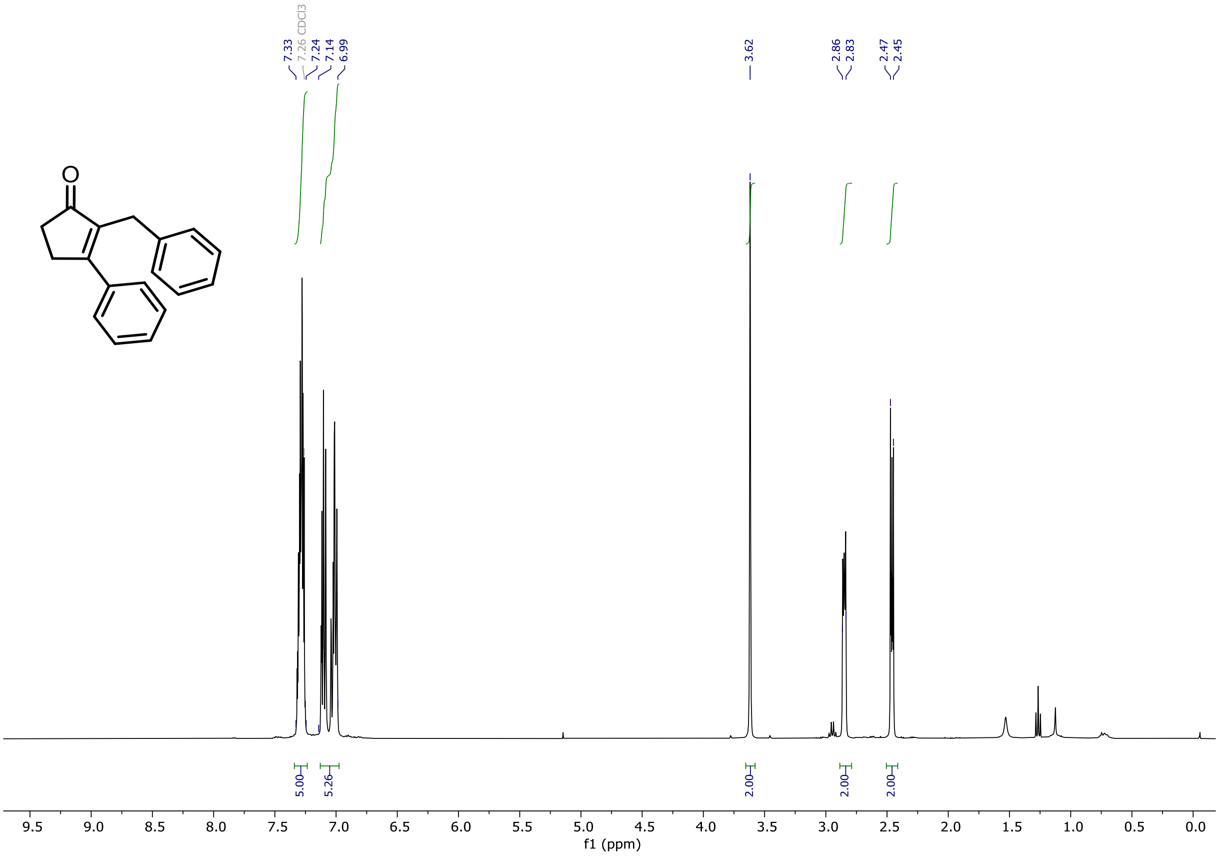

^{1}H NMR (400 MHz, $CDCl_3$) of *2-benzyl-3-phenylcyclopent-2-en-1-one (45)*

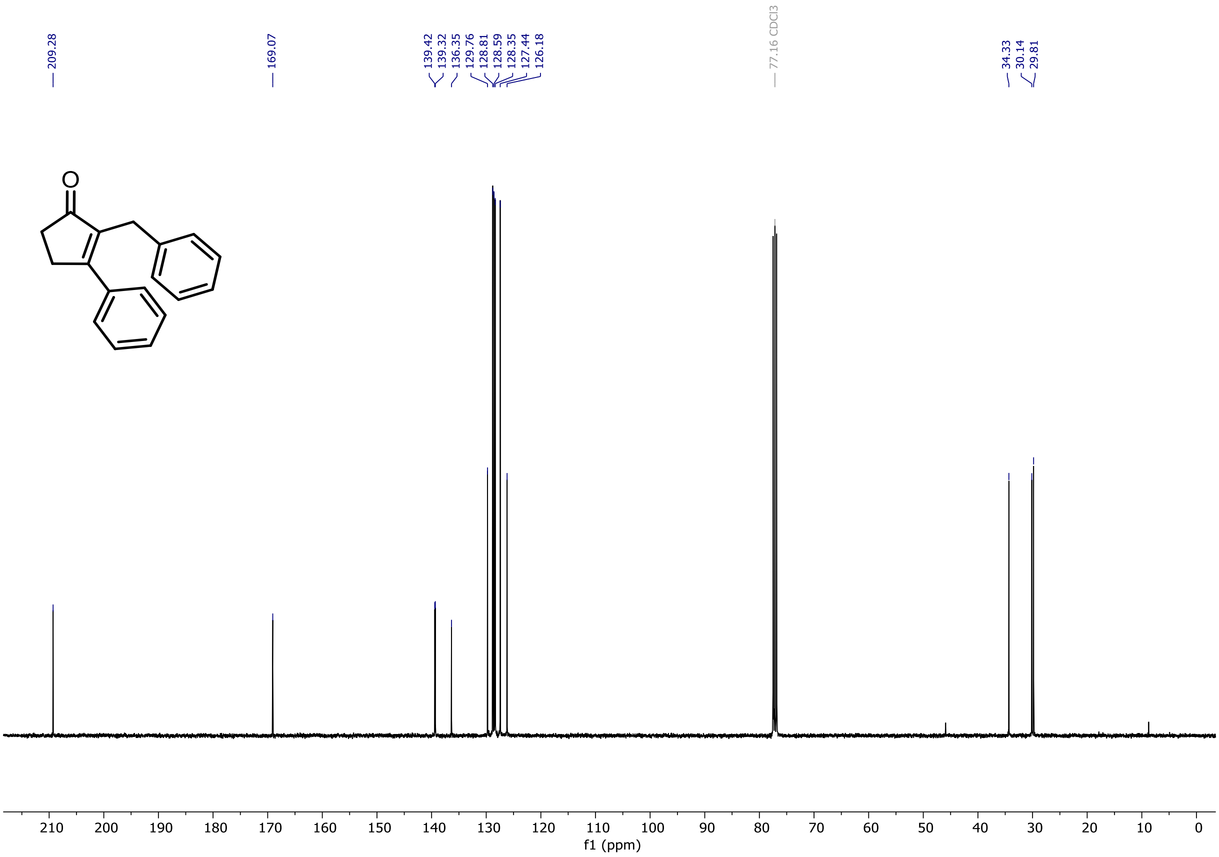

^{13}C NMR (101 MHz, $CDCl_3$) of *2-benzyl-3-phenylcyclopent-2-en-1-one (45)*

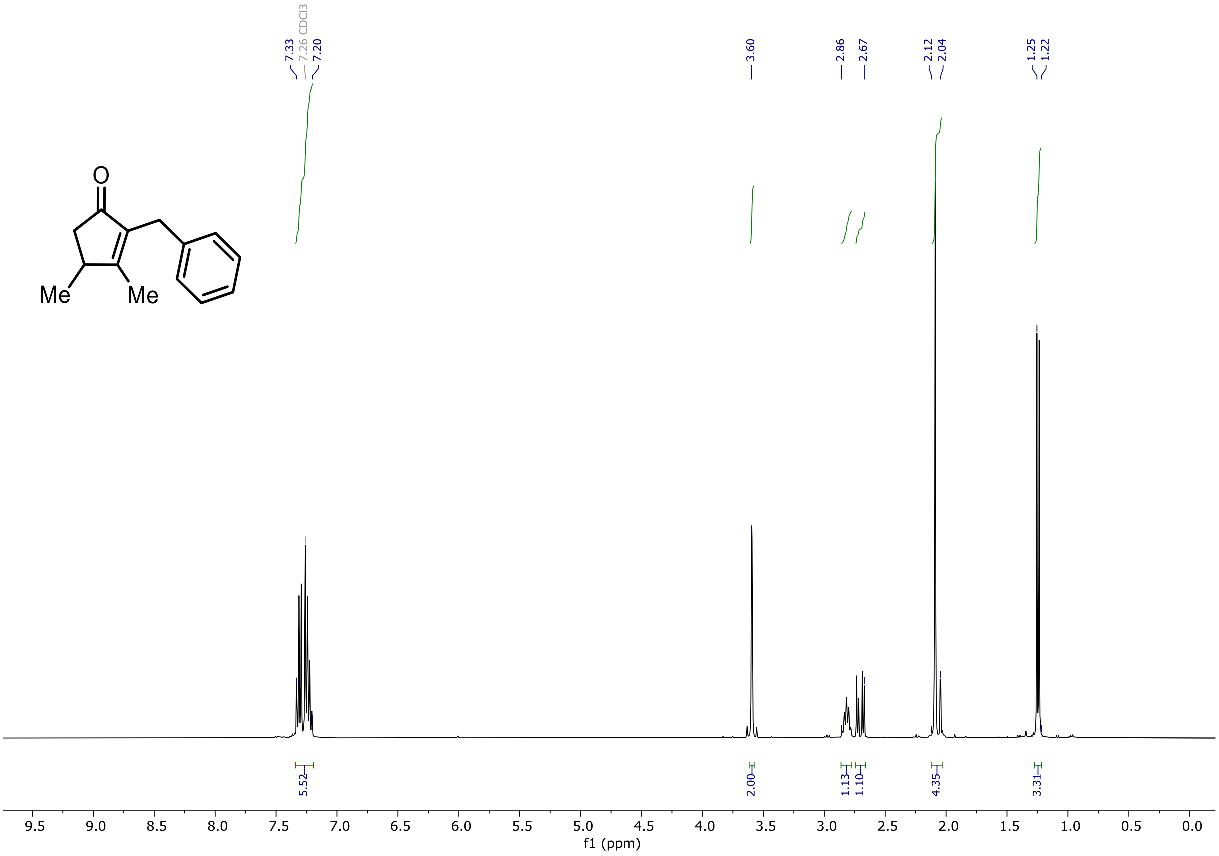

^{1}H NMR (400 MHz, CDCl$_3$) of ***2-benzyl-3,4-dimethylcyclopent-2-en-1-one (46)***

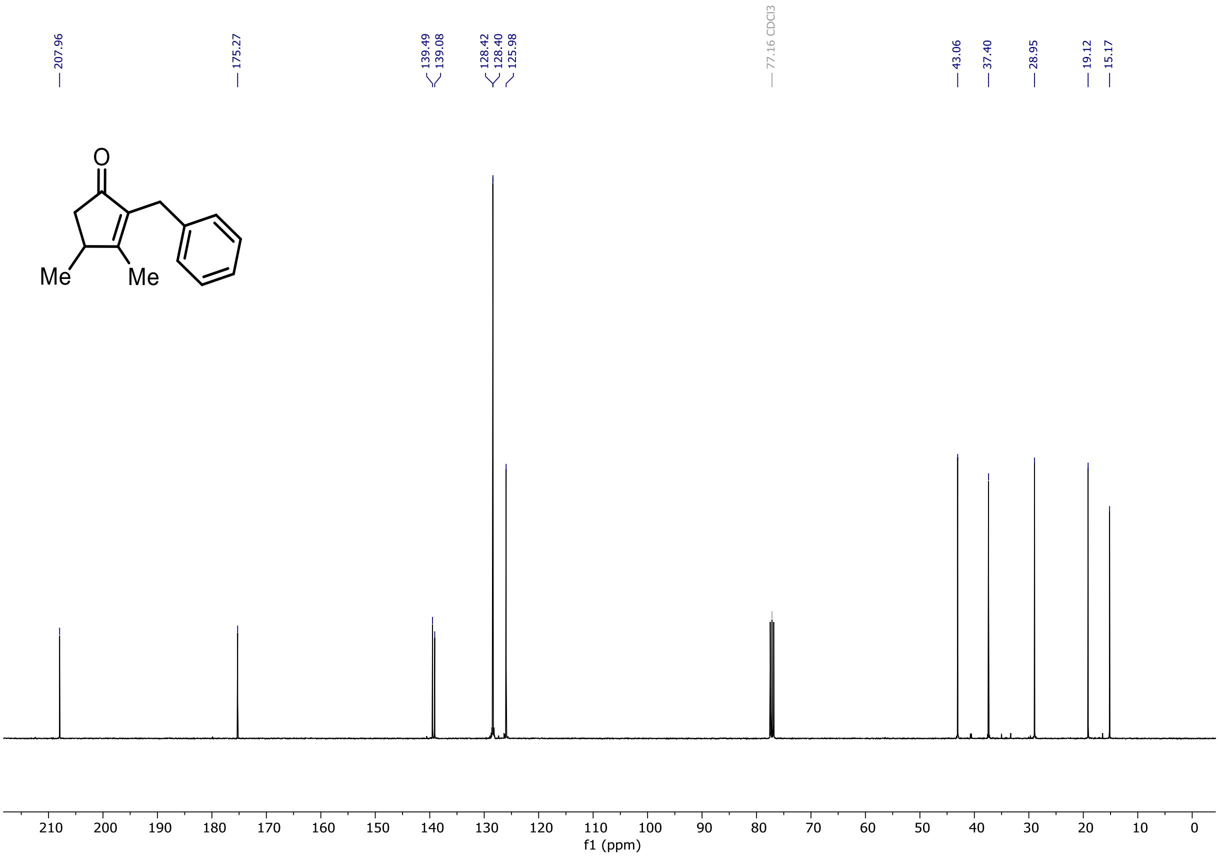

^{13}C NMR (101 MHz, CDCl$_3$) of ***2-benzyl-3,4-dimethylcyclopent-2-en-1-one (46)***

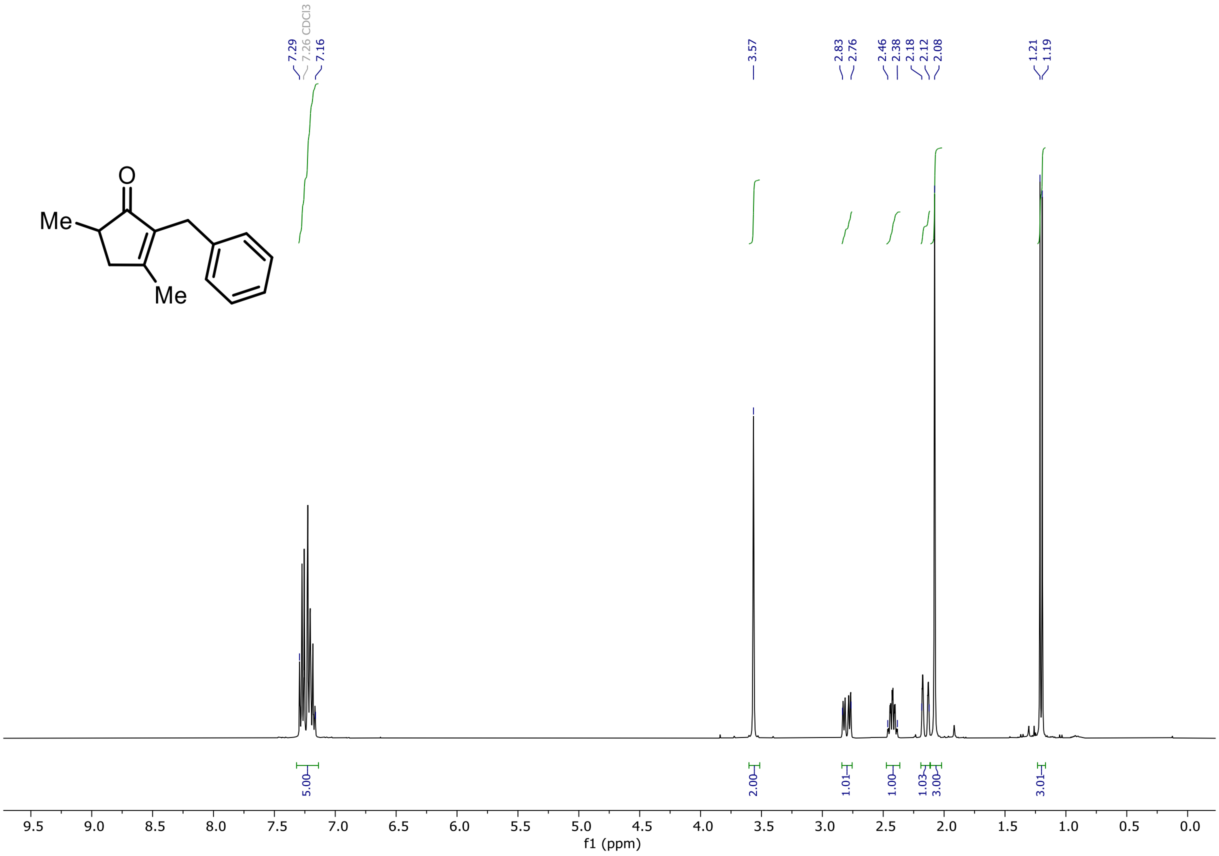

^{1}H NMR (400 MHz, $CDCl_3$) of ***2-benzyl-3,5-dimethylcyclopent-2-en-1-one (47)***

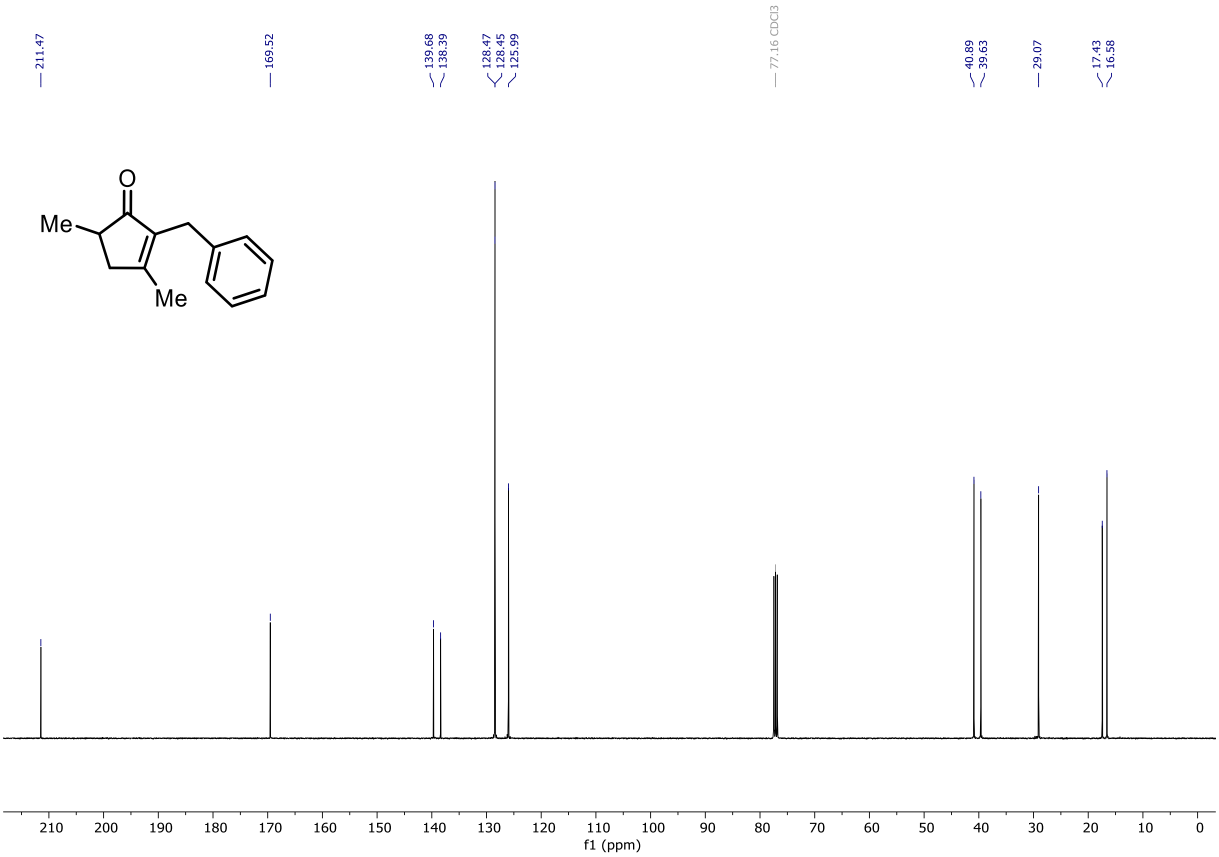

^{13}C NMR (101 MHz, $CDCl_3$) of ***2-benzyl-3,5-dimethylcyclopent-2-en-1-one (47)***

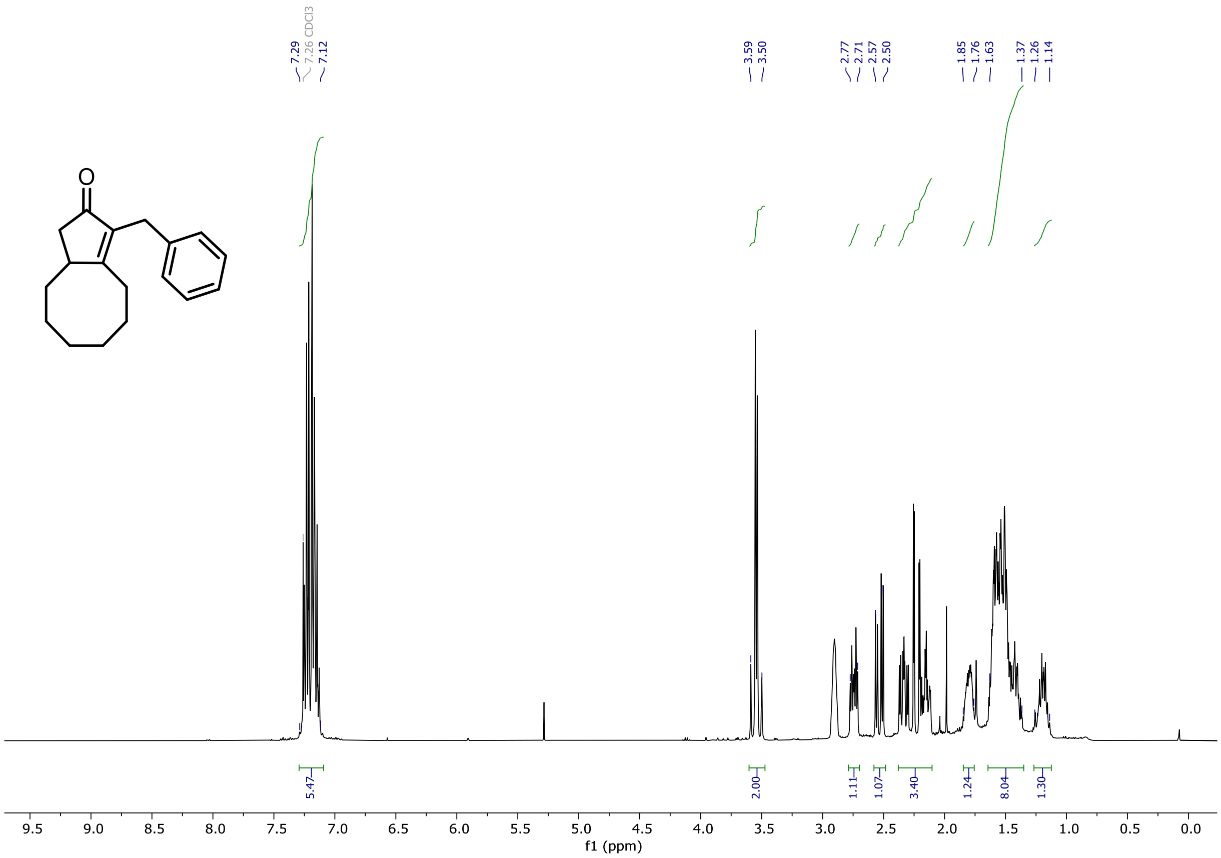

¹H NMR (400 MHz, $CDCl_3$) of ***3-benzyl-1,4,5,6,7,8,9,9a-octahydro-2H-cyclopenta[8]annulen-2-one (48)***

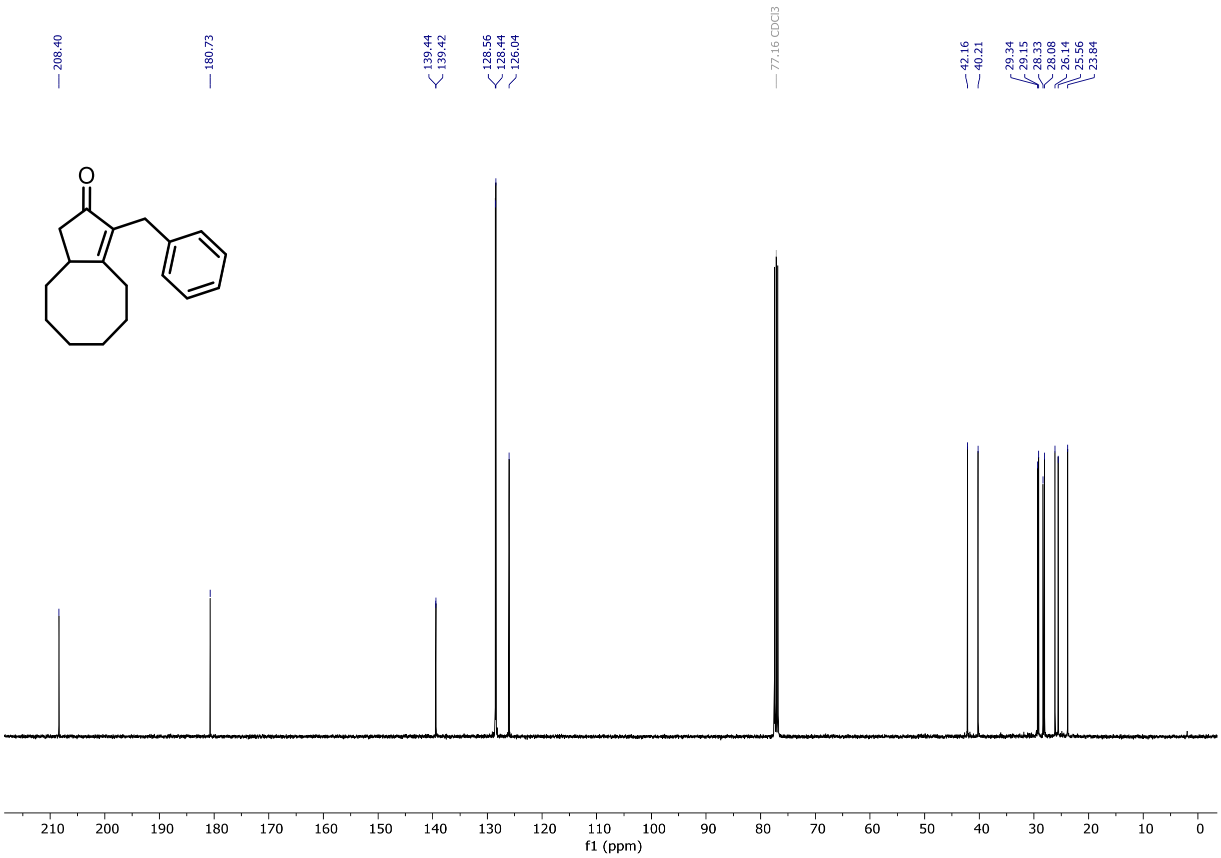

¹³C NMR (101 MHz, $CDCl_3$) of ***3-benzyl-1,4,5,6,7,8,9,9a-octahydro-2H-cyclopenta[8]annulen-2-one (48)***

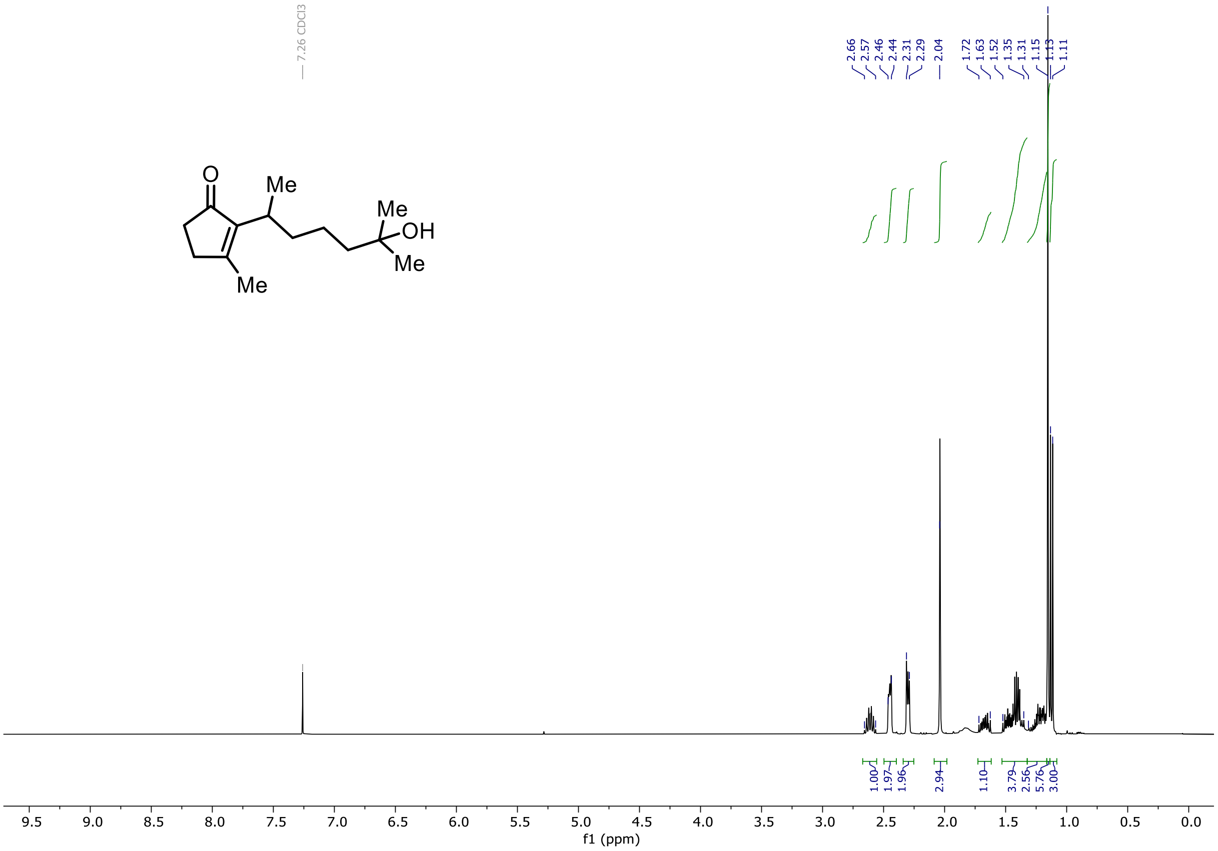

^{1}H NMR (400 MHz, $CDCl_3$) of ***2-(6-hydroxy-2,6-dimethylheptyl)-3-methylcyclopent-2-en-1-one (49)***

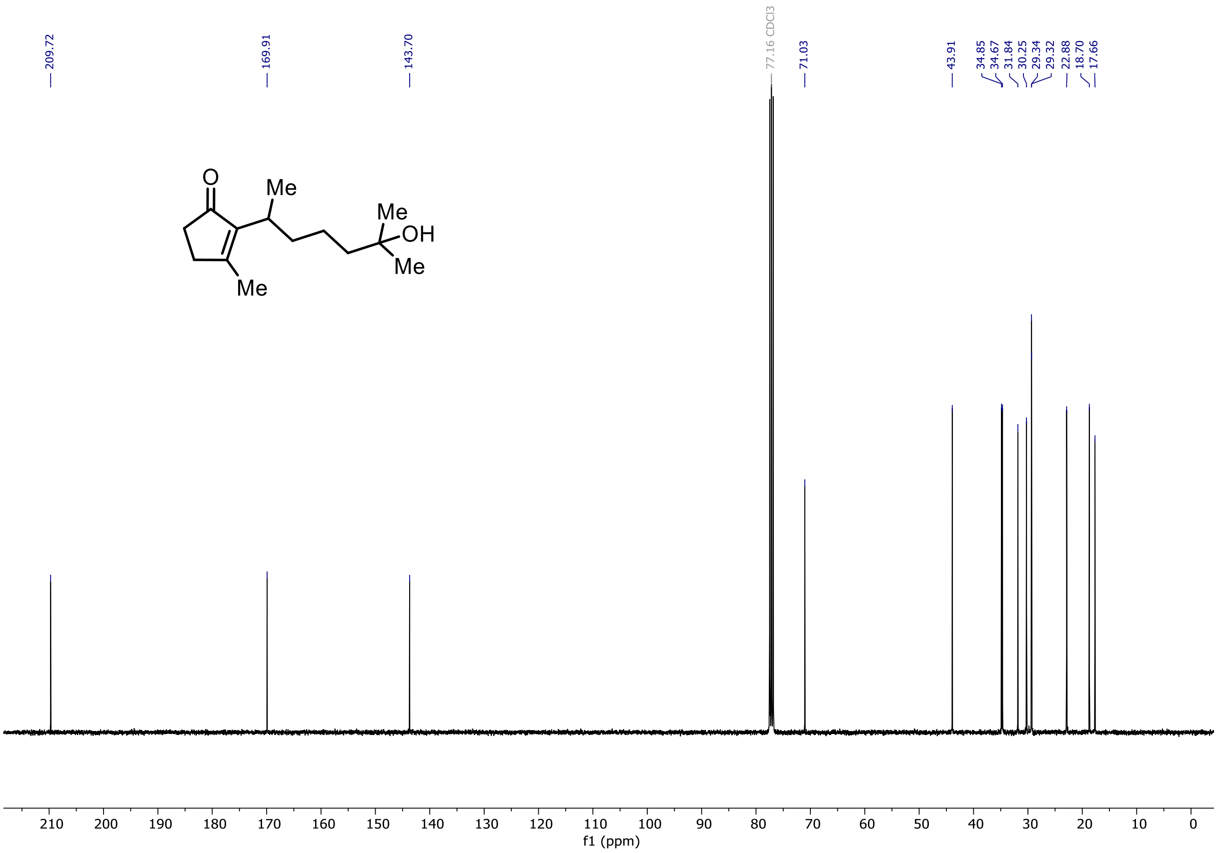

^{13}C NMR (101 MHz, $CDCl_3$) of ***2-(6-hydroxy-2,6-dimethylheptyl)-3-methylcyclopent-2-en-1-one (49)***

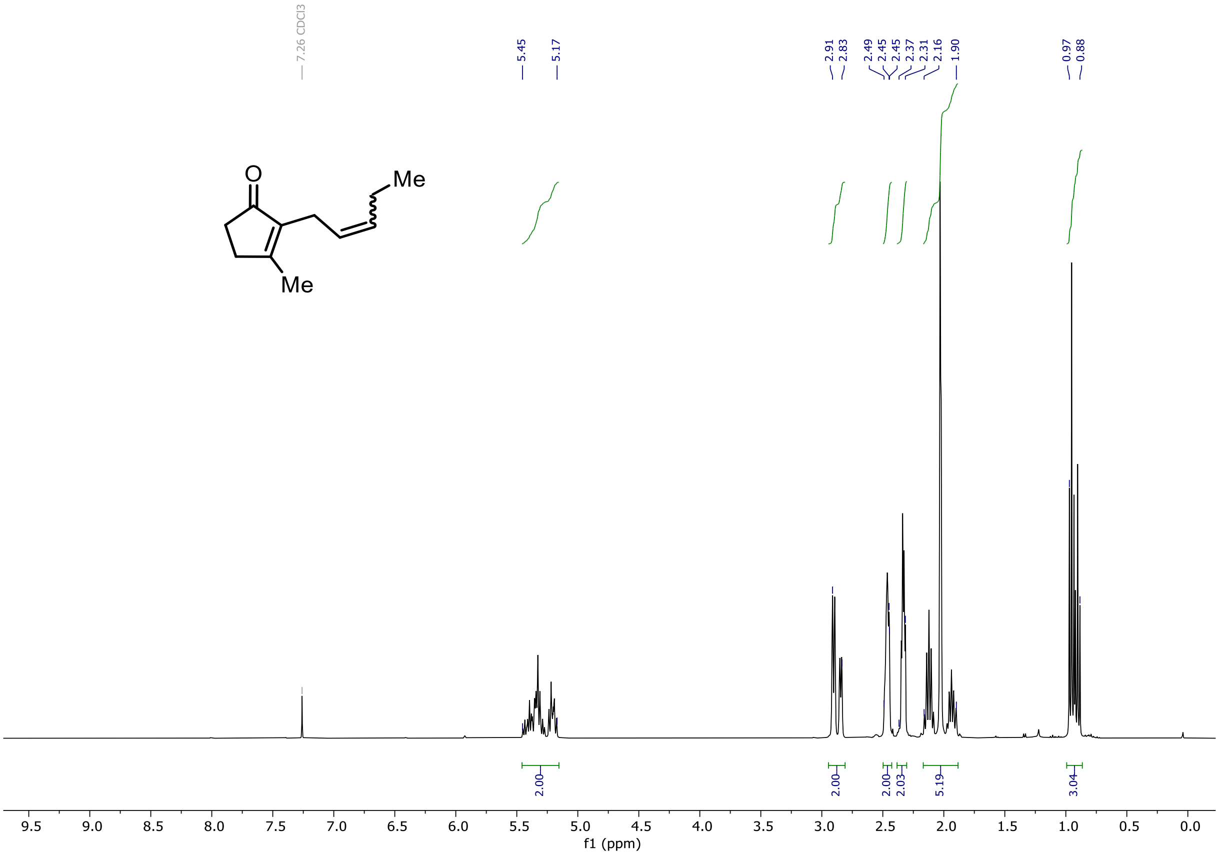

^{1}H NMR (400 MHz, CDCl$_3$) of ***3-methyl-2-(pent-2-en-1-yl)cyclopent-2-en-1-one (50)***

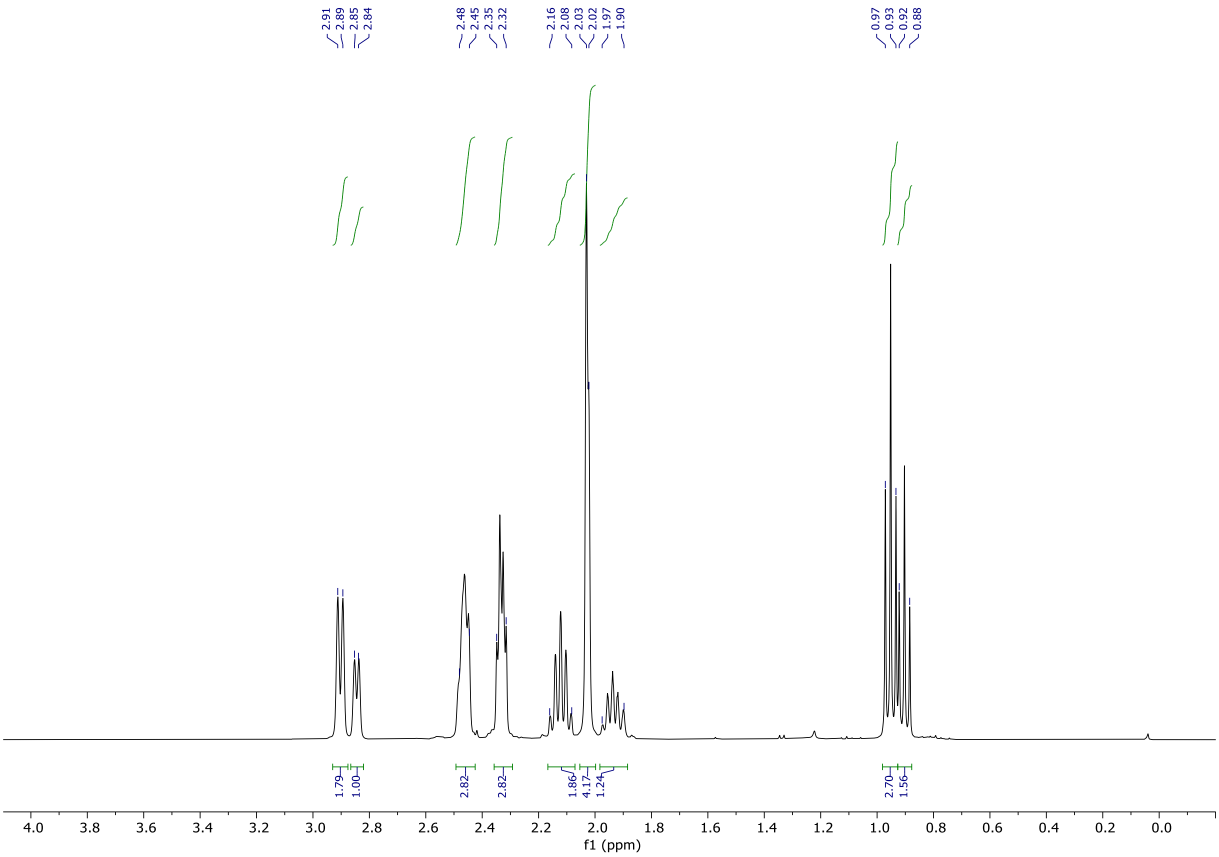

Inset of **^{1}H NMR (400 MHz, CDCl$_3$)** of ***3-methyl-2-(pent-2-en-1-yl)cyclopent-2-en-1-one (50)***

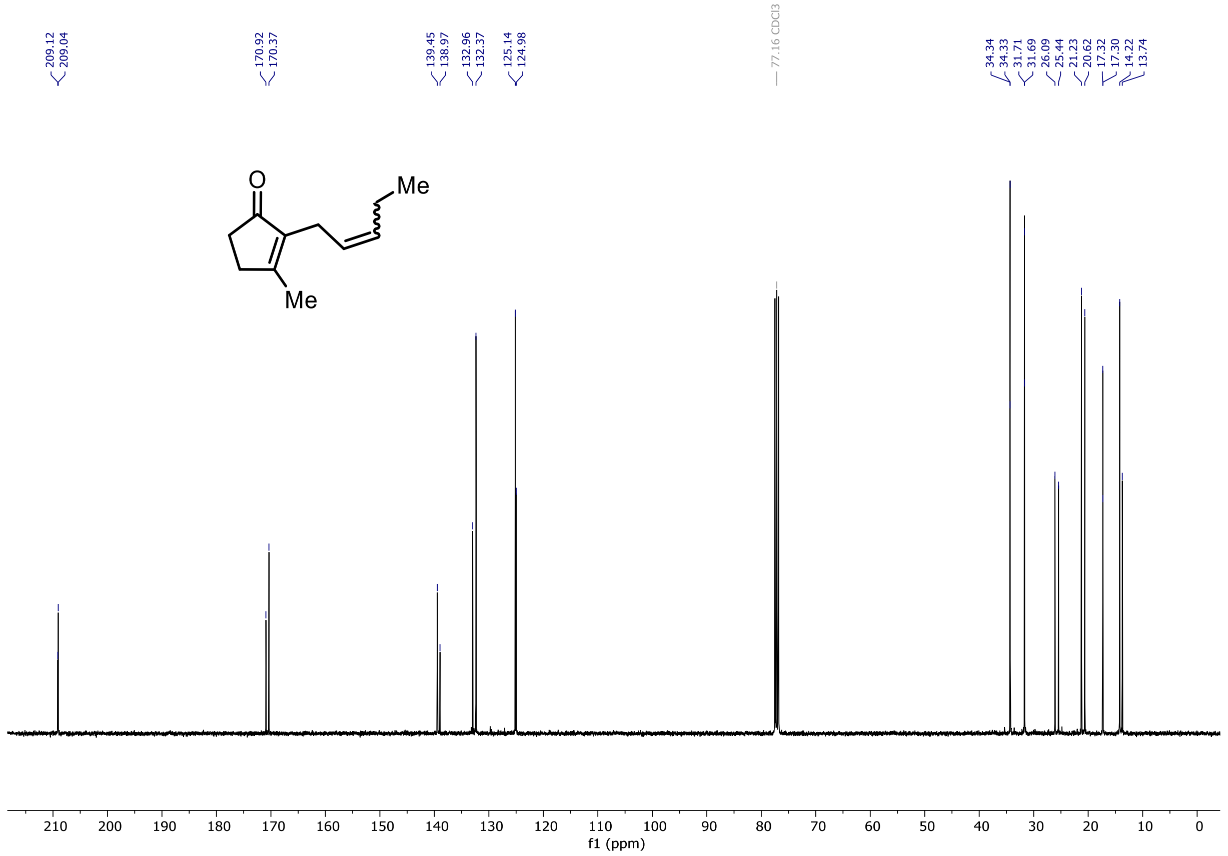

^{13}C NMR (101 MHz, CDCl$_3$) of ***3-methyl-2-(pent-2-en-1-yl)cyclopent-2-en-1-one (50)***

NOESY analysis for Z/E mixture of ***3-methyl-2-(pent-2-en-1-yl)cyclopent-2-en-1-one (50)***

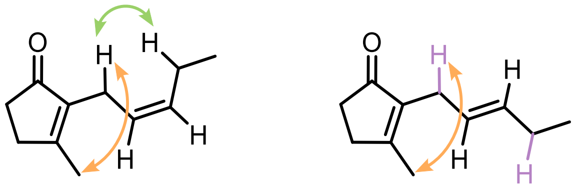

Isomer (*Z*), major   Isomer (*E*), minor

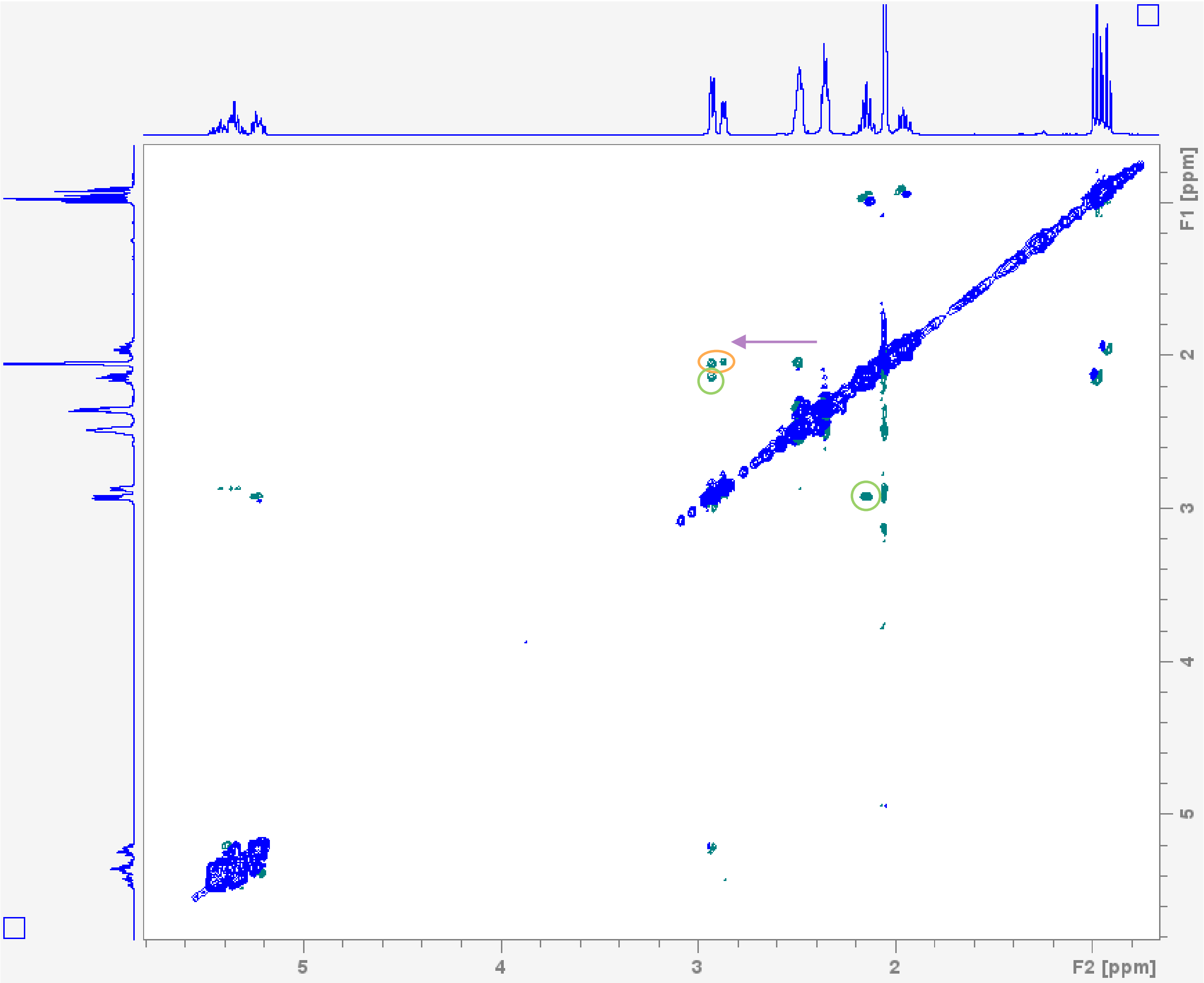

According to the NOESY analysis, we identified isomer (*Z*) as the major one from the 1.8:1 Z/E mixture (see ^{1}H NMR spectrum above). The signals of allylic hydrogens could be attributed unequivocally and helped to determine the stereochemistry of product (**50**). The correlation peaks highlighted in green are diagnostic signals of the (*Z*) isomer. On the other side, the lilac arrow indicates that there is no correlation peak between the corresponding hydrogens in the minor isomer, *i.e.* the (*E*) isomer. Moreover, the correlation peaks highlighted in orange are present in both the isomers, since the stereochemistry of the endocyclic double bond is fixed.
